# Rhodium-Catalyzed [2 + 2 + 2] Cyclotrimerizations of Yndiamides with Alkynes

**DOI:** 10.1021/acs.orglett.2c02770

**Published:** 2022-10-10

**Authors:** Philip J. Smith, Zixuan Tong, Julia Ragus, Pearse Solon, Kirk W. Shimkin, Edward A. Anderson

**Affiliations:** † Chemistry Research Laboratory, Department of Chemistry, University of Oxford, 12 Mansfield Road, OxfordOX1 3TA, U.K.; ‡ Discovery Chemistry, Therapeutics Discovery, Janssen Research & Development, LLC, Spring House, Pennsylvania19477, United States

## Abstract

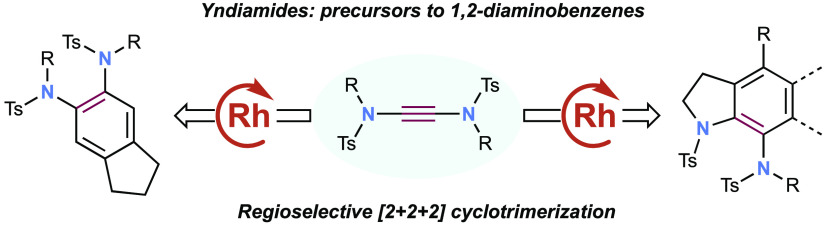

Yndiamides offer opportunities for the synthesis of vicinally nitrogen-disubstituted aromatics and azacycles. Here we report the Rh-catalyzed cyclotrimerization of alkynyl yndiamides with alkynes, the regiochemical outcome of which is controlled by the electronic properties of the alkyne partner, enabling the formation of 7-aminoindolines with excellent selectivity (up to >20:1 r.r.). We also report a complementary synthesis of bicyclic 1,2-dianiline derivatives by cyclotrimerization of yndiamides with terminal diynes, where slow addition of the diyne overcomes self-dimerization.

Transition metal-catalyzed [2 + 2 + 2] alkyne cyclotrimerizations are a powerful method for the synthesis of highly substituted benzene rings. Rhodium catalysts in particular have found widespread use in these 100% atom-economical processes.^1^ While the mechanistic aspects of these reactions have been widely studied,^2^ achieving regioselective [2 + 2 + 2] cyclotrimerizations remains a significant challenge. When applied to heteroatom-substituted alkynes, [2 + 2 + 2] cyclotrimerizations provide access to valuable aromatics featuring heteroatom substituents. Since Witulski’s pioneering use of alkynyl ynamides in Rh-catalyzed cyclotrimerizations (Scheme 1, eq 1),^3^ ynamides have been shown to undergo various transition metal-catalyzed [2 + 2 + 2] cyclotrimerization reactions to form aniline derivatives (Scheme 1, eq 2).^4^ However, as with other alkynes, the regioselectivity of nonsymmetric cyclotrimerizations can be variable.

**Scheme 1:**
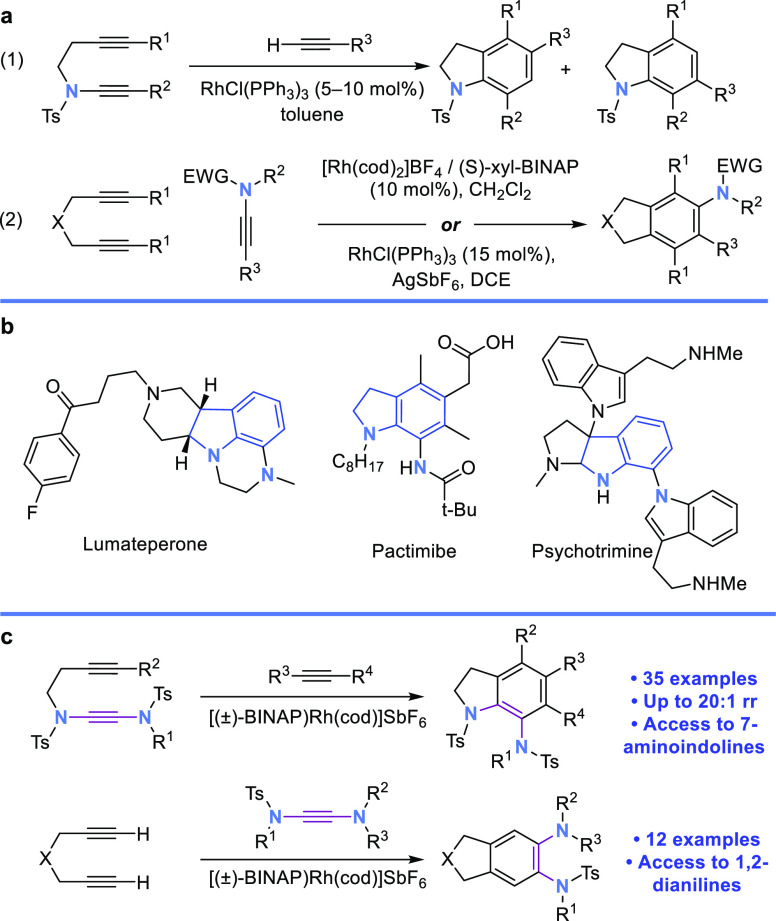
(a) [2 + 2 + 2] Cyclotrimerizations of Ynamides; (b) 7-Aminoindolines in Drug Molecules and Natural Products; (c) This Work: Intermolecular [2 + 2 + 2] Cyclotrimerizations of Yndiamides to Form 7-Aminoindolines and 1,2-Dianilines

Yndiamides (doubly nitrogen-substituted alkynes)^5^ offer unique possibilities for the synthesis of nitrogen-containing organic molecules.^6^ In the context of cyclotrimerization, we recognized that yndiamides could serve as precursors to 7-aminoindolines, valuable motifs that are found in a number of pharmaceuticals^7^ and natural products (Scheme 1b).^8^ However, aside from our initial report of a fully intramolecular yndiamide cyclotrimerization,^5^ no studies on yndiamides have been described, particularly in terms of controlling regioselectivity. Here we report the development of a two-component intermolecular yndiamide cyclotrimerization to form highly substituted 7-aminoindolines (Scheme 1c), many of which display exceptional regioselectivity based on electronic effects. We also describe the use of yndiamides as the monoalkyne component, which react with diynes to form 1,2-dianiline derivatives. Given the importance of indolines and anilines,^9^ this chemistry could find broad application, while also offering new insight into factors affecting regioselectivity in cyclotrimerization processes.

Investigations commenced with alkynyl yndiamide **1a** and 2-butyne-1,4-diol (Table 1), which underwent cyclotrimerization using Wilkinson’s catalyst (5 mol %) at 50 °C in toluene to form aminoindoline **2aa** in moderate yield (entry 1). By using a preformed cationic rhodium catalyst with a noncoordinating counterion,^10^ the yield of **2aa** was dramatically increased (entries 2, 3). Reducing the temperature resulted in lower conversion (entries 4, 5). THF and DCE were also suitable solvents (entries 6, 7), with the latter proving marginally better. Increasing the reaction concentration from 0.033 to 0.1 M further enhanced the yield to 92% (entry 8). Pleasingly, the transformation could also be performed on 1.0 mmol scale (of **1a**) to prepare **2aa** in 92% yield (0.56 g, entry 9). Analysis of the ^1^H NMR spectrum of **2aa** showed the *N*-benzyl protons to be diastereotopic, suggesting that **2aa** features restricted rotation about the C–N axis. This was confirmed by variable temperature NMR (in DMSO-*d*_6_), in which partial coalescence of the *N*-benzyl protons was observed. (See the Supporting Information for details.) Subjection of racemic **2aa** to dynamic chiral HPLC (15–50 °C) enabled the calculation of an inversion barrier of 21.9 kcal mol^–1^.^12^ Equating to a half-life of 1260 s at 298 K, this barrier renders **2aa** formally atropisomeric according to Oki’s definition,^13^ and a “Class 2” atropisomer according to the categorization system of LaPlante et al.^14^ Atropisomeric molecules are of increasing importance in drug discovery, with new methods to prepare C–N axes with restricted rotation being of especial interest.^15^

**Table 1:**
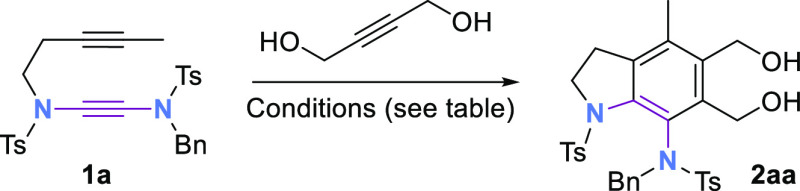
Optimization of the [2 + 2 + 2] Cyclotrimerization of Yndiamide 1a with 2-Butyne-1,4-diol^a^,^b^

entry	catalyst	solvent	temp (°C)	yield (%)^c^
1	(Ph_3_P)_3_RhCl	PhMe	50	36
2	[Rh]-1	PhMe	50	82
3	[Rh]-2	PhMe	50	82
4	[Rh]-2	PhMe	40	25
5	[Rh]-2	PhMe	r.t.	<5
6	[Rh]-2	THF	50	80
7	[Rh]-2	DCE	50	86
**8**	**[Rh]-2**	**DCE**^d^	**50**	**90 (92)**^e^
**9**^f^	**[Rh]-2**	**DCE**^d^	**50**	**(92)**^e^

a [Rh]-1 = [(±)-BIPHEP)Rh(cod)]SbF_6_; [Rh]-2 = [(±)-BINAP)Rh (cod)]SbF_6_.

b Reactions conducted on 0.05 mmol scale under Ar for 16 h, 5 mol % catalyst loading, 0.033 M.

c Yields determined by quantitative ^1^H NMR spectroscopy using 1,3,5-trimethoxybenzene as an internal standard.

d 0.100 M.

e Isolated yield.

f 1 mmol scale (of **1a**).

With optimized conditions established, we investigated the scope of the [2 + 2 + 2] cyclotrimerization using yndiamide **1a** and a range of monosubstituted and disubstituted alkynes (Scheme 2a). Aryl alkynes reacted with **1a** to give arylated indolines **2ab**–**2aq** in good to excellent yields in most cases. Excellent regioselectivity was observed for electron-rich aryl and heteroaryl alkynes (**2ab**–**2ai**), whereas electron-poor substrates proceeded with modest selectivity (**2aj**–**2ap**). Steric effects also appeared influential, with *o*-methoxyphenylacetylene giving **2ao** with inferior selectivity (2:1 r.r.) compared to the *m*- and *p*-methoxy isomers (4:1 and 8:1 r.r. respectively). A plot of the percentage of the major regioisomer against the Hammett substituent constant revealed a clear trend, with the highest regioselectivity achieved with the most electron-rich aryl groups.

**Scheme 2:**
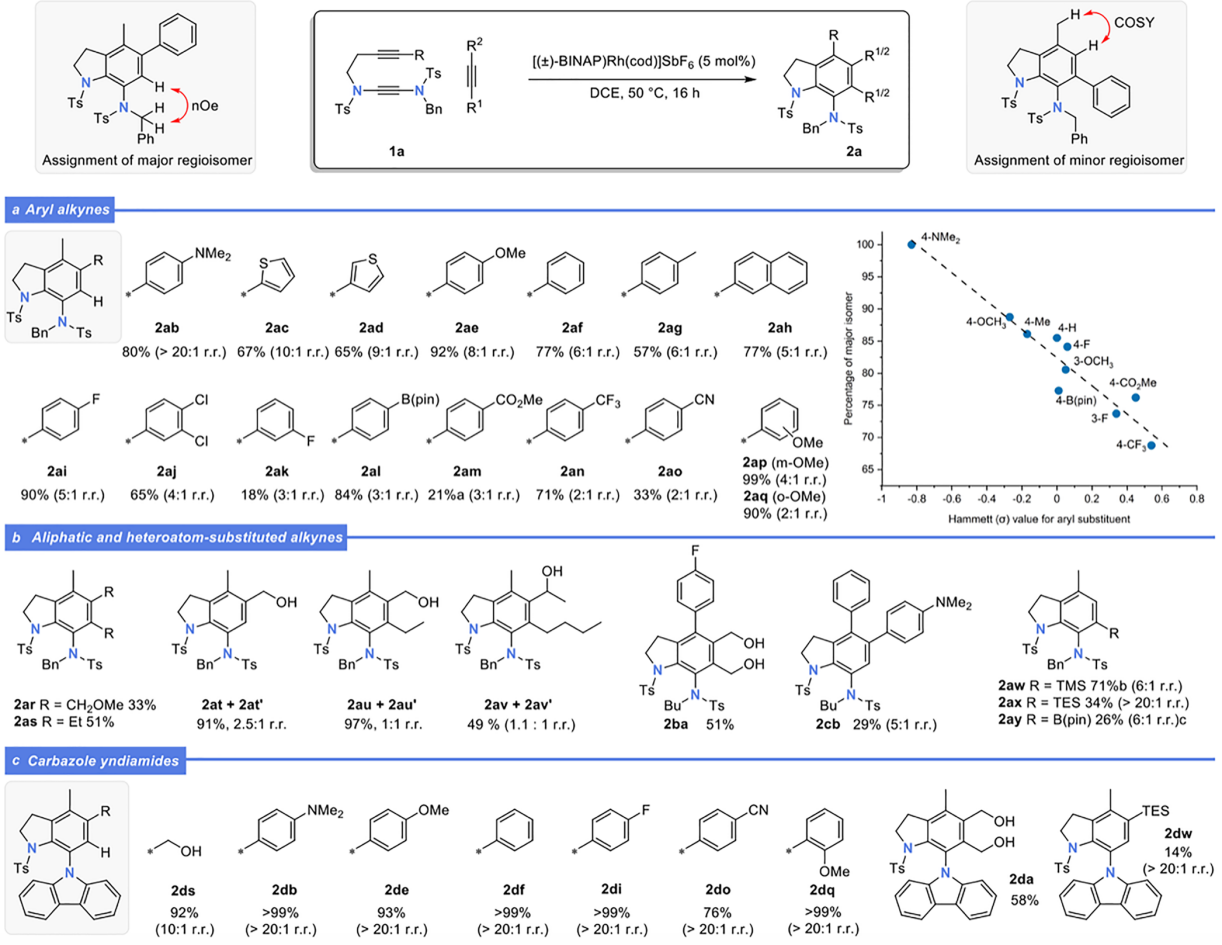
Scope of [2 + 2 + 2] Cyclotrimerization Reaction between Alkynyl Yndiamides and Alkynes Reactions carried out on a 0.1 mmol scale under Ar. Yields are isolated yields. Regioisomeric ratios determined from ^1^H NMR spectroscopic analysis of the crude reaction mixture. NMR yield. 10.0 equiv of TMS acetylene, conc = 0.5 M, sealed tube. Isolated yield of the regioisomer shown.

Aliphatic alkynes were also found to be competent substrates (Scheme 2b), forming indolines **2ar**–**2av** in moderate to good yields, although for nonsymmetrical alkynes low regioselectivity was observed. Other examples include the use of aryl-alkyne yndiamides (**2ba**, **2cb**) and of heteroatom-substituted alkynes as the monoalkyne component (**2aw**–**2ay**). For the latter, trimethylsilylacetylene and triethylsilylacetylene gave the silylated aminoindolines **2aw** and **2ax** with moderate and excellent regioselectivities, respectively, while Bpin-acetylene afforded borylated aminoindoline **2ay**, which contains a valuable boronic ester handle for further manipulation of the aminoindoline product.

The yndiamides examined thus far all featured sulfonamides at the alkyne terminus. To explore the influence of this group on regioselectivity, we next studied the cyclizations of carbazole yndiamide (**1d**), which notably represents a novel class of yndiamide (Scheme 2c). To our delight, this substrate underwent exceptionally regioselective and efficient cyclotrimerizations with aryl alkynes, with a single regioisomer formed in all cases. These results are intriguing given the variable regioselectivity observed for bis-sulfonamide yndiamides; for **1d**, the alkyne substituent appears to have a minimal effect, which may be due to the bulkier nature of the carbazole compared to the sulfonamide, or to differing electronic influence. Reaction of **1d** with 2-butyne-1,4-diol delivered the hexasubstituted indoline-carbazole **2da** in respectable yield, while reaction with triethylsilylacetylene led to a complete switch in regioselectivity compared to yndiamide **1a**, albeit proceeding in poor yield (**2dw**).

Having demonstrated the viability of alkynyl yndiamides as the “diyne” component in Rh-catalyzed two component [2 + 2 + 2] cyclotrimerizations, we next addressed cyclotrimerizations in which the yndiamide serves as the monoalkyne component. We expected this reaction to be more challenging, since di/trimerization of the less-hindered diyne component would be expected to compete with the desired cross-coupling. Specifically, the yndiamide would be required to preferentially intercept the putative metallacyclopentadiene intermediate arising from oxidative coupling with the diyne (see mechanistic discussion below). Initial investigations using yndiamide **6a** and 1,6-heptadiyne gave none of the desired product **7aa** (Table 2, entries 1–4), with dimerization of 1,6-heptadiyne indeed dominating.^16^ However, slow addition of the diyne to a solution of yndiamide and a preformed cationic Rh(I) catalyst enabled formation of **7aa** in low yield (entry 5). Adjusting the rate of addition of 1,6-heptadiyne and the reaction concentration increased the yield of **7aa** to 78% (entries 6–8).

**Table 2:**
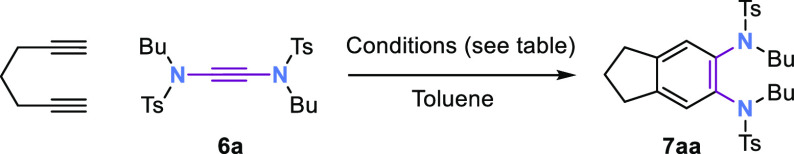
Optimization of Conditions for Cyclotrimerization of Yndiamide 6a with 1,6-Heptadiyne^a^

entry	catalyst	**6a**: diyne	*T* (°C)	time (h)	yield (%)^b^
1	(PPh_3_)_3_RhCl/AgSbF_6_^c^	2:1	85	16	0
2	Rh_2_(C_2_H_4_)Cl_2_/AgSbF_6_/*rac*-BINAP	1:10	100	16^d^	<5
3	Cp*Ru(cod)Cl	1:10	100	1.5^d^	0
4	[Ru(*p*-cymene)Cl_2_]_2_	1:10	100	1.5^d^	0
5	[Rh]-2	1:10	85	1.5^d^	11
6^e^	[Rh]-2	1:10	85	16^d^	45
7	[Rh]-2	1:10	100	1.5^d^	51
8	[Rh]-2	1:5	100	4^f^	62
9^g^	[Rh]-2	1:5	100	4^f^	78

a Reactions conducted under an Ar atmosphere, on a 0.05 mmol scale with 10 mol % catalyst, with a final concentration of 0.05 M after addition of the diyne, unless stated otherwise.

b Yields determined by quantitative ^1^H NMR spectroscopy using 1,3,5-trimethoxybenzene as internal standard.

c Reaction conducted in DCE using 20 mol % catalyst.

d Addition of diyne over 1 h.

e Final reaction concentration 0.01 M.

f Addition of diyne over 1.5 h.

g Final reaction concentration 0.1 M.

We were pleased to find that a range of terminal diynes were competent substrates in this second cyclotrimerization (Scheme 3), forming 1,2-dianiline derivatives **7aa**–**7ea** and **7ba** in low to excellent yields. Carbazole yndiamides **6c**–**6f** also underwent cyclotrimerization with 1,6-heptadiyne, forming *N*-arylated carbazoles **7ac**–**7af**, with variation of the linker group also tolerated (**7cc**). The transformation was successfully carried out on a 1.0 mmol scale to give **7ab** in 58% yield (0.37 g). Disappointingly, no product formation was observed when nonterminal diynes were employed, with only dimerization and trimerization of the diyne component occurring. Procedures for the cyclotrimerization of more substituted diynes with ynamides are known;^4a−4e^ however, attempts to apply these protocols to our system were unsuccessful. Analysis of the ^1^H NMR spectrum of **7ab** showed significant broadening of the peaks corresponding to the *N*-benzyl protons; analysis of this compound by variable temperature ^1^H NMR spectroscopy suggested that this compound also exhibits restricted rotation around the C–N axes.

**Scheme 3:**
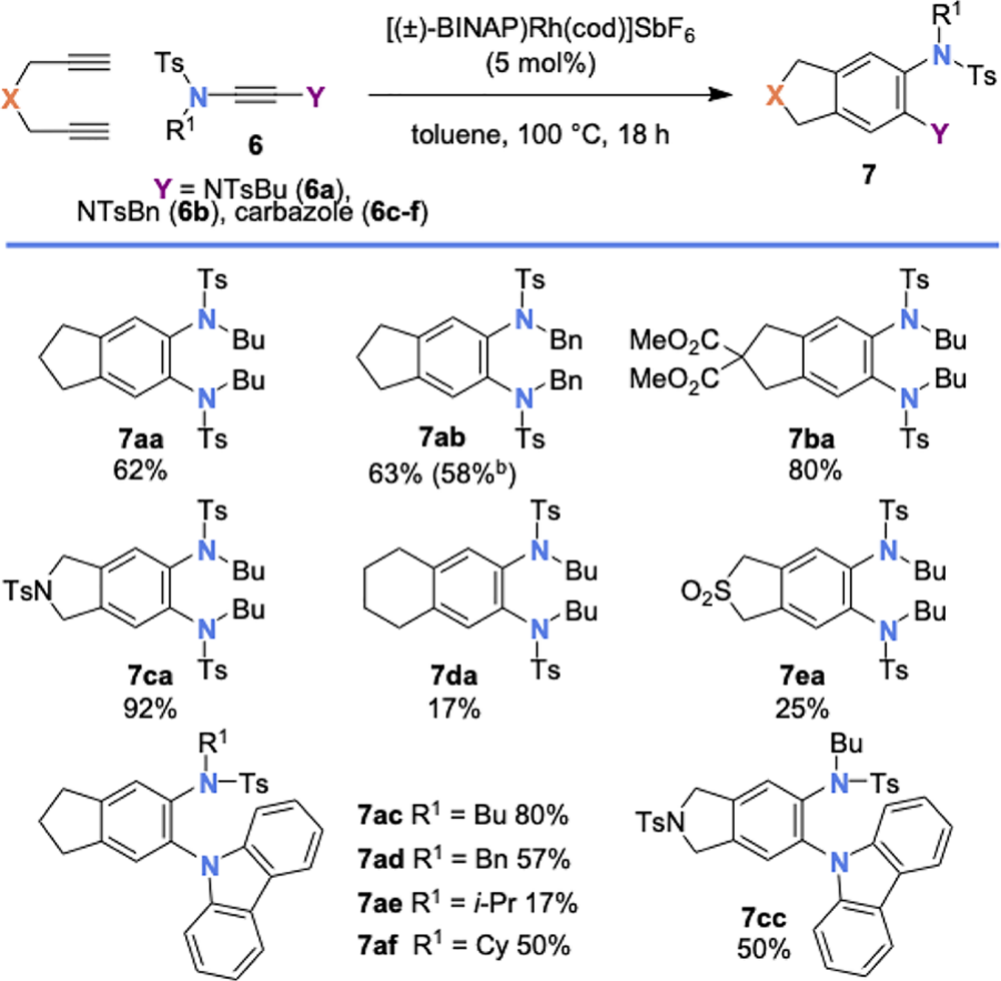
Scope of [2 + 2 + 2] Cyclotrimerization Reaction between Yndiamides and Diynes Reactions carried out on a 0.1 mmol scale under an Ar atmosphere. Yields are isolated yields. Reaction carried out on a 1.0 mmol scale (of **6a**).

Scheme 4 shows a proposed mechanism for the Rh-catalyzed [2 + 2 + 2] cyclotrimerization of alkynyl yndiamides **1** with alkynes. Oxidative cyclization of **1** with the cationic Rh(I) catalyst leads to formation of rhodacyclopentadiene **A**, which is followed by coordination of the monoalkyne to form complex **B**.^17^ This complex undergoes a formal [5 + 2] cycloaddition with the alkyne to form intermediate **C** or **C′** (either of which can lead to the same regioisomer of product **2**),^18^ which then converts to rhodacycloheptatriene intermediates **D** or **D′**. The regioselectivity of alkyne insertion depends on the selectivity of both these steps. From a steric perspective, orientation of the alkyne to position its substituent remote from the metal center may be favored (as in **C**); tighter binding of a more electron-rich alkyne might enhance this steric effect. This structure may also be favored for electron-rich aryl alkynes, which can thereby better donate electron density to the metal center through the cyclobutene π system.^19^ Reductive elimination from **D** (or **D′**) gives the product coordinated to Rh(I) (**E**), dissociation of which liberates arene and re-forms the cationic Rh(I) catalyst.

**Scheme 4:**
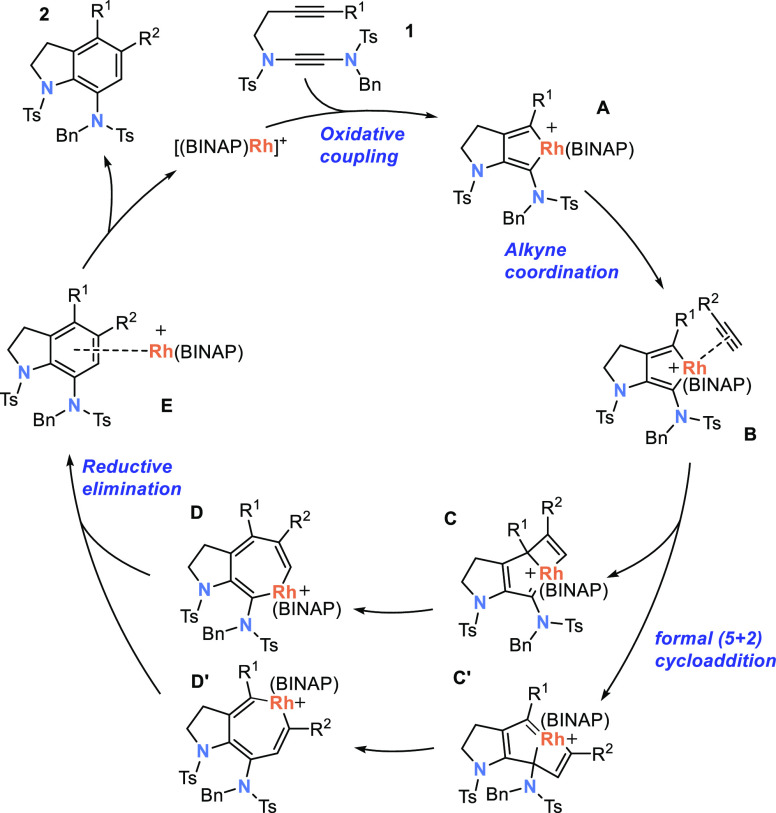
Proposed Mechanism for [2 + 2 + 2] Cyclotrimerization to Form the Major Regioisomer of Product 2

In conclusion, two [2 + 2 + 2] cyclotrimerization reactions of yndiamides with alkynes have been developed; in the first instance, the yndiamide is contained within the diyne component, giving 7-azaindoline products, and in the second, the yndiamide comprises the monoalkyne component, giving 1,2-dianilines on reaction with terminal diynes. Both reactions proceed efficiently using a bench-stable rhodium catalyst and are tolerant of various functional groups, giving opportunities for further product derivatization. The restricted rotation observed around the C–N axis in several of the products may be of interest for applications in medicinal chemistry, where the discovery of new atropisomeric compounds is a vibrant area of research.

## Notes

Data underlying this study are available in the published article and its online Supporting Information.

## Supporting Information Available

Experimental procedures, characterization data, and copies of NMR spectra (PDF)
