# Supplementary material for: Rhodium-Catalyzed [2 + 2 + 2] Cyclotrimerizations of Yndiamides with Alkynes
Source: Org Lett. 2022 Oct 10;24(41):7522–6. doi: 10.1021/acs.orglett.2c02770 (PMC9594354; doi:10.1021/acs.orglett.2c02770)
Supplement: Supplementary file 1 — ol2c02770_si_001.pdf [file ol2c02770_si_001.pdf]

Rhodium-catalyzed [2+2+2] Cyclotrimerizations of Yndiamides with Alkynes

Philip J. Smith,[a] Zixuan Tong,[a] Julia Ragus,[a] Pearse Solon,[a] Kirk W. Shimkin,[b] Edward A. Anderson [a,*]

E-mail: edward.anderson@chem.ox.ac.uk

[a] Chemistry Research Laboratory, 12 Mansfield Road, Oxford, OX1 3TA, UK.

[b] Discovery Chemistry, Therapeutics Discovery, Janssen Research & Development, LLC, Spring House, Pennsylvania 19477, United States

Supporting Information

1. General Procedures **S2**

2. Synthesis of starting materials **S6**

3. [2+2+2] Cyclotrimerization products **S12**

4. NMR Spectra of novel compounds **S48**

5. Dynamic HPLC Data **S108**

6. References **S110**

1. General Procedures

Reactions requiring heating were heated using an oil bath.

Unless stated otherwise, all chemicals were purchased from commercial suppliers (Sigma-Aldrich, Fluorochem, Alfa Aesar, TCI) and used without further purification.

^{1}H, ^{13}C, ^{31}P, and ^{19}F NMR spectra were recorded on a Bruker AVIII400 Spectrometer (400 MHz, 101 MHz, 162 MHz, and 377 MHz respectively) or a Bruker AVII500 (^{1}H: 500 MHz and 13C: 126 MHz) in $CDCl_3$, DMSO-d_6 or MeOD and referenced to residual solvent peaks. Chemical shifts δ are quoted in parts per million (ppm) to the nearest 0.01 for ^{1}H and 0.1 for ^{13}C (for ^{13}C in cases where two peaks have the same chemical shift to the nearest 0.1 ppm, shifts are given to the nearest 0.01 ppm), coupling constants *J* are quoted in Hz to the nearest 0.1 and splitting are recorded as singlet (s), doublet (d), triplet (t), quartet (q), pentet (p), doublet of a doublet (dd), doublet of a doublet of a doublet (ddd), broad singlet (br. s) and multiplet (m), or apparent (app) splitting corresponding to one of the above patterns. Assignments were based upon COSY, HSQC, NOESY and HMBC experiments. Chemical shifts and splitting patterns are recorded as observed.

Infrared spectra were recorded on a Bruker Tensor 27 FT-IR spectrometer fitted with an Attenuated Total Reflectance (ATR) sampling accessory. Absorption maxima are quoted in wavenumbers (cm-1). Absorption maxima with wavenumbers >1000 cm^{-1} are quoted.

Mass spectra were recorded on a Fisons Platform II spectrometer under electrospray ionisation (ESI). High resolution mass spectra are given to four decimal places and were recorded on a Bruker MicroTof (resolution = 10000 FWHM).

Melting points (MP) were obtained using a Gallenkamp melting point machine.

Analytical thin layer chromatography (TLC) was performed on pre-coated SiO2 aluminium sheets from Merck (TLC Silica Gel 60 F254s). Spots were visualised either by the quenching of UV fluorescence or by staining with vanillin solution, KMnO4 solution, ninhydrin solution or PMA solution. Retention factor (Rf) values are given for compounds formed in reactions which were monitored by TLC.

Preparative flash column chromatography was carried out using Geduran Silica Gel 60 (40–63 μm) from Merck.

All compounds were named using PerkinElmer ChemDraw software and key atoms were numbered for ease of data interpretation.

General Procedure 1: Rh-catalyzed cyclotrimerization reactions of yne-yndiamides with alkynes

Yndiamide (0.10 mmol, 1.0 equiv.), (±)-(BINAP)Rh(cod)SbF_6 (5.2 mg, 0.050 mmol, 5 mol%), and alkyne (0.30 mmol, 3.0 equiv.) were added to an oven-dried screw cap vial containing a stirrer bar and fitted with a septum. The vial was then evacuated under high vacuum and backfilled with argon three times, then 1,2-dichloroethane (1 mL, anhydrous) was added by syringe. The septum was then replaced quickly with a screw cap and the reaction mixture was stirred at 50 °C for 16 h or until complete consumption of the yndiamide starting material was confirmed by TLC. The solvent was then removed *in vacuo* and a crude ^{1}H NMR spectrum was obtained to measure the regioisomeric ratio where appicable. The crude material was then purified by flash column chromatography.

General Procedure 2: Rh-catalyzed cyclotrimerization reactions of yndiamides with diynes

Yndiamide (0.10 mmol, 1.0 equiv.) and (±)-(BINAP)Rh(cod)SbF_6 (5.2 mg, 0.050 mmol, 5 mol%) were added to an oven-dried vial containing a stirrer bar. The vial was evacuated under high vacuum and backfilled with argon three times, then toluene (0.2 mL, anhydrous) was added by syringe. In a second vial under an atmosphere of argon the diyne (0.50 mmol, 5.0 equiv.) was dissolved in toluene (0.8 mL, anhydrous). The first vial was heated at 100 °C under an atmosphere of argon and the diyne solution was added to the reaction mixture by syringe pump over 3 h, then the reaction mixture was stirred for an additional 15 h at 100 °C. The reaction mixture was then cooled to room temperature, diluted with $CHCl_3$, and concentrated *in vacuo*. The crude material was then purified by flash column chromatography.

General Procedure 3: Synthesis of yndiamides[1]

To an oven-dried flask equipped with a stirrer bar were added sulfonamide (1.0 equiv.), 1,1-dibromoenamide (1.1 equiv.), 1,10-phenanthroline (40 mol%), CuI (20 mol%, dispensed in N_2-filled glovebox), and Cs_2CO_3 (3.0 equiv., dispensed in N_2-filled glovebox). The vial was then capped with a septum and evacuated under high vacuum for 10 minutes. The vial was then backfilled with argon and the cycle was repeated twice more. Anhydrous THF (3 mL per mmol sulfonamide) was added and the reaction mixture was stirred at 60 °C overnight or until completion was observed by TLC. The reaction mixture was cooled to room temperature and diluted with EtOAc and filtered through a short Celite pad. The crude material was then concentrated in vacuo and purified by flash column chromatography.

Synthesis of [Rh(cod)$_2$]SbF_6 [2]

[Rh(cod)Cl]$_2$ (0.156 g, 0.30 mmol, 1.0 equiv.) was dissolved in CH_2Cl_2 (2.0 mL) under an atmosphere of argon and cyclooctadiene (55 µL, 0.45 mmol, 1.5 equiv.) was added. Separately, a solution of $AgSbF_6$ (0.232 g, 0.68 mmol, 2.3 equiv.) in acetone (1.0 mL) was prepared and was added to the first solution. The reaction mixture was then stirred at room temperature for 20 minutes followed by filtration through Celite (washing with THF). The filtrate was then concentrated *in vacuo* and washed with cold Et_2O (5 × 1 mL) and the resulting solid was dried under high vacuum to give the title compound (0.149 g, 0.27 mmol, 45%) as a deep red solid.

Synthesis of (±)-(BINAP)Rh(cod)SbF_6

[Rh(cod)$_2$]SbF_6 (0.149 g, 0.27 mmol, 1.0 equiv.) and *rac*-BINAP (0.168 g, 0.27 mmol, 1.0 equiv.) were dissolved in anhydrous CH_2Cl_2 (7.5 mL) and the reaction mixture was stirred at room temperature for 1 h. The solvent was removed *in vacuo* and the solid was washed with Et_2O (5 × 1 mL) and dried under high vacuum to give the title compound (0.230 g, 0.22 mmol, 80%) as an orange solid.

Determination of regioisomeric ratio in products 2ab–2aq

The regioisomeric ratio for the cyclotrimerization of yne-yndiamides with aryl alkynes was determined by analysis of the crude reaction mixture ^{1}H NMR spectra (400 MHz, $CDCl_3$). Typically, the *N*-benzyl CH_2 protons (approx. 5.4–4.6 ppm) were found to give a good measure of the ratio between the two regioisomers. Analysis of the purified regioisomeric mixtures by examining NOESY, HMBC and COSY correlations allowed the major product to be identified. An example is given below for the reaction of yne-yndiamide **1a** with phenylacetylene to form **2af** in a 6:1 regioisomeric ratio, identification of the major isomer is shown from the NOESY spectrum and identification of the minor isomer is shown from the COSY and HMBC spectra.

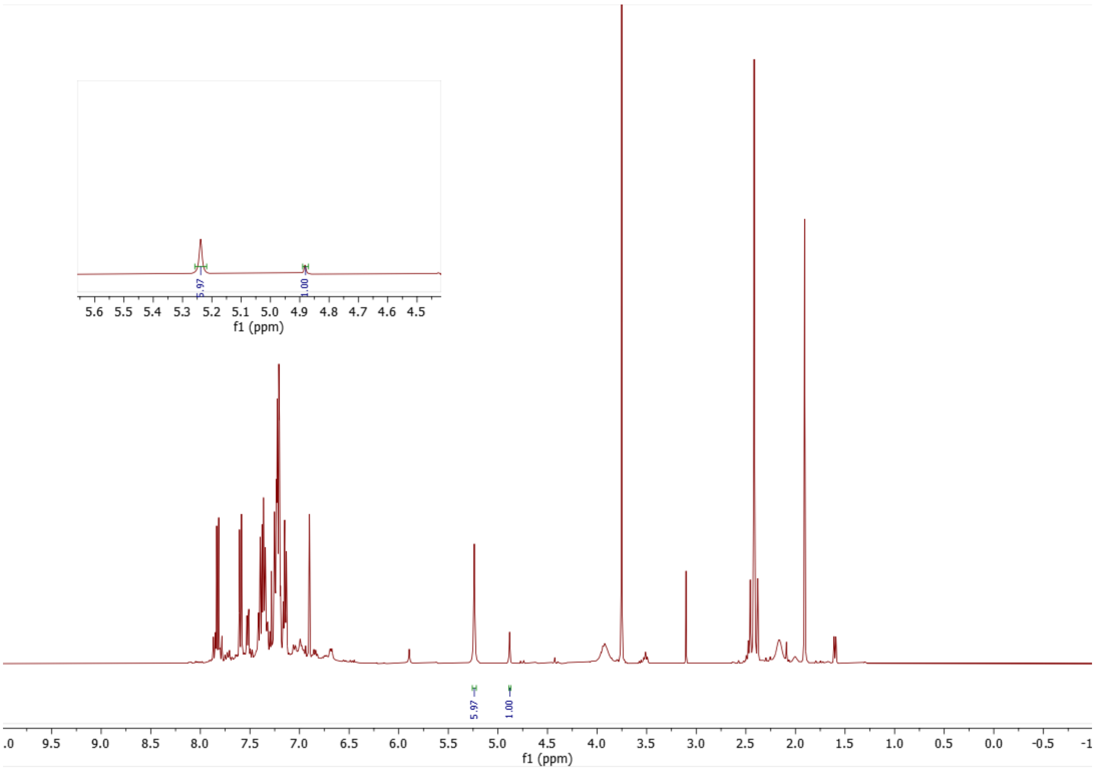

NOESY spectrum (assignment of major isomer):

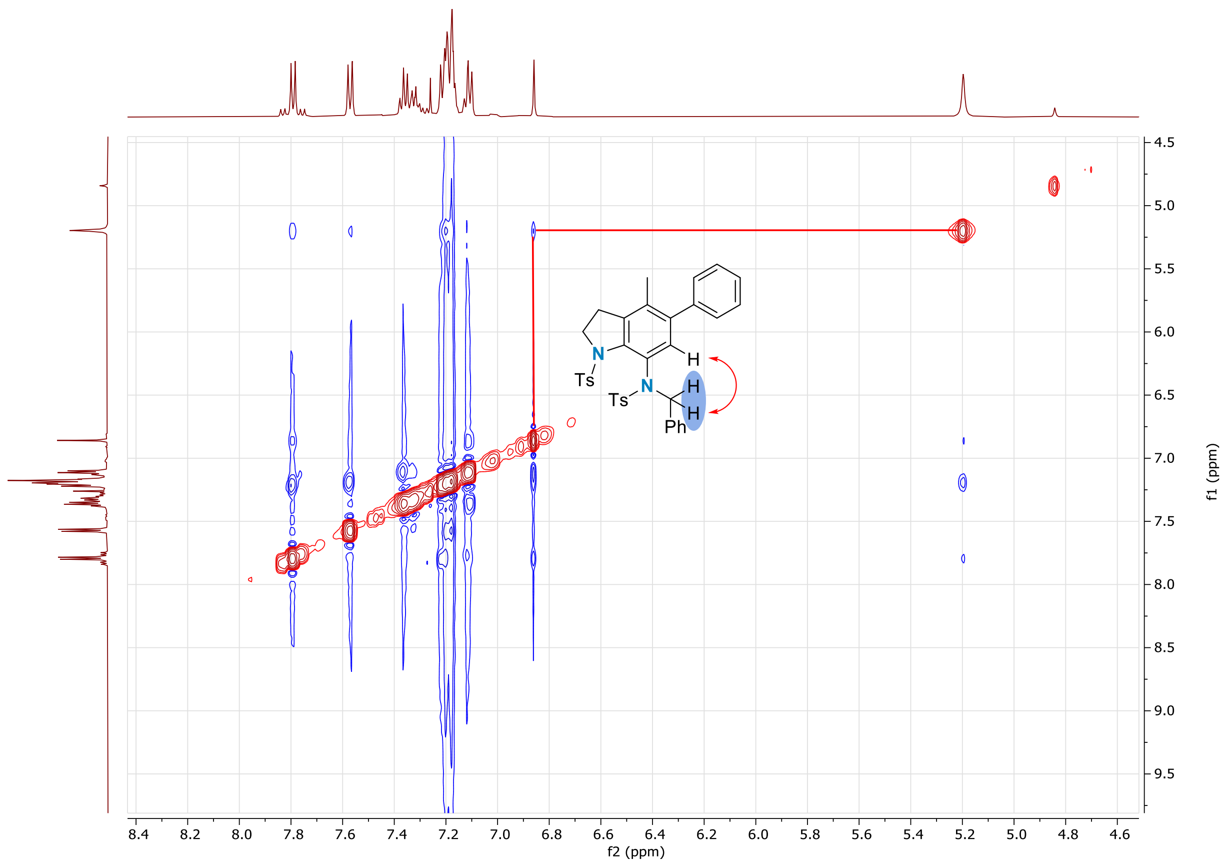

COSY spectrum (assignment of minor isomer):

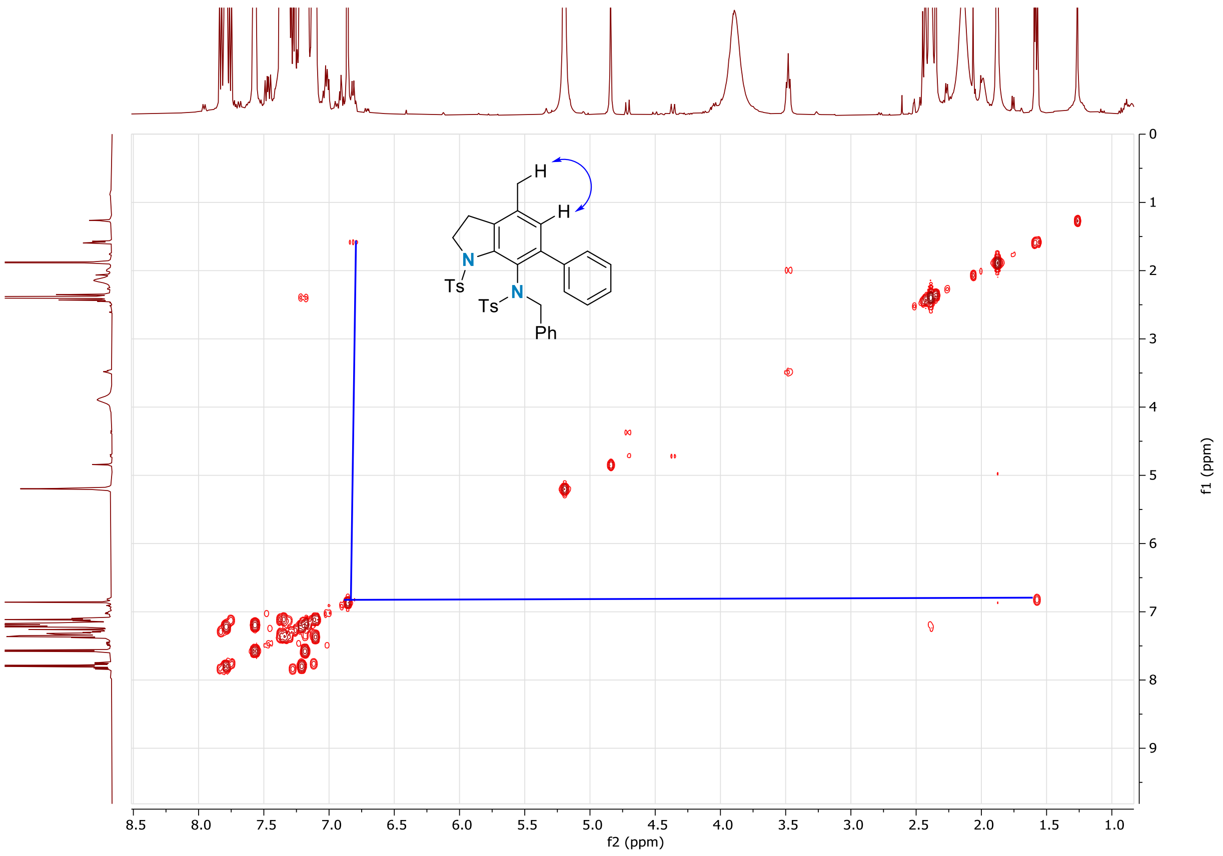

2. Synthesis of starting materials

S1 9-(2,2-Dibromovinyl)-9*H*-carbazole

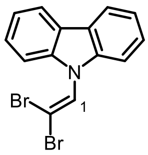

Carbazole (5.00 g, 29.9 mmol, 1.0 equiv.) was dissolved in formic acid (50 mL) and the reaction mixture was heated to 100 °C and stirred for 2 h then cooled to room temperature. The formic acid was removed *in vacuo* and the resulting solid was dried under high vacuum for 1 h to give *N*-formyl carbazole which was used in the next stage without further purification.

Triphenylphosphine (31.4 g, 120 mmol, 4.0 equiv.) was dissolved in anhydrous CH_2Cl_2 (180 mL) at -30 °C under an atmosphere of argon and CBr_4 (19.8 g, 59.8 mmol, 2.0 equiv.) was added slowly as a solution in anhydrous CH_2Cl_2 (30 mL). The reaction mixture was then cooled to -40 °C and stirred at this temperature for 30 minutes. *Note: at this stage an additional 200 mL of anhydrous CH_2Cl_2 was added due to insolubility of the PPh_3/CBr_4 adduct on this scale, this additional solvent is not normally required on smaller scales.* The crude *N*-formylcarbazole from the first stage was then added over 30 minutes as a solution in anhydrous CH_2Cl_2 (90 mL) and the reaction mixture was allowed to slowly warm to room temperature, then stirred at room temperature for 16 h. The reaction mixture was then concentrated *in vacuo* and purified by flash column chromatography (SiO_2, EtOAc in pentane, 5 to 20%) to give the title compound as a white solid (8.81 g, 25.2 mmol, 84%).

R_f (EtOAc in pentane, 10%) 0.90.

^{1}H NMR (400 MHz, $CDCl_3$) δ_H 8.09–8.07 (2H, m, ArH), 7.73 (1H, s, H1), 7.51–7.50 (2H, m, ArH), 7.39–7.37 (2H, m, ArH), 7.35–7.29 (2H, m, ArH).

^{13}C NMR (101 MHz, $CDCl_3$) δ_C 138.9, 129.8, 126.3, 123.9, 121.0, 120.6, 110.8, 95.7.

HRMS (ES$^+$) calc. for $C_{14}H_{10}NBr_2$ 351.9154 $[M+H]^+$, found 351.9152.

IR (thin film, ν_{max} / cm^{-1}) 1608, 1591, 1476, 1446, 1357, 1334, 1313, 1284, 1222, 1154, 1118.

M.P ($CHCl_3$) 104–106 °C.

Yndiamides

1a *N*-Benzyl-4-methyl-*N*-(((4-methyl-*N*-(pent-3-yn-1-yl)phenyl)sulfonamido)ethynyl)benzene sulfonamide

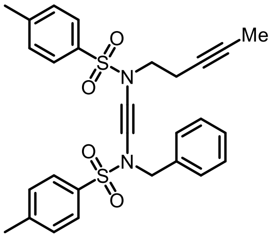

Prepared according to the literature procedure.[3]

1b *N*-Butyl-*N*-(((*N*-(4-(4-fluorophenyl)but-3-yn-1-yl)-4-methylphenyl)sulfonamido)ethynyl)-4-methylbenzenesulfonamide

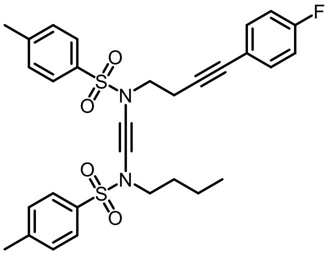

Prepared according to the literature procedure.[3]

1c *N*-Butyl-4-methyl-*N*-(((4-methyl-*N*-(4-phenylbut-3-yn-1-yl)phenyl)sulfonamido)ethynyl)benzenesulfonamide

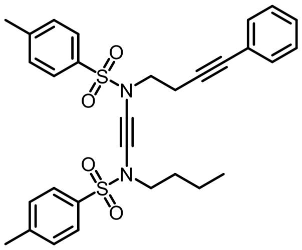

Prepared according to the literature procedure.[3]

1d *N*-((9*H*-Carbazol-9-yl)ethynyl)-4-methyl-*N*-(pent-3-yn-1-yl)benzenesulfonamide

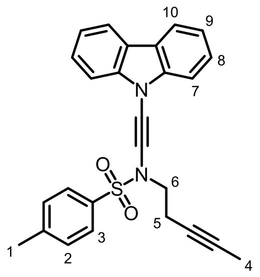

4-Methyl-*N*-(pent-3-yn-1-yl)benzenesulfonamide (0.237 g, 1.0 mmol, 1.0 equiv.), **S1** (0.385 g, 1.1 mmol, 1.1 equiv.), CuI (38.0 mg, 0.2 mmol, 20 mol%), 1,10-phenanthroline (72.0 mg, 0.4 mmol, 40 mol%) and Cs_2CO_3 (0.975 g, 3.0 mmol, 3.0 equiv.) were submitted to General Procedure **3** with anhydrous THF (3.0 mL) for 18 h with purification by flash column chromatography (SiO_2, EtOAc in pentane, 10%) followed by recrystallisation from pentane/Et_2O (1:1 v/v) to give the title compound (80.0 mg, 0.19 mmol, 19%) as a brown solid.

R_f (EtOAc in pentane, 10%) 0.34.

^{1}H NMR (500 MHz, $CDCl_3$) δ_H 8.00 (2H, d, *J* = 7.9 Hz, H3), 7.90 (2H, d, *J* = 8.4 Hz, ArH), 7.46 (4H, dd, *J* = 3.9, 0.8 Hz, ArH), 7.38 (2H, d, *J* = 7.9 Hz, H2), 7.33 (2H, dt, *J* = 8.1, 4.1 Hz, ArH), 3.70 (2H, t, *J* = 7.4 Hz, H6), 2.60 (2H, app. tp, *J* = 7.5, 2.7 Hz, H5), 2.50 (3H, s, H1), 1.71 (3H, t, *J* = 2.7 Hz, H4).

^{13}C NMR (126 MHz, $CDCl_3$) δ_C 144.9, 141.4, 134.9, 129.9, 128.1, 126.8, 123.9, 122.4, 120. 4, 111.6, 78.2, 74.8, 72.2, 65.0, 51.4, 21.9, 19.0, 3.6.

HRMS (ES^+) calc. for $C_{26}H_{23}O_2N_2S$ ($[M+H]^+$) 427.1475, found 427.1476.

IR (solid, ν_{max} / cm^{-1}) 2981, 2972, 2889, 1496, 1464, 1377, 1224, 1170, 1089.

MP (pentane/Et_2O, 1:1) 127–129 °C.

6a *N,N'*-(Ethyne-1,2-diyl)bis(*N*-butyl-4-methylbenzenesulfonamide)

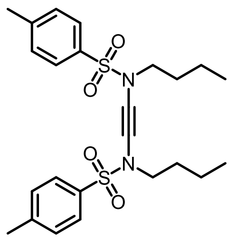

Prepared according to the literature procedure.[4]

6b *N,N'*-(Ethyne-1,2-diyl)bis(*N*-benzyl-4-methylbenzenesulfonamide)

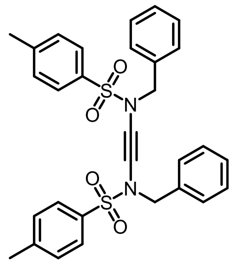

Prepared according to the literature procedure.[1]

6c *N*-((9*H*-carbazol-9-yl)ethynyl)-*N*-butyl-4-methylbenzenesulfonamide

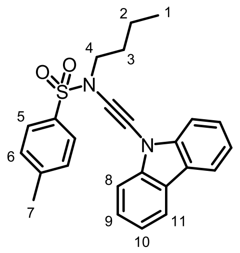

BuNHTs (2.27 g, 10.0 mmol, 1.00 equiv.), **S1** (3.86 g, 11.0 mmol, 1.1 equiv.), CuI (0.381 g, 2.00 mmol, 0.20 equiv.), Cs_2CO_3 (9.75 g, 30.0 mmol, 3.0 equiv) and 1,10-phenanthroline (0.720 g, 4.0 mmol, 0.40 equiv.) were submitted to General Procedure **3** with anhydrous THF (30 mL) for 18 h with purification by flash column chromatography (SiO_2, EtOAc in pentane, gradient, 0 to 10%) followed by recyrstallisation from Et_2O to give the title compound as a cream solid (2.67 g, 6.42 mmol, 64%).

R_f (EtOAc in pentane, 10%) 0.60.

^{1}H NMR (400 MHz, $CDCl_3$) δ_H 8.01 (2H, dd, *J* = 7.7, 2.0 Hz, ArH), 7.89 (2H, d, *J* = 8.5 Hz, H5), 7.49–7.44 (4H, m, ArH), 7.38 (2H, d, *J* = 8.5 Hz, H6), 7.36–7.29 (2H, m, ArH), 3.55 (2H, t, *J* = 7.2 Hz, H4), 2.50 (3H, s, H7), 1.79–1.71 (2H, m, H3), 1.48–1.39 (2H, m, H2), 0.95 (3H, t, *J* = 7.4 Hz, H1).

^{13}C NMR (101 MHz, $CDCl_3$) δ_C 144.7, 141.4, 134.9, 129.9, 128.0, 126.7, 123.8, 122.3, 120.4, 111.5, 72.5, 64.4, 52.0, 30.2, 21.8, 19.6, 13.8.

HRMS (ES^+) calc. for $C_{25}H_{25}O_2N_2S_1$ 417.1631 $[M+H]^+$, found 417.1631.

IR (thin film, ν_{max} / cm^{-1}) 2925, 1597, 1495, 1453, 1364, 1306, 1224, 1169, 1115, 1088.

MP (Et_2O) 115–117 °C.

6d *N*-((9*H*-Carbazol-9-yl)ethynyl)-*N*-benzyl-4-methylbenzenesulfonamide

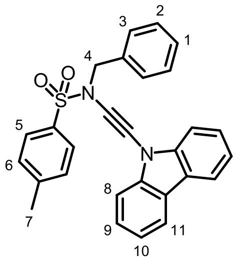

N-Benzyl-4-methylbenzenesulfonamide (0.339 g, 1.30 mmol, 1.0 equiv), **S1** (0.500 g, 1.43 mmol, 1.1 equiv.), CuI (49.5 mg, 0.26 mmol, 20 mol%), 1,10-phenanthroline (93.6 mg, 0.52 mmol, 40 mol%) and Cs_2CO_3 (1.27 g, 3.90 mmol, 3.0 equiv.) were submitted to General Procedure **3** with anhydrous THF (3.9 mL) for 20 h with purification by flash column chromatography (SiO_2, CH_2Cl_2 and EtOAc in pentane, 15:5:80) to give the title compound (0.366 g, 0.81 mmol, 63%) as a brown solid.

$\mathbf{R_f}$ (Pentane : CH_2Cl_2 : EtOAc, 80:15:5) 0.50.

^{1}H NMR (400 MHz, $CDCl_3$) δ_H 7.98 (2H, dt, *J* = 7.7, 1.0 Hz, H11), 7.82 (2H, d, *J* = 8.3 Hz, H3), 7.44–7.37 (4H, m, ArH), 7.34–7.31 (7H, m, ArH), 7.30–7.26 (2H, m, ArH), 4.75 (2H, s, H4), 2.49 (3H, s, H7).

^{13}C NMR (101 MHz, $CDCl_3$) δ_C 144.8, 141.3, 135.0, 134.9, 129.8, 129.0, 128.7, 128.5, 128.1, 126.7, 123.8, 122.3, 120.4, 111.5, 72.9, 65.4, 56.5, 21.8.

HRMS (ES^+) calc. for $C_{28}H_{22}O_2N_2SNa$ ($[M+Na]^+$) 473.1294, found 473.1295.

IR (thin film, ν_{max} / cm^{-1}) 1598, 1494, 1462, 1455, 1365, 1336, 1306, 1223, 1186, 1168, 1090.

MP ($CHCl_3$) 109–112 °C.

6e *N*-((9*H*-Carbazol-9-yl)ethynyl)-*N*-isopropyl-4-methylbenzenesulfonamide

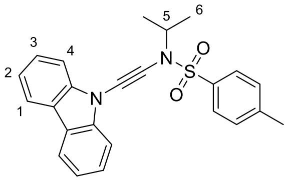

N-Isopropyl-4-methylbenzenesulfonamide (0.300 g, 1.41 mmol, 1.0 equiv), **S1** (0.543 g, 1.55 mmol, 1.1 equiv.), CuI (54.0 mg, 0.28 mmol, 20 mol%), 1,10-phenanthroline (0.101 g, 0.56 mmol, 40 mol%) and Cs_2CO_3 (1.37 g, 4.22 mmol, 3.0 equiv.) were submitted to General Procedure **3** with anhydrous THF (4.2 mL) for 20 h with purification by flash column chromatography (SiO_2, Et_2O in pentane, 20%) and recrystallisation from cyclohexane to give the title compound (0.212 g, 0.53 mmol, 38%) as a brown solid.

$\mathbf{R_f}$ (Et_2O in pentane, 20%) 0.29.

^{1}H NMR (400 MHz, $CDCl_3$) δ_H 8.01 (2H, dt, *J* = 7.7, 1.0 Hz, H1), 7.90 (2H, d, *J* = 8.3, ArH), 7.50–7.45 (4H, m, H3 + H4), 7.38–7.31 (4H, m, H2 + ArH), 4.41 (1H, hept, *J* = 6.5 Hz, H5), 2.49 (3H, s, CH_3), 1.23 (6H, d, *J* = 6.5 Hz, H6)

^{13}C NMR (101 MHz, $CDCl_3$) δ_C 144.6, 141.6, 136.1, 129.9, 127.9, 126.8, 123.9, 122.3, 120.4, 111.6, 69.3, 66.2, 52.5, 21.8, 21.0.

HRMS (ES^+) calc. for $C_{24}H_{23}O_2N_2S$ ($[M+H]^+$) 403.1475, found 403.1473.

IR (thin film, ν_{max} / cm^{-1}) 1462, 1436, 1366, 1171, 723, 677.

MP ($CHCl_3$) 153–154 °C.

6f *N*-((9*H*-Carbazol-9-yl)ethynyl)-*N*-cyclohexyl-4-methylbenzenesulfonamide

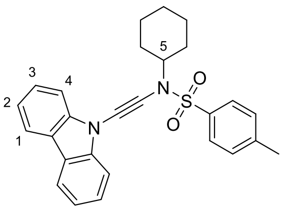

N-Cyclohexyl-4-methylbenzenesulfonamide (0.400 g, 1.58 mmol, 1.0 equiv), **S1** (0.610 g, 1.74 mmol, 1.1 equiv.), CuI (60.0 mg, 0.32 mmol, 20 mol%), 1,10-phenanthroline (0.114 g, 0.63 mmol, 40 mol%) and Cs_2CO_3 (1.54 g, 4.74 mmol, 3.0 equiv.) were submitted to General Procedure **3** with anhydrous THF (4.8 mL) for 20 h with purification by flash column chromatography (SiO_2, Et_2O in pentane, 20%) and recrystallisation from cyclohexane to give the title compound (0.156 g, 0.35 mmol, 22%) as a yellow solid.

R_f (Et_2O in pentane, 20%) 0.39.

^{1}H NMR (500 MHz, $CDCl_3$) δ_H 8.01 (2H, dt, *J* = 7.7, 1.0 Hz, H1), 7.89 (2H, dt, *J* = 8.2, 1.8 Hz, H9), 7.50–7.44 (4H, m, H3 and H4), 7.38–7.31 (4H, m, H10 + H2), 4.00 (1H, tt, *J* = 11.8, 3.9 Hz, H5), 2.49 (3H, s, CH_3), 1.85–1.74 (4H, m, CyH), 1.63–1.52 (3H, m, CyH), 1.36 (2H, qt, *J* = 13.3, 3.9 Hz, CyH), 1.01 (1H, qt, *J* = 13.3, 3.8 Hz, CyH).

^{13}C NMR (126 MHz, $CDCl_3$) δ_C 144.6, 141.6, 136.3, 129.9, 127.8, 126.8, 123.8, 122.3, 120.4, 111.6, 77.4, 70.3, 65.9, 59.5, 31.3, 25.5, 25.0, 21.8.

HRMS (ES^+) calc. for $C_{27}H_{26}O_2N_2SNa$ ($[M+Na]^+$) 465.1607, found 465.1608.

IR (thin film, ν_{max} / cm^{-1}) 1462, 1170, 751, 662.

MP ($CHCl_3$) 86–88 °C.

[2+2+2] Cyclotrimerization products

2aa *N*-Benzyl-*N*-(5,6-bis(hydroxymethyl)-4-methyl-1-tosylindolin-7-yl)-4-methylbenzenesulfonamide

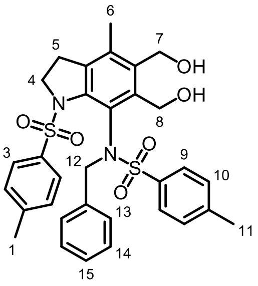

1a (52.0 mg, 0.10 mmol, 1.0 equiv.) was used in General Procedure **1** with 2-butyne-1,4-diol (25.8 mg, 0.30 mmol, 3.0 equiv.) for 16 h with purification by flash column chromatography (SiO_2, MeOH in CH_2Cl_2, 5 to 10%) to give the title compound (52.5 mg, 0.087 mmol, 87%) as a brown solid.

Scaled-up synthesis (1.0 mmol scale)

1a (0.520 g, 1.0 mmol, 1.0 equiv.) was used in General Procedure **1** with 2-butyne-1,4-diol (0.258 g, 3.0 mmol, 3.0 equiv.) for 16 h with purification by flash column chromatography (SiO_2, MeOH in CH_2Cl_2, 1 to 2%) to give the title compound (0.558 g, 92%) as a brown solid.

R_f (MeOH in CH_2Cl_2, 10%) 0.43.

^{1}H NMR (500 MHz, $CDCl_3$) δ_H 7.74 (2H, d, *J* = 8.2 Hz, ArH), 7.42–7.38 (4H, m, ArH), 7.35–7.31 (3H, m, ArH), 7.28 (2H, d, *J* = 8.2 Hz, ArH), 7.22 (2H, d, *J* = 8.2 Hz, ArH), 5.37 (1H, d, *J* = 14.6 Hz, H12), 5.25 (1H, d, *J* = 14.6 Hz, H12), 4.55 (2H, s, H7), 4.21 (1H, d, *J* = 13.5 Hz, H8), 3.85 (1H, d, *J* = 13.5 Hz, H8), 3.77 (1H, ddd, *J* = 12.8, 7.2, 2.4 Hz, H5), 3.69 (1H, s, OH), 3.12 (1H, ddd, *J* = 12.8, 10.6, 7.8 Hz, H5), 2.70 (1H, s, OH), 2.44 (3H, s, CH_3), 2.43 (3H, s, CH_3), 2.17 (3H, s, H6), 2.16–2.06 (2H, m, H4).

^{13}C NMR (126 MHz, $CDCl_3$) δ_C 144.5, 144.1, 143.5, 140.7, 140.53, 140.46, 138.5, 136.3, 136.2, 135.2, 131.2, 129.7, 129.4, 128.8, 128.5, 128.3, 128.2, 127.8, 59.6, 59.5, 56.1, 53.4, 29.3, 21.78, 21.75, 16.1.

HRMS (ES^+) calc. for $C_{32}H_{34}O_6N_2S_2Na$ ($[M+Na]^+$) 629.1750, found 629.1750.

IR (thin film, ν_{max} / cm^{-1}) 3487, 2924, 1598, 1452, 1355, 1328, 1155, 1090, 1039, 1028, 1001, 814, 756, 726, 704, 666.

MP ($CHCl_3$) 135–137 °C.

2ab *N*-Benzyl-*N*-(5-(4-(dimethylamino)phenyl)-4-methyl-1-tosylindolin-7-yl)-4-methylbenzene sulfonamide *(isolated as >20:1 r.r., crude >20:1 r.r.).*

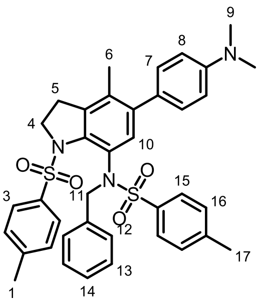

1a (52.0 mg, 0.10 mmol, 1.0 equiv.) was used in General Procedure **1** with 4-ethynyl-*N*,*N*-dimethylaniline (43.6 mg, 0.30 mmol, 3.0 equiv.) for 16 h with purification by flash column chromatography (SiO_2, EtOAc in pentane, 10 to 30%) to give the title compound (53.2 mg, 0.080 mmol, 80%) as a brown solid.

R_f (EtOAc in pentane, 30%) 0.22.

^{1}H NMR (400 MHz, $CDCl_3$) δ_H 7.79 (2H, d, *J* = 8.4 Hz, H15), 7.57 (2H, d, *J* = 8.3 Hz, H3), 7.23–7.14 (9H, m, ArH), 7.02 (2H, d, *J* = 8.8 Hz, H7), 6.88 (1H, s, H10), 6.72 (2H, d, *J* = 8.8 Hz, H8), 5.20 (2H, s, H11), 3.88 (2H, br s, H4), 3.00 (6H, s, H9), 2.39–2.38 (6H, m, H1 + H17), 2.12 (2H, br s, H5), 1.91 (3H, s, H6).

^{13}C NMR (101 MHz, $CDCl_3$) δ_C 149.7, 144.0, 143.1, 140.7, 139.2, 139.0, 137.3, 136.5, 135.1, 130.87, 130.85, 130.2, 130.1, 129.6, 129.5, 129.3, 128.4, 128.3, 128.2, 128.1, 127.4, 112.0, 53.3, 53.0, 40.7, 28.9, 21.72, 21.65, 17.1.

HRMS (ES^+) calc. for $C_{38}H_{40}O_4N_3S_2$ ($[M+H]^+$) 666.2455, found 666.2452.

IR (thin film, ν_{max} / cm^{-1}) 1611, 1525, 1477, 1445, 1352, 1165, 1090, 913, 816, 750, 686.

MP ($CHCl_3$) 115–118 °C.

2ac *N*-Benzyl-4-methyl-*N*-(4-methyl-5-(thiophen-2-yl)-1-tosylindolin-7-yl)benzenesulfonamide and *N*-benzyl-4-methyl-*N*-(4-methyl-6-(thiophen-2-yl)-1-tosylindolin-7-yl)benzenesulfonamide *(isolated as. 19:1 r.r., crude 10:1 r.r.)*

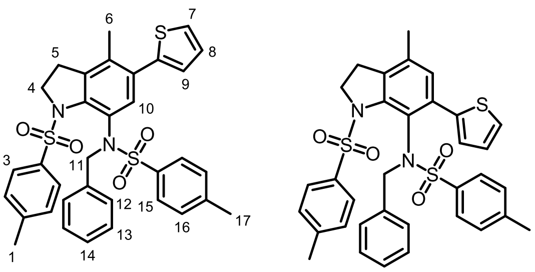

1a (52.0 mg, 0.10 mmol, 1.0 equiv.) was used in General Procedure **1** with 2-ethynylthiophene (32.4 mg, 0.30 mmol, 3.0 equiv.) for 16 h with purification by flash column chromatography (SiO_2, EtOAc in pentane, 10 to

30%) to give the title compounds as an inseparable mixture (42.0 mg, 0.067 mmol, 67%) as a pale brown amorphous solid. *NMR data is given for the major isomer in the isolated mixture.*

R$_f$ (EtOAc in pentane, 30%) 0.40.

^{1}H NMR (400 MHz, CDCl$_3$) δ_H 7.81 (2H, d, *J* = 8.3 Hz, H15), 7.56 (2H, d, *J* = 8.4 Hz, H3), 7.31 (1H, dd, *J* = 5.1, 1.2 Hz, H9), 7.24 (2H, d, *J* = 8.0 Hz, H16), 7.20–7.16 (7H, m, H2 + H12 + H13 + H14), 7.05–7.03 (2H, m, H8 + H10), 6.86 (1H, dd, *J* = 3.5, 1.1 Hz, H7), 5.20 (2H, s, H11), 3.88 (2H, br s, H4), 2.41 (3H, s, CH$_3$), 2.39 (3H, s, CH$_3$), 2.19–2.11 (2H, m, H5), 2.03 (3H, s, H6).

^{13}C NMR (126 MHz, CDCl$_3$) δ_C 144.2, 143.3, 141.6, 139.6, 138.8, 137.6, 137.0, 135.0, 132.8, 131.4, 131.3, 130.2, 129.6, 129.5, 129.4, 128.4, 128.3, 128.0, 127.6, 127.2, 127.0, 125.6, 53.1, 52.9, 28.9, 21.74, 21.68, 17.4.

HRMS (ES$^+$) calc. for $C_{34}H_{33}O_4N_2S_3$ ([M+H]$^+$) 629.1957, found 629.1957.

IR (thin film, ν_{max} / cm^{-1}) 1356, 1169, 1159, 1091, 909, 730, 700.

2ad ***N*-Benzyl-4-methyl-*N*-(4-methyl-5-(thiophen-3-yl)-1-tosylindolin-7-yl)benzenesulfonamide and *N*-benzyl-4-methyl-*N*-(4-methyl-6-(thiophen-3-yl)-1-tosylindolin-7-yl)benzenesulfonamide** *(isolated as 9:1 r.r., crude 9:1 r.r.)*

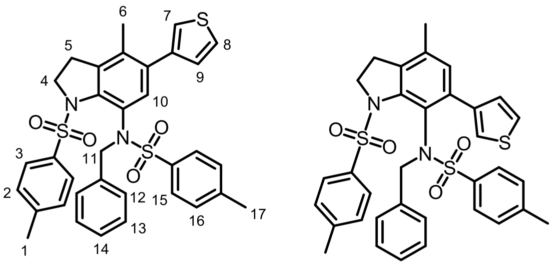

1a (52.0 mg, 0.10 mmol, 1.0 equiv.) was used in General Procedure **1** with 3-ethynylthiophene (32.4 mg, 0.30 mmol, 3.0 equiv.) for 16 h with purification by flash column chromatography (SiO$_2$, EtOAc in pentane, 10 to 30%) to give the title compounds as an inseparable mixture (41.0 mg, 0.065 mmol, 65%) as a pale brown amorphous solid. *NMR data is given for the major isomer in the isolated mixture.*

R$_f$ (EtOAc in pentane, 20%) 0.22.

^{1}H NMR (400 MHz, CDCl$_3$) δ_H 7.80 (2H, d, *J* = 8.3 Hz, H15), 7.56 (2H, d, *J* = 8.3 Hz, H3), 7.31 (1H, dd, *J* = 4.9, 3.0 Hz, H9), 7.25–7.17 (9H, m, ArH), 6.99 (1H, dd, *J* = 3.0, 1.3 Hz, H8), 6.92 (1H, dd, *J* = 4.9, 1.3 Hz, H7), 6.89 (1H, s, H10), 5.18 (2H, s, H11), 3.90–3.81 (2H, m, H4), 2.41 (3H, s, CH$_3$), 2.39 (3H, s, CH$_3$), 2.15–2.11 (2H, m, H5), 1.95 (3H, s, H6).

^{13}C NMR (101 MHz, CDCl$_3$) δ_C 144.1, 143.3, 140.6, 139.4, 138.7, 137.4, 137.2, 135.07, 135.05, 131.14, 131.11, 130.0, 129.7, 129.5, 129.3, 128.9, 128.5, 128.3, 128.0, 127.6, 125.1, 123.2, 53.3, 52.9, 28.9, 21.7, 21.7, 17.2.

HRMS (ES+) calc. for $C_{34}H_{33}O_4N_2S_3$ ([M+H]+) 629.1597, found 629.1595.

IR (thin film, v_{max} / cm^{-1}) 2977, 1598, 1354, 1167, 1159, 1091, 815, 687.

2ae *N*-Benzyl-*N*-(5-(4-methoxyphenyl)-4-methyl-1-tosylindolin-7-yl)-4-methylbenzenesulfonamide and ***N*-benzyl-*N*-(6-(4-methoxyphenyl)-4-methyl-1-tosylindolin-7-yl)-4-methylbenzenesulfonamide** *(isolated as 7:1 r.r., crude 8:1 r.r.)*

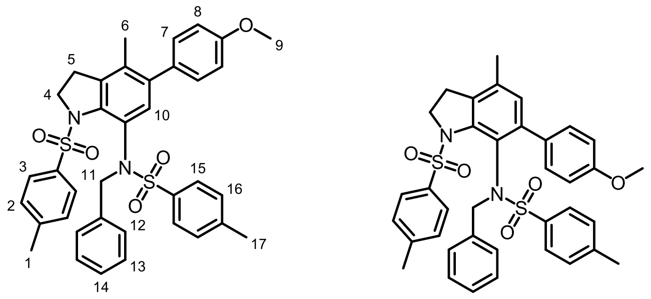

1a (52.0 mg, 0.10 mmol, 1.0 equiv.) was used in General Procedure **1** with 4-ethynylanisole (39.6 mg, 0.30 mmol, 3.0 equiv.) for 16 h with purification by flash column chromatography (SiO_2, EtOAc in pentane, 10 to 40%) to give the title compounds as an inseparable mixture (59.8 mg, 0.092 mmol, 92%) as a yellow foam. *NMR data is given for the major isomer in the isolated mixture.*

R_f (EtOAc in pentane, 30%) 0.37.

^{1}H NMR (500 MHz, $CDCl_3$) δ_H 7.79 (2H, d, *J* = 8.4 Hz, H15), 7.57 (2H, d, *J* = 8.4 Hz, H3), 7.23–7.16 (9H, m, ArH), 7.03 (2H, d, *J* = 8.7 Hz, H7), 6.89 (2H, d, *J* = 8.7 Hz, H8), 6.83 (1H, s, H10), 5.18 (2H, s, H11), 3.89–3.84 (5H, m, H4 + H9), 2.39 (6H, s, H1 + H17), 2.17–2.07 (2H, m, H5), 1.88 (3H, s, H6).

^{13}C NMR (126 MHz, $CDCl_3$) δ_C 158.9, 144.1, 143.2, 140.2, 139.3, 138.8, 137.2, 137.1, 135.1, 132.7, 131.1, 130.9, 130.5, 130.0, 129.7, 129.5, 129.3, 128.5, 128.3, 128.1, 127.5, 113.6, 55.5, 53.2, 53.0, 28.9, 21.8, 21.7, 17.0.

HRMS (ES+) calc. for $C_{37}H_{37}O_5N_2S_2$ ([M+H]+) 653.2138, found 653.2134.

IR (thin film, v_{max} / cm^{-1}) 2979, 2160, 2029, 1977, 1673, 1606, 1454, 1352, 1245, 1158, 1089, 1028.

2af ***N*-Benzyl-4-methyl-*N*-(4-methyl-5-phenyl-1-tosylindolin-7-yl)benzenesulfonamide** & ***N*-benzyl-4-methyl-*N*-(4-methyl-6-phenyl-1-tosylindolin-7-yl)benzenesulfonamide** *(isolated as 6:1 r.r., crude 6:1 r.r.)*

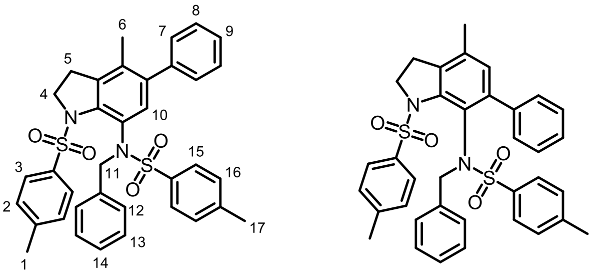

1a (52.0 mg, 0.10 mmol, 1.0 equiv.) was used in General Procedure **1** with phenylacetylene (30.6 mg, 0.30 mmol, 3.0 equiv.) for 16 h with purification by flash column chromatography (SiO_2, EtOAc in pentane, 10 to 20%) to give the title compounds as an inseparable mixture (48.0 mg, 0.077 mmol, 77%) as a yellow foam. *NMR data is given for the major isomer in the isolated mixture.*

R$_f$ (EtOAc in pentane, 30%) 0.45.

^{1}H NMR (500 MHz, $CDCl_3$) δ_H 7.79 (2H, d, *J* = 8.3 Hz, H15), 7.57 (2H, d, *J* = 8.3 Hz, H3), 7.38–7.30 (3H, m, ArH), 7.23–7.16 (8H, m, ArH), 7.12–7.09 (3H, m, ArH), 6.86 (1H, s, H10), 5.20 (2H, s, H11), 3.97–3.81 (2H, m, H4), 2.40–2.38 (6H, m, H1 + H17), 2.18–2.10 (2H, m, H5), 1.88 (3H, s, H6).

^{13}C NMR (126 MHz, $CDCl_3$) δ_C 144.1, 143.2, 140.5, 140.3, 139.3, 138.7, 137.3, 137.1, 135.1, 131.0, 130.9, 130.0, 129.6, 129.5, 129.4, 129.3, 128.5, 128.3, 128.2, 128.0, 127.5, 127.2, 53.2, 52.9, 28.9, 21.7, 21.6, 17.0.

HRMS (ES$^+$) calc. for $C_{36}H_{35}O_4N_2S_2$ ([M+H]$^+$) 623.2033, found 623.2031.

IR (thin film, v_{max} / cm^{-1}) 2980, 1597, 1474, 1354, 1158, 1090.

2ag ***N*-Benzyl-4-methyl-*N*-(4-methyl-5-(*p*-tolyl)-1-tosylindolin-7-yl)benzenesulfonamide** & ***N*-benzyl-4-methyl-*N*-(4-methyl-6-(*p*-tolyl)-1-tosylindolin-7-yl)benzenesulfonamide** *(isolated as 7:1 r.r., crude 6:1 r.r.)*

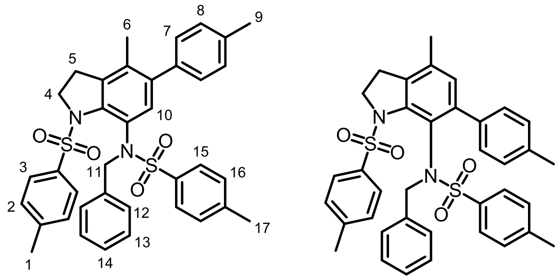

1a (52.0 mg, 0.10 mmol, 1.0 equiv.) was used in General Procedure **1** with 4-methylphenylacetylene (34.8 mg, 0.30 mmol, 3.0 equiv.) for 16 h with purification by flash column chromatography (SiO_2, EtOAc in pentane, 10 to 20%) to give the title compounds as an inseparable mixture (36.0 mg, 0.057 mmol, 57%) as a cream amorphous solid. *NMR data is given for the major isomer in the isolated mixture.*

R$_f$ (Et_2O in pentane, 20%) 0.25.

^{1}H NMR (400 MHz, $CDCl_3$) δ_H 7.78 (2H, d, J = 8.3 Hz, H15), 7.58 (2H, d, J = 8.3 Hz, H3), 7.23–7.14 (11H, m, ArH), 7.00 (2H, d, J = 8.1 Hz, H8), 6.84 (1H, s, H10), 5.19 (2H, s, H11), 3.88 (2H, br s, H4), 2.39 (9H, s, H1 + H9 + H17), 2.13 (2H, br. s, H5), 1.88 (3H, s, H6).

^{13}C NMR (101 MHz, $CDCl_3$) δ_C 144.1, 143.2, 140.5, 139.3, 138.8, 137.4, 137.2, 137.1, 136.9, 135.1, 131.1, 130.9, 130.1, 129.6, 129.5, 129.3 (2 C), 128.9, 128.5, 128.3, 128.1, 127.5, 53.3, 53.0, 28.9, 21.8, 21.7, 21.3, 17.0.

HRMS (ES$^+$) calc. for $C_{37}H_{37}O_4N_2S_2$ ($[M+H]^+$) 637.2189, found 637.2189.

IR (thin film, v_{max} / cm^{-1}) 2981, 1721, 1598, 1356, 1278, 1162, 1091.

2ah ***N*-Benzyl-4-methyl-*N*-(4-methyl-5-(naphthalen-2-yl)-1-tosylindolin-7-yl)benzenesulfonamide** & ***N*-benzyl-4-methyl-*N*-(4-methyl-6-(naphthalen-2-yl)-1-tosylindolin-7-yl)benzenesulfonamide** *(isolated as 5:1 r.r., crude 5:1 r.r.)*

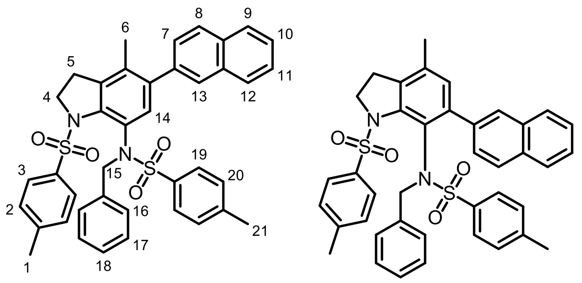

1a (52.0 mg, 0.10 mmol, 1.0 equiv.) was used in General Procedure **1** with 2-ethynylnapthalene (45.6 mg, 0.30 mmol, 3.0 equiv.) for 16 h with purification by flash column chromatography (SiO_2, EtOAc in pentane, 10 to 20%) to give the title compounds as an inseparable mixture (52.0 mg, 0.077 mmol, 77%) as a cream amorphous solid. *NMR data is given for the major isomer in the isolated mixture.*

R_f (EtOAc in pentane, 30%) 0.37.

^{1}H NMR (400 MHz, $CDCl_3$) δ_H 7.85–7.78 (6H, m, ArH), 7.63 (2H, d, J = 8.4 Hz, ArH), 7.55–7.50 (3H, m, ArH), 7.27–7.19 (9H, m, ArH), 6.87 (1H, s, H14), 5.21 (2H, s, H15), 3.96–3.87 (2H, m, H4), 2.41 (3H, s, CH_3), 2.37 (3H, s, CH_3), 2.24–2.16 (2H, m, H5), 1.94 (3H, s, H6).

^{13}C NMR (101 MHz, $CDCl_3$) δ_C 144.2, 143.2, 140.4, 139.4, 138.7, 137.8, 137.1, 135.2, 133.3, 132.4, 131.6, 131.2, 130.0, 129.8, 129.6, 129.5, 129.4, 128.4, 128.3, 128.2, 128.1, 128.0, 127.8, 127.64, 127.61, 127.58, 126.5, 126.2, 53.3, 53.9, 29.0, 21.8, 21.6, 17.1.

HRMS (ES$^+$) calc. for $C_{40}H_{37}O_4N_2S_2$ ($[M+H]^+$) 673.2189, found 673.2188.

IR (thin film, v_{max} / cm^{-1}) 2979, 1355, 1169, 1051, 1033, 1018, 815, 670.

2ai *N*-Benzyl-*N*-(5-(4-fluorophenyl)-4-methyl-1-tosylindolin-7-yl)-4-methylbenzenesulfonamide *(isolated as 5:1 r.r., crude 5:1 r.r.)*

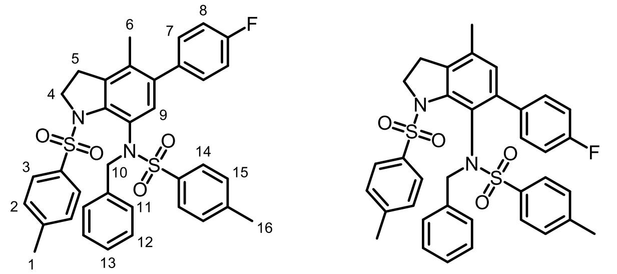

1a (52.0 mg, 0.10 mmol, 1.0 equiv.) was used in General Procedure **1** with 1-ethynyl-4-fluorobenzene (36.0 mg, 0.30 mmol, 3.0 equiv.) for 16 h with purification by flash column chromatography (SiO_2, EtOAc in pentane, 10 to 20%) to give the title compound (35.0 mg, 0.055 mmol, 55%) as an orange foam and a mixture of the title compound and the minor isomer (22.5 mg, 0.035 mmol, 35%) as a brown oil. *NMR data is given for the major isomer in the isolated mixture.*

R$_f$ (EtOAc in pentane, 20%) 0.22.

^{1}H NMR (400 MHz, $CDCl_3$) δ_H 7.79 (2H, d, *J* = 8.4 Hz, H15), 7.57 (2H, d, *J* = 8.4 Hz, H3), 7.24–7.16 (9H, m, ArH), 7.05–7.02 (4H, m, H7 + H8), 6.77 (1H, s, H9), 5.17 (2H, s, H10), 3.88 (2H, br s, H4), 2.40 (6H, s, H1 + H16), 2.15 (2H, br s, H5), 1.86 (3H, s, H6).

^{13}C NMR (101 MHz, $CDCl_3$) δ_C 162.2 (d, $^1J_{C\text{-}F}$ = 246.5 Hz), 144.2, 143.3, 139.43, 139.35, 138.6, 137.7, 137.1, 136.2 (d, $^4J_{C\text{-}F}$ = 3.5 Hz), 135.1, 131.4, 130.94 (d, $^3J_{C\text{-}F}$ = 8.0 Hz), 130.93, 130.0, 129.8, 129.5, 129.3, 128.5, 128.3, 128.0, 127.6, 115.1 (d, $^2J_{C\text{-}F}$ = 21.3 Hz), 53.2, 52.9, 28.9, 21.74, 21.66, 16.9.

^{19}F NMR (377 MHz, $CDCl_3$) δ_F -115.36.

HRMS (ES^+) calc. for $C_{36}H_{34}O_4N_2FS_2$ ($[M+H]^+$) 641.1939, found 641.1934.

IR (thin film, ν_{max} / cm^{-1}) 2977, 2932, 1598, 1506, 1353, 1222, 1158, 1090, 1018.

2aj *N*-Benzyl-*N*-(5-(3,4-dichlorophenyl)-4-methyl-1-tosylindolin-7-yl)-4-methylbenzenesulfonamide & *N*-benzyl-*N*-(6-(3,4-dichlorophenyl)-4-methyl-1-tosylindolin-7-yl)-4-methylbenzenesulfonamide *(isolated as 4:1 r.r., crude 4:1 r.r.)*

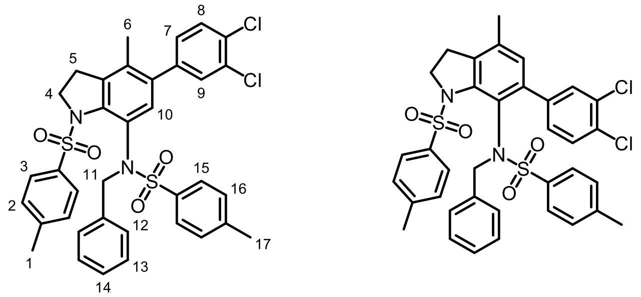

1a (52.0 mg, 0.10 mmol, 1.0 equiv.) was used in General Procedure **1** with 3,4-dichlorophenylacetylene (51.3

mg, 0.30 mmol, 3.0 equiv.) for 16 h with purification by flash column chromatography (SiO_2, EtOAc in pentane, 10 to 20%) to give the title compounds as an inseparable mixture (44.9 mg, 0.065 mmol, 65%) as a pale brown foam. *NMR data is given for the major isomer in the isolated mixture.*

R$_f$ (EtOAc in pentane, 30%) 0.37.

^{1}H NMR (400 MHz, $CDCl_3$) δ_H 7.78 (2H, d, *J* = 8.3 Hz, H15), 7.60 (2H, d, *J* = 8.3 Hz, H3), 7.41 (1H, d, *J* = 8.3 Hz, H8), 7.25–7.18 (8H, m, ArH), 7.10 (1H, d, *J* = 2.1 Hz, H9), 7.06 (1H, d, *J* = 3.8 Hz, ArH), 6.93 (1H, dd, *J* = 8.3, 2.1 Hz, H7), 6.61 (1H, s, H10), 5.16 (2H, s, H11), 3.90–3.84 (2H, m, H4), 2.42–2.40 (6H, m, H1 + H17), 2.20–2.13 (2H, m, H5), 1.88 (3H, s, H6).

^{13}C NMR (101 MHz, $CDCl_3$) δ_C 144.3, 143.5, 140.2, 139.6, 138.5, 137.7, 137.0, 135.2, 132.4, 131.50, 131.48, 131.3, 130.9, 130.8, 130.1, 130.0, 129.9, 129.61, 129.59, 129.4, 128.43, 128.39, 128.0, 127.8, 53.3, 52.9, 29.0, 21.8, 21.7, 16.9.

HRMS (ES$^+$) calc. for $C_{36}H_{33}O_4N_2Cl_2S_2$ ($[M+H]^+$) 691.1253, found 691.1252.

IR (thin film, v_{max} / cm^{-1}) 1465, 1356, 1159, 1091, 909, 815, 732.

2ak *N*-Benzyl-*N*-(5-(3-fluorophenyl)-4-methyl-1-tosylindolin-7-yl)-4-methylbenzenesulfonamide & *N*-benzyl-*N*-(6-(3-fluorophenyl)-4-methyl-1-tosylindolin-7-yl)-4-methylbenzenesulfonamide *(isolated as 8:1 r.r., crude 3:1 r.r.)*

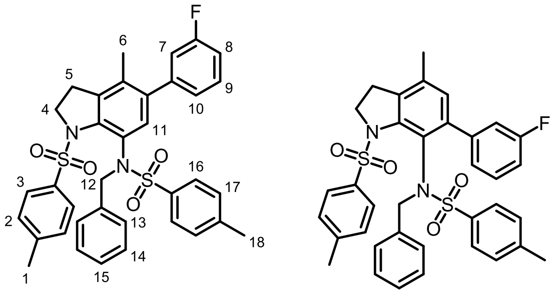

1a (52.0 mg, 0.10 mmol, 1.0 equiv.) was used in General Procedure **1** with 1-ethynyl-3-fluorobenzene (36.0 mg, 0.30 mmol, 3.0 equiv.) for 16 h with purification by flash column chromatography (SiO_2, EtOAc in pentane, 5 to 20%) to give the title compounds as an inseparable mixture (11.7 mg, 0.018 mmol, 18%) as a yellow foam. *NMR data is given for the major isomer in the isolated mixture.*

R$_f$ (EtOAc in pentane, 20%) 0.19.

^{1}H NMR (500 MHz, $CDCl_3$) δ_H 7.78 (2H, d, *J* = 8.3 Hz, H16), 7.58 (2H, d, *J* = 8.4 Hz, H3), 7.31 (1H, td, *J* = 8.0, 6.0 Hz, H9), 7.25–7.17 (9H, m, ArH), 7.03–6.99 (1H, m, H8), 6.87 (1H, dt, *J* = 7.8, 1.2 Hz, H10), 6.78–6.74 (2H, m, H7 + H11), 5.17 (2H, s, H12), 3.88 (2H, br s, H4), 2.40 (6H, m, H1 + H18), 2.17 (2H, br s, H5), 1.88 (3H, s, H6).

^{13}C NMR (126 MHz, $CDCl_3$) δ_C 162.6 (d, $^1J_{C\text{-}F}$ = 246.4 Hz), 144.2, 143.5, 142.5, 139.5, 139.1, 138.5, 138.0, 137.0, 135.1, 131.2, 130.9, 130.0, 129.8, 129.63 (d, $^3J_{C\text{-}F}$ = 8.6 Hz), 129.56, 129.4, 128.5, 128.4, 128.1, 127.7,

125.2 (d, ${}^{4}J_{C-F}$ =2.9 Hz), 116.4 (d, ${}^{2}J_{C-F}$ = 21.3 Hz), 114.1 (d, ${}^{2}J_{C-F}$ = 20.7 Hz), 53.2, 52.9, 28.9, 21.8, 21.7, 17.0. *One ${}^{3}J_{C-F}$ doublet observed as singlet due to overlap.*

^{19}F NMR (471 MHz, $CDCl_3$) δ_F -113.45.

HRMS (ES$^+$) calc. for $C_{36}H_{34}O_4N_2FS_2$ ([M+H]$^+$) 641.1939, found 641.1937.

IR (thin film, v_{max} / cm^{-1}) 2981, 2919, 1714, 1597, 1495, 1454, 1353, 1161, 1091, 1032.

2al ***N*-Benzyl-4-methyl-*N*-(4-methyl-5-(4-(4,4,5,5-tetramethyl-1,3,2-dioxaborolan-2-yl)phenyl)-1-tosylindolin-7-yl)benzenesulfonamide & *N*-benzyl-4-methyl-*N*-(4-methyl-6-(4-(4,4,5,5-tetramethyl-1,3,2-dioxaborolan-2-yl)phenyl)-1-tosylindolin-7-yl)benzenesulfonamide** *(isolated as 8 : 1 r.r., crude 3:1 r.r.)*

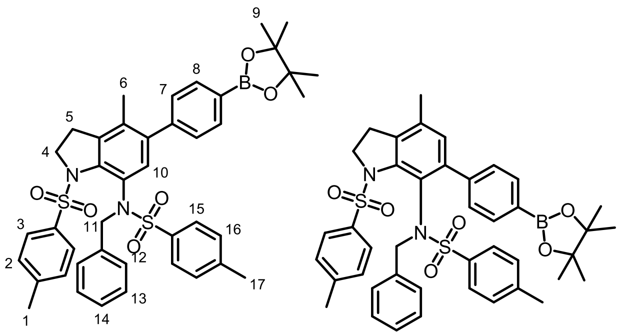

1a (52.0 mg, 0.10 mmol, 1.0 equiv.) was used in General Procedure **1** with 4-ethynylphenylboronic acid pinacol ester (68.4 mg, 0.30 mmol, 3.0 equiv.) for 16 h with purification by flash column chromatography (SiO_2, EtOAc in pentane, 10 to 20%) to give the title compounds as an inseparable mixture (62.9 mg, 0.084 mmol, 84%) as a yellow foam. *NMR data is given for the major isomer in the isolated mixture.*

R$_f$ (EtOAc in pentane, 30%) 0.33.

^{1}H NMR (400 MHz, $CDCl_3$) δ_H 7.80 (2H, d, *J* = 8.1 Hz, ArH), 7.76 (2H, d, *J* = 8.3 Hz, ArH), 7.61 (2H, d, *J* = 8.3 Hz, ArH), 7.22–7.19 (4H, m, ArH), 7.17–7.15 (5H, m, H12 + H13 + H14), 7.10 (2H, d, *J* = 8.1 Hz), 6.76 (1H, s, H10), 5.19 (2H, s, H11), 3.92–3.85 (2H, m, H4), 2.40 (3H, s, CH_3), 2.39 (3H, s, CH_3), 2.24–2.12 (2H, m, H5), 1.88 (3H, s, H6), 1.38 (12H, s, H9).

^{13}C NMR (101 MHz, $CDCl_3$) δ_C 144.1, 143.2, 143.1, 140.3, 139.4, 138.8, 137.8, 137.0, 135.2, 134.6, 131.1, 131.0, 130.0, 129.7, 129.5, 129.4, 128.8, 128.32, 128.30, 128.0, 127.6, 84.0, 53.3, 52.9, 28.9, 25.0, 21.74, 21.65, 17.0 *One aromatic carbon peak not found.*

HRMS (ES$^+$) calc. for $C_{42}H_{46}O_6N_2BS_2$ ([M+H]$^+$) 749.2891, found 749.2885.

IR (thin film, v_{max} / cm^{-1}) 2980, 1358, 1161, 1089.

2am Methyl 4-(7-((*N*-benzyl-4-methylphenyl)sulfonamido)-4-methyl-1-tosylindolin-5-yl)benzoate & methyl 4-(7-((*N*-benzyl-4-methylphenyl)sulfonamido)-4-methyl-1-tosylindolin-6-yl)benzoate

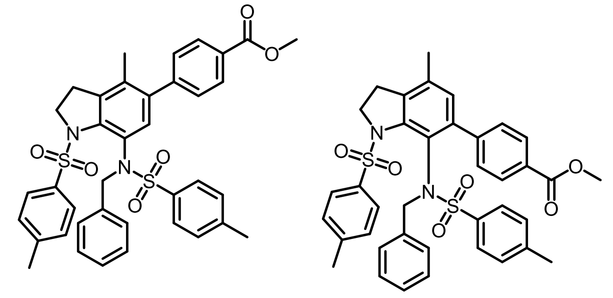

1a (52.0 mg, 0.10 mmol, 1.0 equiv.) was used in General Procedure **1** with methyl 4-ethynylbenzoate (48.0 mg, 0.30 mmol, 3.0 equiv.) for 16 h. *The yield of this compound was calculated by quantitative ^{1}H NMR on the crude reaction mixture as clean material could not be isolated.*

2an *N*-Benzyl-4-methyl-*N*-(4-methyl-1-tosyl-5-(4-(trifluoromethyl)phenyl)indolin-7-yl)benzenesulfonamide & *N*-benzyl-4-methyl-*N*-(4-methyl-1-tosyl-6-(4-(trifluoromethyl)phenyl)indolin-7-yl)benzenesulfonamide *(isolated as 5:1 r.r., crude 2:1 r.r.)*

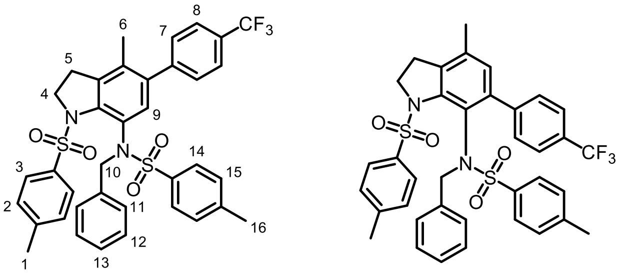

1a (52.0 mg, 0.10 mmol, 1.0 equiv.) was used in General Procedure **1** with 4-ethynyl-α,α,α-trifluorotoluene (51.0 mg, 0.30 mmol, 3.0 equiv.) for 16 h with purification by flash column chromatography (SiO_2, EtOAc in pentane, 10 to 20%) to give the title compounds as an inseparable mixture (49.2 mg, 0.071 mmol, 71%) as a yellow foam. *NMR data is given for the major isomer in the isolated mixture.*

R$_f$ (EtOAc in pentane, 20%) 0.26.

^{1}H NMR (500 MHz, $CDCl_3$) 7.79 (2H, d, J = 8.3 Hz, H14), 7.62–7.57 (4H, m, ArH), 7.24–7.17 (11H, m, ArH), 6.76 (1H, s, H9), 5.17 (2H, s, H10), 3.95–3.82 (2H, m, H4), 2.41–2.40 (6H, m, H1 + H16), 2.25–2.12 (2H, m, H5), 1.88 (3H, s, H6).

^{13}C NMR (126 MHz, $CDCl_3$) δ_C 144.3, 143.4, 139.6, 138.9, 138.5, 138.3, 137.0, 135.1, 134.3 (q, $^{2}J_{C\text{-}F}$ = 53.5 Hz), 131.4, 130.9, 130.1, 129.8, 129.7, 129.61, 129.58, 129.4, 128.5, 128.4, 128.0, 127.6, 125.2 (q, $^{3}J_{C\text{-}F}$ = 3.6 Hz), 124.3 (q, $^{1}J_{C\text{-}F}$ = 271.8 Hz), 53.2, 52.9, 28.9, 21.8, 21.7, 17.0.

^{19}F NMR (377 MHz, $CDCl_3$) δ_F -62.44.

HRMS (ES$^+$) calc. for $C_{37}H_{34}O_4N_2F_3S_2$ ([M+H]$^+$) 691.1907, found 691.1902.

IR (thin film, v_{max} / cm^{-1}) 2920, 2850, 1599, 1512, 1477, 1355, 1266, 1224, 1158, 1091.

2ao *N*-Benzyl-*N*-(5-(4-cyanophenyl)-4-methyl-1-tosylindolin-7-yl)-4-methylbenzenesulfonamide & ***N*-benzyl-*N*-(6-(4-cyanophenyl)-4-methyl-1-tosylindolin-7-yl)-4-methylbenzenesulfonamide** *(isolated as 2:1 r.r., crude 2:1 r.r.)*

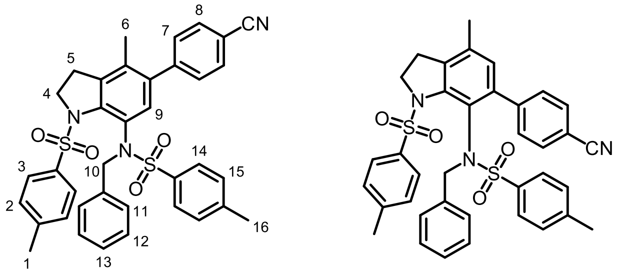

1a (52.0 mg, 0.10 mmol, 1.0 equiv.) was used in General Procedure **1** with 4-ethynylbenzonitrile (38.1 mg, 0.30 mmol, 3.0 equiv.) for 16 h with purification by flash column chromatography (SiO_2, EtOAc in pentane, 10 to 20%) to give the title compounds as an inseparable mixture (21.1 mg, 0.033 mmol, 33%) as an orange foam. *NMR data is given for the major isomer in the isolated mixture.*

R_f (EtOAc in pentane, 30%) 0.35.

^{1}H NMR (400 MHz, $CDCl_3$) δ_H 7.79 (2H, d, *J* = 8.4 Hz, H14), 7.66–7.62 (2H, m, ArH), 7.58 (2H, d, *J* = 8.3 Hz, H3), 7.25–7.14 (11H, m, ArH), 6.71 (1H, s, H9), 5.15 (2H, s, H10), 3.91–3.88 (2H, m, H4), 2.41 (6H, s, H1 + H16), 2.22–2.16 (2H, m, H5), 1.87 (3H, s, H6).

^{13}C NMR (126 MHz, $CDCl_3$) δ_C 145.0, 144.3, 143.5, 139.8, 138.7, 138.4, 138.3, 136.9, 135.0, 132.0, 131.4, 134.0, 130.1, 129.8, 129.6, 129.4, 128.5, 128.4, 128.09, 127.97, 127.7, 118.9, 111.1, 53.2, 52.9, 28.9, 21.8, 21.7, 16.9.

HRMS (ES$^+$) calc. for $C_{37}H_{34}O_4N_3S_2$ ($[M+H]^+$) 648.1985, found 648.1983.

IR (thin film, v_{max} / cm^{-1}) 2922, 2227 (nitrile), 1598, 1475, 1455, 1355, 1158, 1090.

2ap ***N*-Benzyl-*N*-(5-(3-methoxyphenyl)-4-methyl-1-tosylindolin-7-yl)-4-methylbenzenesulfonamide & *N*-benzyl-*N*-(6-(3-methoxyphenyl)-4-methyl-1-tosylindolin-7-yl)-4-methylbenzenesulfonamide**
(isolated as 5:1 r.r., crude 4:1 r.r.)

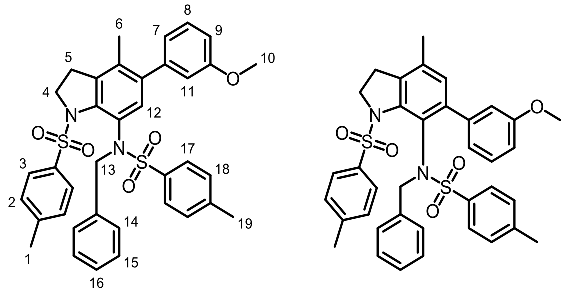

1a (52.0 mg, 0.10 mmol, 1.0 equiv.) was used in General Procedure **1** with 3-ethynylanisole (39.6 mg, 0.30 mmol, 3.0 equiv.) for 16 h with purification by flash column chromatography (SiO_2, EtOAc in pentane, 10 to 40%) to give the title compounds as an inseparable mixture (65.0 mg, 0.099 mmol, 99%) as an orange oil. *NMR data is given for the major isomer in the isolated mixture.*

R_f (EtOAc in pentane, 30%) 0.33.

^{1}H NMR (500 MHz, $CDCl_3$) δ_H 7.78 (2H, d, J = 8.4 Hz, H17), 7.57 (2H, d, J = 8.2 Hz, H3), 7.30–7.27 (2H, m, ArH), 7.22–7.15 (8H, m, ArH), 7.14–7.11 (1H, m, ArH), 6.85 (1H, s, ArH), 6.70–6.68 (1H, m, ArH), 6.64 (1H, t, J = 1.7 Hz, ArH), 5.18 (2H, s, H13), 3.95–3.85 (2H, m, H4), 3.83 (3H, s, H10), 2.40 (3H, s, CH3), 2.39 (3H, s, CH3), 2.20–2.10 (2H, m, H5), 1.88 (3H, s, H6).

^{13}C NMR (126 MHz, $CDCl_3$) δ_C 159.4, 144.1, 143.2, 141.7, 140.4, 139.3, 138.7, 137.4, 137.1, 135.1, 130.8, 130.4, 130.1, 129.6, 129.5, 129.4, 129.2, 128.5, 128.3, 128.1, 127.5, 122.0, 115.3, 112.5, 55.5, 53.2, 53.0, 28.9, 21.8, 21.7, 17.0.

HRMS (ES^+) calc. for $C_{37}H_{37}N_2O_5S_2$ ($[M+H]^+$) 653.2138, found 653.2150.

IR (thin film, v_{max} / cm^{-1}) 3063, 3030, 2952, 2920, 1598, 1470, 1355, 1167, 1159, 1090, 1046.

2aq *N*-Benzyl-*N*-(5-(2-methoxyphenyl)-4-methyl-1-tosylindolin-7-yl)-4-methylbenzenesulfonamide & ***N*-benzyl-*N*-(6-(2-methoxyphenyl)-4-methyl-1-tosylindolin-7-yl)-4-methylbenzenesulfonamide** *(isolated as 2:1 r.r., crude 2:1 r.r.)*

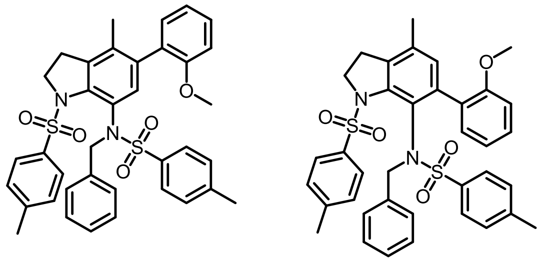

1a (52.0 mg, 0.10 mmol, 1.0 equiv.) was used in General Procedure **1** with 2-ethynylanisole (39.6 mg, 0.30 mmol, 3.0 equiv.) for 16 h with purification by flash column chromatography (SiO_2, EtOAc in pentane, 10 to 20%) to give the title compounds as an inseparable mixture (58.9 mg, 0.090 mmol, 90%) as a yellow foam. *NMR data is given for the mixture of isomers in the mixture as the peaks could not be satisfactorily assigned individually.*

R_f (EtOAc in pentane, 20%) 0.35.

^{1}H NMR (400 MHz, $CDCl_3$) δ_H 7.83 (1H, d, *J* = 8.3 Hz), 7.74 (2H, d, *J* = 8.2 Hz), 7.71 (1H, d, *J* = 8.3 Hz), 7.54 (1H, d, *J* = 8.4 Hz), 7.39–7.27 (4H, m), 7.23–7.06 (13H, m), 7.00–6.64 (5H, m), 5.18 (2H, s), 4.81 (1H, s), 3.99–3.81 (5H, m), 3.71 (2H, s), 3.49 (1H, t, *J* = 7.2 Hz), 2.43 (2H, s), 2.39 (3H, s), 2.36 (3H, s), 2.33 (2H, s), 2.22–2.10 (2H, m), 2.03 (1H, d, *J* = 11.4 Hz), 1.72 (3H, s), 1.62 (2H, d, *J* = 7.0 Hz).

^{13}C NMR (126 MHz, $CDCl_3$) δ_C 160.1, 156.7, 144.0, 143.9, 143.8, 143.0, 142.7, 138.5, 137.7, 137.5, 137.2, 136.0, 135.5, 135.1, 134.3, 133.4, 132.5, 131.3, 130.6, 130.4, 129.5, 129.44, 129.38, 129.2, 129.01, 128.96, 128.6, 128.5, 128.3, 128.2, 128.1, 127.8, 127.44, 127.35, 127.3, 126.2, 120.6, 120.4, 112.9, 112.0, 111.2, 110.6, 96.3, 91.2, 55.8, 55.6, 54.5, 53.3, 52.9, 48.2, 29.8, 28.8, 27.0, 21.8, 21.64, 21.58, 16.6, 16.1 *Four aromatic peaks not found for minor isomer.*

HRMS (ES^+) calc. for $C_{37}H_{37}O_5N_2S_2$ ($[M+H]^+$) 653.2138, found 653.2136.

IR (thin film, ν_{max} / cm^{-1}) 2900, 1597, 1494, 1462, 1381, 1355, 1246, 1160, 1090.

2ar ***N*-Benzyl-*N*-(5,6-bis(methoxymethyl)-4-methyl-1-tosylindolin-7-yl)-4-methylbenzenesulfonamide**

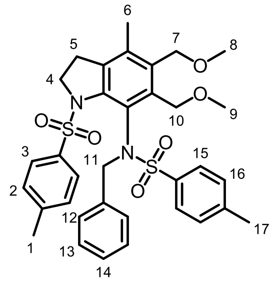

1a (52.0 mg, 0.10 mmol, 1.0 equiv.) was used in General Procedure **1** with 1,4-dimethoxybut-2-yne (34.2 mg, 0.30 mmol, 3.0 equiv.) for 16 h with purification by flash column chromatography (SiO_2, EtOAc in pentane, 10 to 40%) to give the title compound (20.8 mg, 0.033 mmol, 33%) as a viscous yellow oil.

R$_f$ (EtOAc in pentane, 40%) 0.39.

^{1}H NMR (400 MHz, $CDCl_3$) δ_H 7.72 (2H, d, *J* = 8.3 Hz, ArH), 7.54 (2H, d, *J* = 8.3 Hz, ArH), 7.42–7.39 (2H, m, ArH), 7.24–7.14 (7H, m, ArH), 5.14 (1H, d, *J* = 13.9 Hz, H11), 5.07 (1H, d, *J* = 13.9 Hz, H11), 4.49 (1H, d, *J* = 11.5 Hz, CH_2), 4.41 (1H, d, *J* = 11.4 Hz, CH_2), 4.08 (1H, d, *J* = 11.0 Hz, CH_2), 4.00 (1H, d, *J* = 11.0 Hz, CH_2), 3.95–3.89 (1H, m, H4), 3.64–3.56 (1H, m, H4), 3.31 (3H, s, CH_3), 3.16 (3H, s, CH_3), 2.43 (3H, s, CH_3), 2.41 (3H, s, CH_3), 2.23–2.17 (1H, m, H5), 2.13–2.03 (4H, m, H5 + H6).

^{13}C NMR (101 MHz, $CDCl_3$) δ_C 144.4, 142.9, 141.3, 140.3, 139.9, 138.9, 137.6, 136.5, 135.6, 135.0, 131.8, 130.8, 129.6, 129.1, 128.4, 128.3, 128.0, 127.9, 68.7, 68.4, 58.4, 58.1, 56.5, 53.0, 29.1, 21.8, 21.7, 16.2.

HRMS (ES$^+$) calc. for $C_{34}H_{38}O_6N_2S_2Na$ ($[M+Na]^+$) 657.2063, found 657.2059.

IR (thin film, ν_{max} / cm^{-1}) 2932, 1448, 1330, 1159, 1090, 1003, 913, 815, 727, 666.

2as ***N*-Benzyl-*N*-(5,6-diethyl-4-methyl-1-tosylindolin-7-yl)-4-methylbenzenesulfonamide**

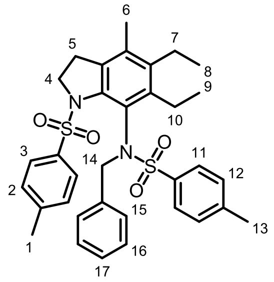

1a (52.0 mg, 0.10 mmol, 1.0 equiv.) was used in General Procedure **1** with 3-hexyne (24.6 mg, 0.30 mmol, 3.0 equiv.) for 16 h with purification by flash column chromatography (EtOAc in pentane, 0 to 20%) to give the title compound (31.0 mg, 0.051 mmol, 51%) as a sticky white gum.

R$_f$ (EtOAc in pentane, 20%) 0.32.

^{1}**H NMR** (400 MHz, $CDCl_3$) δ_H 7.70 (2H, d, *J* = 8.3 Hz, ArH), 7.44–7.41 (4H, m, ArH), 7.20–7.12 (7H, m, ArH), 5.08 (1H, d, *J* = 13.6 Hz, H14), 5.02 (1H, d, *J* = 13.6 Hz, H14), 3.94 (1H, ddd, *J* = 12.7, 7.3, 1.6 Hz, H4), 3.68 (1H, ddd, *J* = 12.7, 10.8, 7.8 Hz, H4), 2.64 (2H, qd, *J* = 7.3, 2.3 Hz, H7), 2.55 (1H, dt, *J* = 14.8, 7.4 Hz, H10), 2.43–2.39 (7H, m, H1 + H13 + H10), 2.23–2.17 (1H, m, H5), 2.05–1.98 (4H, m, H6 + H4), 1.09–1.03 (6H, m, H8 + H9).

^{13}**C NMR** (126 MHz, $CDCl_3$) δ_C 145.2, 144.2, 142.6, 142.3, 139.5, 139.2, 137.6, 135.8, 134.9, 133.4, 131.6, 130.7, 129.4, 129.0, 128.4 (2 C), 127.8, 127.6, 56.3, 52.9, 29.2, 22.8, 22.7, 21.8, 21.7, 16.2, 16.0, 14.8.

HRMS (ES^+) calc. for $C_{34}H_{38}O_4N_2NaS_2$ ($[M+Na]^+$) 625.2165, found 625.2164.

IR (thin film, v_{max} / cm^{-1}) 2966, 2925, 1598, 1495, 1455, 1353, 1334, 1159, 1090, 1040.

2at *N*-Benzyl-*N*-(5-(hydroxymethyl)-4-methyl-1-tosylindolin-7-yl)-4-methylbenzenesulfonamide and **2at' *N*-Benzyl-*N*-(6-(hydroxymethyl)-4-methyl-1-tosylindolin-7-yl)-4-methylbenzenesulfonamide**

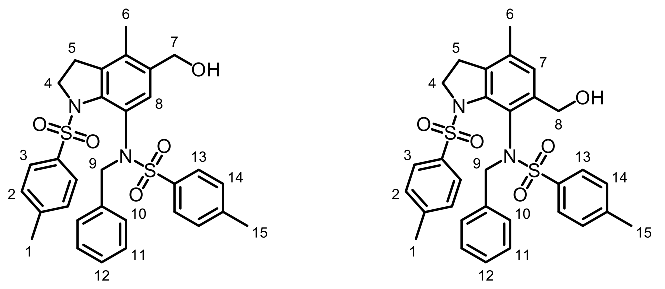

c(52.0 mg, 0.10 mmol, 1.0 equiv.) was used in General Procedure **1a** with propargyl alcohol (16.8 mg, 0.30 mmol, 3.0 equiv.) for 16 h with purification by flash column chromatography (SiO_2, EtOAc in pentane, 20 to 50%) to give the title compounds (37.6 mg, 0.065 mmol, 65%) as a white foam and (14.7 mg, 0.026 mmol, 26%) as a colourless oil respectively.

2at *N*-Benzyl-*N*-(5-(hydroxymethyl)-4-methyl-1-tosylindolin-7-yl)-4-methylbenzenesulfonamide

$\mathbf{R_f}$ (EtOAc in pentane, 50%) 0.27.

^{1}**H NMR** (500 MHz, $CDCl_3$) δ_H 7.82 (2H, d, *J* = 8.3 Hz, ArH), 7.50 (2H, d, *J* = 8.4 Hz, ArH), 7.28–7.24 (2H, m, ArH), 7.19–7.14 (7H, m, ArH), 6.97 (1H, s, H8), 5.17 (2H, s, H9), 4.47 (2H, d, *J* = 6.1 Hz, H7), 3.91–3.69 (2H, m, H4), 2.42 (3H, s, CH_3), 2.39 (3H, s, CH_3), 2.11–2.01 (2H, m, H5), 1.93 (3H, s, H6), 1.40 (1H, t, *J* = 6.1, OH).

^{13}**C NMR** (126 MHz, $CDCl_3$) δ_C 144.2, 143.3, 139.2, 138.8, 137.7, 137.6, 137.1, 135.0, 131.5, 130.2, 129.6, 129.5, 129.4, 128.8, 128.5, 128.3, 128.0, 127.5, 63.1, 53.1, 52.9, 28.3, 21.8, 21.7, 15.0.

HRMS (ES^+) calc. for $C_{31}H_{33}O_5N_2S_2$ ($[M+H]^+$) 577.1825, found 577.1823.

IR (thin film, v_{max} / cm^{-1}) 2975, 2364, 1597, 1353, 1167, 1157, 1090, 816, 727, 674, 672.

MP ($CHCl_3$) 130–132 °C.

2at' *N*-Benzyl-*N*-(6-(hydroxymethyl)-4-methyl-1-tosylindolin-7-yl)-4-methylbenzenesulfonamide

R$_f$ (EtOAc in pentane, 50%) 0.42.

^{1}H NMR (500 MHz, $CDCl_3$) δ_H 7.79 (2H, d, *J* = 8.4 Hz, ArH), 7.45 (2H, d, *J* = 8.2 Hz, ArH), 7.38 (2H, dd, *J* = 6.5, 2.9 Hz, ArH), 7.29–7.25 (5H, m, ArH), 7.21 (2H, d, *J* = 8.2 Hz, ArH), 7.13 (1H, s, H7), 5.25 (1H, d, *J* = 14.6 Hz, H9), 5.19 (1H, d, *J* = 14.6 Hz, H9), 4.11 (1H, dd, *J* = 12.8, 5.2 Hz, H8), 3.80 (1H, ddd, *J* = 12.9, 6.4, 4.3 Hz, H4), 3.72 (1H, dd, *J* = 12.8, 9.0 Hz, H8), 3.38 (1H, dt, *J* = 12.8, 8.7 Hz, H4), 2.44–2.42 (6H, m, H1 + H15), 2.09–2.04 (5H, m, H5 + H6), 1.94 (1H, dd, *J* = 9.0, 5.2 Hz, OH).

^{13}C NMR (126 MHz, $CDCl_3$) δ_C 144.5, 144.4, 143.4, 141.0, 139.2, 138.5, 136.3, 135.6, 135.1, 131.6, 131.0, 129.6, 129.3, 128.6, 128.3, 128.0, 61.6, 55.4, 53.5, 28.4, 21.8, 21.7, 18.9 (*Two aromatic carbon peaks could not be identified due to overlap)*.

HRMS (ES^+) calc. for $C_{31}H_{32}O_5N_2NaS_2$ ($[M+Na]^+$) 599.1645, found 599.1641.

IR (thin film, ν_{max} / cm^{-1}) 3552, 2924, 1598, 1496, 1448, 1355, 1328, 1155, 1090, 1041, 912, 814, 736, 650.

MP ($CHCl_3$) 105–107 °C.

2au *N*-Benzyl-*N*-(6-ethyl-5-(hydroxymethyl)-4-methyl-1-tosylindolin-7-yl)-4-methylbenzenesulfonamide and **2au' *N*-Benzyl-*N*-(5-ethyl-6-(hydroxymethyl)-4-methyl-1-tosylindolin-7-yl)-4-methylbenzenesulfonamide**

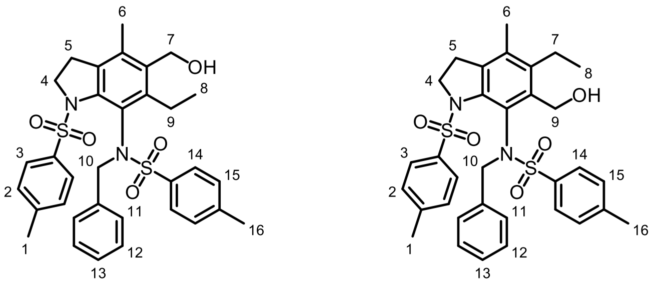

1a (52.0 mg, 0.10 mmol, 1.0 equiv.) was used in General Procedure **1** with 2-pentyn-1-ol (25.2 mg, 0.30 mmol, 3.0 equiv.) for 16 h with purification by flash column chromatography (SiO_2, EtOAc in pentane, 10 to 50%) to give the title compounds (29.6 mg, 0.049 mmol, 49%) as a brown oil and (31.4 mg, 0.051 mmol, 51%) as a pale cream foam respectively.

2au *N*-Benzyl-*N*-(6-ethyl-5-(hydroxymethyl)-4-methyl-1-tosylindolin-7-yl)-4-methylbenzenesulfonamide

R$_f$ (EtOAc in pentane, 50%) 0.23.

^{1}H NMR (400 MHz, $CDCl_3$) δ_H 7.68 (2H, d, *J* = 8.3 Hz, H14), 7.49 (2H, d, *J* = 8.3 Hz, H3), 7.45–7.41 (2H, m, ArH), 7.24–7.21 (2H, m, ArH), 7.20–7.11 (5H, m, ArH), 5.10 (1H, d, *J* = 13.6 Hz, H10), 5.03 (1H, d, *J* = 13.6 Hz, H10), 4.69 (2H, s, H7), 3.97–3.92 (1H, m, H4), 3.72–3.64 (1H, m, H4), 2.75–2.65 (1H, m, H9), 2.58 (1H, dt, *J* = 14.7, 7.5 Hz, H9), 2.44 (3H, s, CH_3), 2.40 (3H, s, CH_3), 2.27–2.21 (1H, m, H5), 2.17 (3H, s, H6), 2.13–2.05 (1H, m, H5), 1.06 (3H, t, *J* = 7.5 Hz, H8). *OH peak not observed.*

^{13}C NMR (101 MHz, $CDCl_3$) δ_C 146.7, 144.4, 142.8, 141.8, 139.1, 138.1, 137.7, 136.0, 135.5, 135.0, 131.6, 131.0, 129.6, 129.0, 128.3 (2 C), 127.9, 127.8, 59.6, 56.2, 53.0, 29.1, 23.0, 21.8, 21.7, 16.9, 16.2.

HRMS (ES$^+$) calc. for $C_{33}H_{36}O_5N_2NaS_2$ ([M+Na]$^+$) 627.1958, found 627.1960.

IR (thin film, v_{max} / cm^{-1}) 2957, 2925, 2360, 1455, 1332, 1163, 1089, 1030, 1005, 913, 814, 774, 732, 700, 664.

2au’ *N*-Benzyl-*N*-(5-ethyl-6-(hydroxymethyl)-4-methyl-1-tosylindolin-7-yl)-4-methylbenzene sulfonamide

R$_f$ (EtOAc in pentane, 50%) 0.50.

^{1}H NMR (500 MHz, $CDCl_3$) δ_H 7.80 (2H, d, *J* = 8.3 Hz, ArH), 7.41 (2H, d, *J* = 8.3 Hz, ArH), 7.33 (2H, dd, *J* = 7.6, 1.9 Hz, ArH), 7.30–7.25 (6H, m, ArH), 7.20 (1H, d, *J* = 8.3 Hz, H13), 5.27 (1H, d, *J* = 14.5 Hz, H10), 5.19 (1H, d, *J* = 14.5 Hz, H10), 4.13 (1H, dd, *J* = 13.0, 4.4 Hz, H9), 3.83–3.71 (2H, m, H9 + H4), 3.33–3.27 (1H, ddd, *J* = 12.7, 9.5, 7.8 Hz, H4), 2.89–2.82 (1H, dq, *J* = 14.9, 7.5 Hz, H7), 2.62 (1H, dt, *J* = 14.9, 7.5 Hz, H7), 2.46–2.42 (6H, m, H1 + H16), 2.35–2.30 (1H, m, OH), 2.22–2.09 (2H, m, H5), 2.03 (3H, s, H6), 1.02 (3H, t, *J* = 7.5 Hz, H8).

^{13}C NMR (101 MHz, $CDCl_3$) δ_C 144.5, 144.3, 143.3, 141.8, 140.0, 138.7, 138.5, 136.1, 135.2, 134.4, 131.0, 129.6, 129.3, 128.8, 128.5, 128.3, 128.2, 128.0, 58.5, 55.7, 53.3, 29.5, 22.7, 21.8, 21.7, 16.0, 14.7.

HRMS (ES$^+$) calc. for $C_{33}H_{36}O_5N_2NaS_2$ ([M+Na]$^+$) 627.1958, found 627.1949.

IR (thin film, v_{max} / cm^{-1}) 3541, 2960, 2924, 1598, 1454, 1356, 1328, 1155, 1090, 1039.

2av & **2av’** ***N*-Benzyl-*N*-(6-butyl-5-(1-hydroxyethyl)-4-methyl-1-tosylindolin-7-yl)-4-methylbenzenesulfonamide & *N*-Benzyl-*N*-(5-butyl-6-(1-hydroxyethyl)-4-methyl-1-tosylindolin-7-yl)-4-methylbenzenesulfonamide** *(isolated as 1 : 1 r.r., crude 1 : 1 r.r.)*

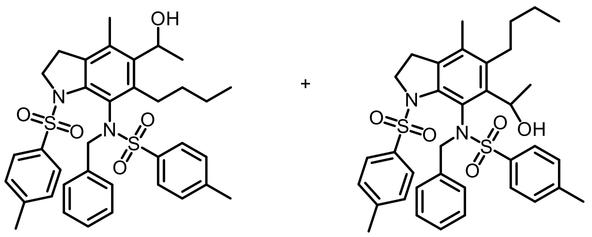

1a (52.0 mg, 0.10 mmol, 1.0 equiv.) was used in General Procedure **1** with oct-3-yn-2-ol (37.8 mg, 0.30 mmol, 3.0 equiv.) for 16 h with purification by flash column chromatography (SiO_2, EtOAc in pentane, 10 to 50%) to give the title compounds as an inseparable mixture (31.7 mg, 0.049 mmol, 49%) as a yellow foam.

R$_f$ (EtOAc in pentane, 50%) 0.60.

^{1}H NMR (500 MHz, $CDCl_3$) δ_H 8.01 (2H, d, *J* = 8.2 Hz), 7.60 (2H, d, *J* = 8.4 Hz), 7.55 (2H, dt, *J* = 5.7, 1.6 Hz), 7.46 (2H, dd, *J* = 6.7, 3.0 Hz), 7.44 (2H, d, *J* = 8.2 Hz), 7.38–7.33 (8H, m), 7.30 (2H, d, *J* = 7.4 Hz), 7.22 (2H, d, *J* = 8.1 Hz), 7.18 (4H, d, *J* = 7.9 Hz), 5.47 (1H, d, *J* = 14.4 Hz), 5.36 (1H, d, *J* = 14.4 Hz), 5.27 (1H, d, *J* =

13.9 Hz), 5.20 (1H, qd, *J* = 7.0, 3.1 Hz), 5.02 (1H, d, *J* = 13.9 Hz), 4.87 (1H, qd, *J* = 6.8, 3.8 Hz), 3.94 (1H, ddd, *J* = 12.8, 7.2, 1.6 Hz), 3.66 (1H, ddd, *J* = 12.8, 7.2, 2.6 Hz), 3.58 (1H, ddd, *J* =1 2.8, 11.0, 7.8 Hz), 3.19–3.06 (2H, m), 2.98–2.90 (2H, m), 2.75–2.67 (1H, m), 2.55 (1H, ddd, *J* = 12.8, 10.6, 4.8 Hz), 2.46 (3H, s), 2.45 (3H, s), 2.43 (3H, s), 2.39 (3H, s), 2.22–2.15 (2H, m), 2.08 (2H, ddd, *J* = 14.9, 10.4, 7.4 Hz), 2.01 (3H, s), 1.99 (3H, s), 1.46–1.40 (3H, m), 1.36–1.29 (6H, m), 1.27–1.18 (3H, m), 0.95 (3H, t, *J* = 7.1 Hz), 0.91 (3H, t, *J* =7.3 Hz), 0.79 (3H, d, *J* = 6.9 Hz).

^{13}C NMR (126 MHz, $CDCl_3$) δ_C 145.5, 145.1, 144.4, 143.5, 143.4, 143.3, 143.2, 139.8, 139.6, 139.0, 138.6, 138.5, 138.4, 136.5, 136.3, 135.6, 135.4, 134.9, 134.8, 131.7, 130.9, 129.53, 129.47, 129.46, 129.1, 128.66, 128.65, 128.6, 128.51, 128.46, 128.24, 128.19, 128.14, 128.11, 127.7, 67.4, 66.4, 55.0, 54.4, 53.3, 53.0, 33.7, 33.3, 29.9, 29.8, 29.5, 29.4, 23.3, 23.2, 23.0, 21.79, 21.77, 21.76, 21.7, 21.3, 16.1, 16.0, 14.2, 14.1 (O*ne aromatic carbon peak not identified)*.

HRMS (ES^+) calc. for $C_{36}H_{42}O_5N_2S_2Na$ ($[M+Na]^+$) 669.2427, found 669.2421.

IR (thin film, v_{max} / cm^{-1}) 3300 (br), 2986, 2160, 2500 (br), 2028, 1977, 1673, 1349, 1162, 1089, 1029.

2ba ***N*-Butyl-*N*-(4-(4-fluorophenyl)-5,6-bis(hydroxymethyl)-1-tosylindolin-7-yl)-4-methylbenzenesulfonamide**

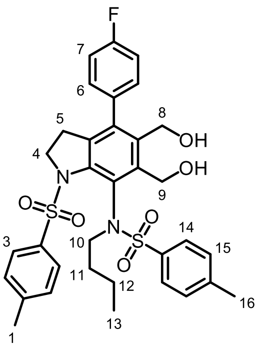

1b (56.6 mg, 0.10 mmol, 1.0 equiv.) was used in General Procedure **1** with 2-butyne-1,4-diol (25.8 mg, 0.30 mmol, 3.0 equiv.) for 16 h with purification by flash column chromatography (SiO_2, EtOAc in pentane, 10 to 30%) to give the title compound (33.5 mg, 0.051 mmol, 51%) as a cream foam.

R_f (EtOAc in pentane, 30%) 0.24.

^{1}H NMR (400 MHz, $CDCl_3$) δ_H 7.85 (2H, d, *J* = 8.3 Hz, ArH), 7.54 (1H, ddd, *J* = 8.2, 5.4, 2.3 Hz, H6), 7.37 (2H, d, *J* = 8.3 Hz, ArH), 7.33–7.30 (2H, m, ArH), 7.26–7.23 (2H, m, ArH), 7.12 (1H, td, *J* = 8.6, 2.8 Hz, H7), 6.94 (1H, td, *J* = 8.6, 2.8 Hz, H7), 6.61 (1H, ddd, *J* = 8.2, 5.4, 2.3 Hz, H6), 5.11 (1H, d, *J* = 12.7 Hz), 4.85 (1H, t, *J* = 11.4 Hz), 4.71–4.69 (1H, m), 4.25 (1H, d, *J* = 5.8 Hz), 3.92–3.88 (2H, m, H10), 3.68 (2H, ddd, *J* = 13.0, 6.6, 2.5 Hz), 3.14 (1H, ddd, *J* = 12.9, 10.9, 8.6 Hz), 2.46 (3H, s, CH_3), 2.44 (3H, s, CH_3), 1.77–1.64 (4H, m, H5 + H11), 1.34 (1H, q, *J* = 7.4 Hz, H12), 0.91 (3H, t, *J* = 7.4 Hz, H13) *OH protons not observed*.

^{13}C NMR (101 MHz, $CDCl_3$) δ_C 162.5 (d, $^1J_{C-F}$ = 247.5 Hz), 144.6, 143.84, 143.82, 141.8, 140.6, 140.2, 140.1, 137.5, 134.9, 133.7 (d, $^4J_{C-F}$ = 3.6 Hz), 131.7 (d, $^3J_{C-F}$ = 8.0 Hz), 131.4, 130.2 (d, $^3J_{C-F}$ = 8.1 Hz), 129.7, 129.5,

128.3, 128.0, 115.7 (d, $^2J_{C-F}$ = 21.1 Hz), 115.2 (d, $^2J_{C-F}$ = 21.9 Hz), 60.4, 60.0, 53.4, 52.9, 30.5, 29.9, 21.76, 21.75, 20.6, 14.1. *Two additional aromatic carbon signals observed due to diastereotopic character of 4-C_6H_4F ring.*

^{19}F NMR (377 MHz, $CDCl_3$): δ_F -113.85.

HRMS (ES$^+$) calc. for $C_{34}H_{37}O_6N_2FNaS_2$ ([M+Na]$^+$) 675.1969, found 675.1967.

IR (thin film, ν_{max} / cm^{-1}) 2988, 1598, 1511, 1358, 1331, 1165, 1090, 1032.

2cb ***N*-Butyl-*N*-(5-(4-(dimethylamino)phenyl)-4-phenyl-1-tosylindolin-7-yl)-4-methylbenzenesulfonamide** & ***N*-butyl-*N*-(6-(4-(dimethylamino)phenyl)-4-phenyl-1-tosylindolin-7-yl)-4-methylbenzenesulfonamide** *(isolated as 5:1 r.r., crude 5:1 r.r.)*

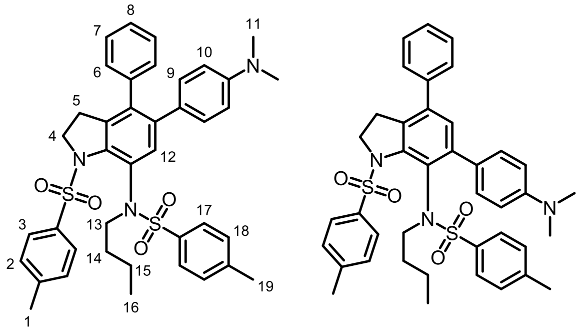

1c (54.8 mg, 0.10 mmol, 1.0 equiv.) was used in General Procedure **1** with 4-ethynyl-*N*,*N*-dimethylaniline (43.6 mg, 0.30 mmol, 3.0 equiv.) for 16 h with purification by flash column chromatography (SiO_2, EtOAc in pentane, 10 to 30%) to give the title compounds as an inseparable mixture (20.0 mg, 0.029 mmol, 29%) as a brown foam. *NMR data is given for the major isomer in the isolated mixture.*

R_f (EtOAc in pentane, 20%) 0.19.

^{1}H NMR (400 MHz, $CDCl_3$) δ_H 7.82 (2H, d, *J* = 8.2 Hz, H17), 7.71 (2H, d, *J* = 8.2 Hz, H3), 7.33 (2H, d, *J* = 8.2 Hz, ArH), 7.28 (2H, d, *J* = 8.2 Hz, ArH), 7.22–7.15 (3H, m, ArH), 7.06 (1H, s, H12), 6.87–6.80 (4H, m, ArH), 6.50 (2H, d, *J* = 8.9 Hz, H10), 3.93–3.87 (2H, m, H4), 3.79–3.70 (2H, m, H13), 2.90 (6H, s, H11), 2.46 (3H, s, CH_3), 2.45 (3H, s, CH_3), 2.30–2.12 (2H, m, H5), 1.67 (2H, app. p, *J* = 7.9 Hz, H14), 1.29–1.17 (2H, m, H15), 0.85 (3H, t, *J* = 7.4 Hz, H16).

^{13}C NMR (101 MHz, $CDCl_3$) δ_C 149.3, 144.0, 143.3, 139.9, 139.5, 139.2, 137.1, 135.8, 132.1, 130.5, 129.7, 129.52, 129.50, 129.4 (2 C), 128.6, 128.14, 128.11, 128.06, 127.8, 126.9, 111.8, 53.1, 50.5, 40.5, 30.33, 30.27, 21.7 (2 C), 20.6, 13.9.

HRMS (ES$^+$) calc. for $C_{40}H_{44}O_4N_3S_2$ 694.2768, found 694.2764.

IR (thin film, ν_{max} / cm^{-1}) 2979, 1611, 1525, 1355, 1167, 1089.

2aw ***N*-Benzyl-4-methyl-*N*-(4-methyl-1-tosyl-6-(trimethylsilyl)indolin-7-yl)benzenesulfonamide** & ***N*-benzyl-4-methyl-*N*-(4-methyl-1-tosyl-5-(trimethylsilyl)indolin-7-yl)benzenesulfonamide** *(isolated as 6:1 r.r., crude 6:1 r.r.)*

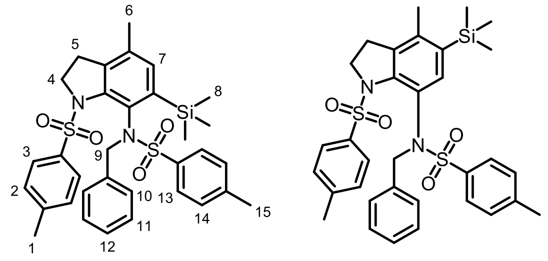

1a (52.0 mg, 0.10 mmol, 1.0 equiv.) was used in General Procedure **1** (using a sealed tube) with trimethylsilylacetylene (0.14 mL, 1.0 mmol, 10.0 equiv.) for 16 h with purification by flash column chromatography (SiO_2, Et_2O in pentane, 10 to 40% + 1% Et_3N) to give the title compounds as an inseparable mixture (43.8 mg, 0.071 mmol, 71%) as a yellow foam. *NMR data is given for the major isomer in the isolated mixture.*

R_f (Et_2O in pentane, 30%) 0.18.

^{1}H NMR (400 MHz, $CDCl_3$) δ_H 7.82 (2H, d, *J* = 8.3 Hz, H13), 7.69 (2H, d, *J* = 8.3 Hz, H3), 7.29–7.26 (2H, m, ArH), 7.25–7.21 (2H, m, ArH), 7.17–7.11 (4H, m, ArH), 7.10–7.06 (1H, m, ArH), 6.82 (1H, qt, *J* = 7.1, 2.6 Hz, H7), 4.76 (2H, s, H9), 3.47 (2H, t, *J* = 7.3 Hz, H4), 2.42 (3H, s, CH_3), 2.38 (3H, s, CH_3), 2.03–1.97 (2H, m, H5), 1.57–1.53 (3H, m, H6), 0.16 (9H, s, H8).

^{13}C NMR (101 MHz, $CDCl_3$) δ_C 145.1, 144.0, 142.9, 138.0, 135.9, 135.4, 134.3, 130.4, 129.5, 129.0, 128.5, 128.3, 127.8, 127.5, 126.5, 112.3, 105.2, 102.1, 54.1, 48.0, 27.0, 21.7, 21.6, 15.6, -0.3.

HRMS (ES$^+$) calc. for $C_{33}H_{39}O_4N_2S_2Si$ ([M+H]$^+$) 619.2115, found 619.2116.

IR (thin film, ν_{max} / cm^{-1}) 2920, 1598, 1456, 1355, 1250, 1163, 1090, 1018, 1008.

2ax ***N*-Benzyl-4-methyl-*N*-(4-methyl-1-tosyl-6-(triethylsilyl)indolin-7-yl)benzenesulfonamide**
(isolated as > 20:1 r.r., crude > 20:1 r.r.)

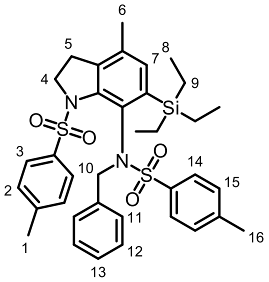

1a (52.0 mg, 0.10 mmol, 1.0 equiv.) was used in General Procedure **1** with triethylsilylacetylene (42.1 mg, 0.30 mmol, 3.0 equiv.) for 16 h with purification by flash column chromatography (SiO_2, EtOAc in pentane, 10 to 20%) to give the title compound (22.3 mg, 0.034 mmol, 34%) as a pale brown amorphous solid.

R$_f$ (EtOAc in pentane, 20%) 0.54.

^{1}H NMR (400 MHz, $CDCl_3$) δ_H 7.80 (2H, d, *J* = 8.3 Hz, H14), 7.65 (2H, d, *J* = 8.3 Hz, H3), 7.30–7.27 (4H, m, ArH, H2 + H15), 7.16–7.05 (5H, m, ArH, H11 + H12 + H13), 6.88 (1H, dt, *J* = 7.1, 2.7 Hz, H7), 4.72 (2H, s, H10), 3.49 (2H, t, *J* = 7.2 Hz, H4), 2.42 (3H, s, CH_3), 2.37 (3H, s, CH_3), 2.08–1.96 (2H, m, H5), 1.56 (3H, d, *J* = 7.1 Hz, H6), 0.98 (9H, t, *J* = 7.6 Hz, H8), 0.62 (6H, q, *J* = 7.6 Hz, H9).

^{13}C NMR (101 MHz, $CDCl_3$) δ_C 145.1, 144.0, 142.8, 137.9, 135.9, 135.4, 134.3, 130.5, 129.5, 129.0, 128.4, 128.3, 127.8, 127.4, 126.4, 112.7, 103.7, 102.8, 54.3, 47.9, 27.1, 21.7, 21.6, 15.6, 7.5, 4.3.

HRMS (ES$^+$) calc. for $C_{36}H_{45}O_4N_2S_2Si$ ($[M+H]^+$) 661.2585, found 661.2578.

IR (thin film, ν_{max} / cm^{-1}) 2980, 1355, 1164, 1090, 1007.

2ay ***N*-Benzyl-4-methyl-*N*-(4-methyl-5-(4,4,5,5-tetramethyl-1,3,2-dioxaborolan-2-yl)-1-tosylindolin-7-yl)benzenesulfonamide** *(isolated as > 20:1 r.r., crude 6:1 r.r.)*

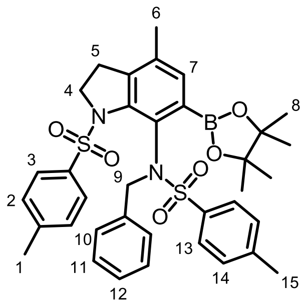

1a (52.0 mg, 0.10 mmol, 1.0 equiv.) was used in General Procedure **1** with 2-ethynyl-4,4,5,5-tetramethyl-1,3,2-dioxaborolane (45.6 mg, 0.30 mmol, 3.0 equiv.) for 16 h with purification by flash column chromatography (SiO_2, EtOAc in pentane, 40%) to give the title compound (17.7 mg, 0.026 mmol, 26%) as a viscous brown oil.

R$_f$ (EtOAc in pentane, 20%) 0.24.

^{1}H NMR (500 MHz, $CDCl_3$) δ_H 7.79 (2H, d, *J* = 8.2 Hz, H13), 7.51 (2H, d, *J* = 8.3 Hz, H3), 7.37 (1H, br. s, H7), 7.23 (2H, d, *J* = 8.2 Hz, H14), 7.21–7.14 (7H, m, ArH), 5.15 (2H, s, H9), 3.93–3.69 (2H, m, H4), 2.43 (3H, s, CH_3), 2.38 (3H, s, CH_3), 2.11 (3H, s, H6), 2.05–2.00 (2H, m, H5), 1.29 (12H, s, H8).

^{13}C NMR (126 MHz, $CDCl_3$) δ_C 144.0, 142.8, 140.3, 139.8, 139.2, 138.3, 137.4, 137.0, 135.1, 129.8, 129.6, 129.2, 128.6, 128.52, 128.47, 128.2, 127.9, 127.4, 83.7, 53.6, 52.6, 28.2, 25.1, 21.8, 21.7, 18.2.

HRMS (ES$^+$) calc. for $C_{36}H_{42}O_6N_2BS_2$ ($[M+H]^+$) 673.2577, found 673.2572.

IR (thin film, ν_{max} / cm^{-1}) 1597, 1354, 1167, 1144, 1091.

2ds (7-(9*H*-Carbazol-9-yl)-4-methyl-1-tosylindolin-5-yl)methanol

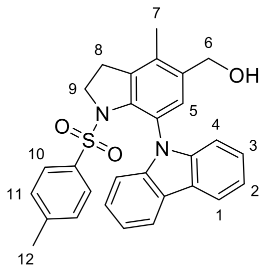

1d (42.7 mg, 0.10 mmol, 1.0 equiv.) was used in General Procedure **1** with propargyl alcohol (17.0 mg, 0.30 mmol, 3.0 equiv.) for 16 h with purification by flash column chromatography (SiO_2, EtOAc in pentane, 40%) to give the title compound (44.0 mg, 0.092 mmol, 92%) as a yellow solid.

R_f (EtOAc in pentane, 40%) 0.20.

^{1}H NMR (500 MHz, $CDCl_3$) δ_H 7.98 (2H, d, *J* = 7.7 Hz, H1), 7.37–7.31 (3H, m, H3 + H5), 7.25–7.18 (4H, m, H2 + H4), 6.81–6.75 (2H, m, H10), 6.68 (2H, d, *J* = 8.0 Hz, H11), 4.70 (2H, s, H6), 4.24 (2H, t, J = 7.3 Hz, H9), 2.95 (2H, t, *J* = 7.3 Hz, H8), 2.31 (3H, s, H7), 2.23 (3H, s, H12), 1.60 (1H, br. s, OH).

^{13}C NMR (126 MHz, $CDCl_3$) δ_C 143.0, 140.4, 139.1, 138.6, 138.3, 135.7, 132.5, 128.7, 127.3, 126.9, 126.3, 125.7, 123.8, 120.0, 119.8, 110.6, 63.2, 52.6, 29.9, 21.6, 15.4.

HRMS (ES^+) calc. for $C_{29}H_{27}O_3N_2S$ ($[M+H]^+$) 483.1737, found 483.1738.

IR (thin film, v_{max} / cm^{-1}) 1597, 1497, 1480, 1453, 1233, 1165, 751, 725.

MP ($CHCl_3$/pentane, 10%) 84–86 °C.

2db 4-(7-(9*H*-Carbazol-9-yl)-4-methyl-1-tosylindolin-5-yl)-*N*,*N*-dimethylaniline

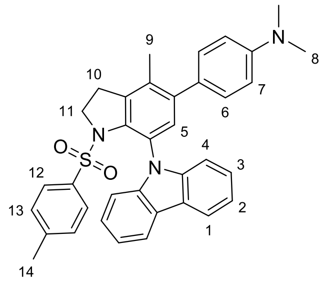

1d (42.7 mg, 0.10 mmol, 1.0 equiv.) was used in General Procedure **1** with 4-ethynyl-*N*,*N*-dimethylaniline (43.6 mg, 0.30 mmol, 3.0 equiv.) for 16 h with purification by flash column chromatography (SiO_2, EtOAc in pentane, 20%) then recrystallised ($CHCl_3$/pentane) to give the title compound (57.0 mg, 0.099 mmol, >99%) as a yellow solid.

R_f (EtOAc in pentane, 20%) 0.19.

^{1}H NMR (500 MHz, $CDCl_3$) δ_H 8.02 (2H, d, *J* = 7.7 Hz, H1), 7.40–7.35 (2H, m, H3), 7.33 (2H, d, *J* = 8.1 Hz, H12), 7.28 (1H, s, H5), 7.27–7.21 (4H, m, H2 + H13), 6.89–6.82 (2H, m, H6), 6.77 (2H, d, *J* = 8.8 Hz, H4), 6.74 (2H, d, *J* = 8.0 Hz, H7), 4.27 (2H, t, *J* = 7.3 Hz, H12), 3.00–2.96 (8H, m, H8 + H11), 2.29 (3H, s, H9), 2.26 (3H, s, H14).

^{13}C NMR (126 MHz, $CDCl_3$) δ_C 149.7, 142.9, 141.8, 140.4, 138.9, 137.0, 135.7, 131.7, 130.2, 128.9, 128.7, 128.3, 126.7, 126.4, 125.6, 123.7, 119.9, 119.6, 112.1, 110.8, 52.5, 40.6, 30.4, 21.6, 17.5.

HRMS (ES^+) calc. for $C_{36}H_{34}O_2N_3S$ ($[M+H]^+$) 572.2366, found 572.2372.

IR (thin film, ν_{max} / cm^{-1}) 1611, 1479, 1452, 1167, 909, 725.

MP ($CHCl_3$/pentane, 10%) 124–126 °C.

2de 9-(5-(4-Methoxyphenyl)-4-methyl-1-tosylindolin-7-yl)-9*H*-carbazole

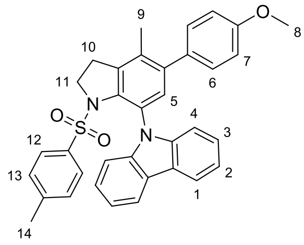

1d (42.7 mg, 0.10 mmol, 1.0 equiv.) was used in General Procedure **1** with 4-ethynylanisole (39.6 mg, 0.30 mmol, 3.0 equiv.) for 16 h with purification by flash column chromatography (SiO_2, EtOAc in pentane, 20%) then recrystallised ($CHCl_3$/pentane) to give the title compound (52.0 mg, 0.093 mmol, 93%) as a yellow solid.

R_f (EtOAc in pentane, 20%) 0.40.

^{1}H NMR (500 MHz, $CDCl_3$) δ_H 7.98 (2H, d, *J* = 7.7 Hz, H1), 7.37–7.32 (2H, m, H3), 7.28 (1H, s, H5), 7.28–7.25 (2H, m, H12), 7.25–7.20 (4H, m, H2 + H13), 6.94 (2H, dt, *J* = 8.6, 2.8 Hz, H4), 6.80 (2H, dt, *J* = 8.1, 2.2 Hz, H6), 6.69 (2H, d, *J* = 8.1 Hz, H7), 4.27 (2H, t, *J* = 7.3 Hz, H11), 3.83 (3H, s, H8), 3.01 (2H, t, *J* = 7.3 Hz, H10), 2.24 (6H, m, H9 + H14).

^{13}C NMR (126 MHz, $CDCl_3$) δ_C 159.0, 143.0, 141.3, 140.4, 139.0, 137.7, 135.7, 132.8, 131.8, 130.5, 129.1, 128.7, 126.8, 126.4, 125.7, 123.8, 120.0, 119.7, 113.8, 110.7, 55.4, 52.6, 30.4, 21.6, 17.4.

HRMS (ES^+) calc. for $C_{35}H_{31}O_3N_2S$ ($[M+H]^+$) 559.2050, found 559.2049.

IR (thin film, ν_{max} / cm^{-1}) 1611, 1479, 1452, 1167, 909, 725.

MP ($CHCl_3$/pentane, 10%) 124–126 °C.

2df 9-(4-Methyl-5-phenyl-1-tosylindolin-7-yl)-9*H*-carbazole

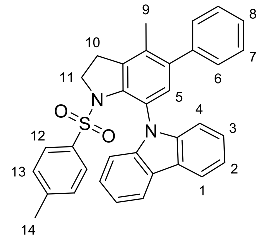

1d (42.7 mg, 0.10 mmol, 1.0 equiv.) was used in General Procedure **1** with phenylacetylene (30.6 mg, 0.30 mmol, 3.0 equiv.) for 16 h with purification by flash column chromatography (SiO_2, EtOAc in pentane, 20%) then recrystallised ($CHCl_3$/pentane) to give the title compound (53.0 mg, 0.10 mmol, >99%) as a yellow solid.

R_f (EtOAc in pentane, 20%) 0.31.

^{1}H NMR (500 MHz, $CDCl_3$) δ_H 7.99 (2H, d, *J* = 7.7 Hz, H1), 7.43–7.37 (2H, m, H3), 7.36–7.31 (5H, m, H6, H7 + H8), 7.29 (2H, d, *J* = 8.1 Hz, H4), 7.25 (1H, s, H5), 7.25–7.21 (2H, m, H2), 6.84–6.78 (2H, m, H12), 6.70 (2H, d, *J* = 8.1 Hz, H13), 4.29 (2H, t, *J* = 7.3 Hz, H11), 3.02 (2H, t, *J* = 7.3 Hz, H10), 2.24 (6H, s, H14 + H9).

^{13}C NMR (126 MHz, $CDCl_3$) δ_C 143.0, 141.6, 140.41, 140.37, 139.0, 137.9, 135.7, 131.7, 129.4, 129.1, 128.7, 128.3, 127.3, 126.8, 126.3, 125.7, 123.8, 120.0, 119.8, 110.7, 52.6, 30.4, 21.6, 17.4.

HRMS (ES^+) calc. for $C_{34}H_{29}O_2N_2S$ ($[M+H]^+$) 529.1944, found 529.1948.

IR (thin film, ν_{max} / cm^{-1}) 1478, 1452, 1233, 1184, 910, 750, 724.

MP ($CHCl_3$/pentane, 10%) 98–100 °C.

2di 9-(4-Methyl-5-phenyl-1-tosylindolin-7-yl)-9*H*-carbazole

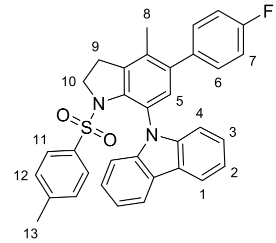

1d (42.7 mg, 0.10 mmol, 1.0 equiv.) was used in General Procedure **1** with 4-fluorophenylacetylene (36.0 mg, 0.30 mmol, 3.0 equiv.) for 16 h with purification by flash column chromatography (SiO_2, EtOAc in pentane, 20%) then recrystallised ($CHCl_3$/pentane) to give the title compound (55.0 mg, 0.099 mmol, >99%) as a yellow solid.

R_f (EtOAc in pentane, 20%) 0.35.

^{1}H NMR (500 MHz, $CDCl_3$) δ_H 7.99 (2H, dt, *J* = 7.7, 0.9 Hz, H1), 7.38–7.34 (2H, m, H3), 7.30–7.26 (4H, m, H6 and H4), 7.25–7.21 (2H, m, H2), 7.20 (1H, s, H5), 7.09 (2H, tt, *J* = 8.9, 2.9 Hz, H7), 6.82–6.76 (2H, m, H11),

6.69 (2H, d, J = 8.0 Hz, H12), 4.29 (2H, t, J = 7.3 Hz, H10), 3.03 (2H, t, J = 7.3 Hz, H9), 2.24 (3H, s, H13), 2.22 (3H, s, H8).

^{13}C NMR (126 MHz, $CDCl_3$) δ_C 162.3 (d, $^1J_{C-F}$ = 246.6 Hz), 143.0, 140.5, 140.3, 139.1, 138.2, 136.3 (d, $^4J_{C-F}$ = 3.3 Hz), 135.7, 131.7, 131.0 (d, $^3J_{C-F}$ = 8.0 Hz), 129.0, 128.7, 126.9, 126.3, 125.7, 123.8, 120.0, 119.8, 115.3 (d, $^2J_{C-F}$ = 21.4 Hz), 110.7, 52.5, 30.4, 21.6, 17.3.

^{19}F NMR (377 MHz, $CDCl_3$) δ_F -115.2.

HRMS (ES^+) calc. for $C_{34}H_{28}FN_2O_2S$ ($[M+H]^+$) 547.1850, found 547.1859.

IR (thin film, v_{max} / cm^{-1}) 1653, 1494, 1479, 1232, 1168, 1158, 750, 724.

MP ($CHCl_3$/pentane, 10%) 103–105 °C.

2dq 9-(5-(2-Methoxyphenyl)-4-methyl-1-tosylindolin-7-yl)-9H-carbazole

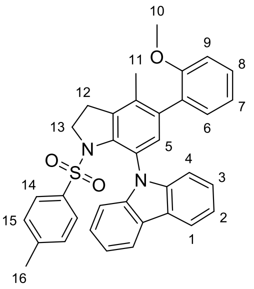

1d (42.7 mg, 0.10 mmol, 1.0 equiv.) was used in General Procedure **1** with 2-ethynylanisole (39.6 mg, 0.30 mmol, 3.0 equiv.) for 16 h with purification by flash column chromatography (SiO_2, EtOAc in pentane, 20%) then recrystallised ($CHCl_3$/pentane) to give the title compound (56.0 mg, 0.10 mmol, >99%) as a yellow solid.

R_f (EtOAc in pentane, 20%) 0.27.

^{1}H NMR (500 MHz, $CDCl_3$) δ_H 8.00 (2H, dt, J = 7.8, 1.0 Hz, H1), 7.38–7.30 (5H, m, ArH), 7.25–7.20 (3H, m, H2 and ArH), 7.17 (1H, dd, J = 7.4, 1.8 Hz, ArH), 6.99 (1H, td, J = 7.5, 1.1 Hz, ArH), 6.96 (1H, dd, J = 8.4, 1.0 Hz, H9), 6.85–6.77 (2H, m, H14), 6.73 (2H, d, J = 8.1 Hz, H15), 4.25 (2H, t, J = 7.3 Hz, H13), 3.81 (3H, s, H10), 2.99 (2H, t, J = 7.3 Hz, H12), 2.25 (3H, s, H16), 2.10 (3H, s, H11).

^{13}C NMR (126 MHz, $CDCl_3$) δ_C 156.8, 143.0, 140.4, 138.4, 138.2, 137.6, 135.7, 133.1, 131.3, 129.5, 129.2, 129.1, 128.7, 126.54, 126.47, 125.6, 123.7, 120.6, 120.0, 119.6, 110.9, 55.5, 52.5, 30.3, 21.6, 17.0. *One aromatic carbon signal not found.*

HRMS (ES^+) calc. for $C_{35}H_{31}N_2O_3S$ ($[M+H]^+$) 559.2050, found 559.2051.

IR (thin film, v_{max} / cm^{-1}) 1383, 1250, 1155, 1073, 954.

MP ($CHCl_3$/pentane, 10%) 102–104 °C.

2ep 4-(7-(9*H*-Carbazol-9-yl)-4-methyl-1-tosylindolin-5-yl)benzonitrile

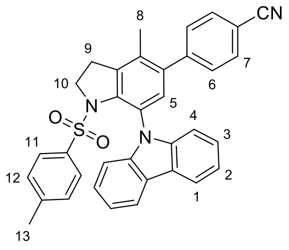

1d (42.7 mg, 0.10 mmol, 1.0 equiv.) was used in General Procedure **1** with 4-ethynylbenzonitrile (38.1 mg, 0.30 mmol, 3.0 equiv.) for 16 h with purification by flash column chromatography (SiO_2, EtOAc in pentane, 40%) then recrystallised ($CHCl_3$/pentane) to give the title compound (42.0 mg, 0.076 mmol, 76%) as an amber solid.

R$_f$ (EtOAc in pentane, 20%) 0.42.

^{1}H NMR (500 MHz, $CDCl_3$) δ_H H 7.99–7.94 (2H, m, H1), 7.69 (2H, dt, *J* = 8.4, 1.7 Hz, H7), 7.44 (2H, dt, *J* = 8.4, 1.7 Hz, H6), 7.34 (2H, td, *J* = 7.5, 1.2 Hz, H3), 7.24–7.20 (4H, m, H2 and H4), 7.18 (1H, s, H5), 6.79–6.70 (2H, m, H11), 6.66 (2H, d, *J* = 8.1 Hz, H12), 4.32 (2H, t, *J* = 7.3 Hz, H10), 3.09 (2H, t, *J* = 7.3 Hz, H9), 2.23 (3H, s, H8), 2.23 (3H, s, H13).

^{13}C NMR (126 MHz, $CDCl_3$) δ_C 145.2, 143.1, 140.3, 139.5, 139.2, 135.7, 132.3, 131.5, 130.2, 128.9, 128.7, 127.2, 126.2, 125.8, 123.9, 120.1, 120.0, 118.9, 111.3, 110.6, 52.5, 30.5, 21.6, 17.3. *One aromatic carbon signal not found.*

HRMS (ES$^+$) calc. for $C_{35}H_{28}N_3O_2S$ ($[M+H]^+$) 554.1897, found 554.1896.

IR (thin film, ν_{max} / cm^{-1}) 1383, 1251, 1156, 1073, 955.

MP ($CHCl_3$/pentane, 10%) 144–146 °C.

2da (7-(9*H*-Carbazol-9-yl)-4-methyl-1-tosylindoline-5,6-diyl)dimethanol

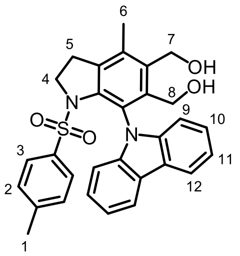

1d (42.6 mg, 0.10 mmol, 1.0 equiv.) was used in General Procedure **1** with 2-butyne-1,4-diol (25.8 mg, 0.30 mmol, 3.0 equiv.) for 16 h with purification by flash column chromatography (SiO_2, EtOAc in pentane, 10 to 50%) to give the title compound (29.9 mg, 0.058 mmol, 58%) as a brown foam.

R$_f$ (EtOAc in pentane, 50%) 0.25.

^{1}H NMR (400 MHz, $CDCl_3$) δ_H 7.83 (2H, d, *J* = 7.7 Hz, H3), 7.27–7.23 (2H, m, ArH), 7.17–7.09 (2H, m, H2), 6.94 (2H, d, *J* = 8.1 Hz, ArH), 6.53 (4H, s, ArH), 4.69 (2H, d, *J* = 4.1 Hz, CH_2), 4.17 (2H, t, *J* = 7.3 Hz, H4), 3.97 (2H, d, *J* = 2.8 Hz, CH_2), 3.05 (1H, br s, OH), 2.97 (2H, t, *J* = 7.3 Hz, H5), 2.38 (3H, s, H6), 2.15 (3H, s, H1), 1.86 (1H, br s, OH).

Note: the abnormally low chemical shift of the tosyl-CH_3 protons (2.15 ppm) is generally seen for all carbazole-substituted 7-aminoindolines prepared in this work.

^{13}C NMR (101 MHz, $CDCl_3$) δ_C 142.6, 141.5, 140.8, 139.2, 138.8, 138.5, 136.0, 135.7, 128.6, 125.9, 125.5, 125.3, 123.5, 120.1, 120.0, 110.6, 59.5, 58.8, 52.9, 30.6, 21.6, 16.3.

HRMS (ES$^+$) calc. for $C_{30}H_{28}O_4N_2SNa$ ([M+Na]$^+$) 535.1662, found 535.1662.

IR (thin film, ν_{max} / cm^{-1}) 3353, 2947, 2907, 1770, 1759, 1373, 1315, 1246.

2dw 9-(4-Methyl-1-tosyl-6-(triethylsilyl)indolin-7-yl)-9*H*-carbazole *(isolated as > 20:1 r.r., crude > 20:1 r.r.)*

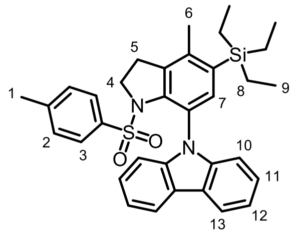

1d (42.6 mg, 0.10 mmol, 1.0 equiv.) was used in General Procedure **1** with triethylsilylacetylene (42.1 mg, 0.30 mmol, 3.0 equiv.) for 16 h with purification by flash column chromatography (SiO_2, EtOAc in pentane, 10 to 50%) to give the title compound (8.0 mg, 0.014 mmol, 14%) as a brown foam.

R_f (EtOAc in pentane, 10%) 0.34.

^{1}H NMR (400 MHz, $CDCl_3$) 8.02 (2H, d, *J* = 7.6 Hz, ArH), 7.38 (1H, s, H7), 7.35 (2H, t, *J* = 7.7 Hz, ArH), 7.24–7.21 (4H, m, ArH), 6.79 (2H, d, *J* = 8.2 Hz, H3), 6.72 (2H, d, *J* = 8.2 Hz, H2), 4.19 (2H, t, *J* = 7.4 Hz, H4), 2.87 (2H, t, *J* = 7.4 Hz, H5), 2.36 (3H, s, H6), 2.25 (3H, s, H1), 0.94 (9H, t, *J* = 7.7 Hz, H9), 0.85–0.80 (6H, m, H8).

^{13}C NMR (126 MHz, $CDCl_3$) δ_C 143.1, 140.5, 139.6, 139.4, 138.2, 135.7, 135.6, 135.1, 128.8, 126.5, 126.3, 125.7, 123.7, 120.1, 119.6, 110.6, 52.1, 30.0, 21.6, 19.6, 7.7, 4.2

HRMS (ES$^+$) calc. for $C_{34}H_{39}N_2O_2SSi$ ([M+H]$^+$) 567.2496, found 567.2510

IR (thin film, ν_{max} / cm^{-1}) 2989, 2947, 1597, 1464, 1453, 1365, 1336, 1233, 1168, 1003.

7aa *N,N'*-(2,3-Dihydro-1*H*-indene-5,6-diyl)bis(*N*-butyl-4-methylbenzenesulfonamide)

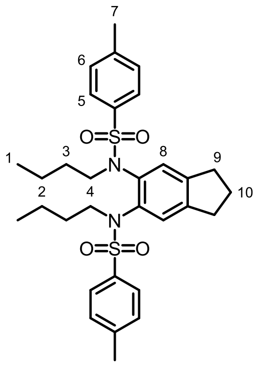

6a (47.6 mg, 0.10 mmol, 1.0 equiv.) and 1,6-heptadiyne (46.0 mg, 0.50 mmol, 5.0 equiv.) were used in General Procedure **2** with purification by flash column chromatography (SiO_2, EtOAc in pentane, 5 to 10%) to give the title compound (35.0 mg, 0.062 mmol, 62%) as a cream solid.

R_f (EtOAc in pentane, 20%) 0.30.

^{1}H NMR (500 MHz, $CDCl_3$) δ_H 7.81 (4H, d, *J* = 8.1 Hz, H5), 7.34 (4H, d, *J* = 8.1 Hz, H6), 6.90 (2H, s, H8), 3.75–3.64 (4H, m, H4), 2.86 (4H, dt, *J* = 8.1, 4.0 Hz, H9), 2.46 (6H, s, H7), 2.11 (2H, p, *J* = 7.5 Hz, H10), 1.52–1.32 (4H, m, H3), 1.15 (4H, app. h, *J* = 7.4 Hz, H2), 0.80 (6H, t, *J* = 7.4 Hz, H1).

^{13}C NMR (126 MHz, $CDCl_3$) δ_C 145.1, 143.5, 138.3, 137.1, 129.7, 128.2, 126.7, 51.5, 32.8, 29.7, 25.6, 21.7, 20.2, 13.8.

HRMS (ES^+) calc. for $C_{31}H_{41}O_4N_2S_2$ ($[M+H]^+$) 569.2502, found 569.2501.

IR (thin film, ν_{max} / cm^{-1}) 2974, 1350, 1331, 1305, 1162, 1095.

MP (Et_2O in pentane, 10%) 168–170 °C

7ab *N,N'*-(2,3-Dihydro-1*H*-indene-5,6-diyl)bis(*N*-benzyl-4-methylbenzenesulfonamide)

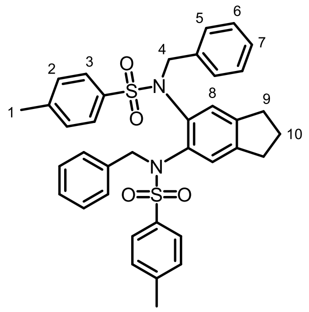

6b (54.4 mg, 0.10 mmol, 1.0 equiv.) and 1,6-heptadiyne (46.0 mg, 0.50 mmol, 5.0 equiv.) were used in General Procedure **2** with purification by flash column chromatography (SiO_2, EtOAc in pentane, 5 to 20%) to give the title compound (39.8 mg, 0.063 mmol, 63%) as a cream solid.

Note: this compound exhibited restricted rotation about the C(aryl)-N axis leading to broadening in the ^{1}H NMR spectrum; this was confirmed by a variable temperature (25–85 °C) ^{1}H NMR experiment.

Scaled up procedure (1.0 mmol scale)

6b (0.544 g, 1.0 mmol, 1.0 equiv.) and 1,6-heptadiyne (0.460 g, 5.0 mmol, 5.0 equiv.) were used in General Procedure **2** with purification by flash column chromatography (SiO_2, EtOAc in pentane, 10%) to give the title compound (0.372 g, 0.58 mmol, 58%) as a cream solid.

R_f (EtOAc in pentane, 20%) 0.40.

^{1}H NMR (400 MHz, $CDCl_3$) δ_H 7.73 (4H, d, *J* = 8.3 Hz, H3), 7.28–7.26 (4H, m, H2), 7.22–7.11 (6H, m, ArH), 7.09–7.04 (4H, m, ArH), 6.54 (2H, s, H8), 5.04–4.99 (4H, m, H4), 2.73–2.67 (4H, m, H9), 2.45 (6H, s, H1), 2.03 (2H, p, *J* = 7.5 Hz, H10).

^{13}C NMR (101 MHz, $CDCl_3$) δ_C 144.9, 143.5, 138.5, 136.8, 136.0, 130.4, 129.6, 128.4, 128.1, 128.0, 127.7, 55.3, 32.7, 25.4, 21.7.

HRMS (ES^+) calc. for $C_{37}H_{37}O_4N_2S_2$ ($[M+H]^+$) 637.2189, found 637.2182.

IR (thin film, ν_{max} / cm^{-1}) 1493, 1342, 1157, 1090, 1041.

MP ($CHCl_3$) 177–179 °C.

7ba Dimethyl 5,6-bis((*N*-butyl-4-methylphenyl)sulfonamido)-1,3-dihydro-2*H*-indene-2,2-dicarboxylate

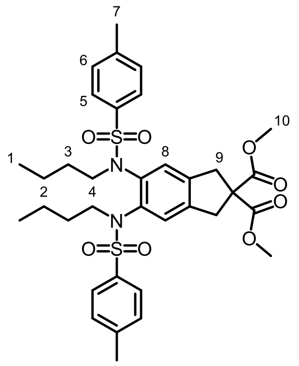

6a (54.4 mg, 0.10 mmol, 1.0 equiv.) and dimethyl 2,2-di(prop-2-yn-1-yl)malonate (0.104 g, 0.50 mmol, 5.0 equiv.) were used in General Procedure **2** with purification by flash column chromatography (SiO_2, EtOAc in pentane, 5 to 30%) to give the title compound (54.8 mg, 0.080 mmol, 80%) as a white solid.

R_f (EtOAc in pentane, 20%) 0.23.

^{1}H NMR (400 MHz, $CDCl_3$) δ_H 7.78 (4H, d, *J* = 8.4 Hz, H5), 7.36–7.31 (4H, m, H6), 6.85 (2H, s, H8), 3.78 (6H, s, H10), 3.71–3.62 (4H, m, H4), 3.58–3.55 (4H, m, H9), 2.46 (6H, s, H7), 1.48–1.30 (4H, m, H3), 1.14 (4H, app. p, *J* = 7.4 Hz, H2), 0.80 (6H, t, *J* = 7.3 Hz, H1).

^{13}**C NMR** (101 MHz, $CDCl_3$) δ_C 171.8, 143.7, 140.7, 138.3, 137.9, 129.7, 128.2, 126.5, 60.5, 53.3, 51.6, 40.4, 29.7, 21.7, 20.1, 13.8.

HRMS (ES^+) calc. for $C_{35}H_{45}O_8N_2S_2$ ($[M+H]^+$) 685.2612, found 685.2596.

IR (thin film, ν_{max} / cm^{-1}) 2959, 1736, 1494, 1435, 1345, 1274, 1247, 1162, 1089.

MP ($CHCl_3$) 135–137 °C.

7ca *N,N'*-(2-Tosylisoindoline-5,6-diyl)bis(*N*-butyl-4-methylbenzenesulfonamide)

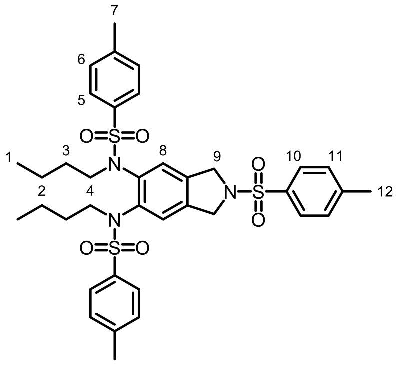

6a (54.4 mg, 0.10 mmol, 1.0 equiv.) and 4-methyl-*N*,*N*-di(prop-2-yn-1-yl)benzenesulfonamide (0.124 g, 0.50 mmol, 5.0 equiv.) were used in General Procedure **2** with purification by flash column chromatography (SiO_2, CH_2Cl_2) to give the title compound (66.2 mg, 0.092 mmol, 92%) as a sticky yellow oil.

R_f (CH_2Cl_2) 0.39.

^{1}**H NMR** (400 MHz, $CDCl_3$) δ_H 7.76–7.74 (6H, m, H5 + H10), 7.35–7.33 (6H, m, H6 + H11), 6.85 (2H, s, H8), 4.54 (4H, s, H9), 3.66–3.65 (4H, m, H4), 2.47 (6H, s, H7), 2.43 (3H, s, H12), 1.33–1.32 (4H, s, H3), 1.13 (4H, app. h, *J* = 7.3 Hz, H2), 0.78 (6H, t, *J* = 7.3 Hz, H1).

^{13}**C NMR** (101 MHz, $CDCl_3$) δ_C 144.3, 144.0, 139.1, 137.7, 136.9, 133.5, 130.1, 129.9, 128.1, 127.7, 125.2, 53.5, 51.4, 29.7, 21.72, 21.68, 20.1, 13.8.

HRMS (ES^+) calc. for $C_{37}H_{46}O_6N_3S_3$ ($[M+H]^+$) 724.2543, found 724.2537.

IR (thin film, ν_{max} / cm^{-1}) 2959, 2930, 1598, 1494, 1345, 1163, 1090, 1037.

7ea ***N,N'*-(2,2-Dioxido-1,3-dihydrobenzo[c]thiophene-5,6-diyl)bis(*N*-butyl-4-methylbenzenesulfonamide)**

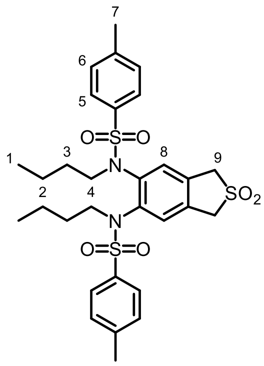

6a (54.4 mg, 0.10 mmol, 1.0 equiv.) and 3-(prop-2-yn-1-ylsulfonyl)prop-1-yne (71.0 mg, 0.50 mmol, 5.0 equiv.) were used in General Procedure **2** using 1,4-dioxane as the solvent at 90 °C, with purification by flash column chromatography (SiO_2, EtOAc in pentane, 10 to 40%) to give the title compound (16.0 mg, 0.026 mmol, 26%) as a viscous brown oil.

R$_f$ (EtOAc in pentane, 30%) 0.26.

^{1}H NMR (400 MHz, $CDCl_3$) δ_H 7.78 (4H, d, *J* = 8.0 Hz, H5), 7.36 (4H, d, *J* = 8.0 Hz, H6), 7.05 (2H, s, H8), 4.33 (4H, s, H9), 3.70–3.61 (4H, m, H4), 2.48 (6H, s, H7), 1.46–1.28 (4H, m, H3), 1.20–1.11 (4H, m, H2), 0.81 (6H, t, *J* = 7.4 Hz, H1).

^{13}C NMR (101 MHz, $CDCl_3$) δ_C 144.2, 140.1, 131.8, 130.0 (2 C), 128.7, 128.1, 56.9, 51.4, 29.8, 21.8, 20.1, 13.8.

HRMS (ES$^+$) calc. for $C_{30}H_{39}O_6N_2S_3$ ([M+H]$^+$) 619.1965, found 619.1963.

IR (thin film, ν_{max} / cm^{-1}) 2962, 2932, 1493, 1344, 1321, 1163, 1089, 1037.

7da ***N,N'*-(5,6,7,8-Tetrahydronaphthalene-2,3-diyl)bis(*N*-butyl-4-methylbenzenesulfonamide)**

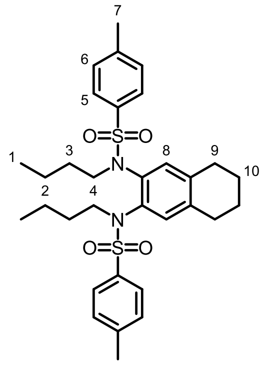

6a (54.4 mg, 0.10 mmol, 1.0 equiv.) and 1,7-octadiyne (53.1 mg, 0.50 mmol, 5.0 equiv.) were used in General Procedure **2** with purification by flash column chromatography (SiO_2, CH_2Cl_2) and recrystallisation (Et_2O in pentane, 10%) to give the title compound (10.0 mg, 0.017 mmol, 17%) as a white solid.

R$_f$ (EtOAc in pentane, 10%) 0.26.

^{1}H NMR (400 MHz, CDCl$_3$) δ$_H$ 7.79 (4H, d, *J* = 8.3 Hz, H5), 7.37–7.30 (4H, m, H6), 6.70 (2H, s, H8), 3.75–3.59 (4H, m, H4), 2.66–2.62 (4H, m, H9), 2.46 (6H, s, H7), 1.80–1.75 (4H, m, H10), 1.52 –1.31 (4H, m, H3), 1.21–1.12 (4H, m, H2), 0.81 (6H, t, *J* = 7.3 Hz, H1).

^{13}C NMR (101 MHz, CDCl$_3$) δ$_C$ 143.5, 138.2, 137.9, 136.2, 131.3, 129.6, 128.2, 51.5, 29.8, 29.0, 22.7, 21.7, 20.2, 13.8.

HRMS (ES$^+$) calc. for $C_{32}H_{43}O_4N_2S_2$ ([M+H]$^+$) 583.2659, found 583.2649.

IR (solid, v_{max} / cm^{-1}) 2958, 2934, 1498, 1343, 1153, 1089, 1043.

MP (Et_2O in pentane, 10%) 189–191 °C.

7ac *N*-(6-(9*H*-Carbazol-9-yl)-2,3-dihydro-1*H*-inden-5-yl)-*N*-butyl-4-methylbenzenesulfonamide

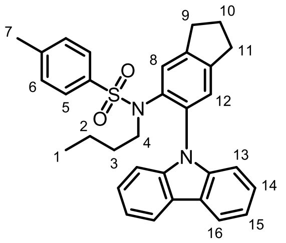

6c (41.6 mg, 0.10 mmol, 1.0 equiv.) and 1,6-heptadiyne (46.0 mg, 0.50 mmol, 5 equiv.) were used in General Procedure **2** with purification by flash column chromatography (SiO$_2$, EtOAc in pentane, 20%) followed by trituration (Et_2O in pentane, 10%) to give the title compound (30.0 mg, 0.58 mmol, 58%) as a white solid.

6c (1.00 g, 2.40 mmol, 1.0 equiv.) and 1,6-heptadiyne (1.37 mL, 12.0 mmol, 5 equiv.) were used in General Procedure **2** (*modification: dissolve **6c** and **Rh-1** in 10 mL PhMe and add 1,6-heptadiyne in 20 mL PhMe*) with purification by flash column chromatography (SiO$_2$, Et_2O in pentane, 10%) followed by trituration (Et_2O in pentane, 10%) to give the title compound (0.650 g, 1.28 mmol, 53%) as a white solid.

R$_f$ (Et_2O in pentane, 10%) 0.16.

^{1}H NMR (400 MHz, CDCl$_3$) δ$_H$ 8.13 (2H, d, *J* = 7.7 Hz, H5), 7.59 (1H, s, H8), 7.38–7.33 (2H, m, ArH), 7.29–7.23 (3H, m, ArH), 7.22 (1H, s, H12), 7.19–7.17 (3H, m, ArH), 6.94–6.92 (2H, m, ArH), 3.09 (2H, t, *J* = 7.5 Hz, H9), 2.98 (2H, t, *J* = 7.5 Hz, H11), 2.80 (2H, t, *J* = 8.4 Hz, H4), 2.29 (3H, s, H7), 2.22 (2H, q, *J* = 7.5 Hz, H10), 1.24 (2H, qd, *J* = 8.4, 6.7 Hz, H3), 0.83–0.78 (2H, m, H2), 0.59 (3H, t, *J* = 7.3 Hz, H1).

^{13}C NMR (101 MHz, CDCl$_3$) δ$_C$ 146.3, 146.1, 143.4, 142.3, 136.6, 136.5, 133.8, 129.3, 128.1, 127.6, 127.1, 125.9, 123.3, 120.2, 119.8 (2 C), 51.0, 33.1, 32.8, 29.6, 25.9, 21.5, 19.8, 13.6.

HRMS (ES$^+$) calc. for $C_{32}H_{33}N_2O_2S$ [M+H]$^+$ 509.2263, found 509.2257.

IR (thin film, v_{max} / cm^{-1}) 2981, 2888, 1382, 1157, 1088.

M.P (pentane) 169–171 °C.

7ad *N*-(6-(9*H*-Carbazol-9-yl)-2,3-dihydro-1H-inden-5-yl)-*N*-benzyl-4-methylbenzenesulfonamide

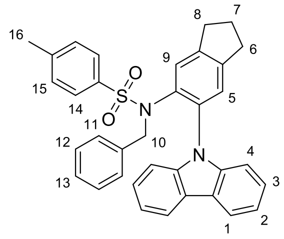

6d (45.0 mg, 1.0 equiv., 0.10 mmol) and 1,6-heptadiyne (1.37 mL, 12.0 mmol, 5 equiv.) were used in General Procedure **2** with purification by flash column chromatography (SiO_2, Et_2O in pentane, 10%) followed by trituration (EtOAc in pentane, 20%) to give the title compound (31.0 mg, 0.057 mmol, 57%) as a pale yellow solid.

R$_f$ (Et_2O in pentane, 10%) 0.24.

^{1}H NMR (500 MHz, $CDCl_3$) δ_H 8.16 (2H, dt, *J* = 7.7, 1.0 Hz, H1), 7.39 (1H, s, H9), 7.33 (2H, t, *J* = 7.6 Hz, H3), 7.29–7.24 (2H, m, H2), 7.19 (1H, s, H5), 7.14 (2H, br. s, H4), 7.07 (1H, tt, *J* = 7.5, 1.3 Hz, H11), 7.02–6.94 (4H, m, H14 + ArH), 6.81 (2H, d, *J* = 8.0 Hz, H15), 6.71 (2H, d, *J* = 7.4 Hz, H10), 4.20 (2H, s, H10), 2.98 (2H, t, *J* = 7.6 Hz, H8), 2.94 (2H, t, *J* = 7.6 Hz, H6), 2.25 (3H, s, H16), 2.18 (2H, p, *J* = 7.6 Hz, H7).

^{13}C NMR (126 MHz, $CDCl_3$) δ_C 146.2, 146.1, 143.1, 142.3, 136.9, 136.7, 134.8, 133.8, 129.3, 129.0, 128.0, 127.9, 127.8, 127.5, 127.0, 125.9, 123.3, 120.0, 119.7, 110.9, 77.3, 77.0, 76.8, 55.4, 32.8, 32.6, 25.7, 21.4.

HRMS (ES$^+$) calc. for $C_{35}H_{31}N_2O_2S$ ($[M+H]^+$) 543.2101, found 543.2100.

IR (thin film, v_{max} / cm^{-1}) 1597, 1479, 1334, 1184, 1100, 751.

M.P (pentane) 202–204 °C.

7ae *N*-(6-(9H-Carbazol-9-yl)-2,3-dihydro-1H-inden-5-yl)-*N*-isopropyl-4-methylbenzenesulfonamide

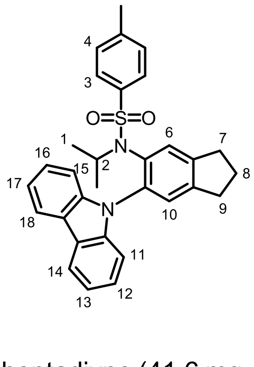

6e (36.2 mg, 0.09 mmol, 1.0 equiv.) and 1,6-heptadiyne (41.6 mg, 0.45 mmol, 5.0 equiv.) were used in General

Procedure **2**, with purification by flash column chromatography (SiO_2, PhMe) followed by recystallisation ($CHCl_3$/pentane) to give the title compound (7.6 mg, 0.015 mmol, 17%) as a white solid.

R$_f$ (PhMe, 20%) 0.24.

^{1}H NMR (400 MHz, $CDCl_3$) δ_H 8.13 (2H, t, *J* = 7.1 Hz, ArH), 7.66 (2H, d, *J* = 6.5 Hz, ArH), 7.39 (1H, t, *J* = 7.7 Hz, ArH), 7.35 – 7.32 (1H, m, ArH), 7.31 – 7.29 (3H, m, H3 + ArH), 7.26 – 7.21 (2H + $CHCl_3$, m, ArH), 7.11 (1H, d, *J* = 8.2 Hz, ArH), 6.96 (2H, d, *J* = 8.0 Hz, ArH, H4), 3.58 (1H, p, *J* = 6.7 Hz, H2), 3.09 (1H, dt, *J* = 16.0, 7.5 Hz), 2.97 (1H, td, *J* = 7.3, 4.4 Hz), 2.29 (3H, s, H5), 2.26 – 2.19 (2H, m), 0.89 (3H, d, *J* = 6.7 Hz, H1), 0.29 (3H, d, J=6.7 Hz, H1').

^{13}C NMR (101 MHz, $CDCl_3$) δ_C 146.6, 145.8, 143.5, 143.0, 142.5, 136.9, 135.6, 133.9, 129.5, 129.3, 128.7, 127.2, 126.2, 125.5, 123.7, 123.1, 120.4, 120.0, 119.8, 119.6, 112.7, 110.6, 53.4, 33.0, 32.8, 25.9, 21.8, 21.5, 20.7 *Note diastereotopic character of carbazole ring leads to additional carbon signals*.

HRMS (ES$^+$) calc. for $C_{31}H_{31}O_2N_2S$ ([M+H]$^+$) 495.2101, found 495.2103.

IR (thin film, ν_{max} / cm^{-1}) 1626, 1597, 1497, 1478, 1453, 1389, 1338, 1317, 1231, 1161, 1132, 1086.

MP ($CHCl_3$/pentane) 130–132 °C.

7af *N*-(6-(9*H*-carbazol-9-yl)-2,3-dihydro-1H-inden-5-yl)-*N*-cyclohexyl-4-methylbenzenesulfonamide

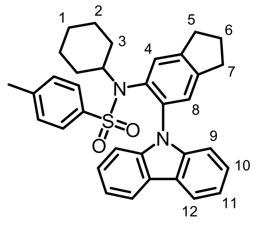

6f (44 mg, 0.1 mmol, 1.0 equiv.)and 1,6- hexadiyne (46 mg, 0.5 mmol, 5 equiv.) were used in General Procedure **2**, with purification by flash column chromatography (SiO_2, toluene) and recrystallisation ($CHCl_3$/pentane) to give the title compound (27.0 mg, 0.050 mmol, 50%) as a white solid.

R$_f$ (toluene) 0.19.

^{1}H NMR (500 MHz, $CDCl_3$) δ_H 8.13 (2H, d, *J* = 7.7 Hz, ArH), 7.65 (1H, s, H9), 7.60 (1H, d, *J* = 8.2 Hz, ArH), 7.43–7.32 (2H, m, ArH), 7.32–7.28 (3H, m, ArH), 7.28–7.19 (2H, m, H13 + ArH), 7.10 (1H, d, *J* = 8.2 Hz, ArH), 6.96 (2H, d, *J* = 8.0 Hz, TsH), 3.17–3.04 (3H, m, H5 + H10), 3.01–2.94 (2H, m, H12), 2.30 (3H, s, CH_3), 2.23 (2H, p, *J* = 7.5 Hz, H11), 1.54–1.44 (2H, m, CyH), 1.26 (1H, d, *J* = 8.0 Hz, CyH), 1.11 (1H, s, CyH), 1.02–0.92 (1H, m, CyH), 0.87–0.80 (1H, m, CyH), 0.78–0.54 (4H, m, CyH).

^{13}C NMR (126 MHz, $CDCl_3$) δ_C 146.4, 145.7, 143.4, 142.8, 142.5, 137.2, 135.2, 135.0, 129.29, 129.27, 128.6, 127.2, 126.1, 125.4, 123.6, 123.0, 120.4, 119.8, 119.6, 112.6, 110.6, 62.2, 33.0, 32.8, 31.8, 31.5, 26.2, 25.9, 25.1, 21.5. *Extra carbon peaks due to restricted rotation.*

HRMS (ES+) calc. for $C_{34}H_{35}N_2O_2S$ ([M+H]+) 535.2414, found 535.2414.

IR (thin film, v_{max} / cm^{-1}) 1479, 1452, 1317, 1231, 1160, 1090.

7bc Dimethyl 5-((*N*-butyl-4-methylphenyl)sulfonamido)-6-(9H-carbazol-9-yl)-1,3-dihydro-2H-indene-2,2-dicarboxylate

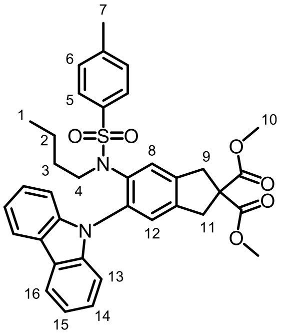

6c (41.7 mg, 0.10 mmol, 1.0 equiv.) and dimethyl 2,2-di(prop-2-yn-1-yl)malonate (0.104 g, 0.50 mmol, 5.0 equiv.) were used in General Procedure **2**, with purification by flash column chromatography (SiO_2, Et_2O in PhMe, 20%) then recrystallisation ($CHCl_3$/pentane) to give the title compound (26.9 mg, 0.043 mmol, 43%) as a pale yellow solid.

R$_f$ (EtOAc in pentane, 20%) 0.19.

^{1}H NMR (400 MHz, $CDCl_3$) δ_H 8.12 (1H, d, *J* = 7.6 Hz, H5), 7.57 (1H, s, ArH), 7.36 (2H, ddd, J=8.2, 7.1, 1.2 Hz, ArH), 7.28–7.24 (2H + $CHCl_3$, m, H6), 7.22 (1H, s, ArH), 7.20–7.16 (4H, m), 6.95–6.93 (2H, m), 3.82 (6H, s, H10), 3.80 (2H, s, CH_2), 3.68 (2H, s, CH_2), 2.80–2.76 (2H, m, H4), 2.29 (3H, s, H7), 1.19 (2H, p, *J* = 7.9 Hz, H3), 0.77 (2H, p, *J* = 7.3 Hz, H2), 0.58 (3H, t, *J* = 7.3 Hz, H1).

^{13}C NMR (101 MHz, $CDCl_3$) δ_C 171.9, 143.6, 142.1, 141.9, 141.7, 137.8, 136.3, 134.8, 129.4, 128.1, 127.5, 127.1, 126.0, 123.4, 120.2, 119.9, 110.8, 60.6, 53.4, 51.0, 40.7, 40.4, 29.6, 21.5, 19.8, 13.5.

HRMS (ES+) calc. for $C_{36}H_{37}O_6N_2S$ ([M+H]+) 625.2367, found 625.2365.

IR (thin film, v_{max} / cm^{-1}) 1734, 1598, 1500, 1478, 1453, 1435, 1382, 1317, 1277, 1250, 1233, 1201, 1162, 1088, 1072, 956, 915, 859, 816, 752.

MP ($CHCl_3$/pentane) 78–80 °C.

7cc ***N*-(6-(9*H*-Carbazol-9-yl)-2-tosylisoindolin-5-yl)-*N*-butyl-4-methylbenzenesulfonamide**

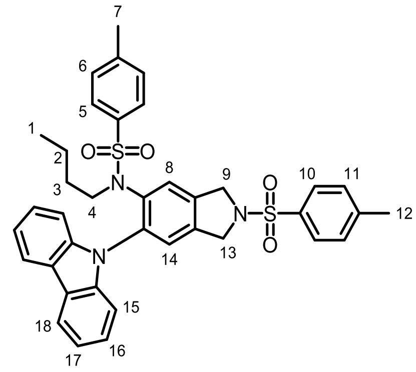

6c (41.7 mg, 0.10 mmol, 1.0 equiv.) and 4-methyl-*N*,*N*-di(prop-2-yn-1-yl)benzenesulfonamide (0.124 g, 0.50 mmol, 5.0 equiv.) were used in General Procedure **2**, with purification by flash column chromatography (SiO_2, EtOAc in pentane, 20%) then recrystallisation ($CHCl_3$/pentane) to give the title compound (33.2 mg, 50%) as a pale yellow solid.

R_f (EtOAc in pentane, 30%) 0.26;

^{1}H NMR (400 MHz, $CDCl_3$) δ_H 8.12 (2H, d, *J* = 7.7 Hz, H5), 7.82 (2H, d, *J* = 8.3 Hz, H10), 7.59 (1H, s, H8), 7.40 – 7.37 (2H, m, H11), 7.35 – 7.33 (1H, m), 7.29 – 7.25 (2H, m), 7.21 (1H, s, H14), 7.17 (1H, d, *J* = 8.4 Hz), 7.10 – 7.08 (2H, m), 6.96 – 6.94 (2H, m), 4.77 (2H, s, H9), 4.67 (2H, s, H13), 2.74 (2H, t, *J* = 8.1 Hz, H4), 2.47 (3H, s, H12), 2.29 (3H, s), 1.16 (2H, ddd, *J* = 15.8, 8.7, 6.5 Hz, H3), 0.76 (2H, h, *J* = 7.3 Hz, H2), 0.57 (3H, t, *J* = 7.3 Hz, H1).

^{13}C NMR (151 MHz, $CDCl_3$) δ_C 144.2, 143.9, 141.9, 138.7, 138.2, 137.8, 136.0, 135.7, 133.6, 130.2, 129.5, 128.1, 127.9, 126.19, 126.15, 125.9, 123.5, 120.4, 120.2, 110.5, 77.4, 77.2, 77.0, 53.7, 53.5, 50.8, 29.5, 21.7, 21.6, 19.7, 13.5.

HRMS (ES^+) calc. for $C_{38}H_{38}N_3O_4S_2$ ($[M+H]^+$) 664.2298, found 664.2295.

IR (thin film, ν_{max} / cm^{-1}) 1453, 1348, 1164, 751, 668.

MP ($CHCl_3$/pentane) 178–180 °C.

NMR spectra of novel compounds

Dibromoenamides

S1 9-(2,2-Dibromovinyl)-9H-carbazole

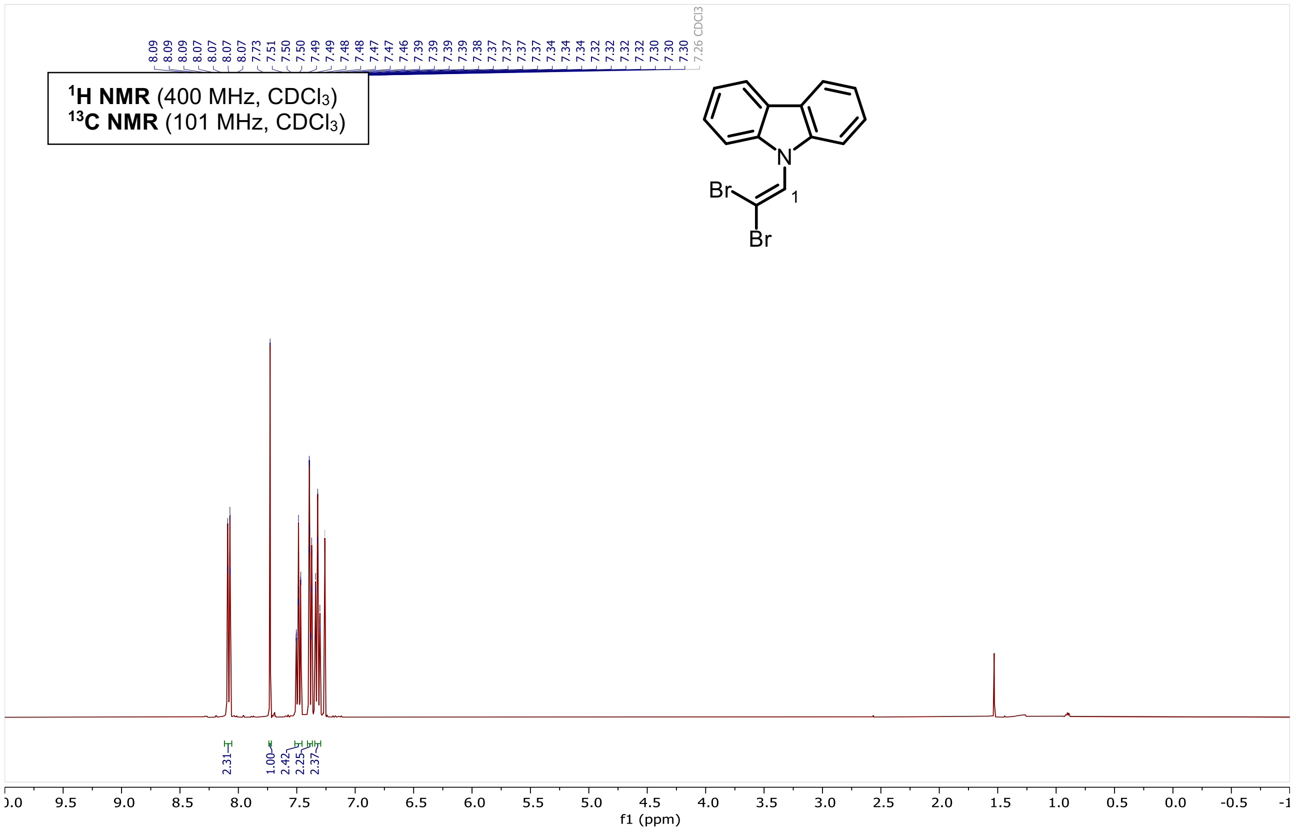

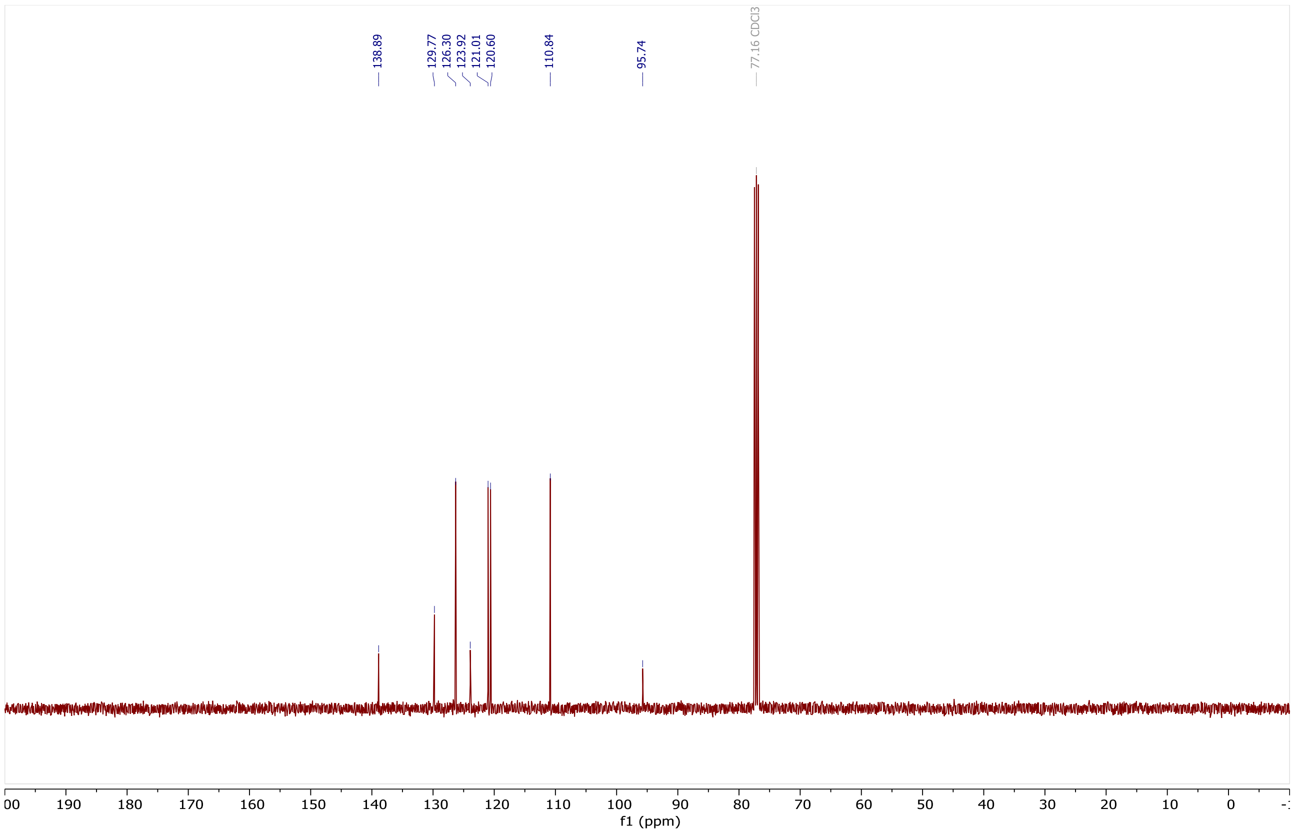

Yndiamides

1e *N*-((9H-Carbazol-9-yl)ethynyl)-4-methyl-*N*-(pent-3-yn-1-yl)benzenesulfonamide

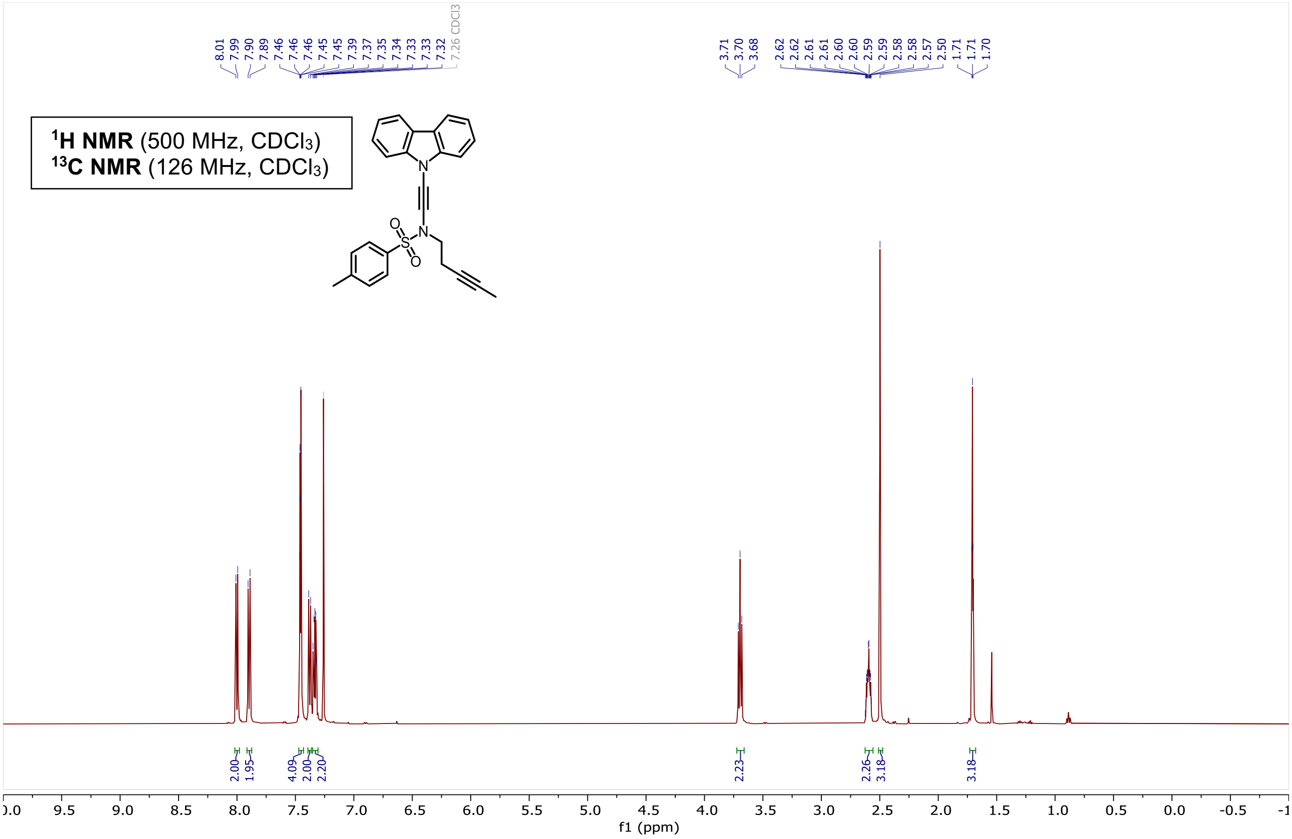

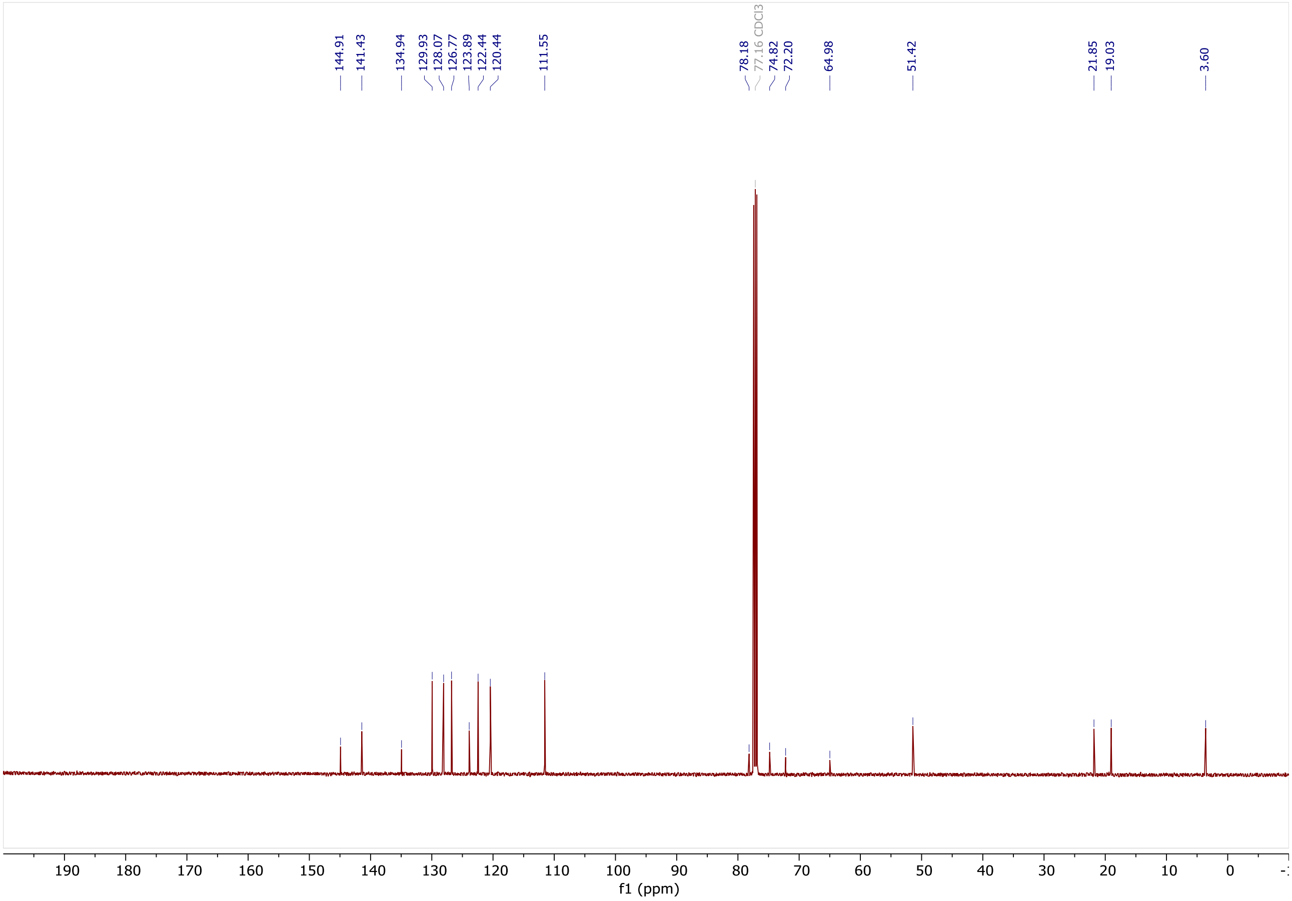

6c *N*-((9H-Carbazol-9-yl)ethynyl)-*N*-butyl-4-methylbenzenesulfonamide

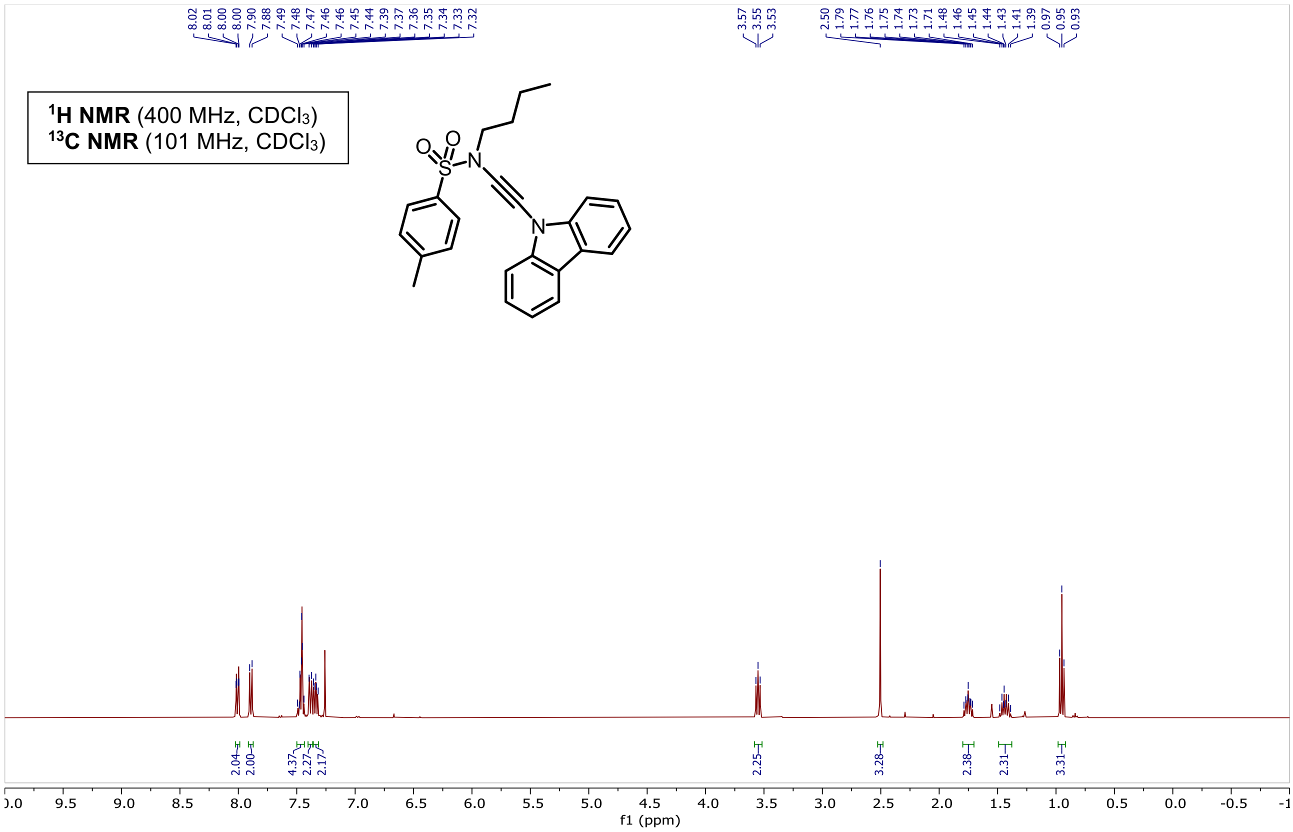

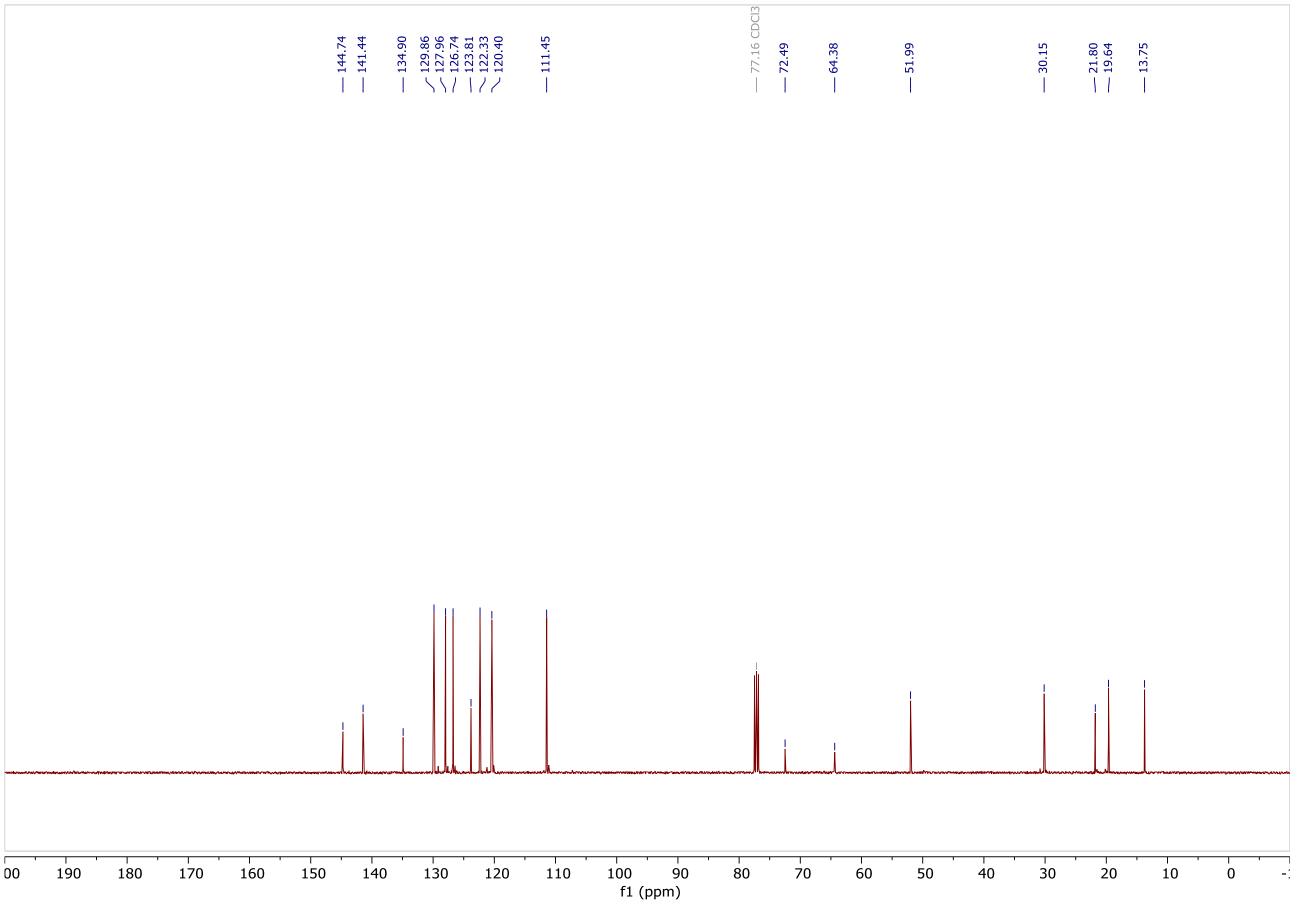

6d *N*-((9H-Carbazol-9-yl)ethynyl)-N-benzyl-4-methylbenzenesulfonamide

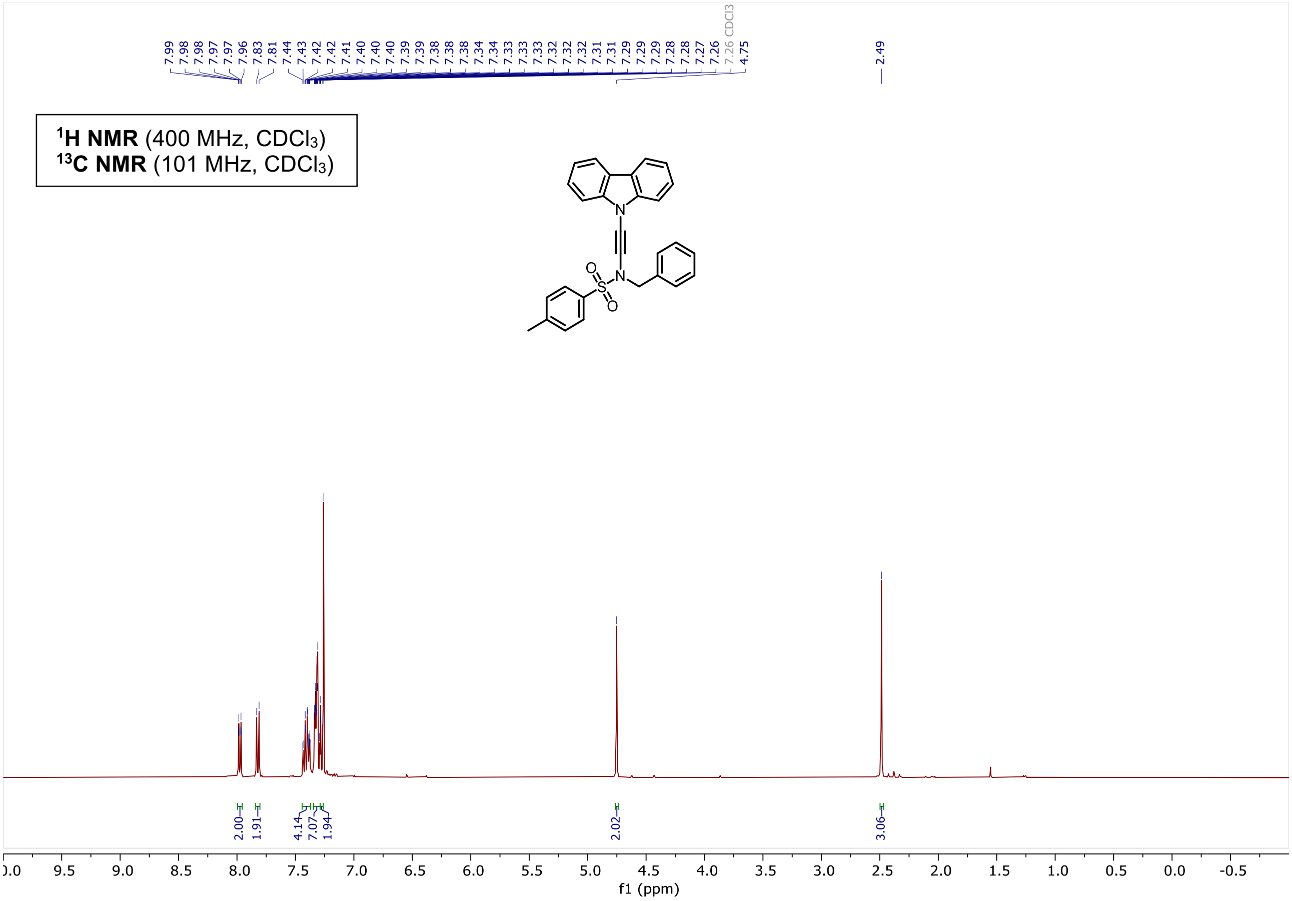

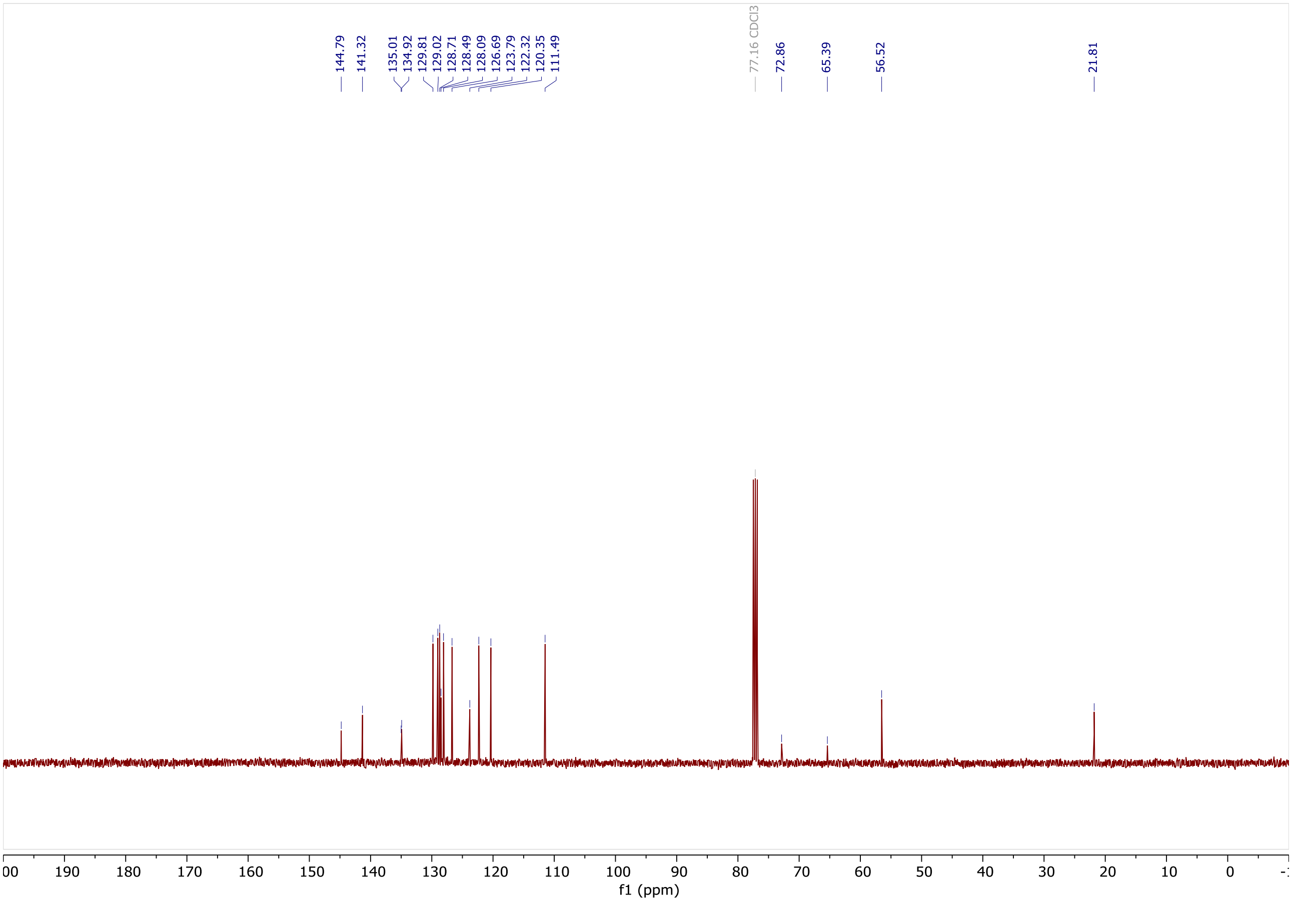

6e *N*-((9*H*-Carbazol-9-yl)ethynyl)-*N*-isopropyl-4-methylbenzenesulfonamide

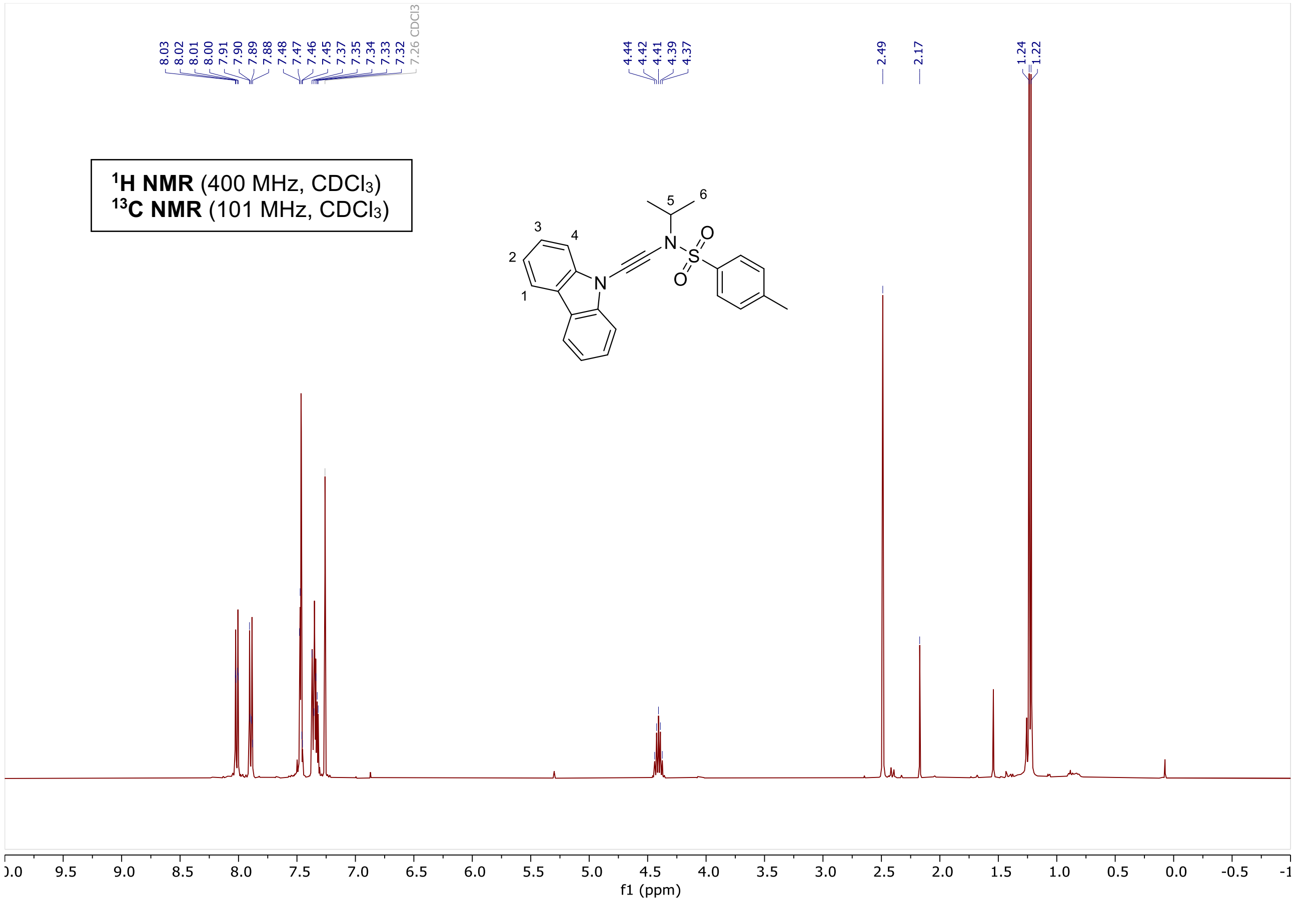

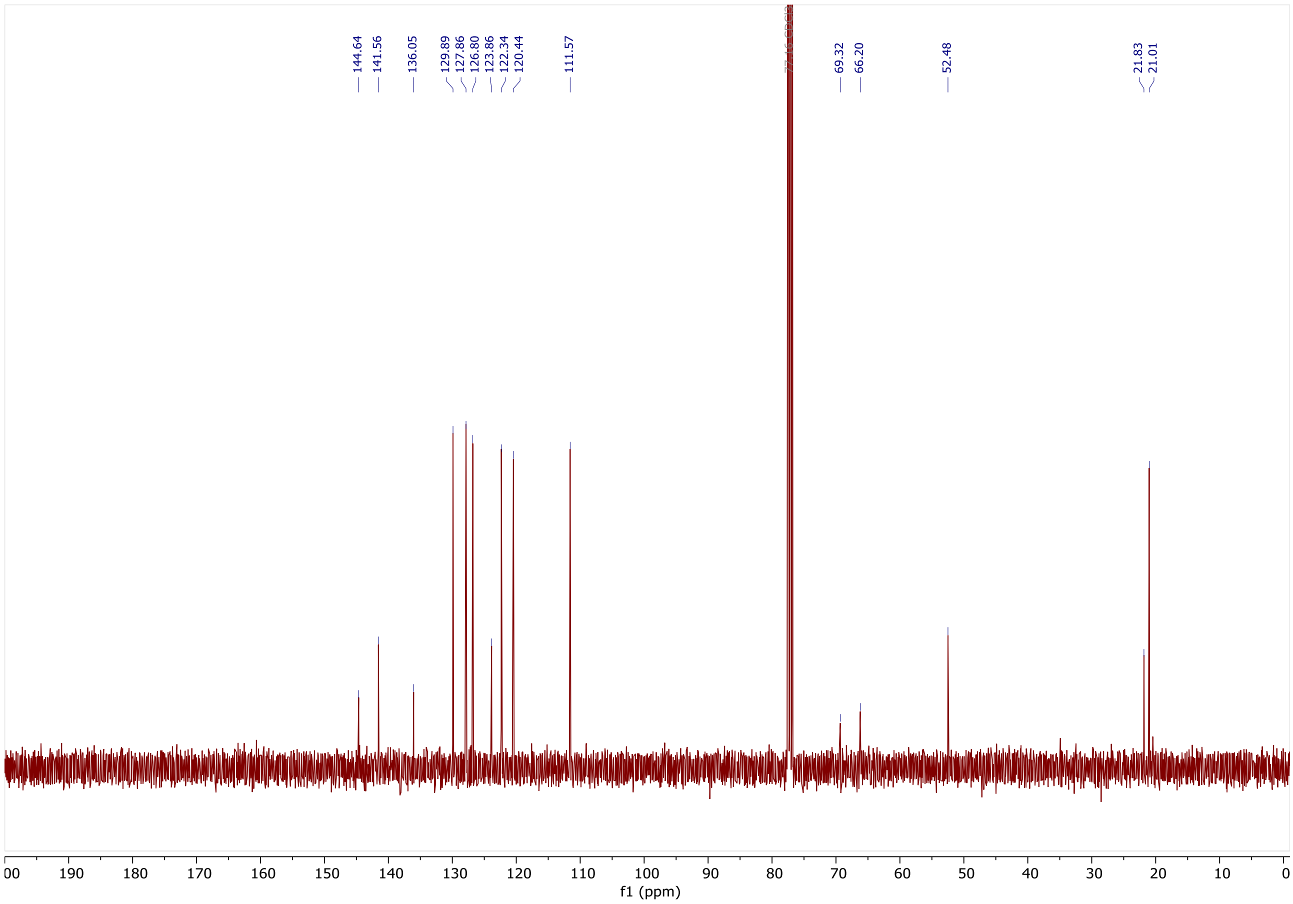

6f *N*-((9*H*-Carbazol-9-yl)ethynyl)-*N*-cyclohexyl-4-methylbenzenesulfonamide

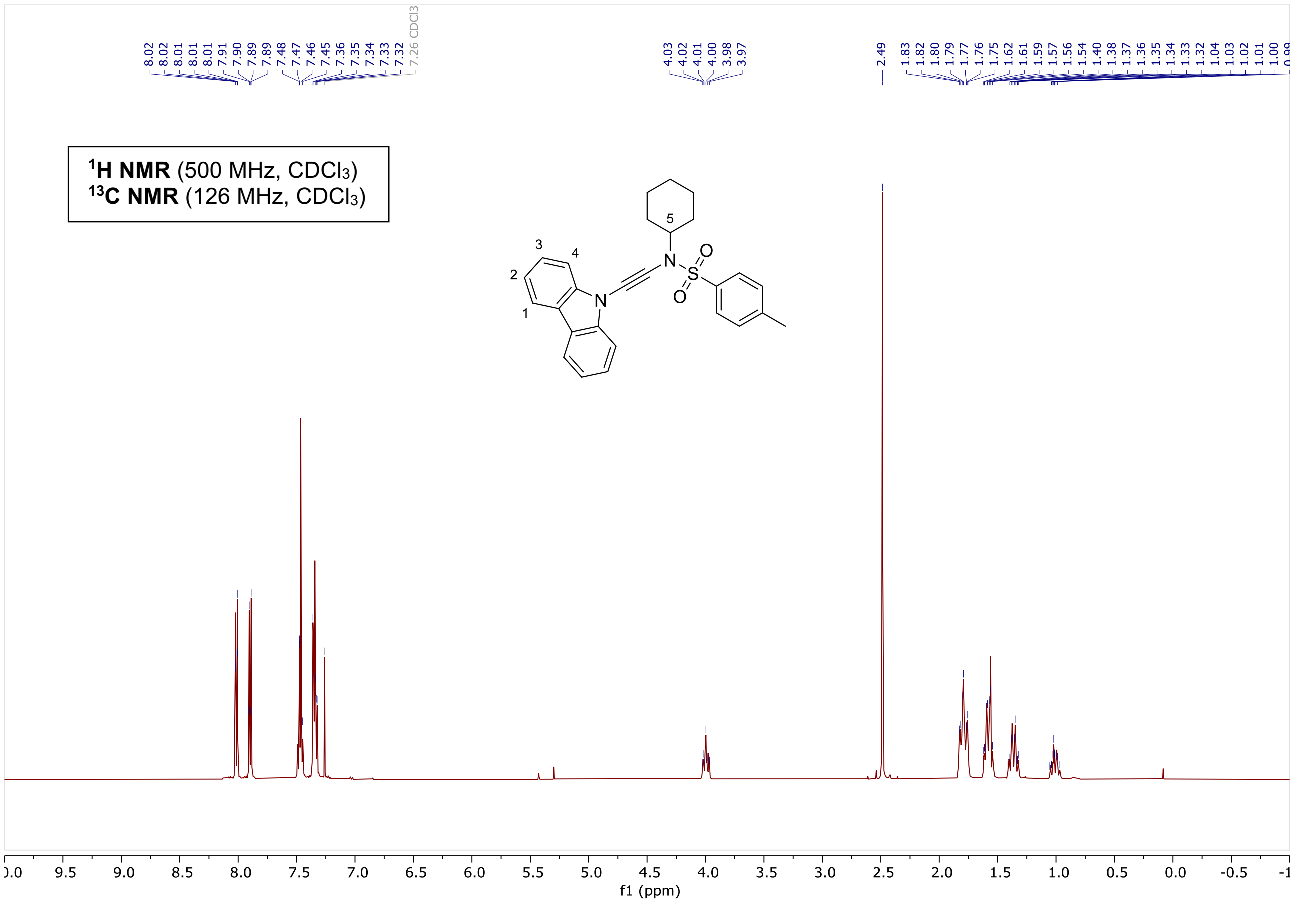

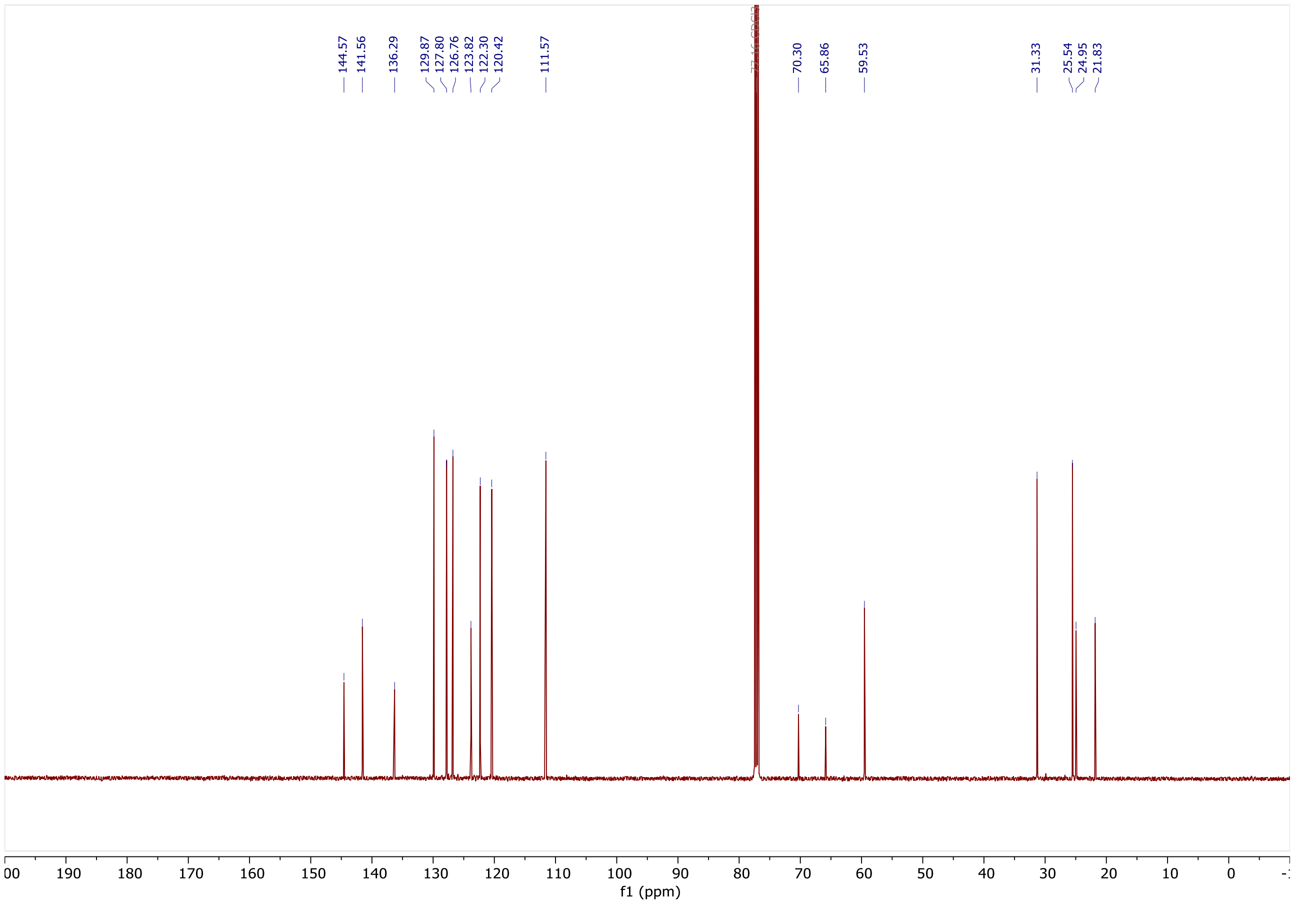

[2+2+2] Cyclotrimerization products

2aa *N*-Benzyl-*N*-(5,6-bis(hydroxymethyl)-4-methyl-1-tosylindolin-7-yl)-4-methylbenzenesulfonamide

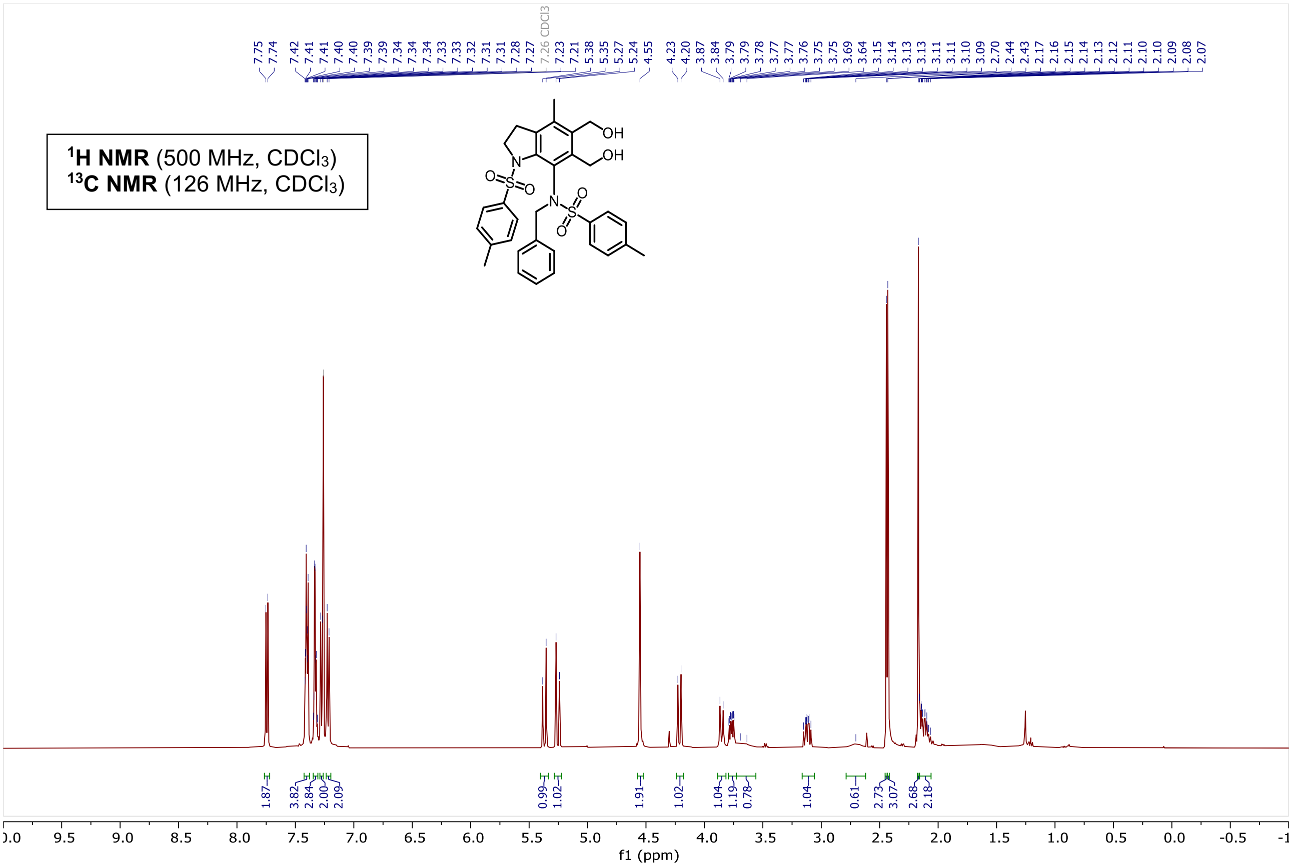

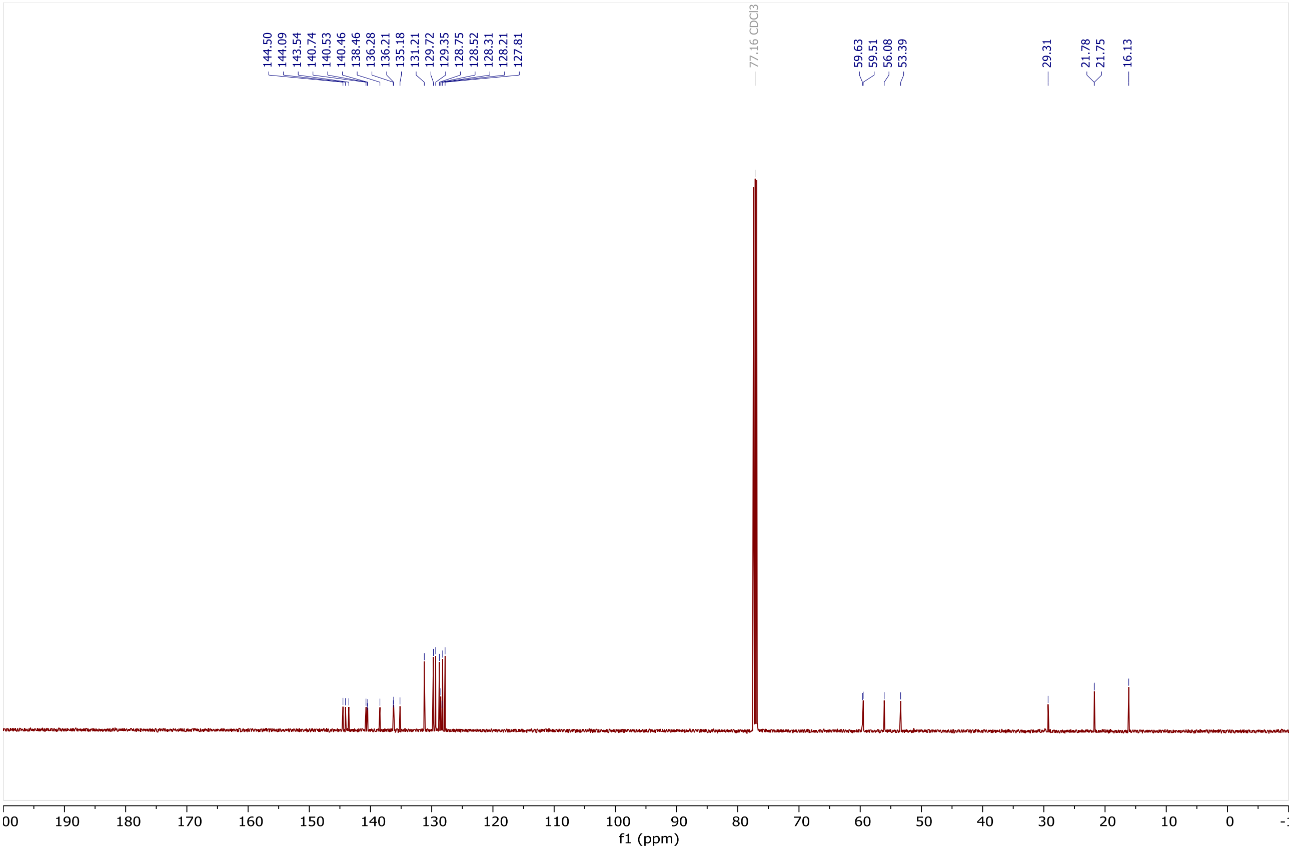

2ab *N*-Benzyl-*N*-(5-(4-(dimethylamino)phenyl)-4-methyl-1-tosylindolin-7-yl)-4-methylbenzenesulfonamide

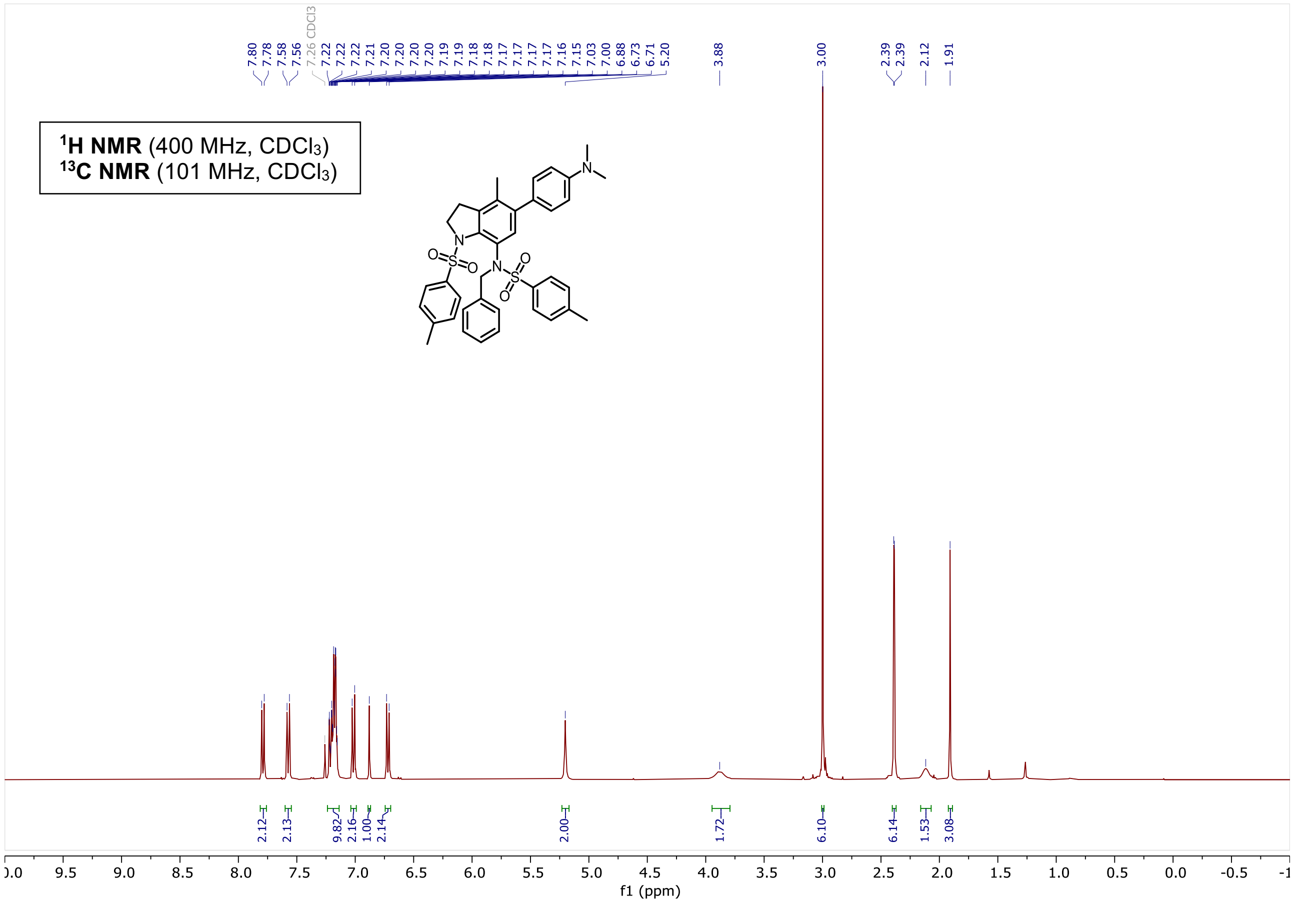

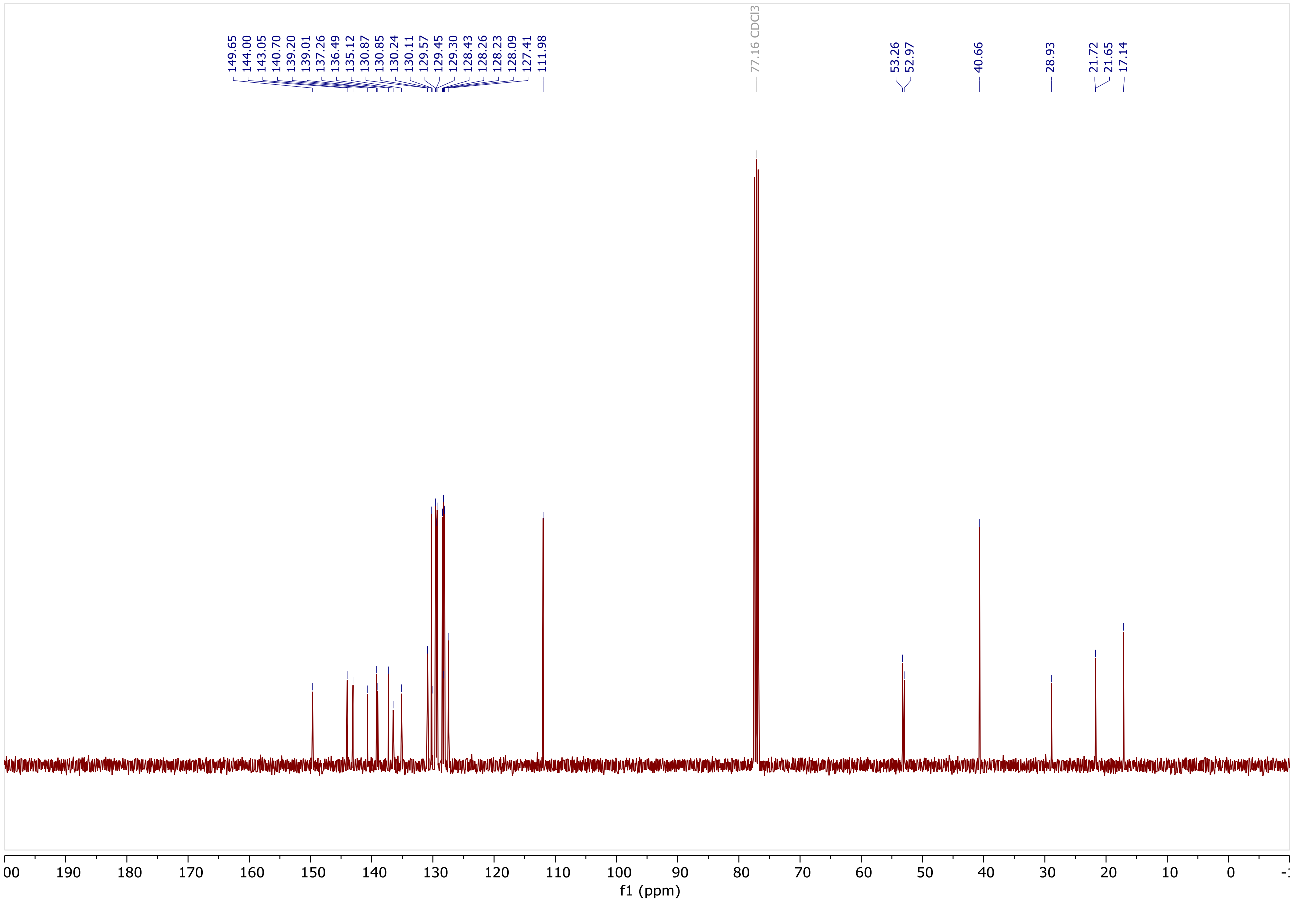

2ac ***N*-Benzyl-4-methyl-*N*-(4-methyl-5-(thiophen-2-yl)-1-tosylindolin-7-yl)benzenesulfonamide and *N*-benzyl-4-methyl-*N*-(4-methyl-6-(thiophen-2-yl)-1-tosylindolin-7-yl)benzenesulfonamide**

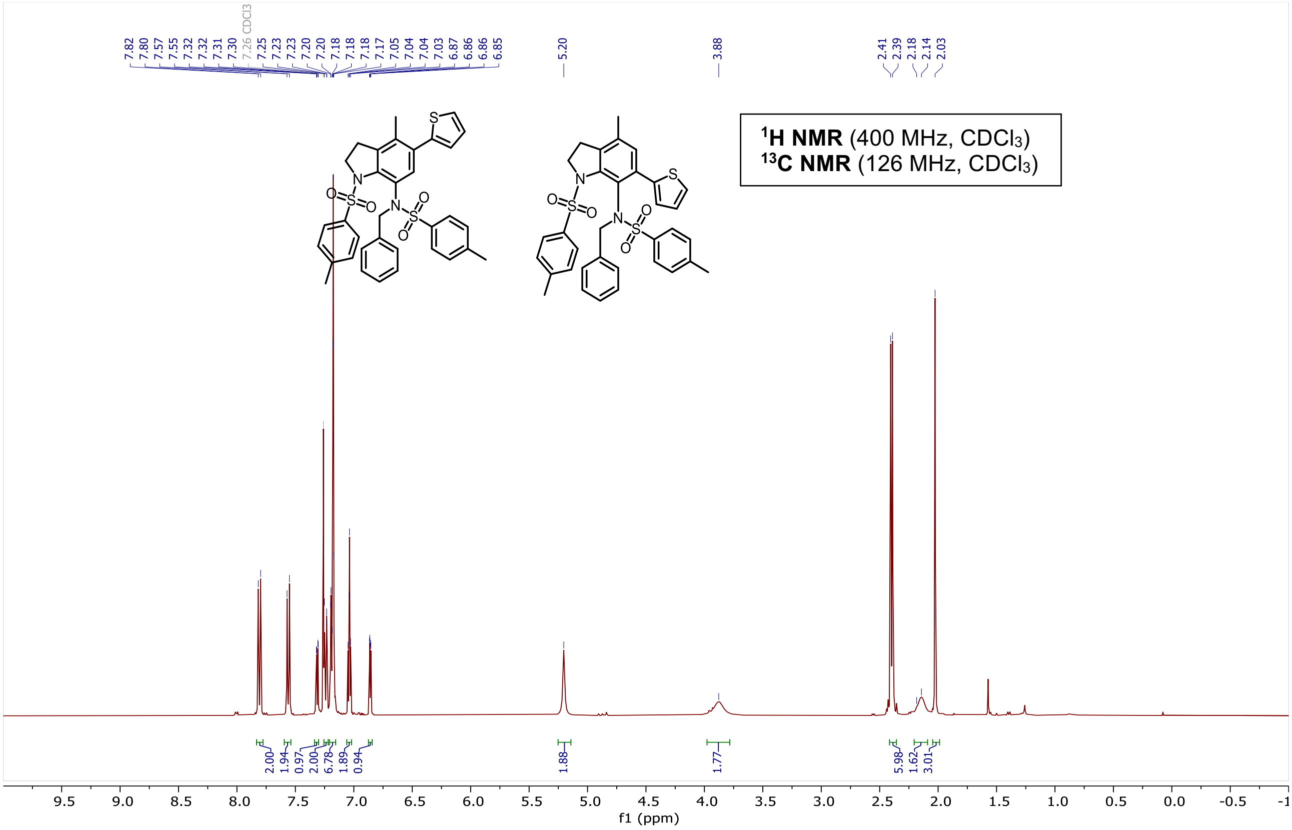

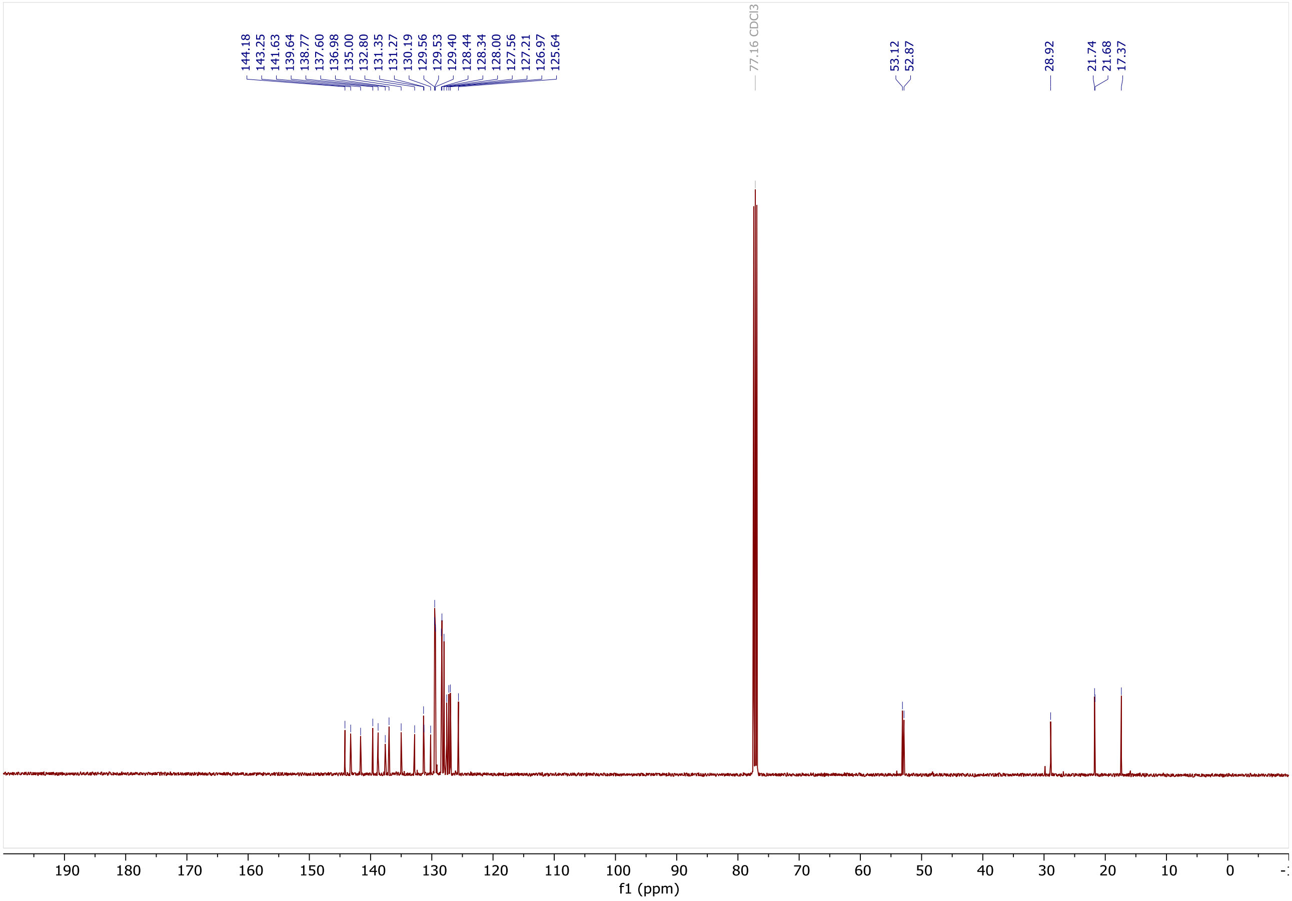

2ad ***N*-Benzyl-4-methyl-*N*-(4-methyl-5-(thiophen-3-yl)-1-tosylindolin-7-yl)benzenesulfonamide and *N*-benzyl-4-methyl-*N*-(4-methyl-6-(thiophen-3-yl)-1-tosylindolin-7-yl)benzenesulfonamide**

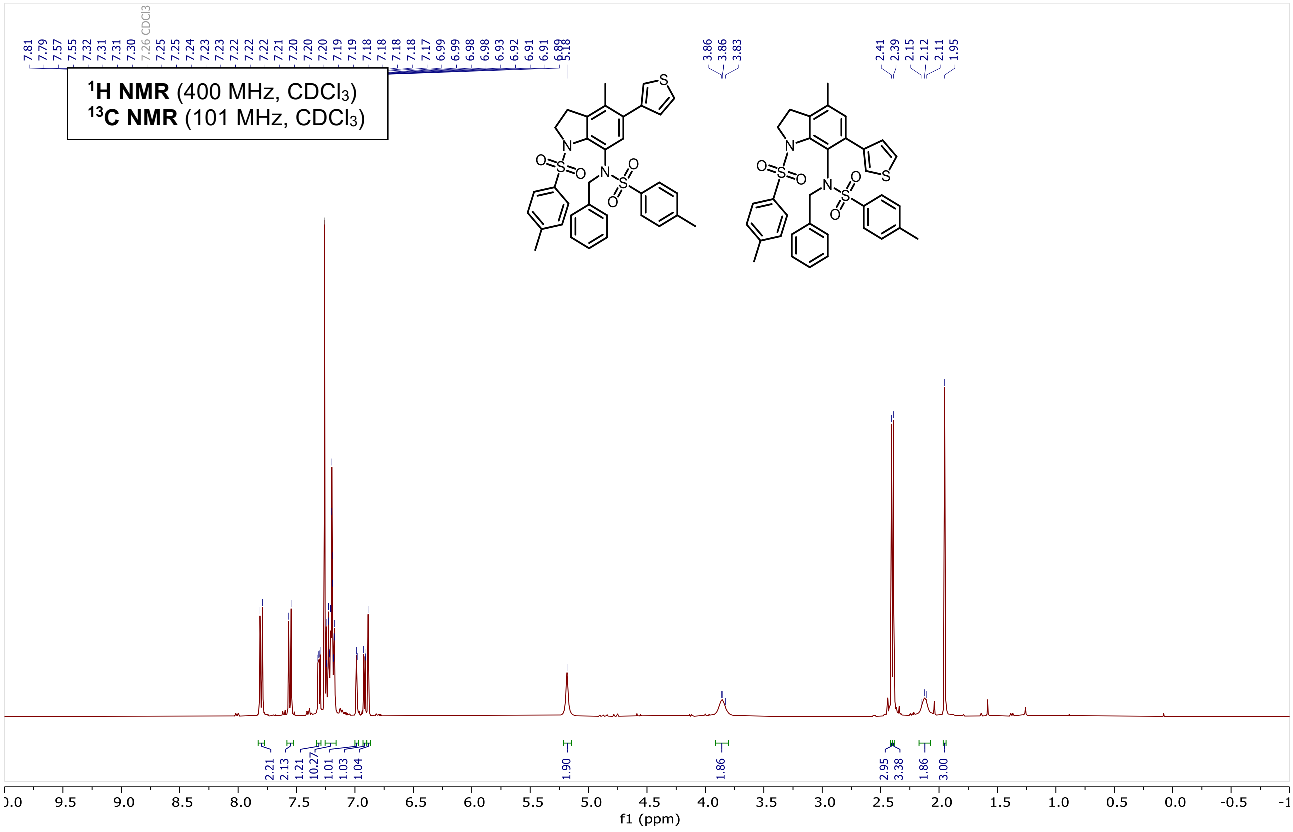

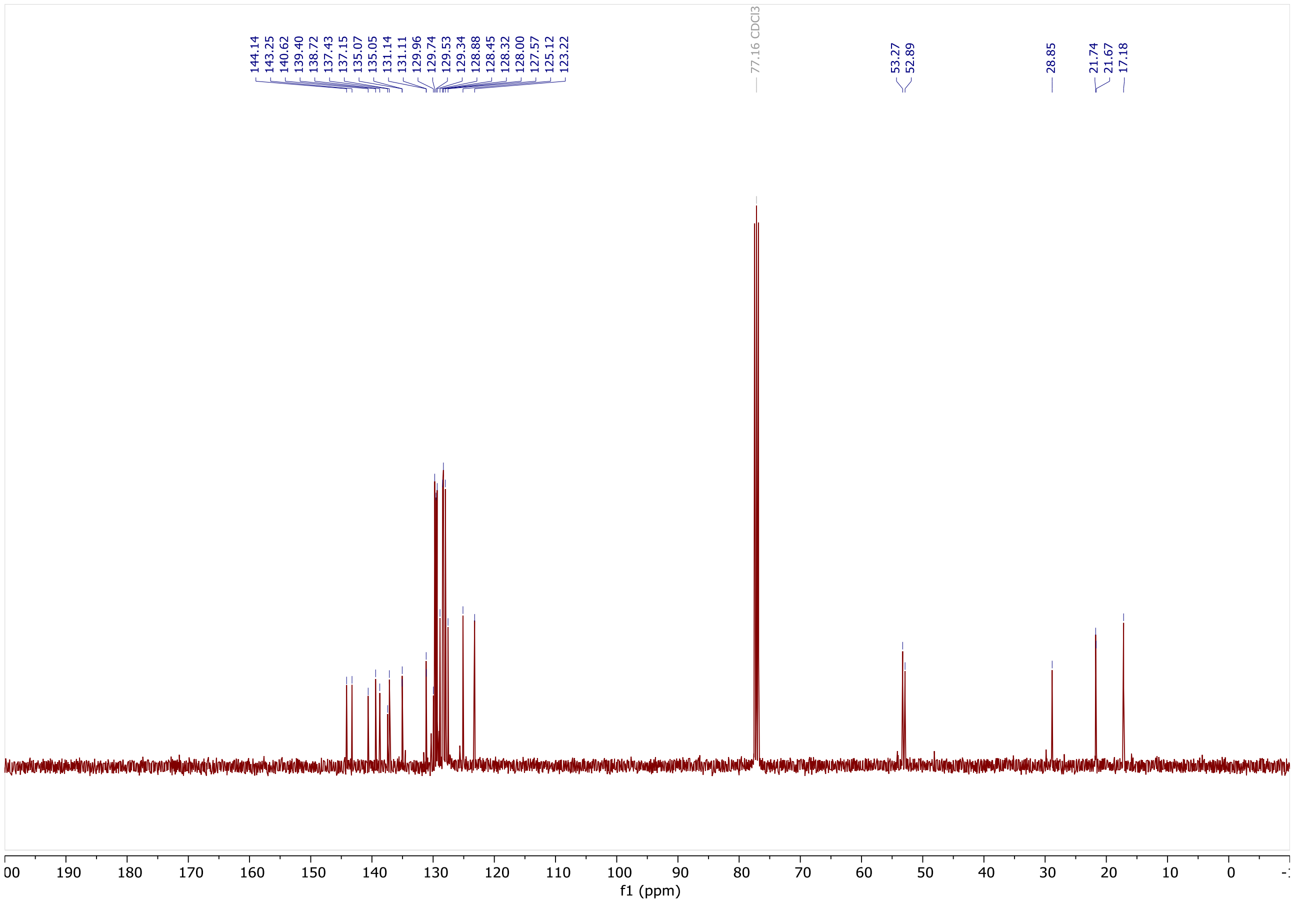

2ae ***N*-Benzyl-*N*-(5-(4-methoxyphenyl)-4-methyl-1-tosylindolin-7-yl)-4-methylbenzenesulfonamide** and ***N*-benzyl-*N*-(5-(4-methoxyphenyl)-4-methyl-1-tosylindolin-7-yl)-4-methylbenzenesulfonamide**

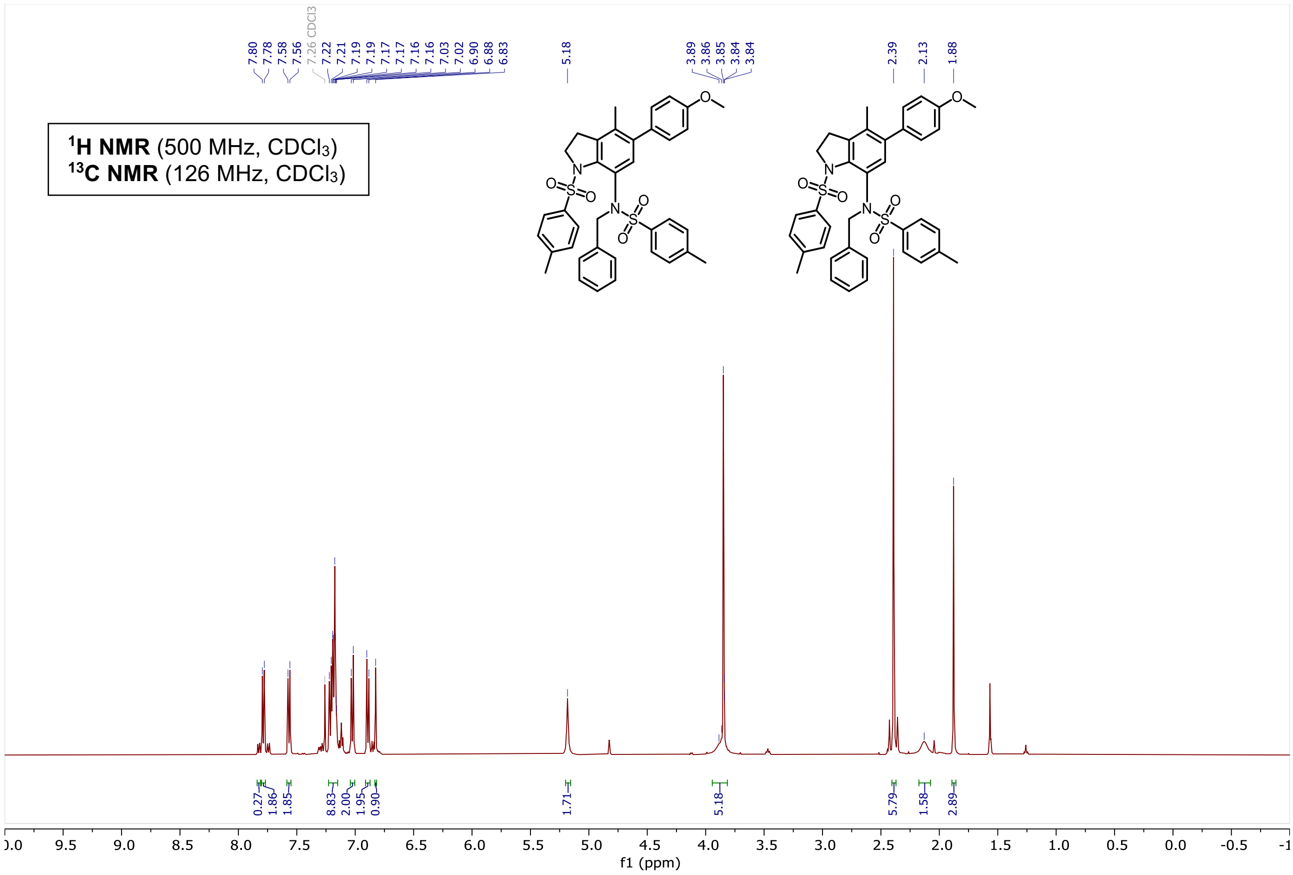

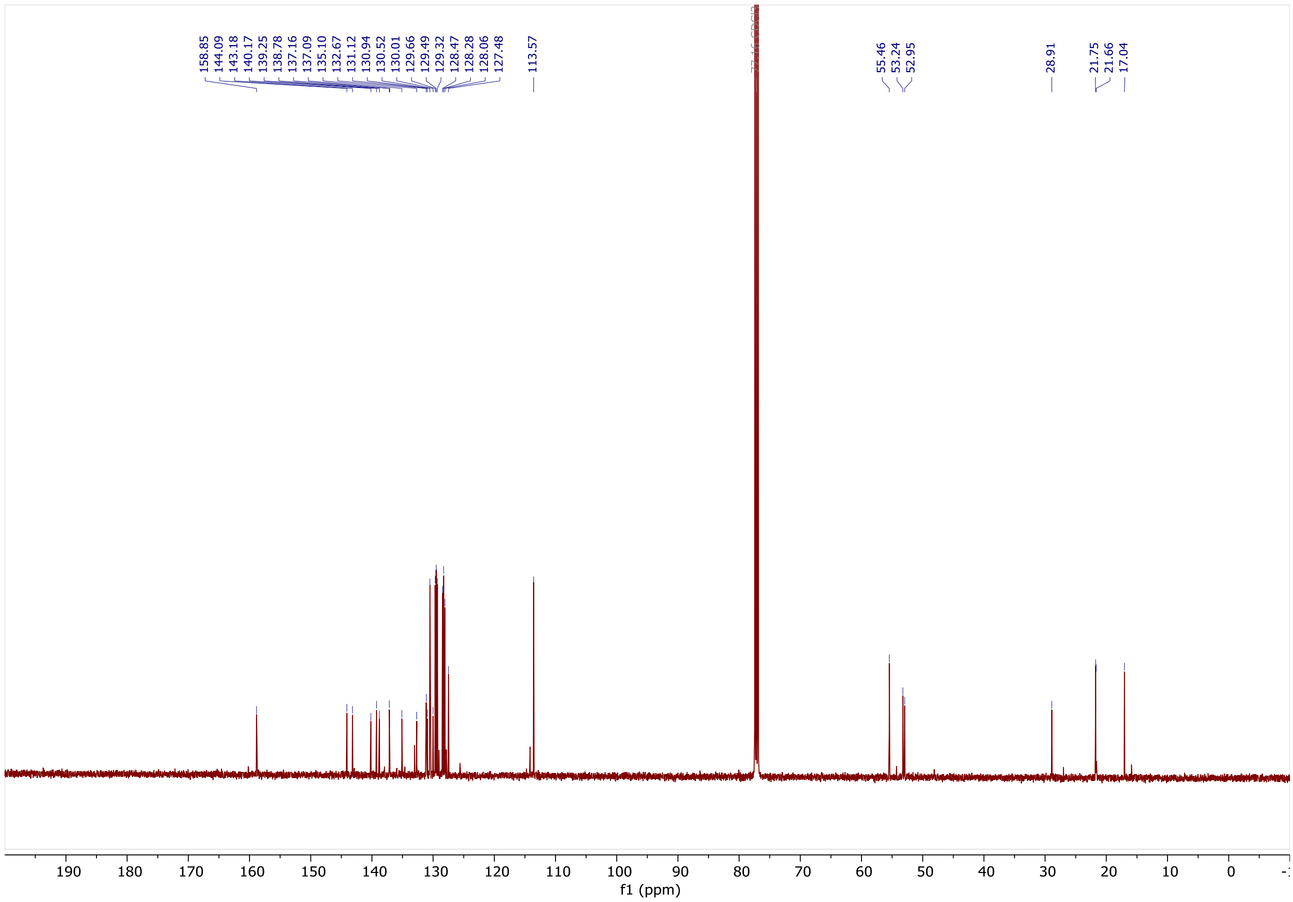

2af *N*-Benzyl-4-methyl-*N*-(4-methyl-5-phenyl-1-tosylindolin-7-yl)benzenesulfonamide and *N*-benzyl-4-methyl-*N*-(4-methyl-6-phenyl-1-tosylindolin-7-yl)benzenesulfonamide

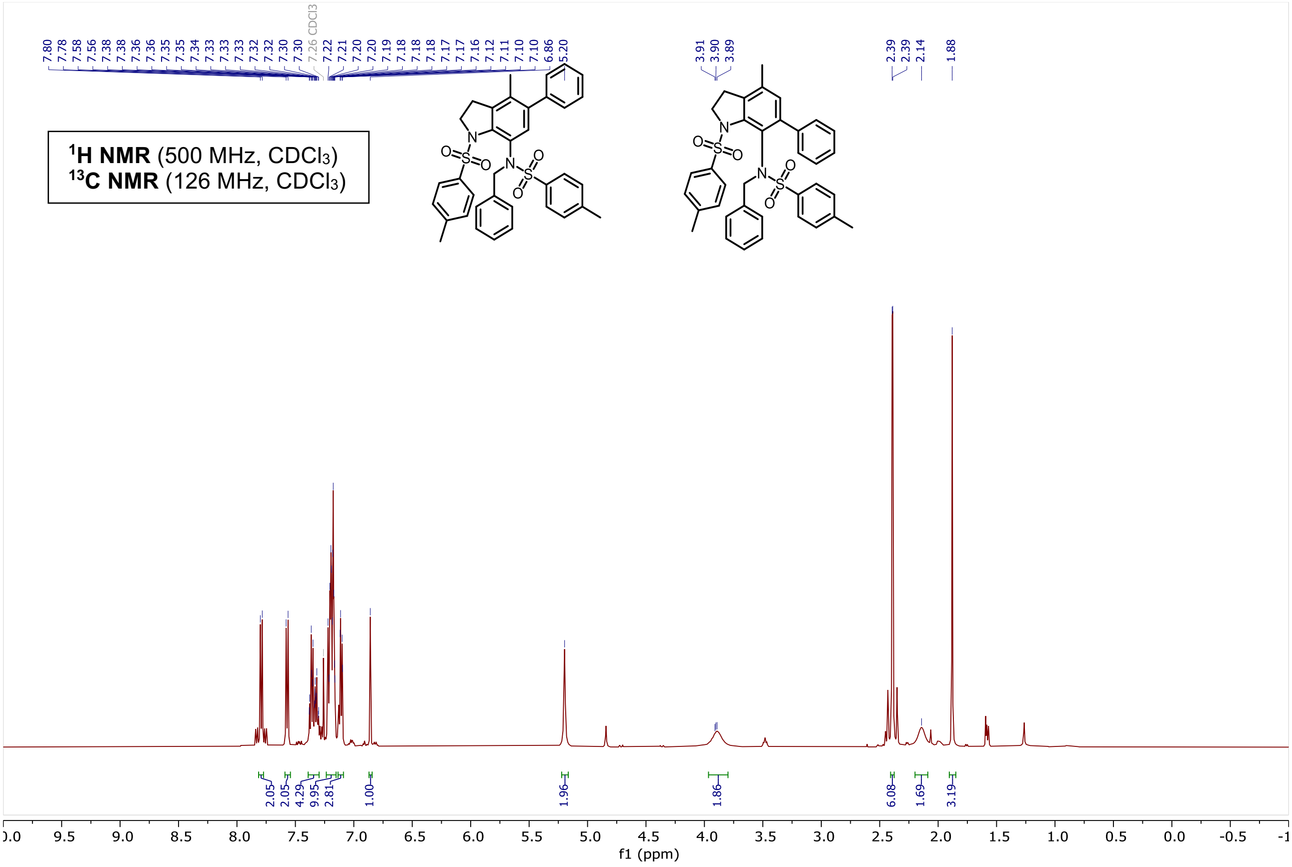

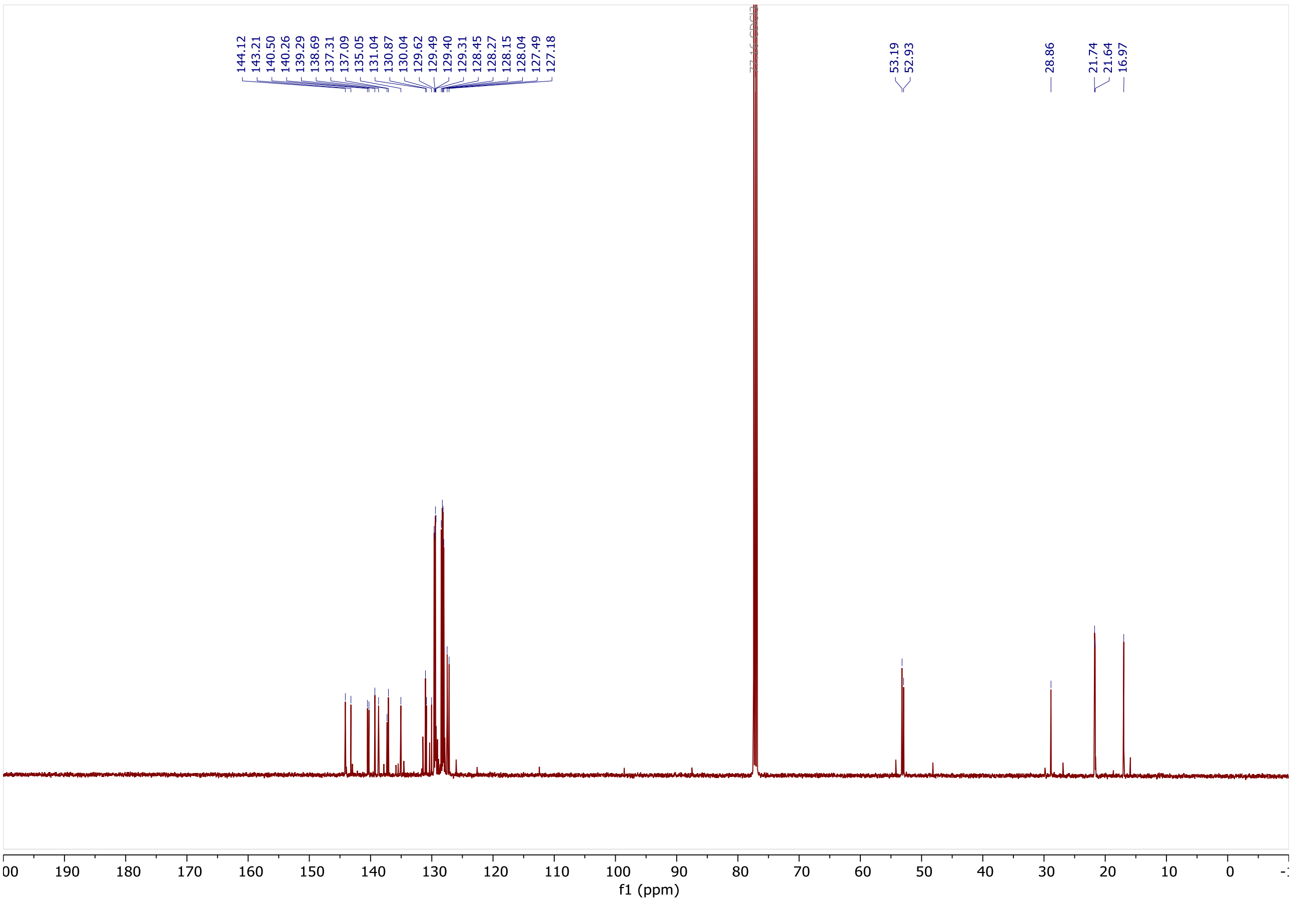

2ag *N*-Benzyl-4-methyl-*N*-(4-methyl-5-(p-tolyl)-1-tosylindolin-7-yl)benzenesulfonamide and ***N*-benzyl-4-methyl-*N*-(4-methyl-6-(p-tolyl)-1-tosylindolin-7-yl)benzenesulfonamide**

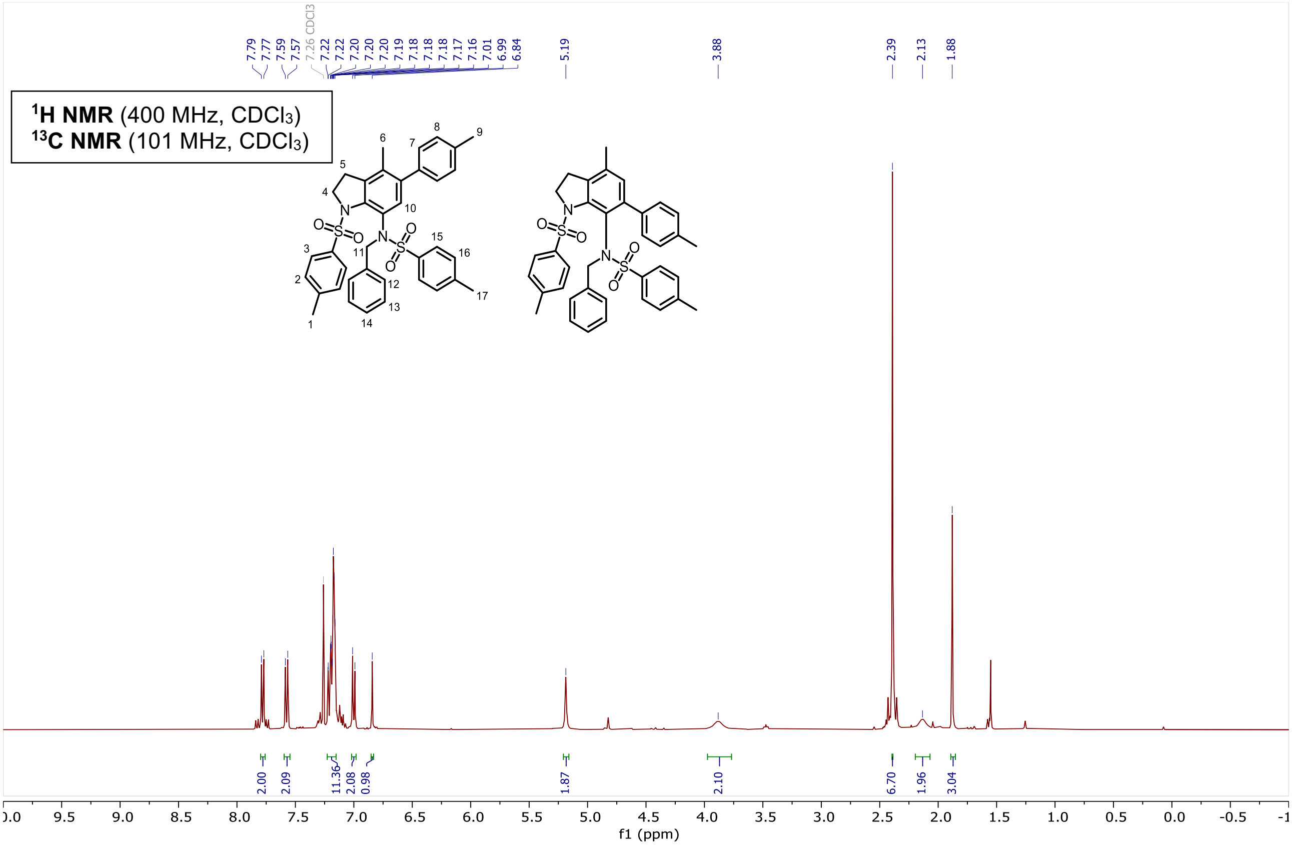

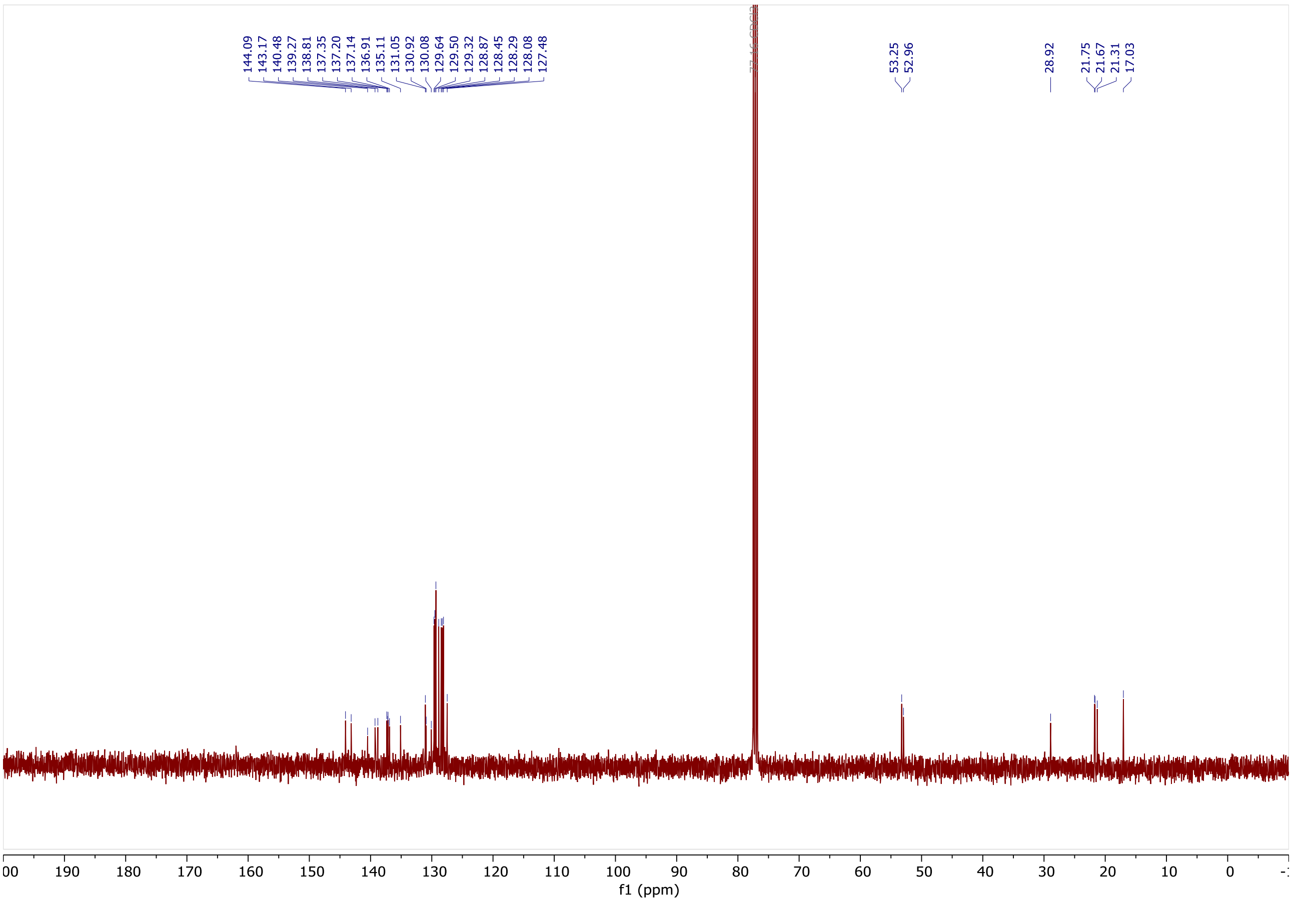

2ah ***N*-Benzyl-4-methyl-*N*-(4-methyl-5-(naphthalen-2-yl)-1-tosylindolin-7-yl)benzenesulfonamide**
and ***N*-benzyl-4-methyl-*N*-(4-methyl-6-(naphthalen-2-yl)-1-tosylindolin-7-yl)benzenesulfonamide**

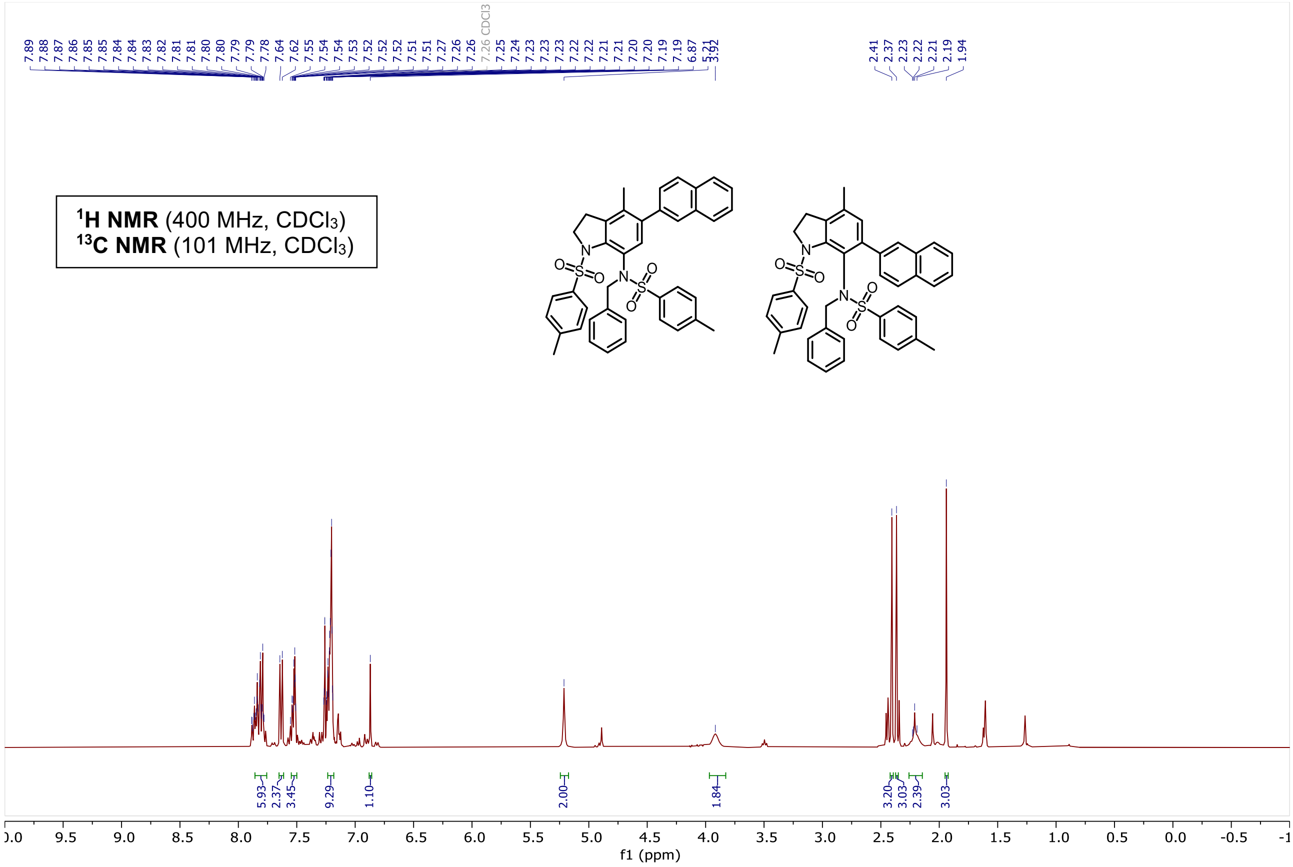

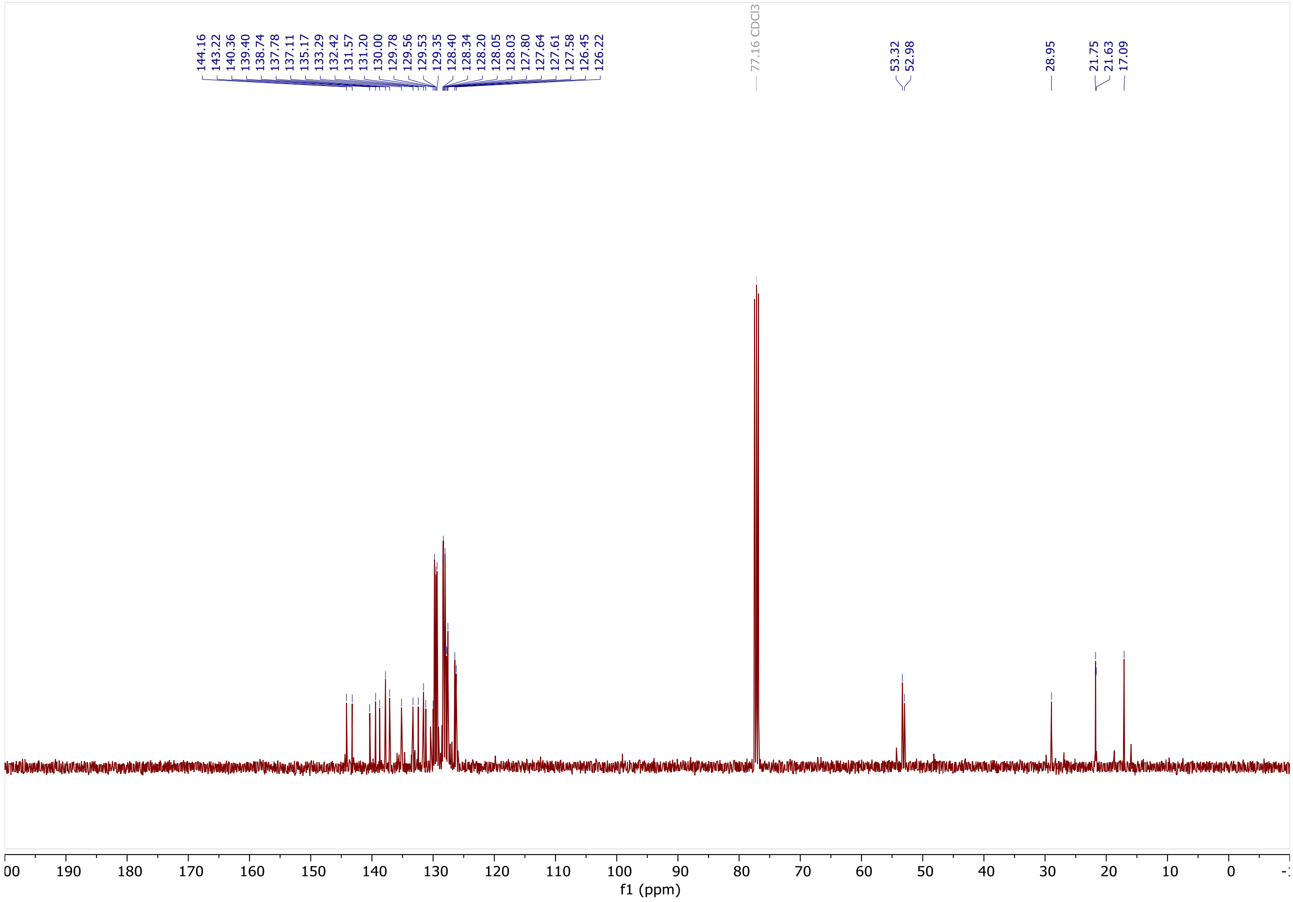

2ai *N*-Benzyl-*N*-(5-(4-fluorophenyl)-4-methyl-1-tosylindolin-7-yl)-4-methylbenzenesulfonamide

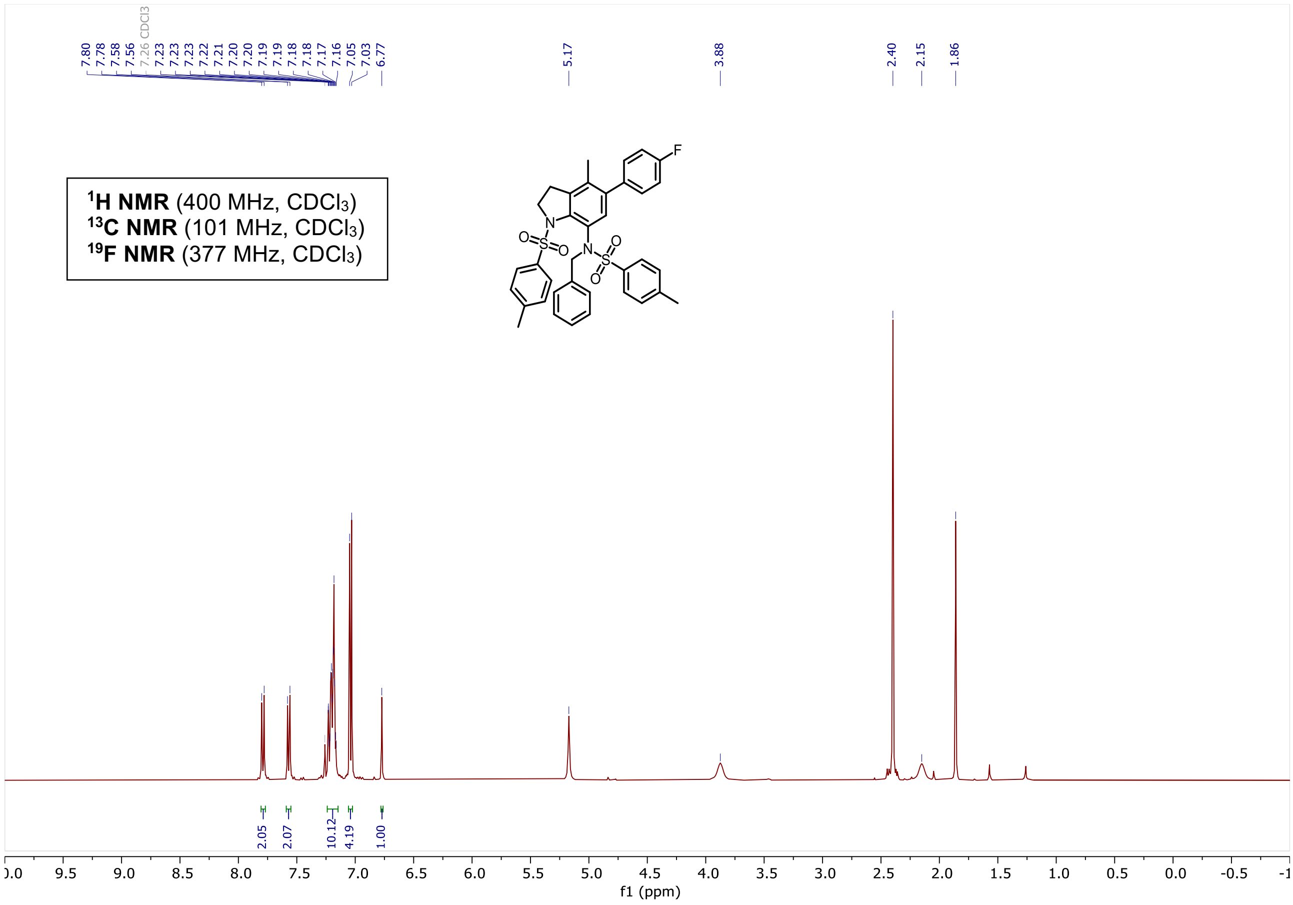

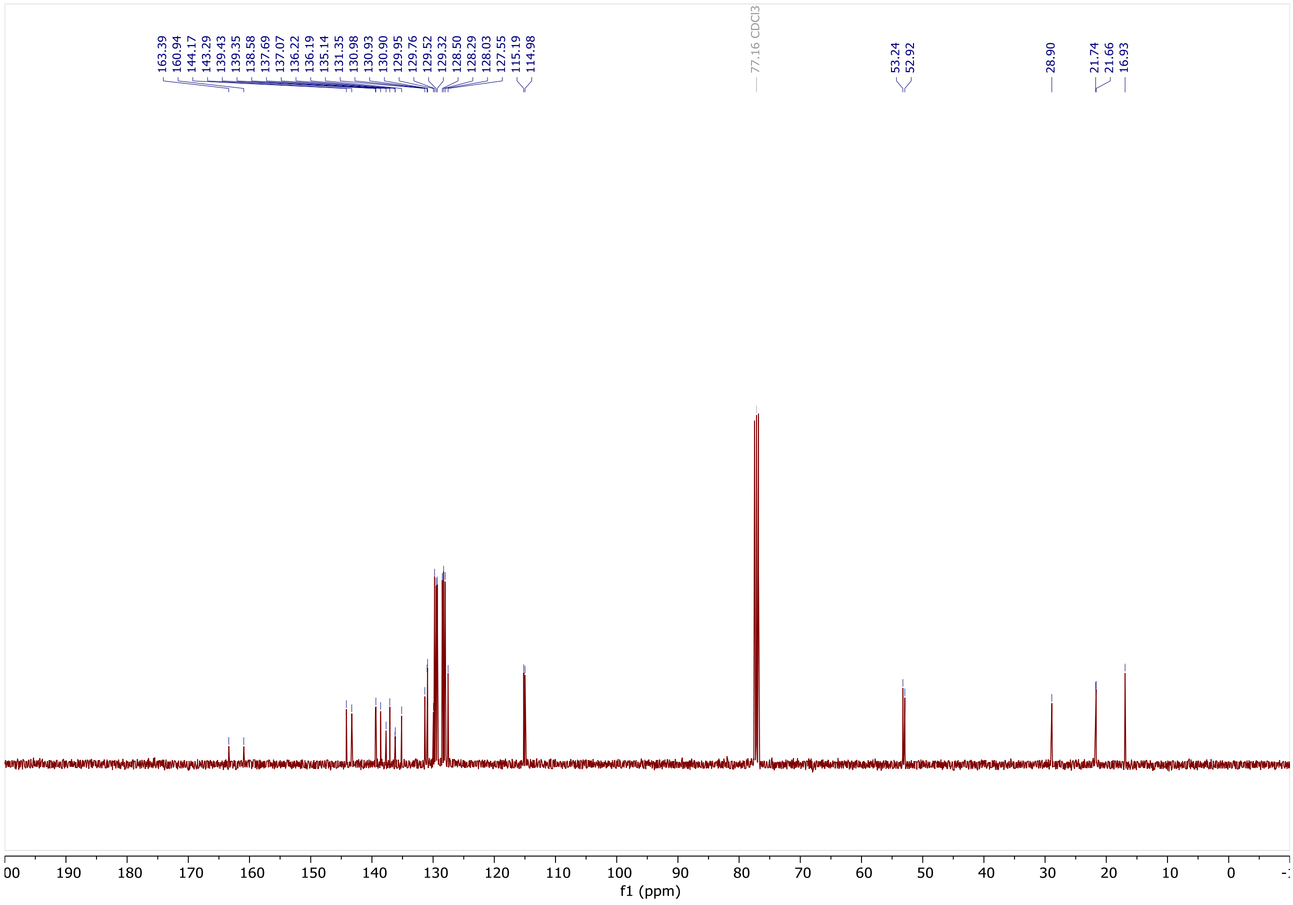

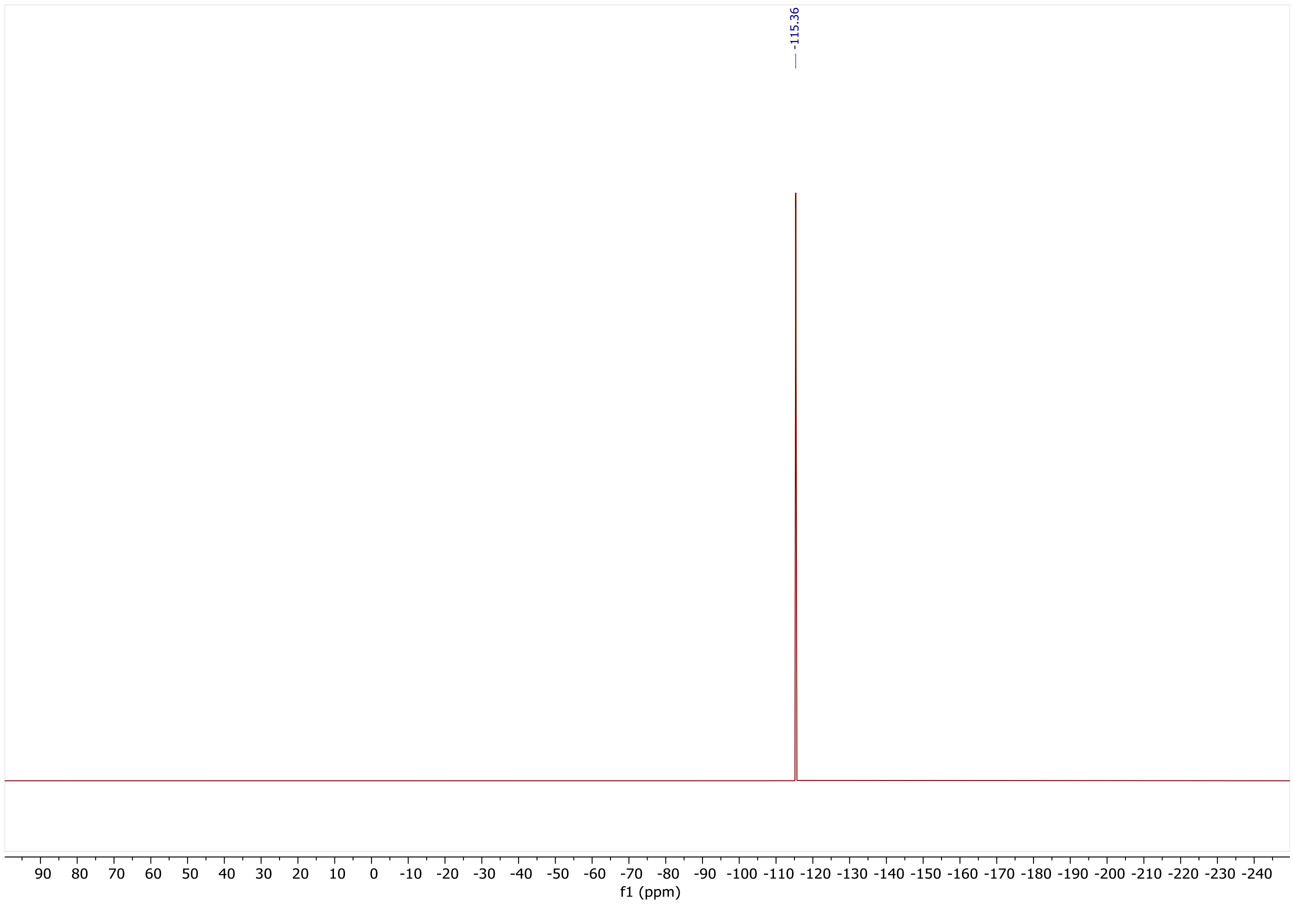

-115.36
90 80 70 60 50 40 30 20 10 0 -10 -20 -30 -40 -50 -60 -70 -80 -90 -100 -110 -120 -130 -140 -150 -160 -170 -180 -190 -200 -210 -220 -230 -240
f1 (ppm)

2aj ***N*-Benzyl-*N*-(5-(3,4-dichlorophenyl)-4-methyl-1-tosylindolin-7-yl)-4-methylbenzenesulfonamide & *N*-benzyl-*N*-(6-(3,4-dichlorophenyl)-4-methyl-1-tosylindolin-7-yl)-4-methylbenzenesulfonamide**

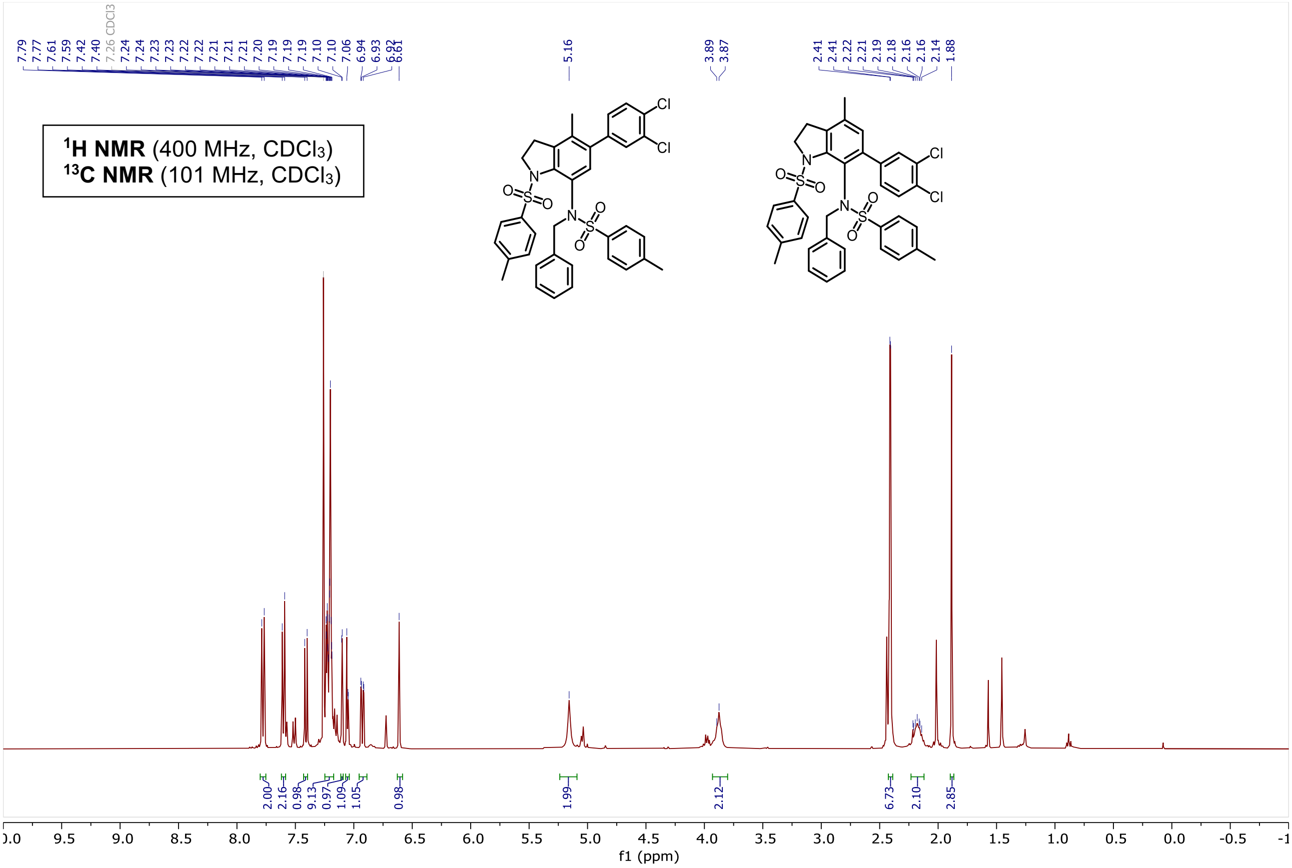

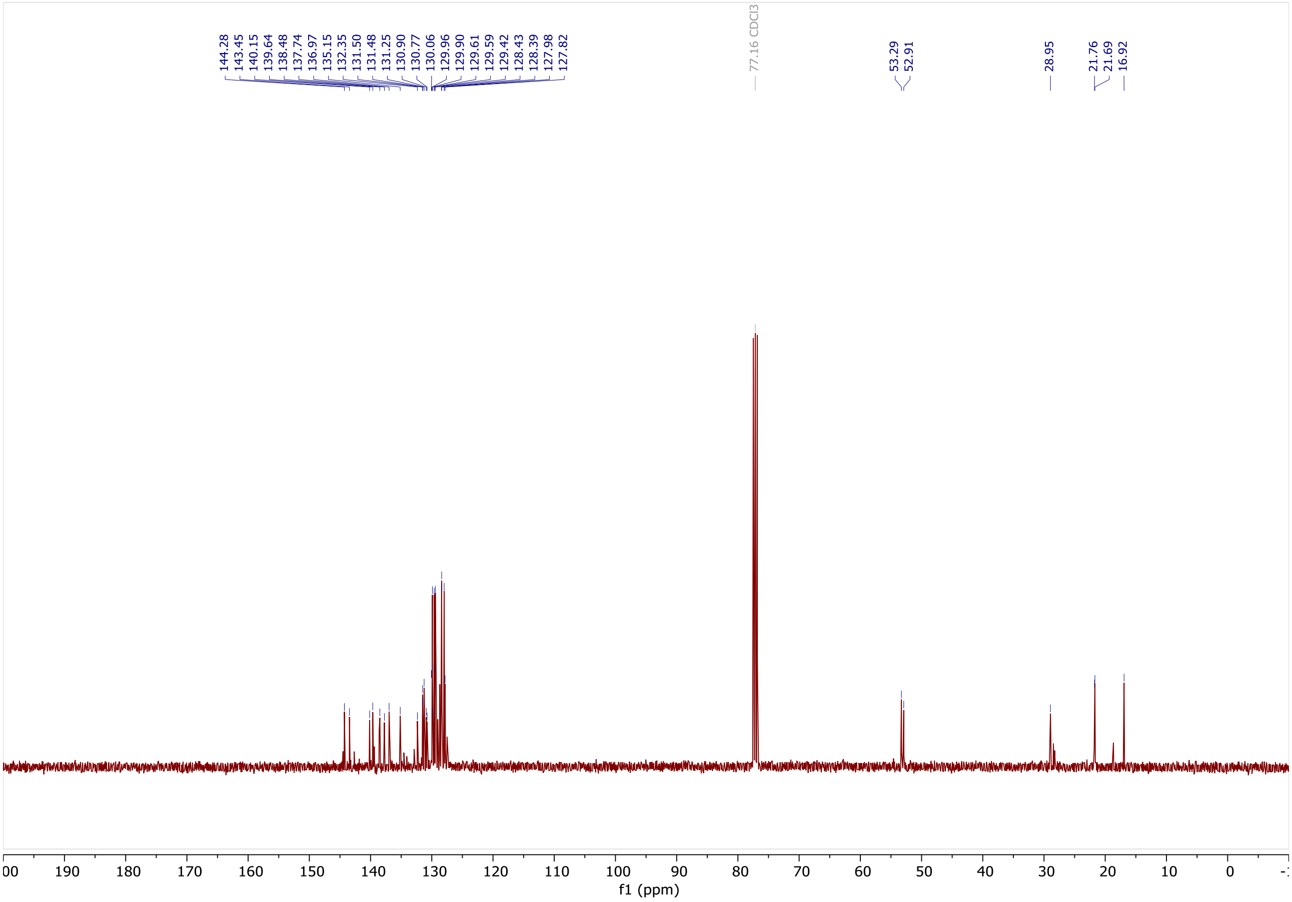

2ak ***N*-Benzyl-*N*-(5-(3-fluorophenyl)-4-methyl-1-tosylindolin-7-yl)-4-methylbenzenesulfonamide & *N*-benzyl-*N*-(6-(3-fluorophenyl)-4-methyl-1-tosylindolin-7-yl)-4-methylbenzenesulfonamide**

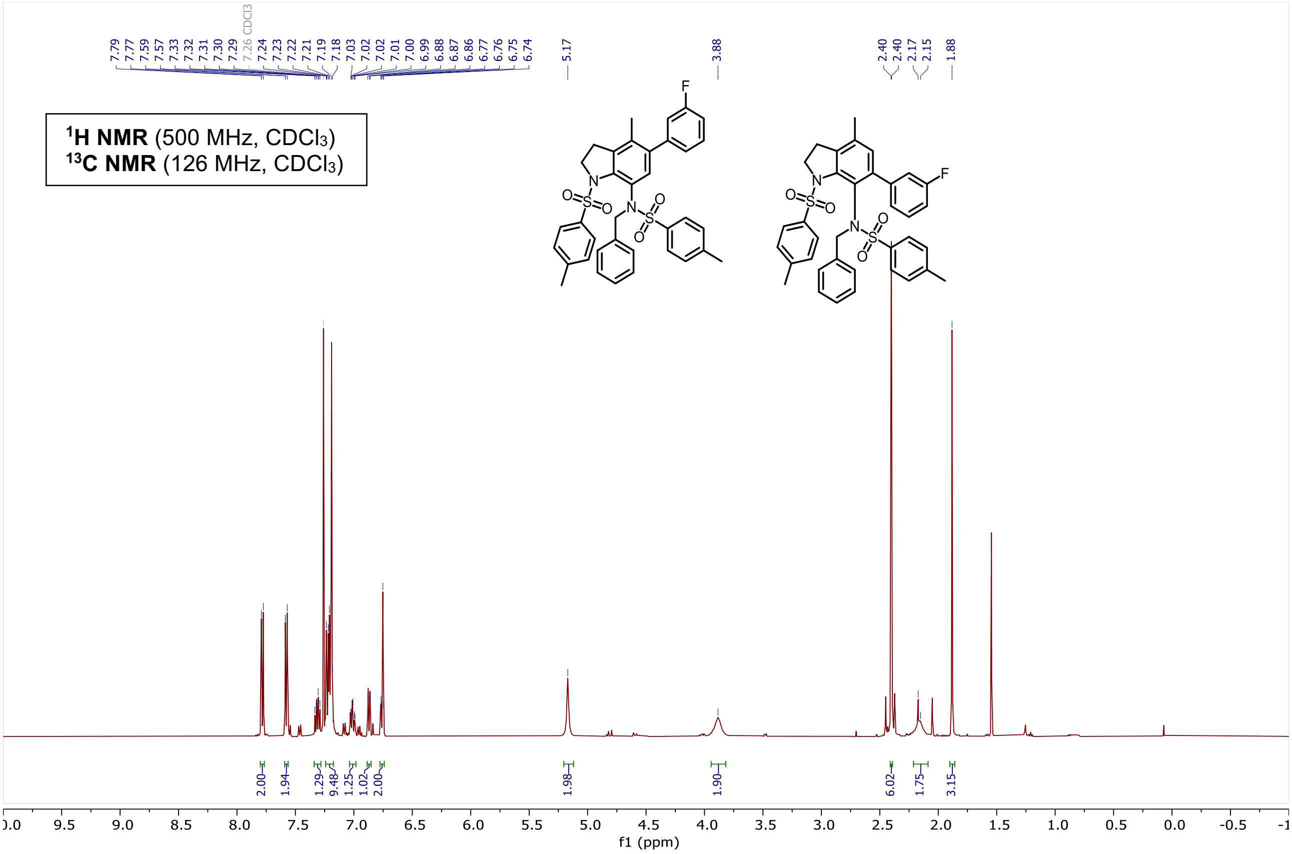

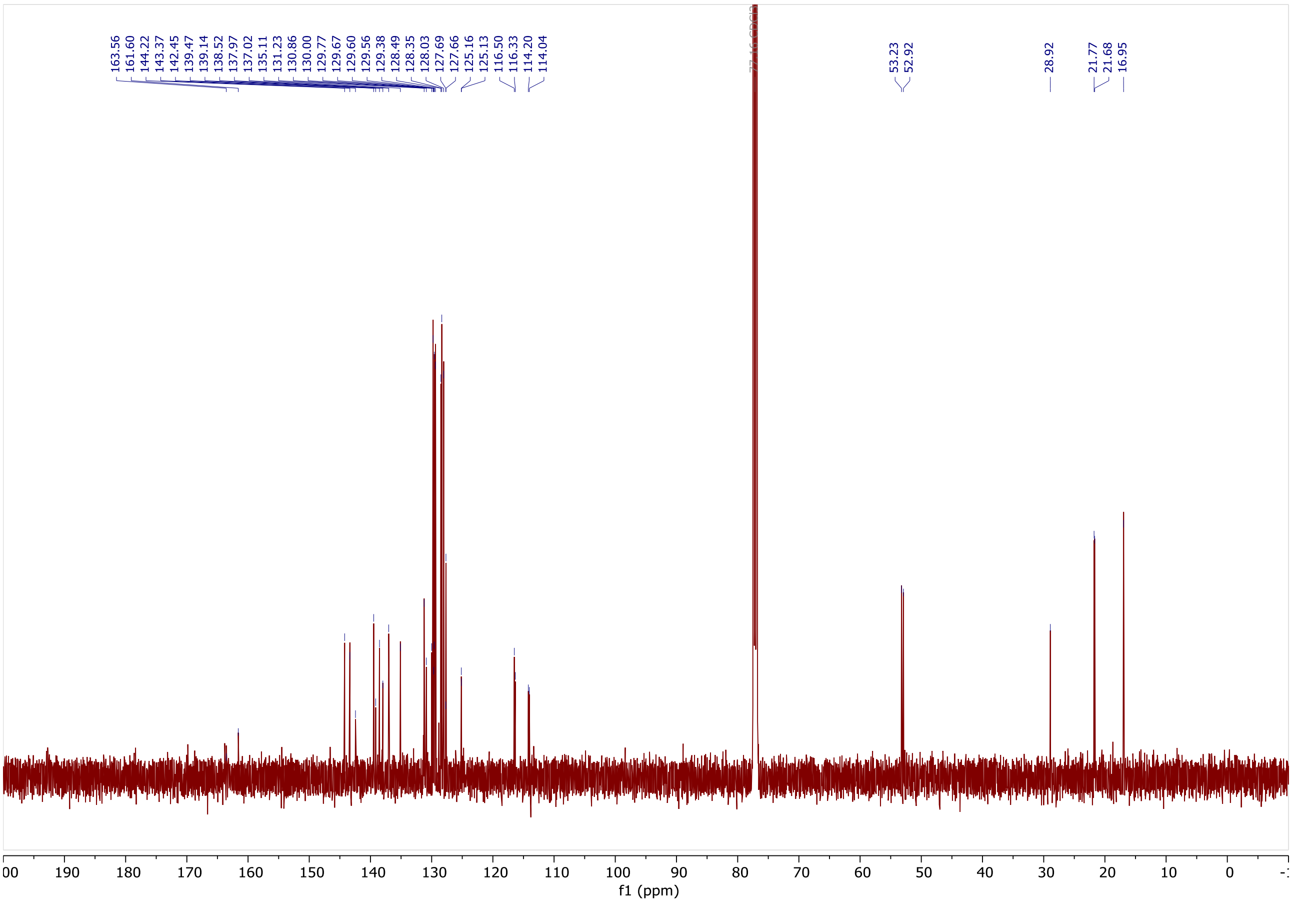

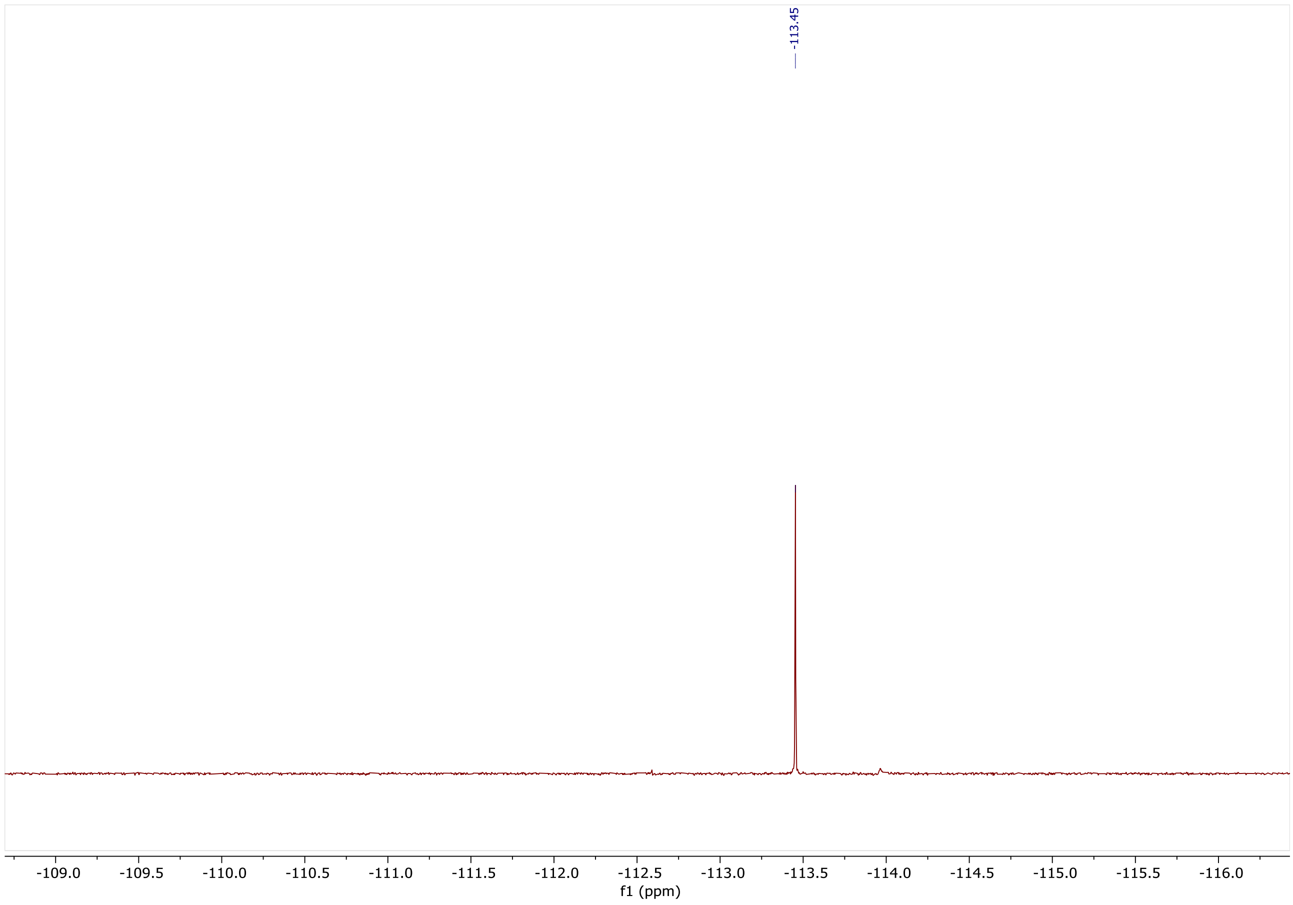
-113.45
-109.0
-109.5
-110.0
-110.5
-111.0
-111.5
-112.0
-112.5
-113.0
-113.5
-114.0
-114.5
-115.0
-115.5
-116.0
f1 (ppm)

2al ***N*-Benzyl-4-methyl-*N*-(4-methyl-5-(4-(4,4,5,5-tetramethyl-1,3,2-dioxaborolan-2-yl)phenyl)-1-tosylindolin-7-yl)benzenesulfonamide** and ***N*-benzyl-4-methyl-*N*-(4-methyl-6-(4-(4,4,5,5-tetramethyl-1,3,2-dioxaborolan-2-yl)phenyl)-1-tosylindolin-7-yl)benzenesulfonamide**

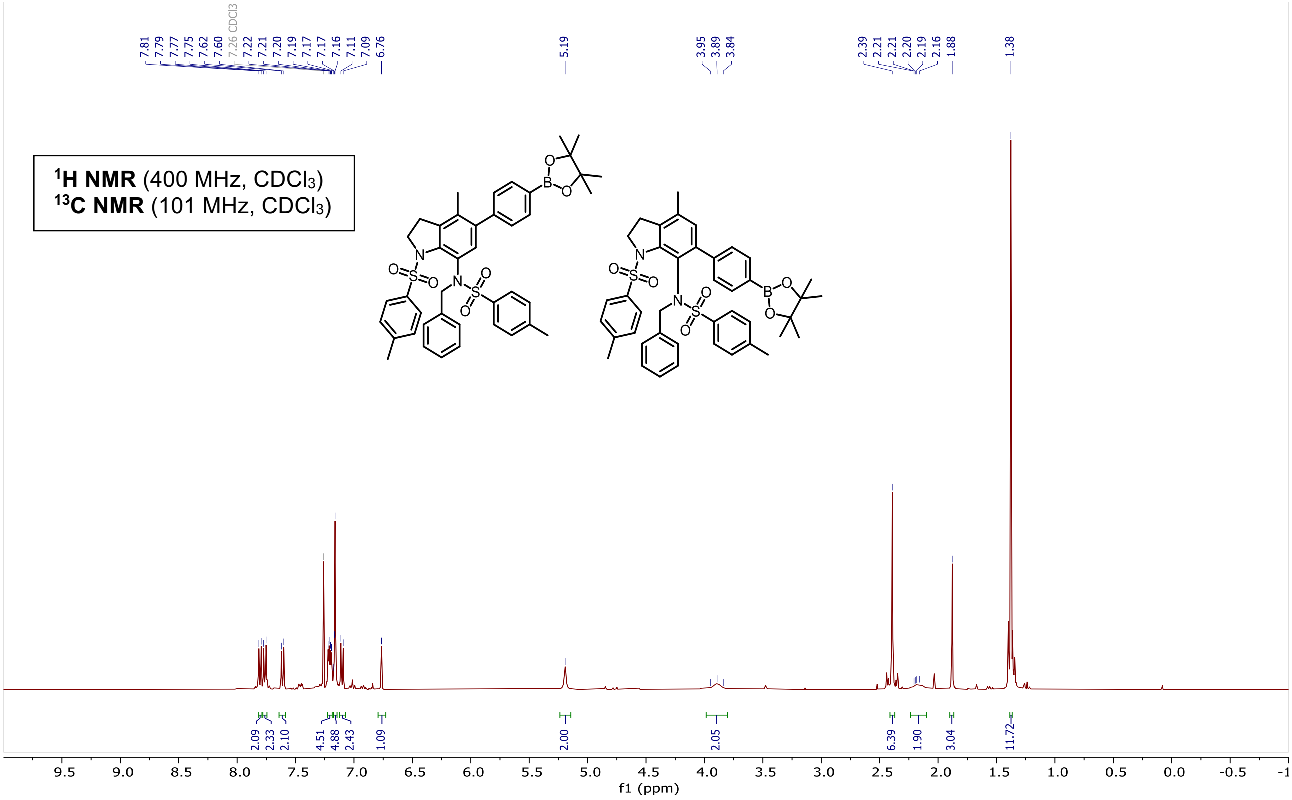

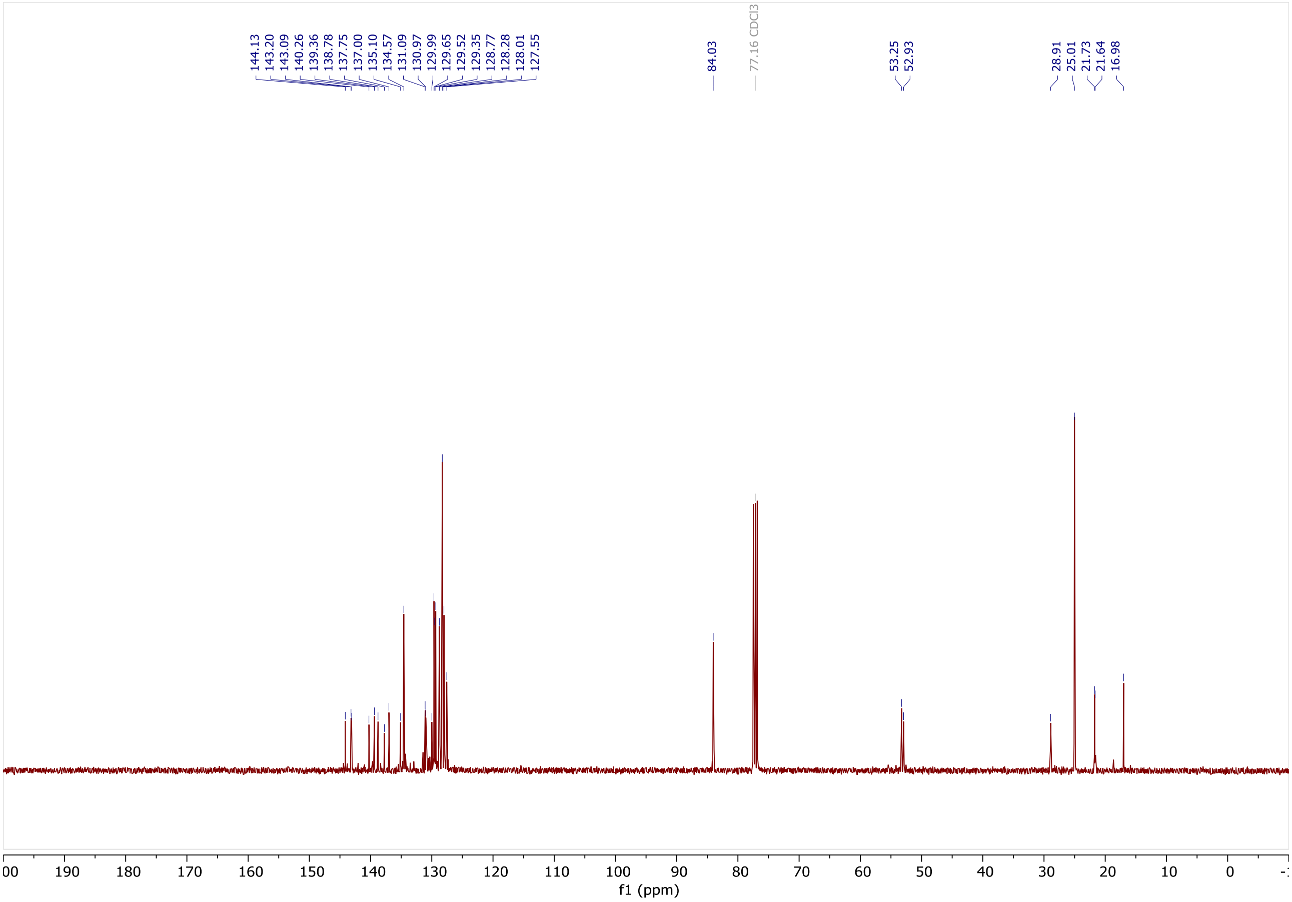

2an ***N*-Benzyl-4-methyl-*N*-(4-methyl-1-tosyl-5-(4-(trifluoromethyl)phenyl)indolin-7-yl)benzenesulfonamide and *N*-benzyl-4-methyl-*N*-(4-methyl-1-tosyl-6-(4-(trifluoromethyl)phenyl)indolin-7-yl)benzenesulfonamide**

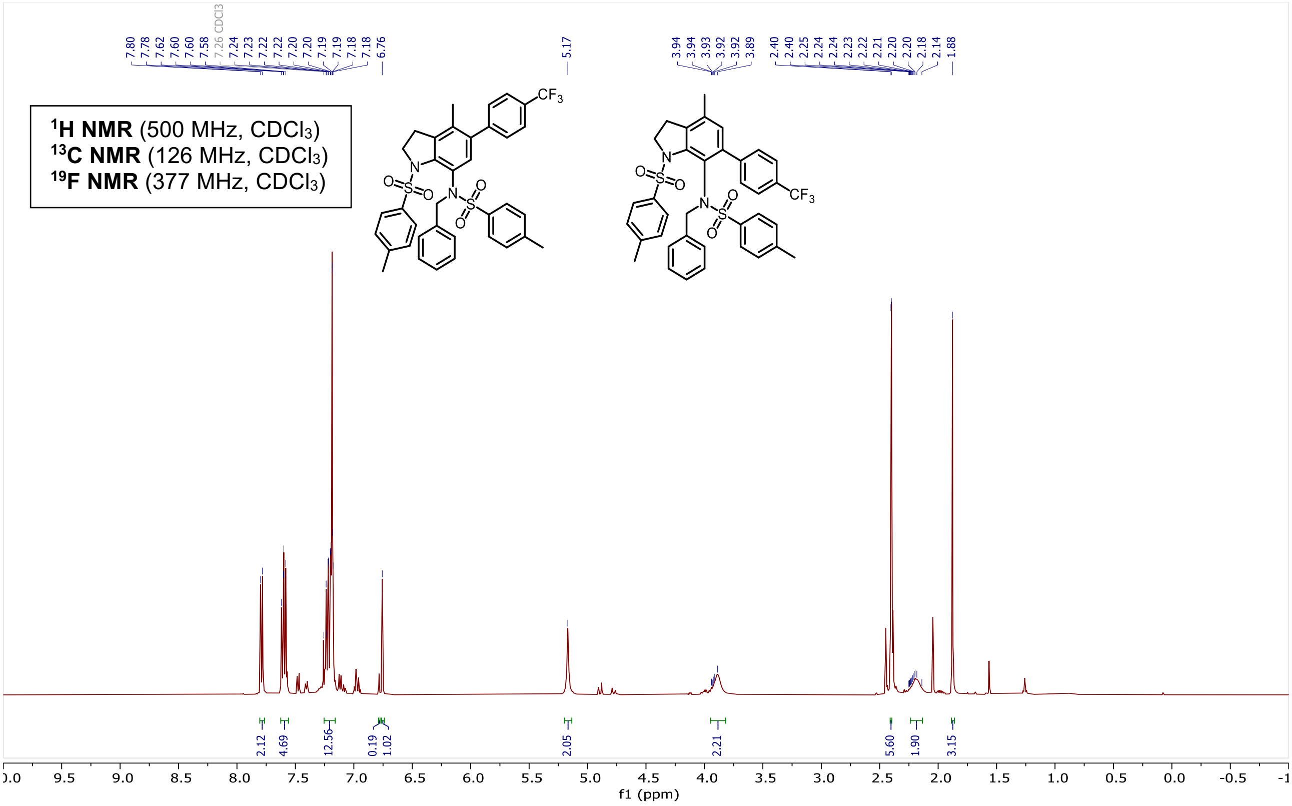

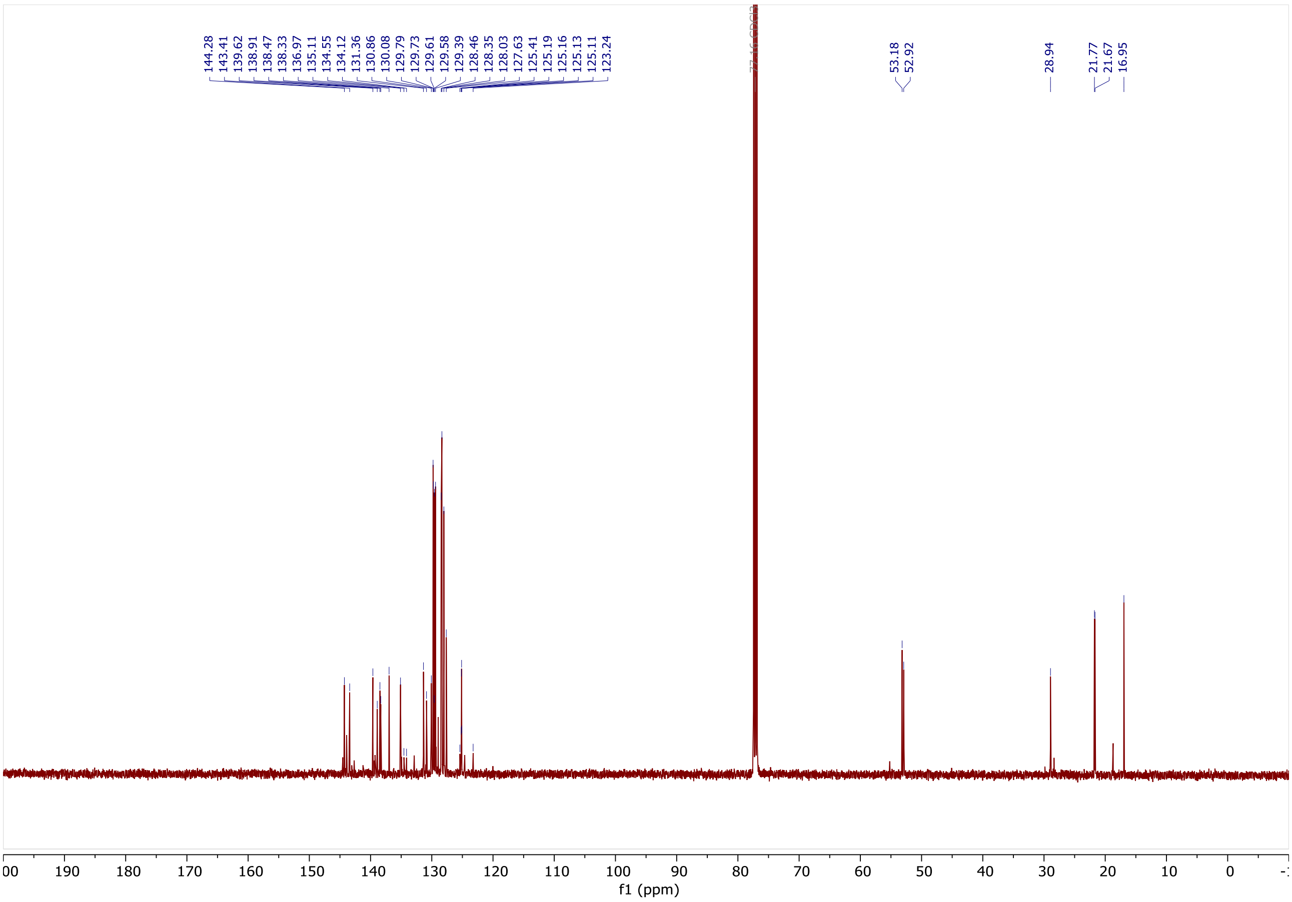

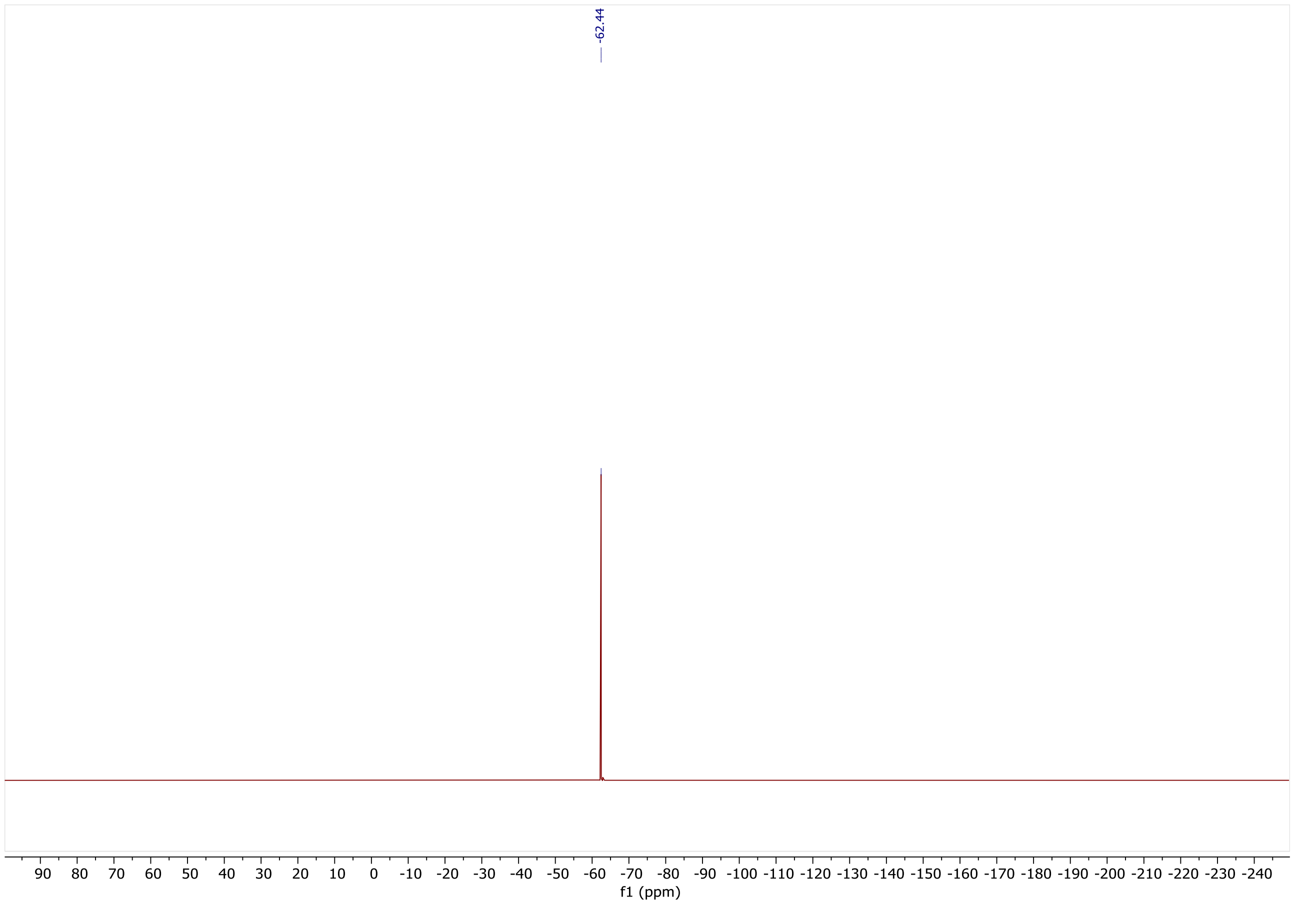
-62.44
90 80 70 60 50 40 30 20 10 0 -10 -20 -30 -40 -50 -60 -70 -80 -90 -100 -110 -120 -130 -140 -150 -160 -170 -180 -190 -200 -210 -220 -230 -240
f1 (ppm)

2ao ***N*-Benzyl-*N*-(5-(4-cyanophenyl)-4-methyl-1-tosylindolin-7-yl)-4-methylbenzenesulfonamide & *N*-benzyl-*N*-(6-(4-cyanophenyl)-4-methyl-1-tosylindolin-7-yl)-4-methylbenzenesulfonamide**

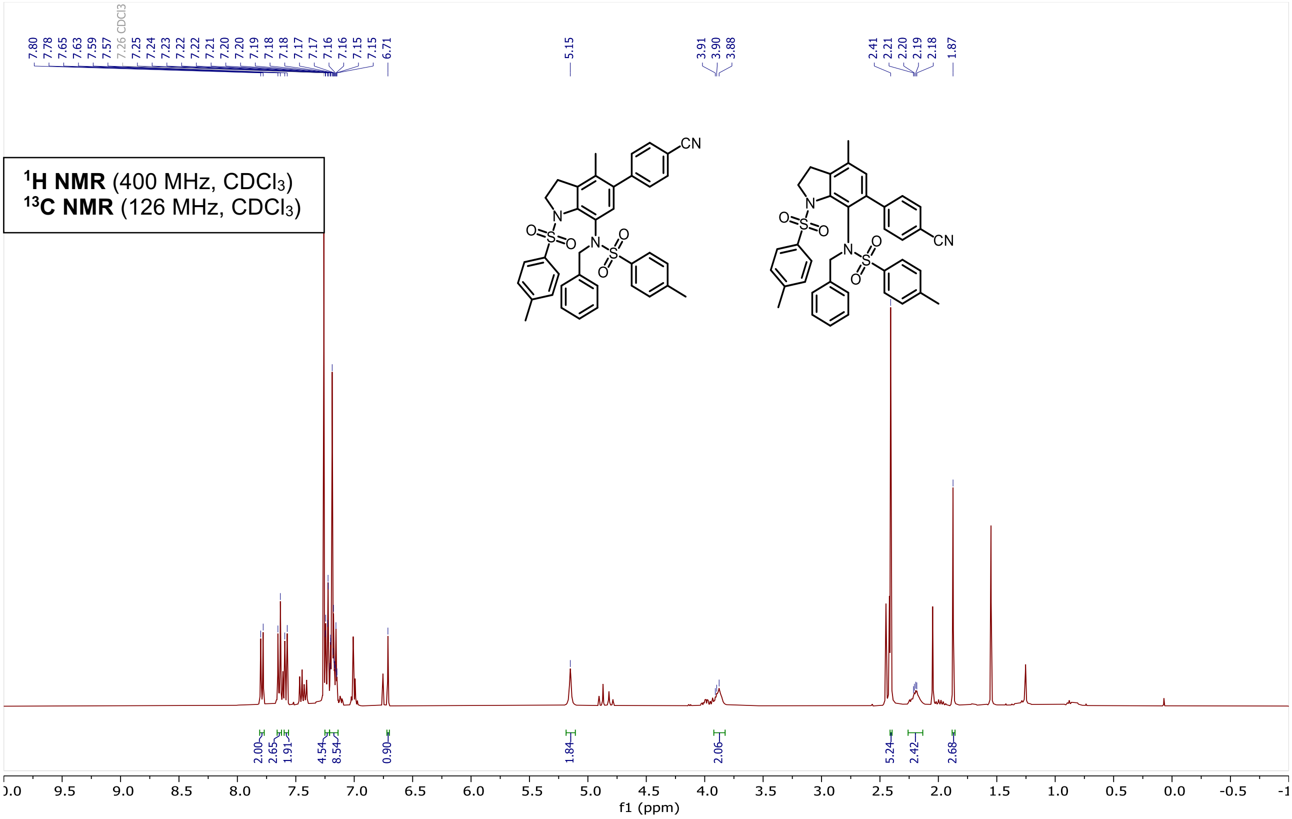

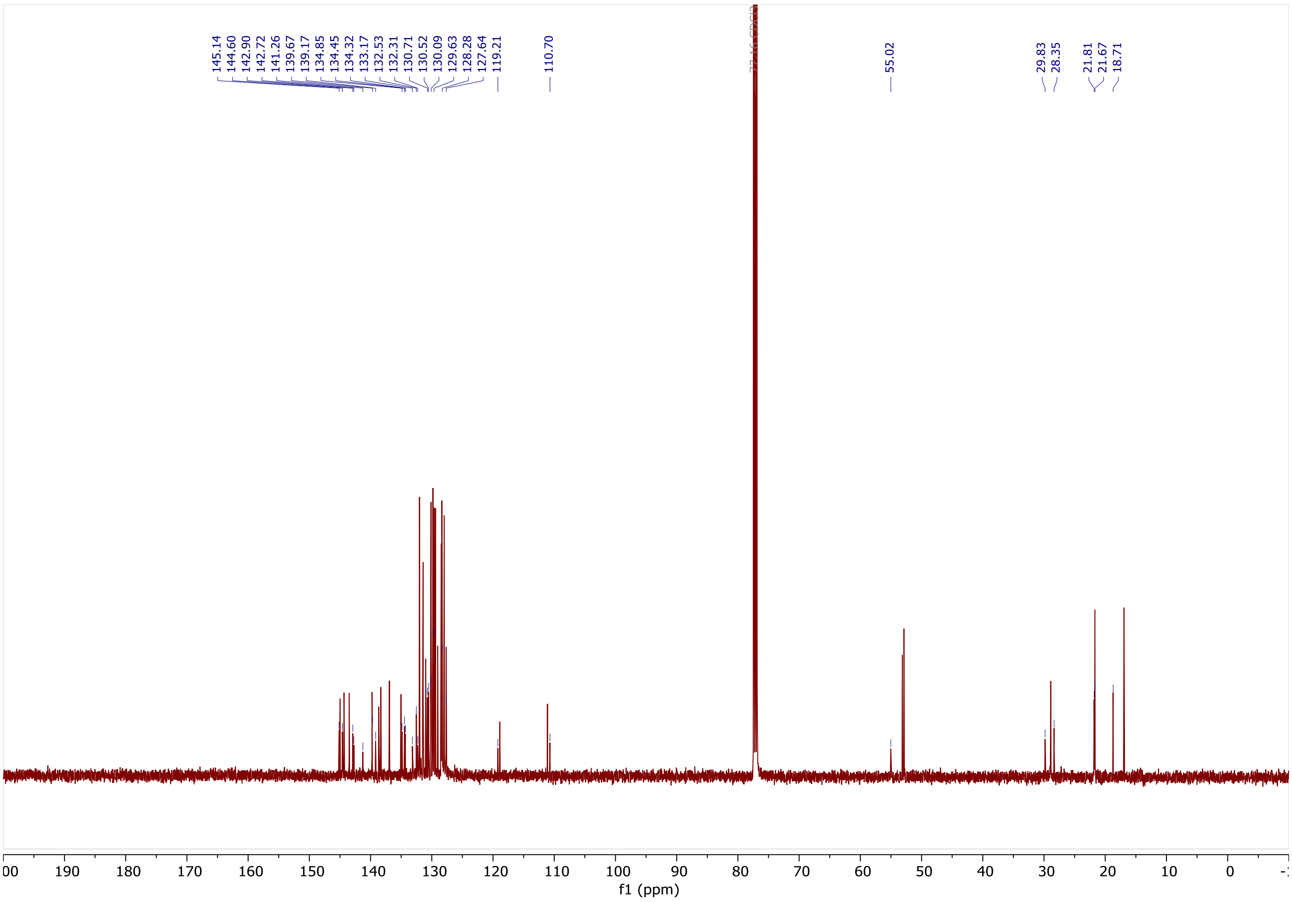

2ap ***N*-Benzyl-*N*-(5-(3-methoxyphenyl)-4-methyl-1-tosylindolin-7-yl)-4-methylbenzenesulfonamide & *N*-benzyl-*N*-(6-(3-methoxyphenyl)-4-methyl-1-tosylindolin-7-yl)-4-methylbenzenesulfonamide**

^{1}H NMR (500 MHz, $CDCl_3$)
^{13}C NMR (126 MHz, $CDCl_3$)

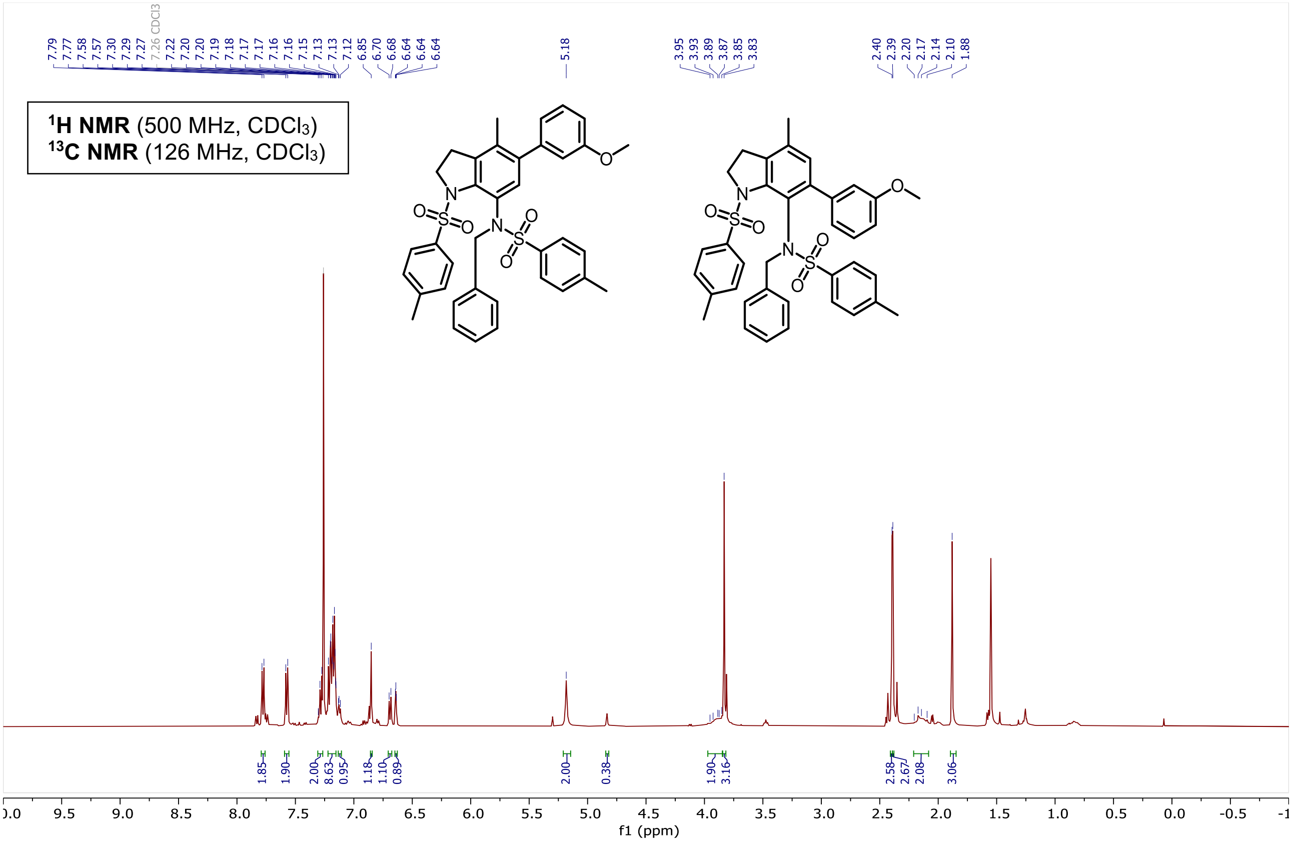

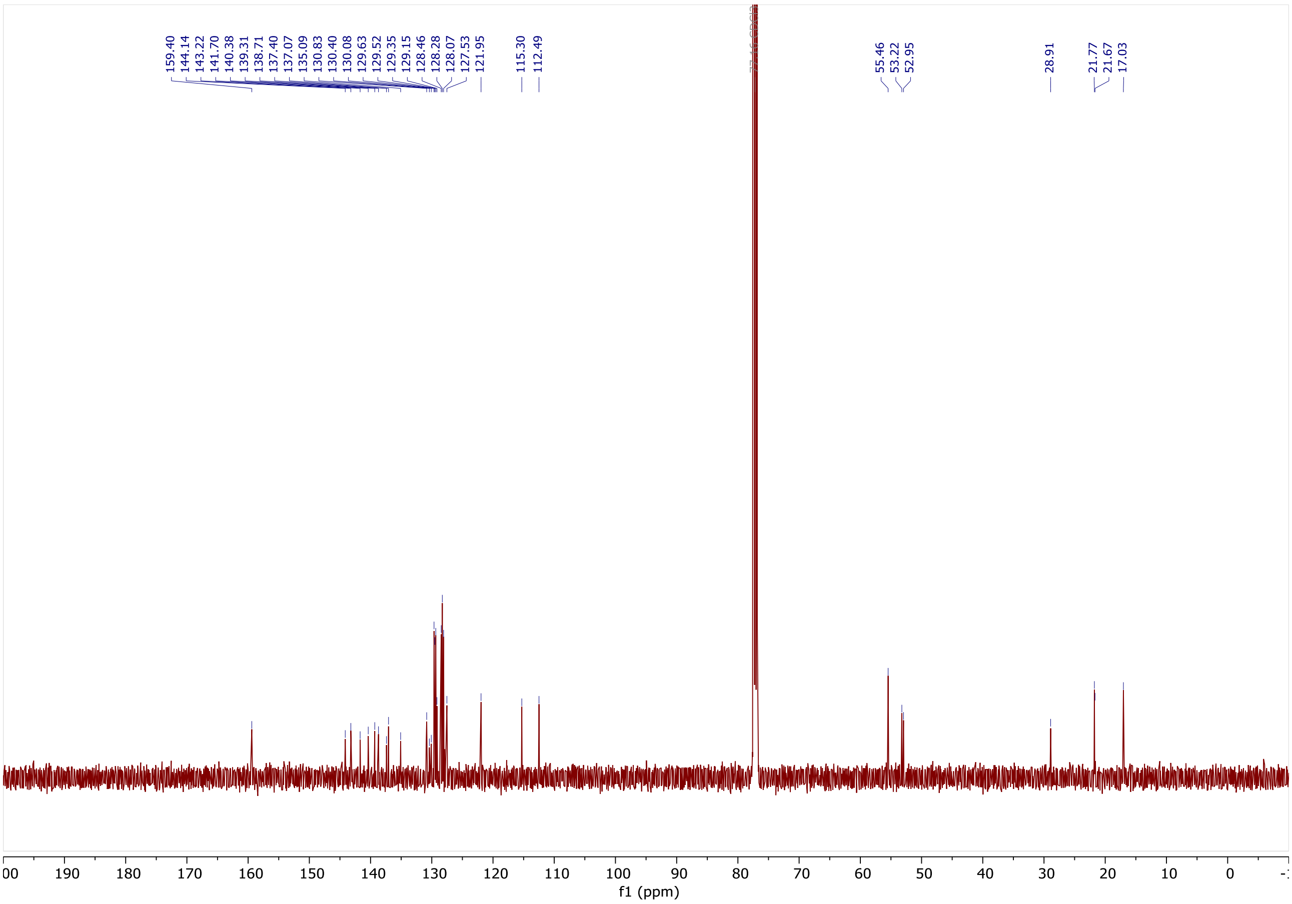

2aq ***N*-Benzyl-*N*-(5-(2-methoxyphenyl)-4-methyl-1-tosylindolin-7-yl)-4-methylbenzenesulfonamide and *N*-benzyl-*N*-(6-(2-methoxyphenyl)-4-methyl-1-tosylindolin-7-yl)-4-methylbenzenesulfonamide**

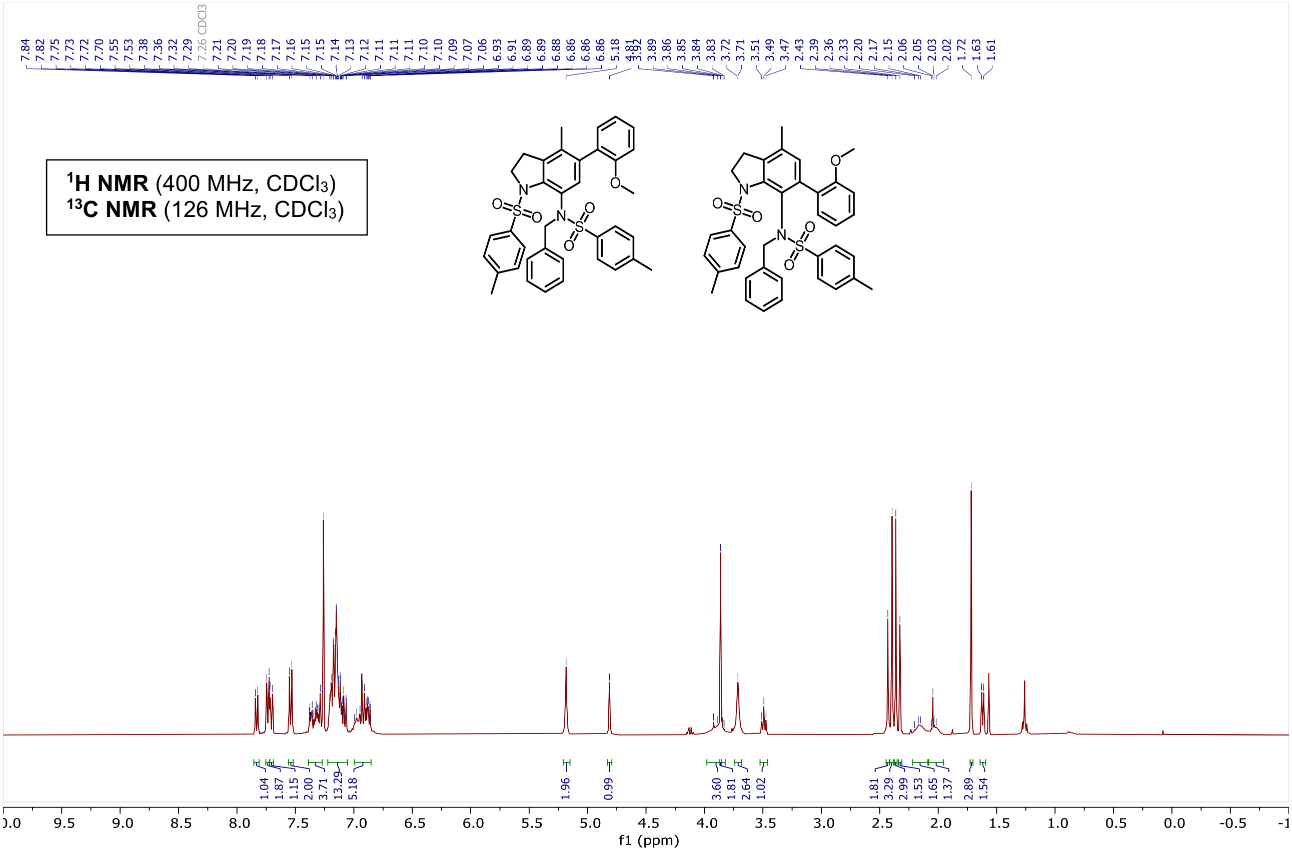

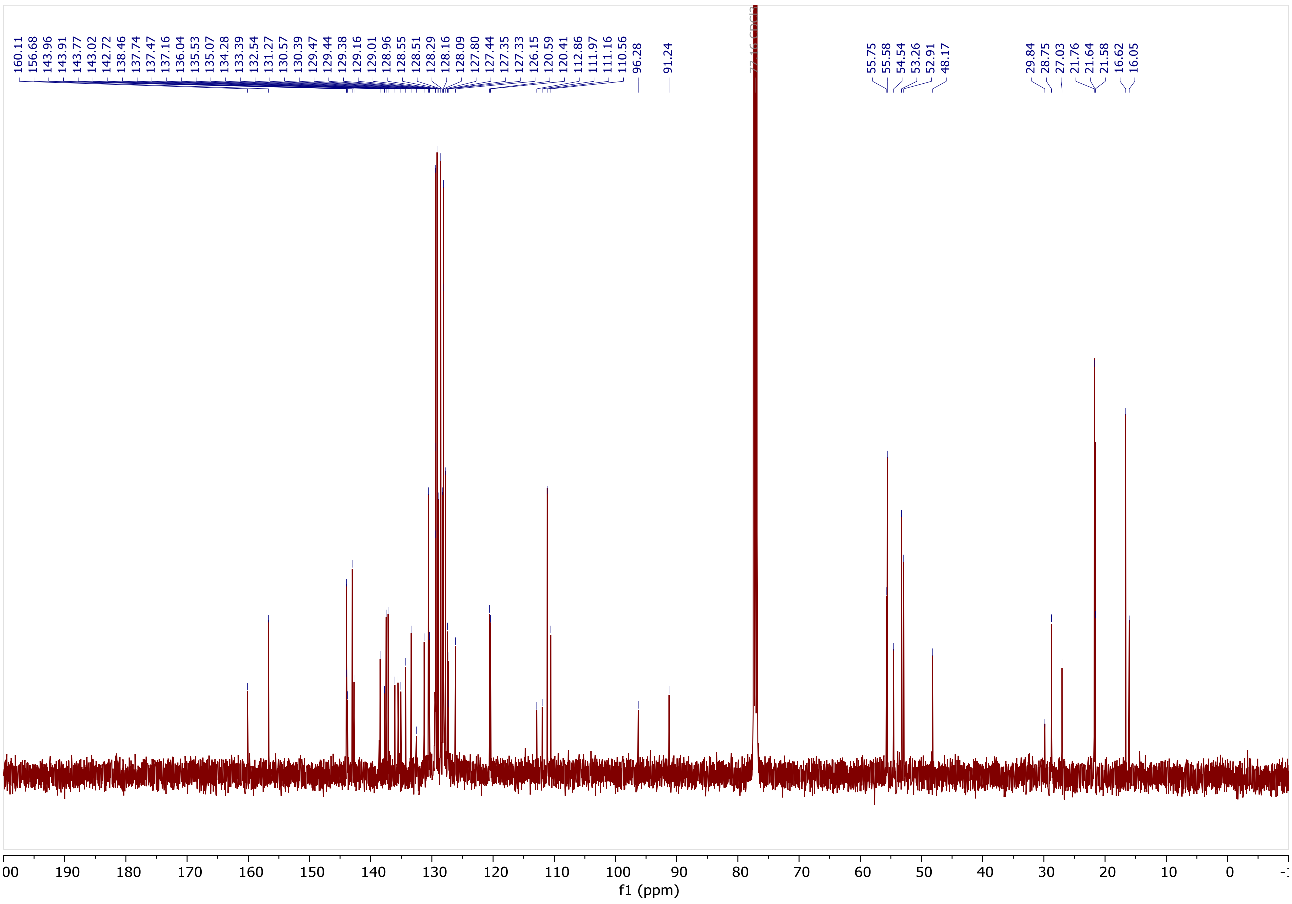

2ar *N*-Benzyl-*N*-(5,6-bis(methoxymethyl)-4-methyl-1-tosylindolin-7-yl)-4-methylbenzenesulfonamide

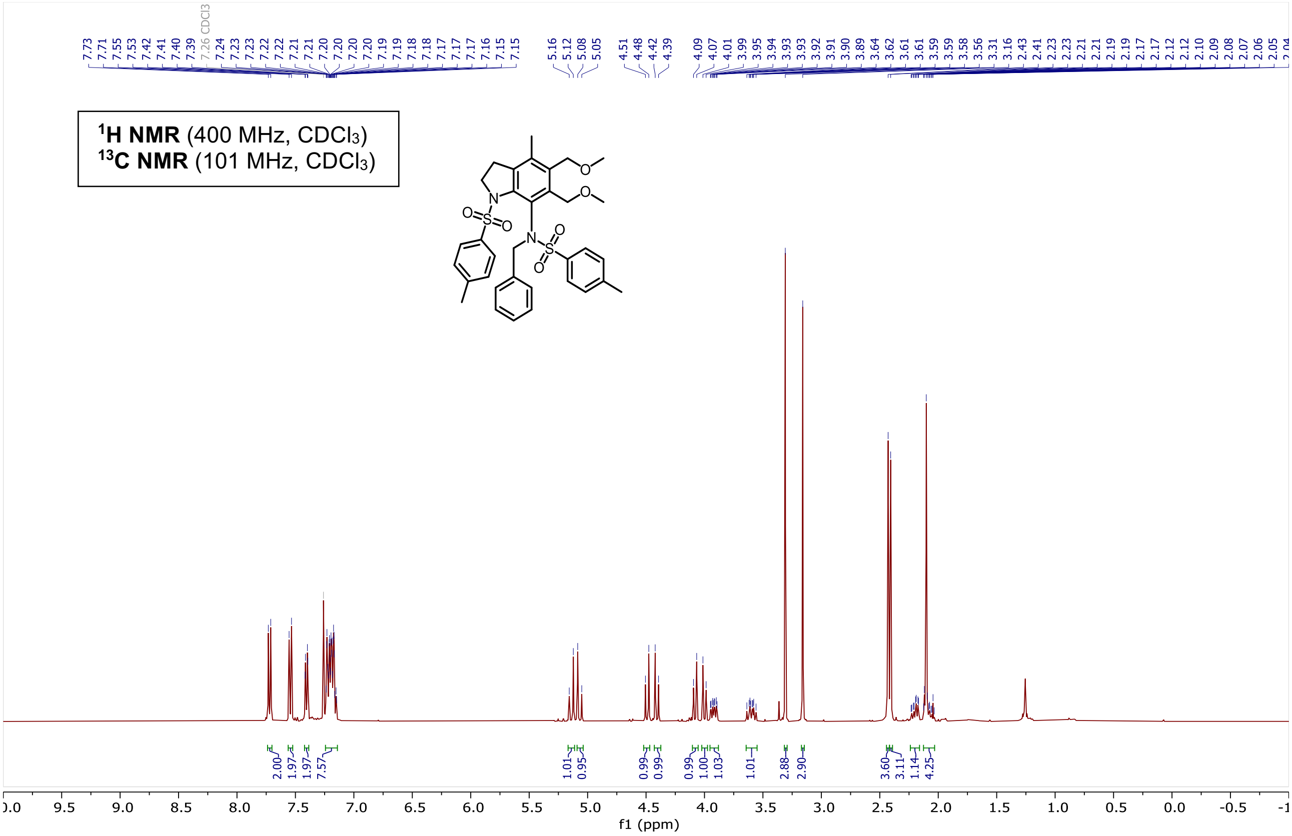

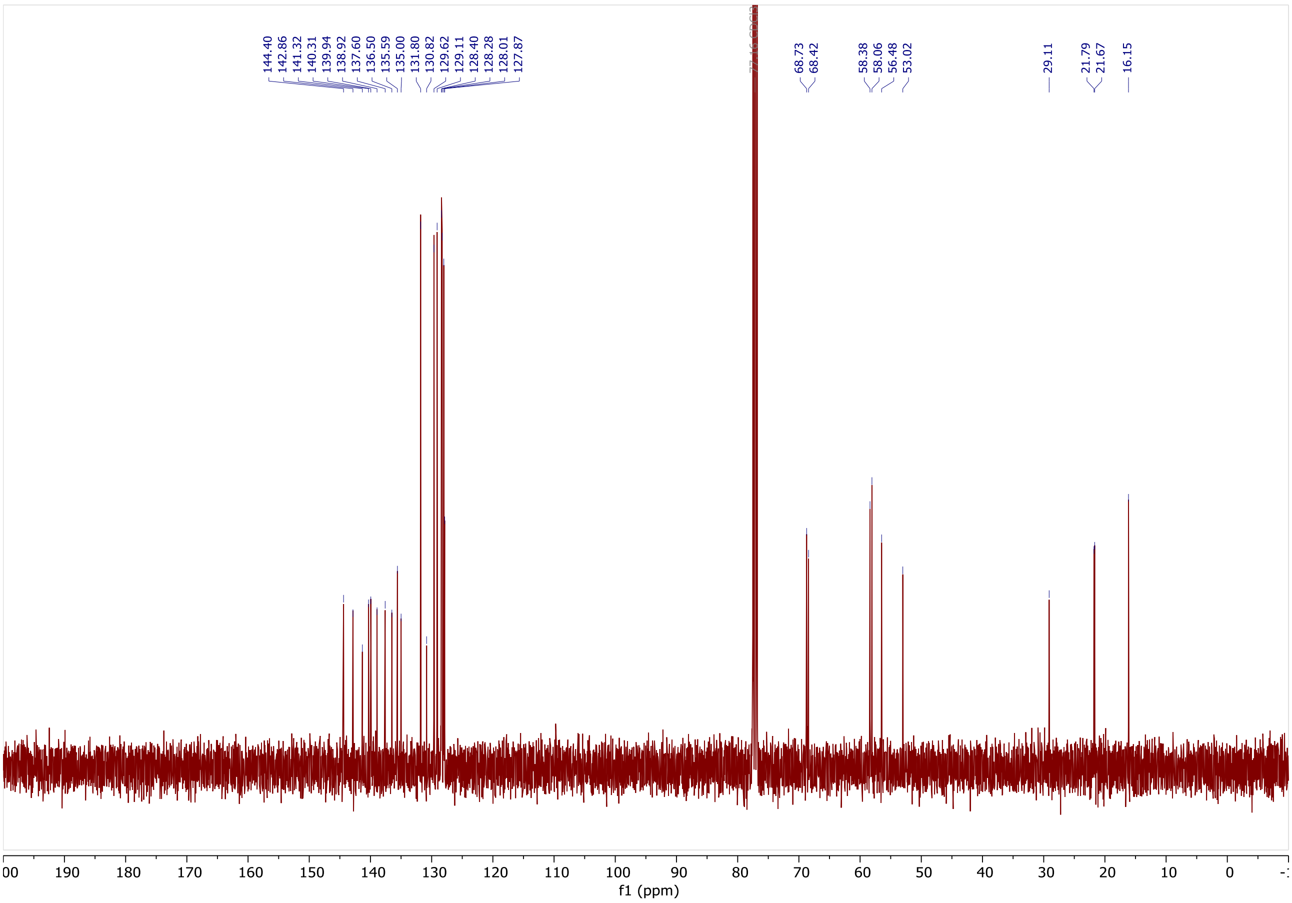

2as *N*-Benzyl-*N*-(5,6-diethyl-4-methyl-1-tosylindolin-7-yl)-4-methylbenzenesulfonamide

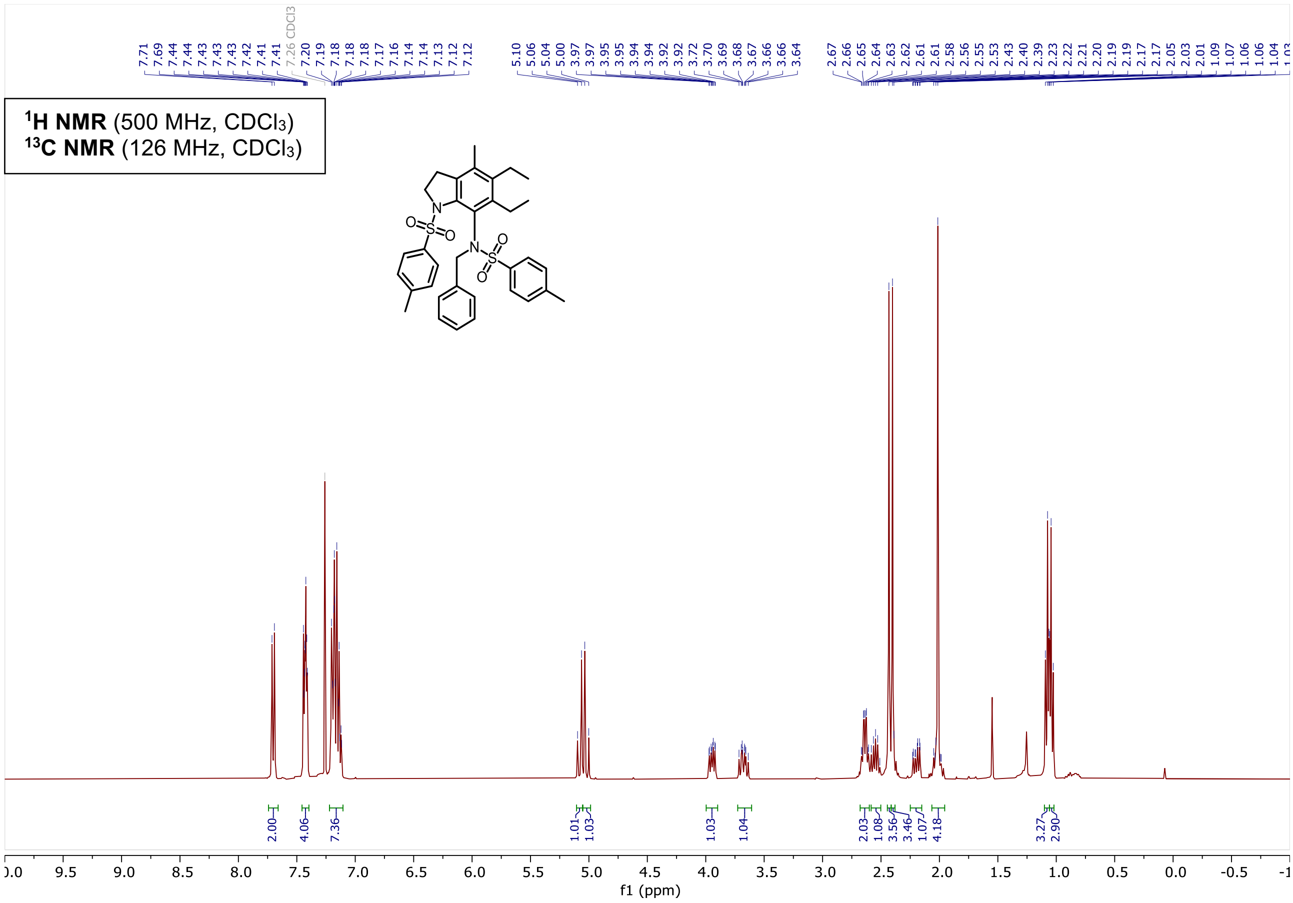

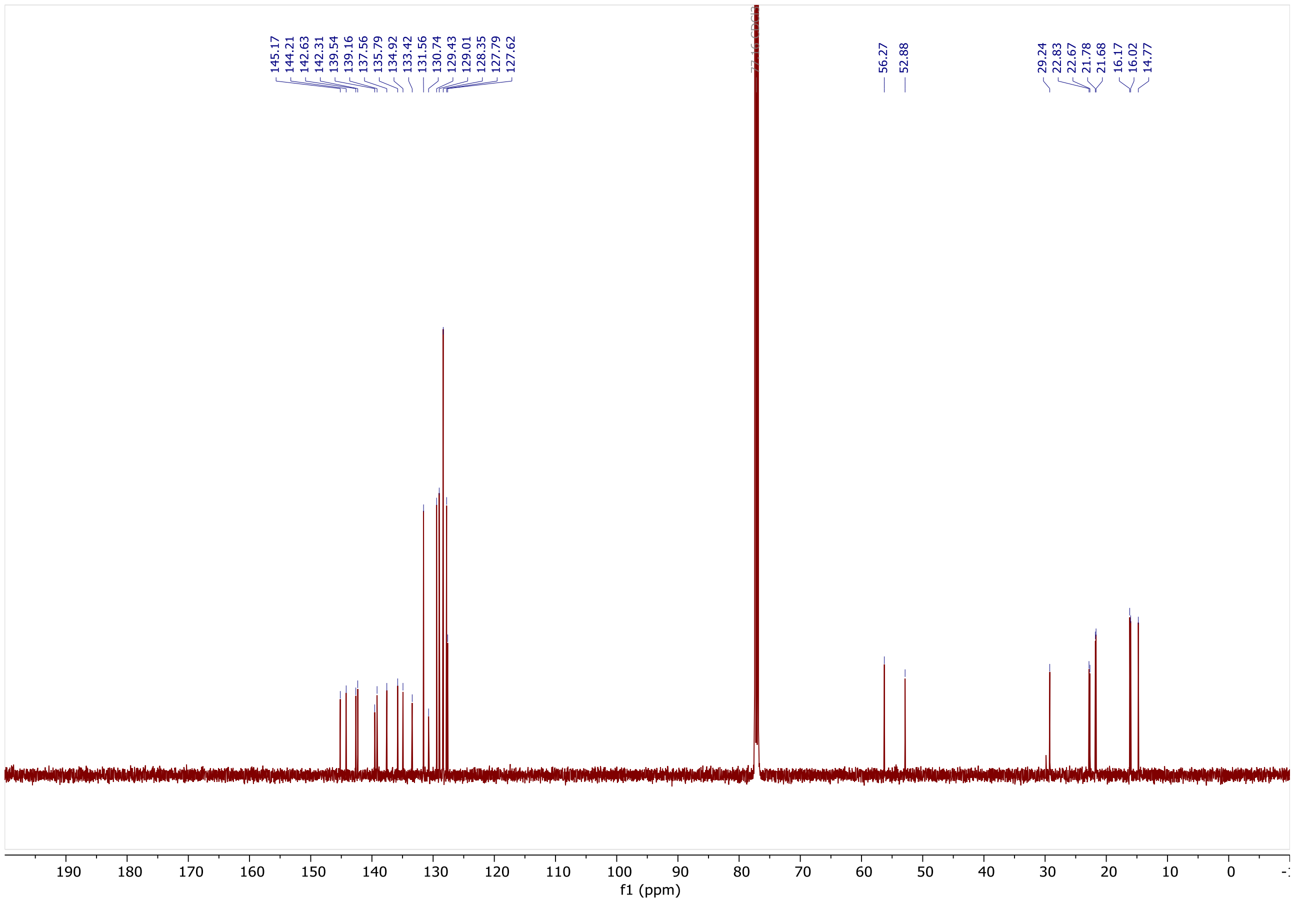

2at ***N*-Benzyl-*N*-(5-(hydroxymethyl)-4-methyl-1-tosylindolin-7-yl)-4-methylbenzenesulfonamide**

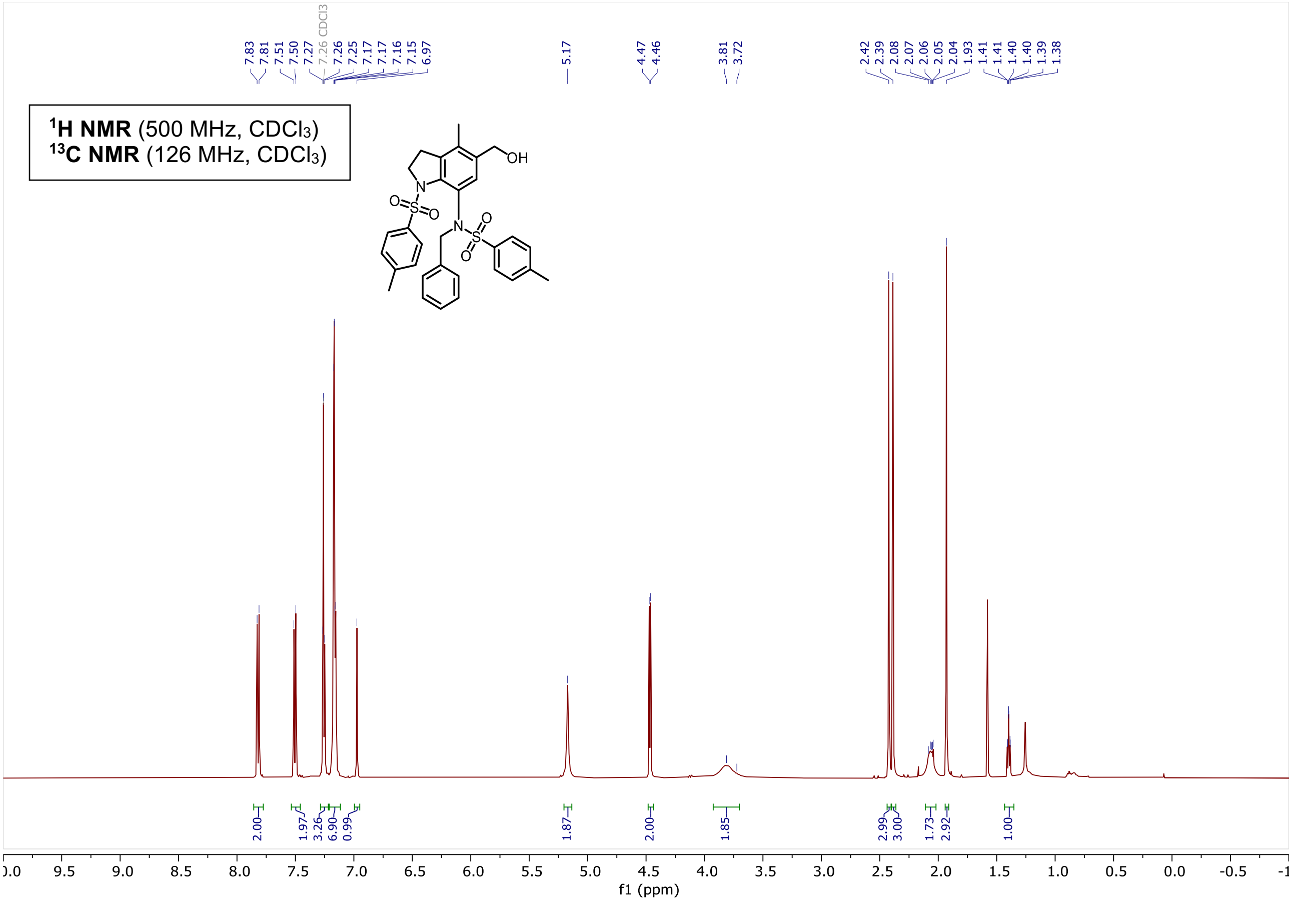

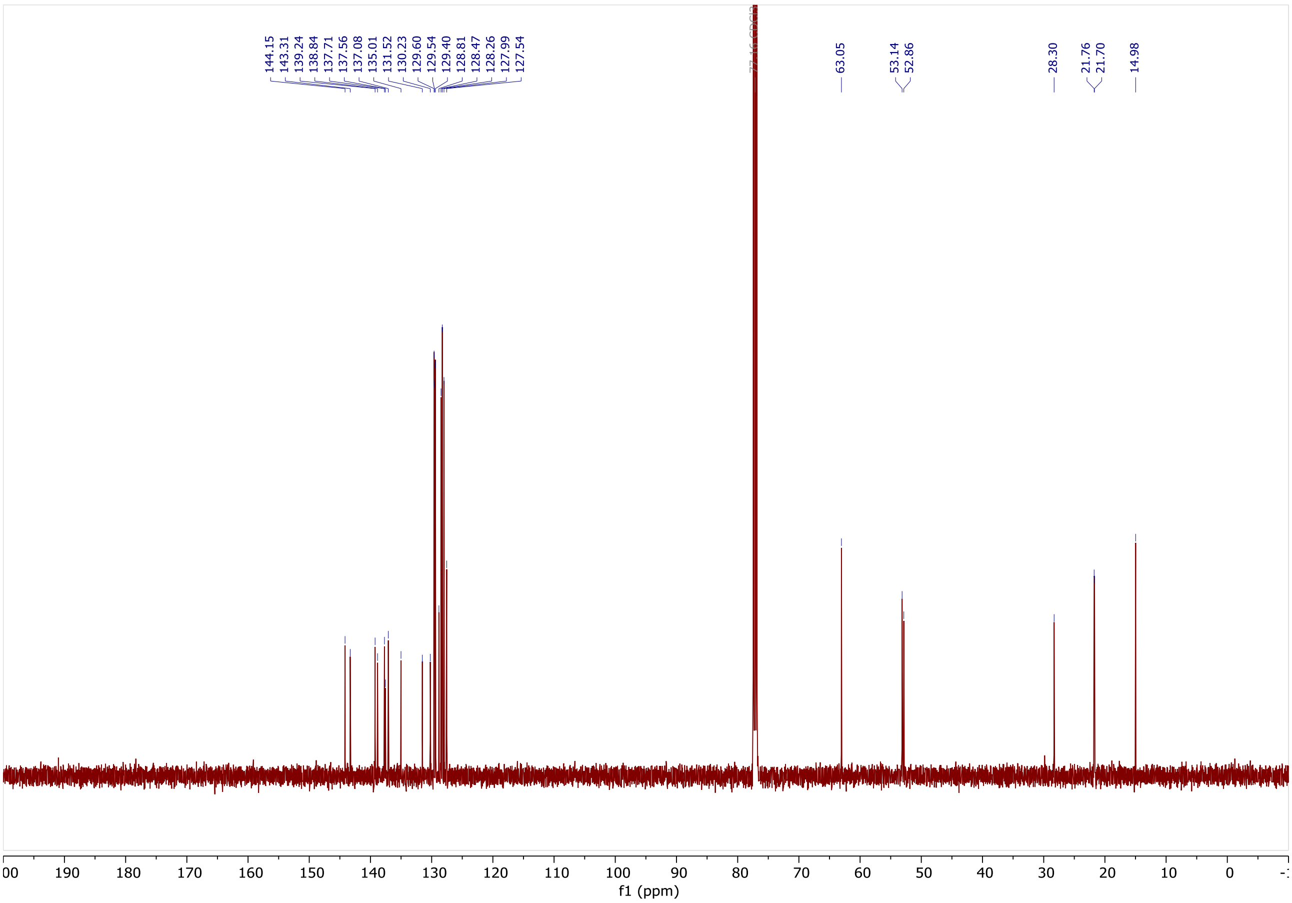

2at' ***N*-Benzyl-*N*-(6-(hydroxymethyl)-4-methyl-1-tosylindolin-7-yl)-4-methylbenzenesulfonamide**

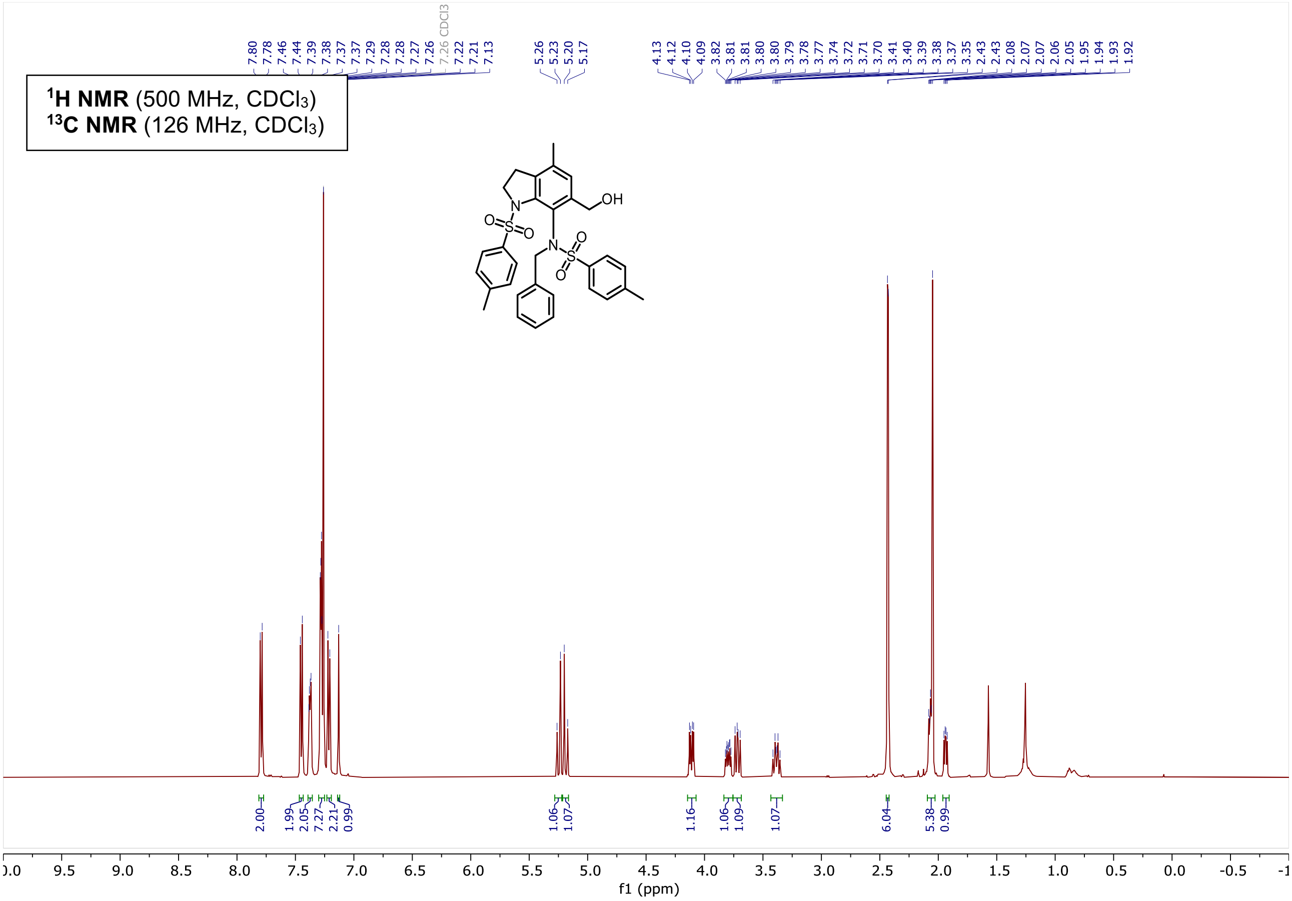

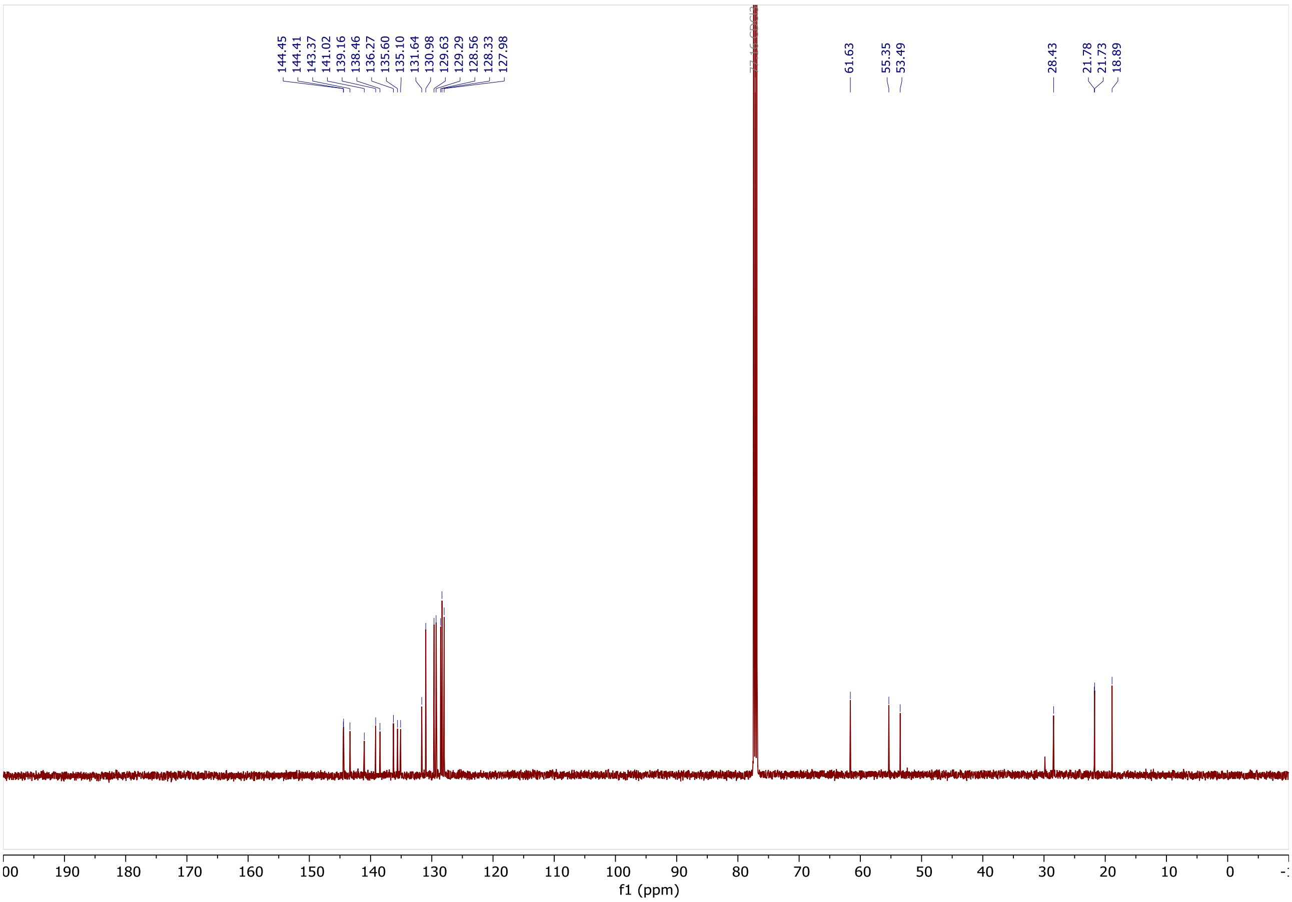

2au ***N*-Benzyl-*N*-(6-ethyl-5-(hydroxymethyl)-4-methyl-1-tosylindolin-7-yl)-4-methylbenzenesulfonamide**

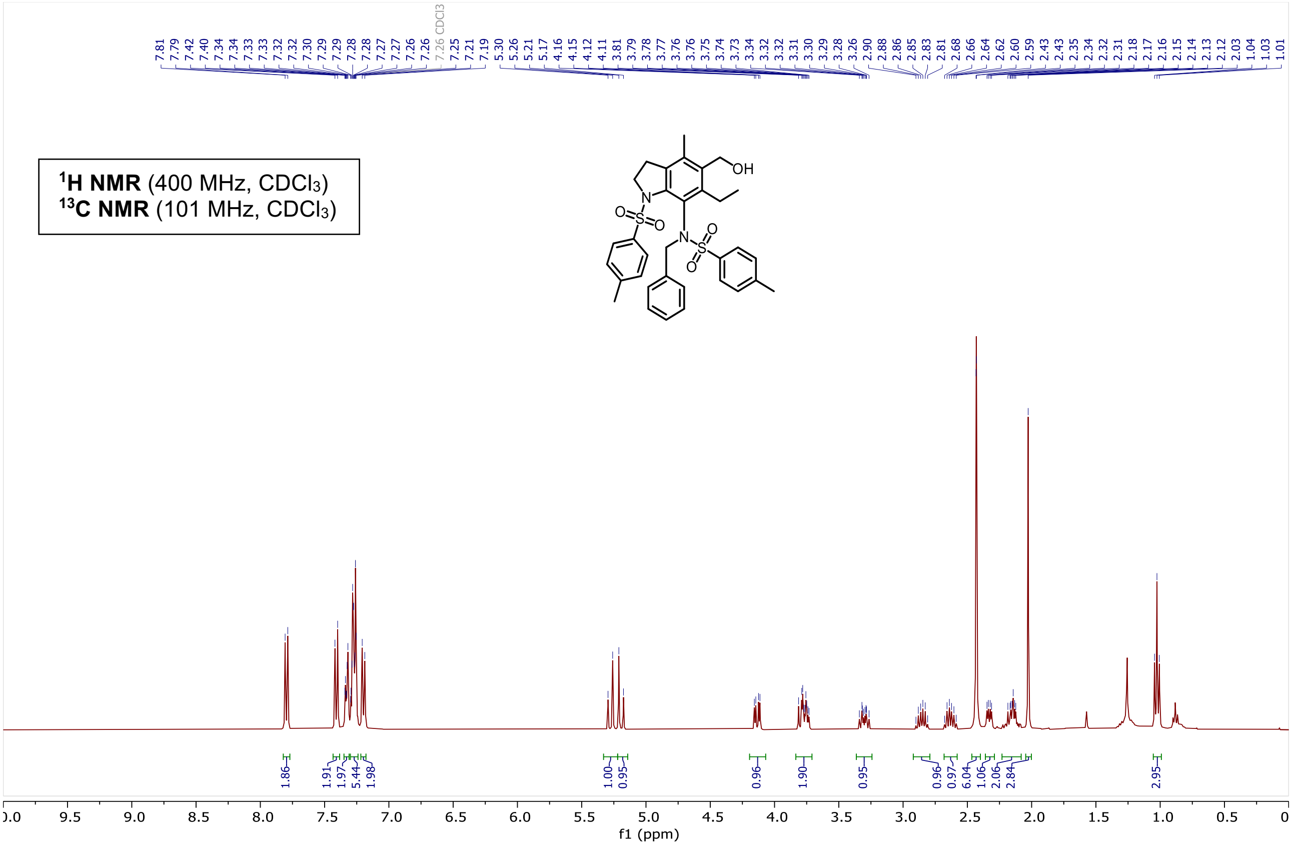

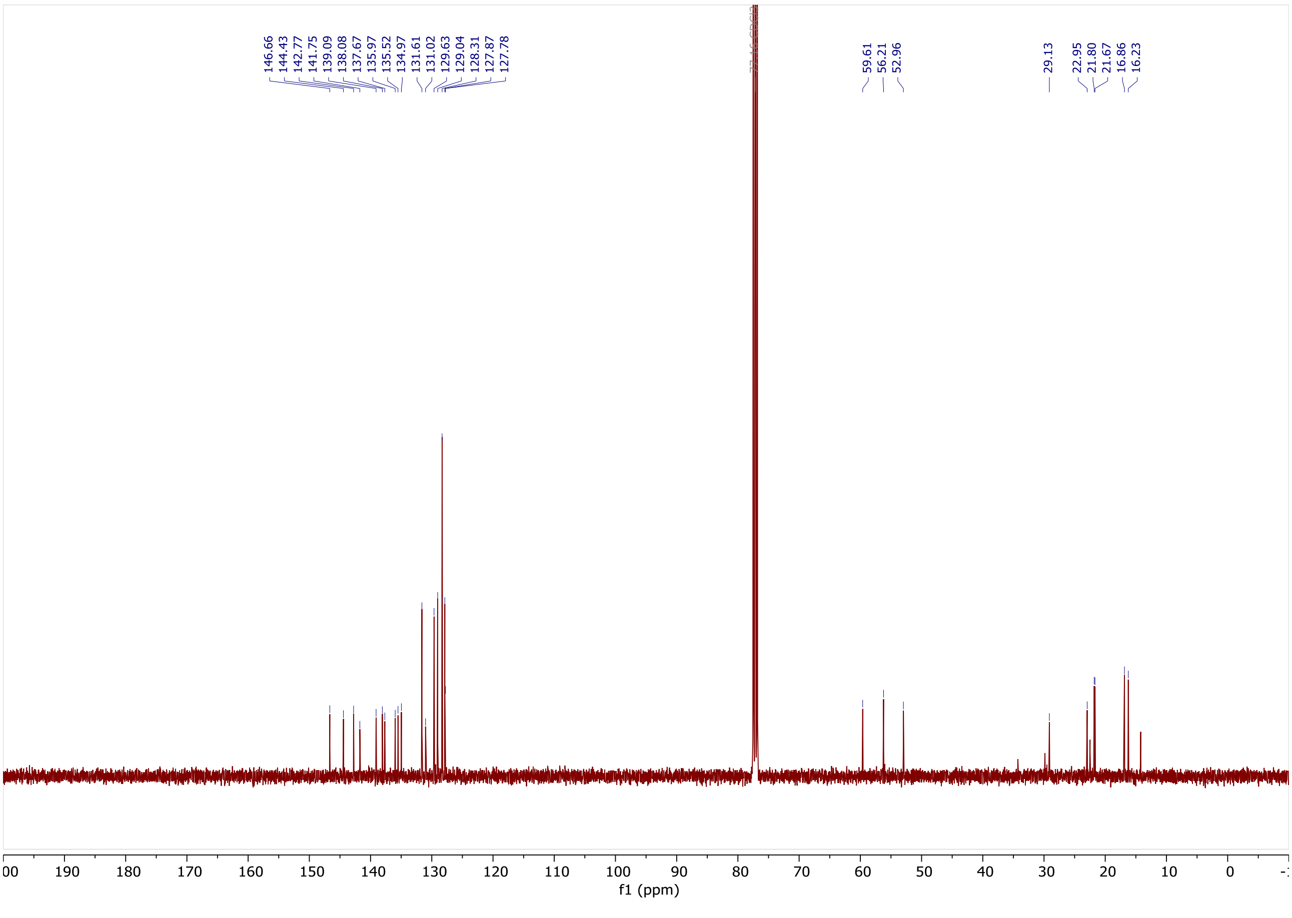

2au' ***N*-Benzyl-*N*-(5-ethyl-6-(hydroxymethyl)-4-methyl-1-tosylindolin-7-yl)-4-methylbenzenesulfonamide**

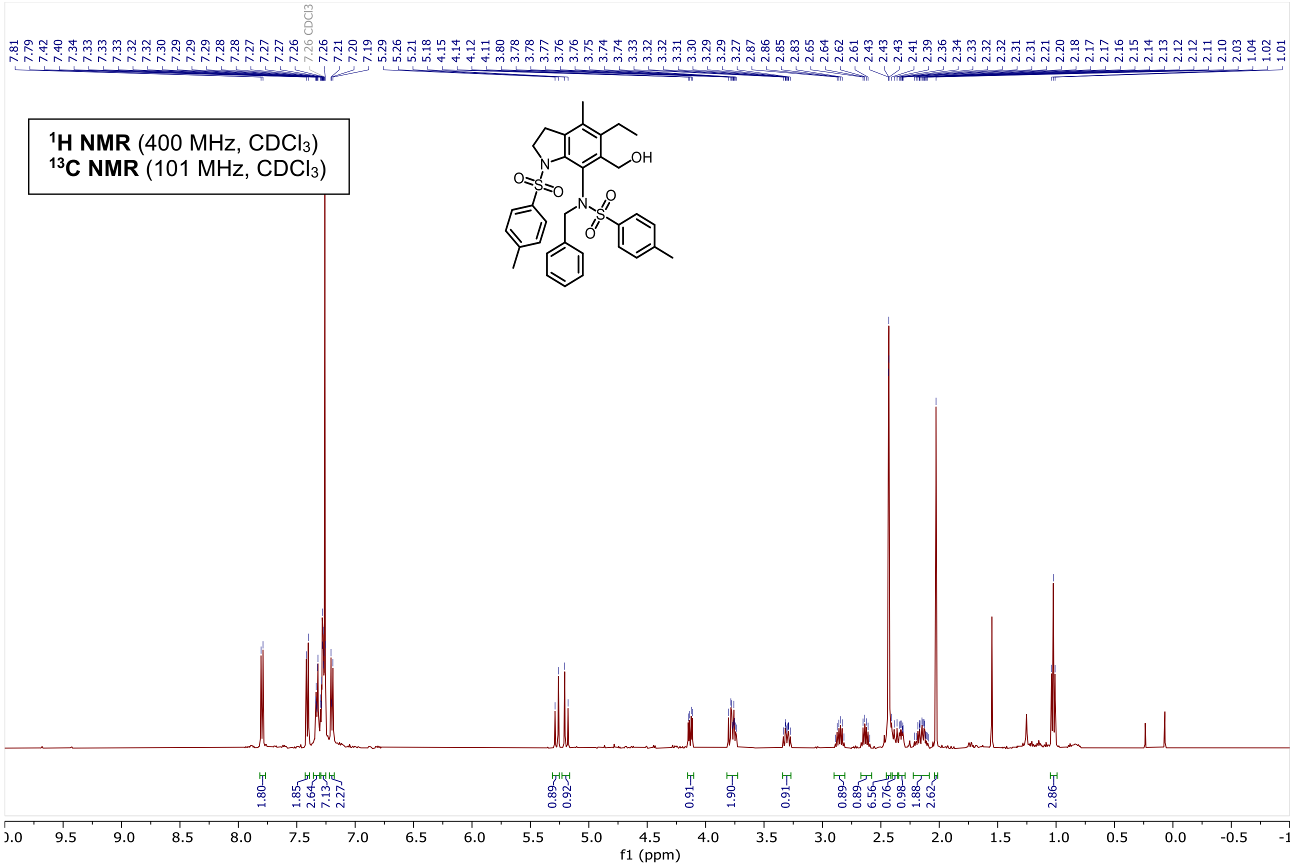

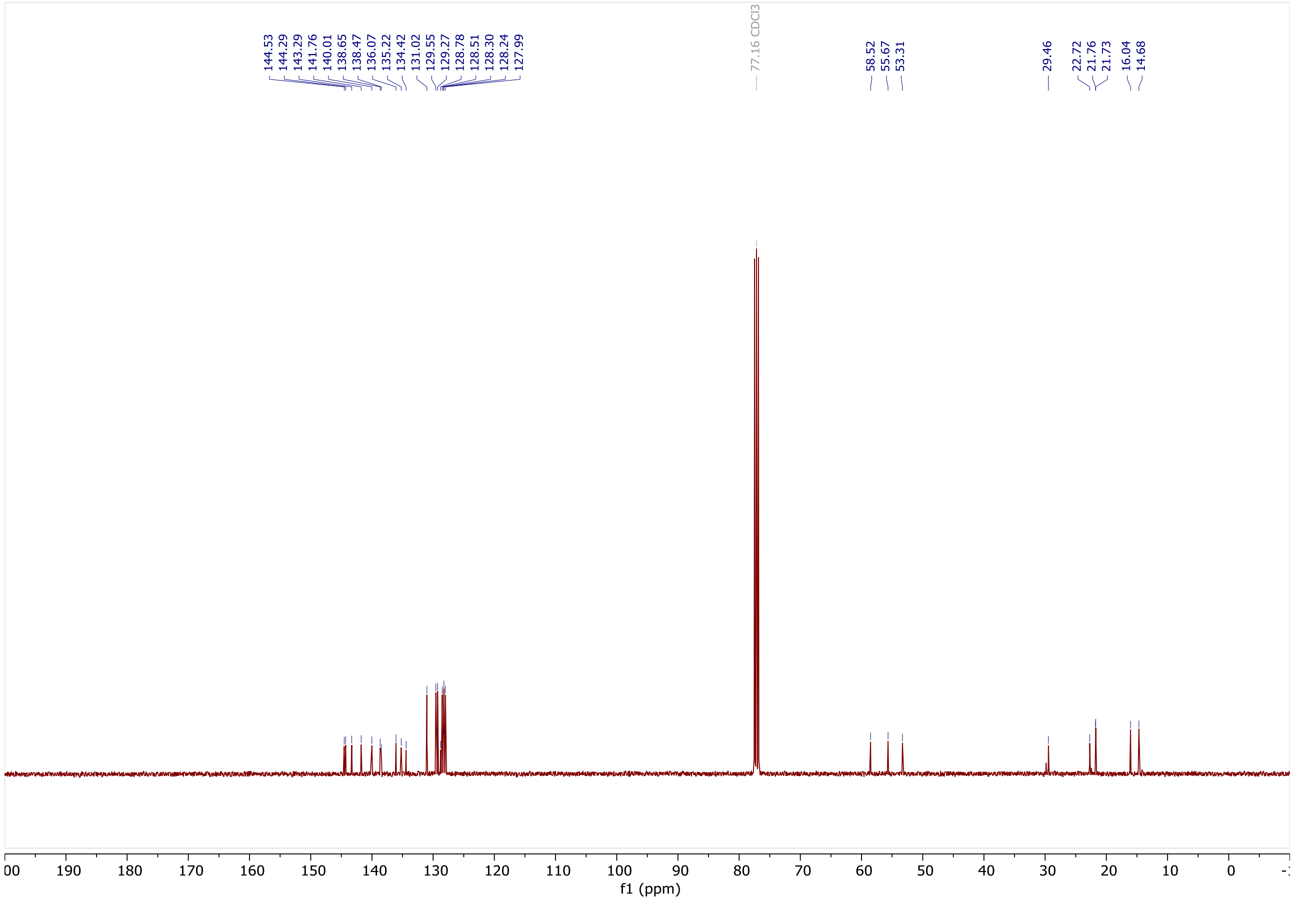

2av ***N*-Benzyl-*N*-(6-butyl-5-(1-hydroxyethyl)-4-methyl-1-tosylindolin-7-yl)-4-methylbenzenesulfonamide & 2av’ *N*-Benzyl-*N*-(5-butyl-6-(1-hydroxyethyl)-4-methyl-1-tosylindolin-7-yl)-4-methylbenzenesulfonamide**

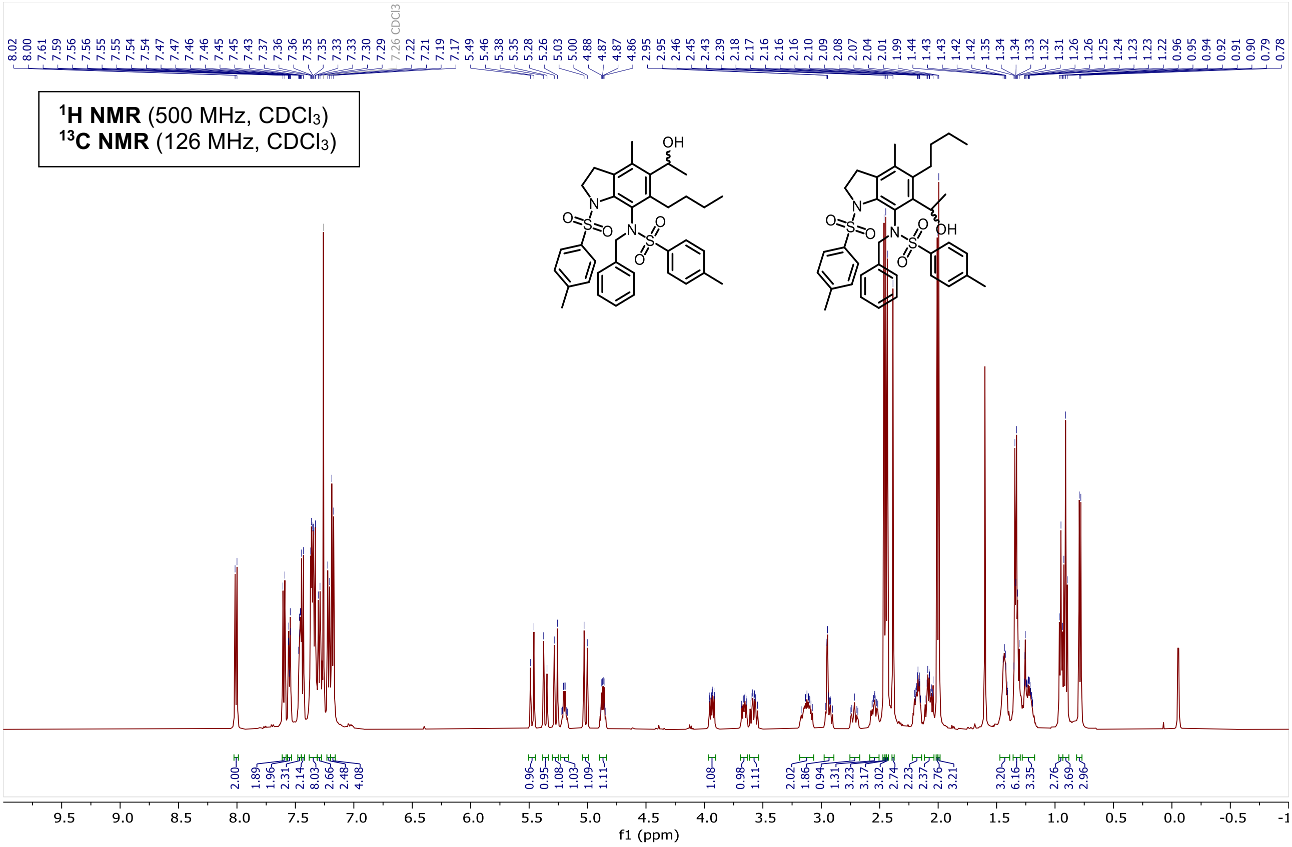

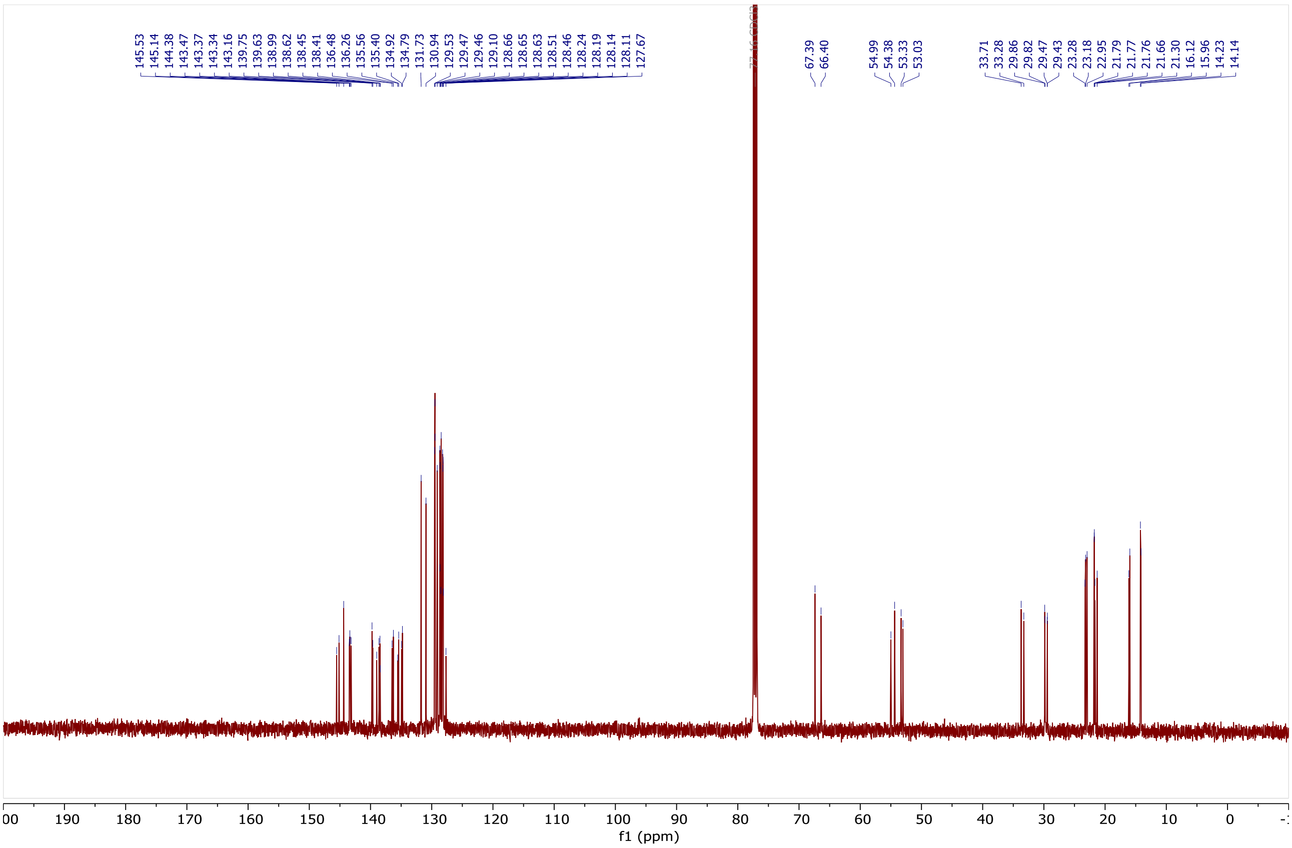

2ba ***N*-Butyl-*N*-(4-(4-fluorophenyl)-5,6-bis(hydroxymethyl)-1-tosylindolin-7-yl)-4-methylbenzenesulfonamide**

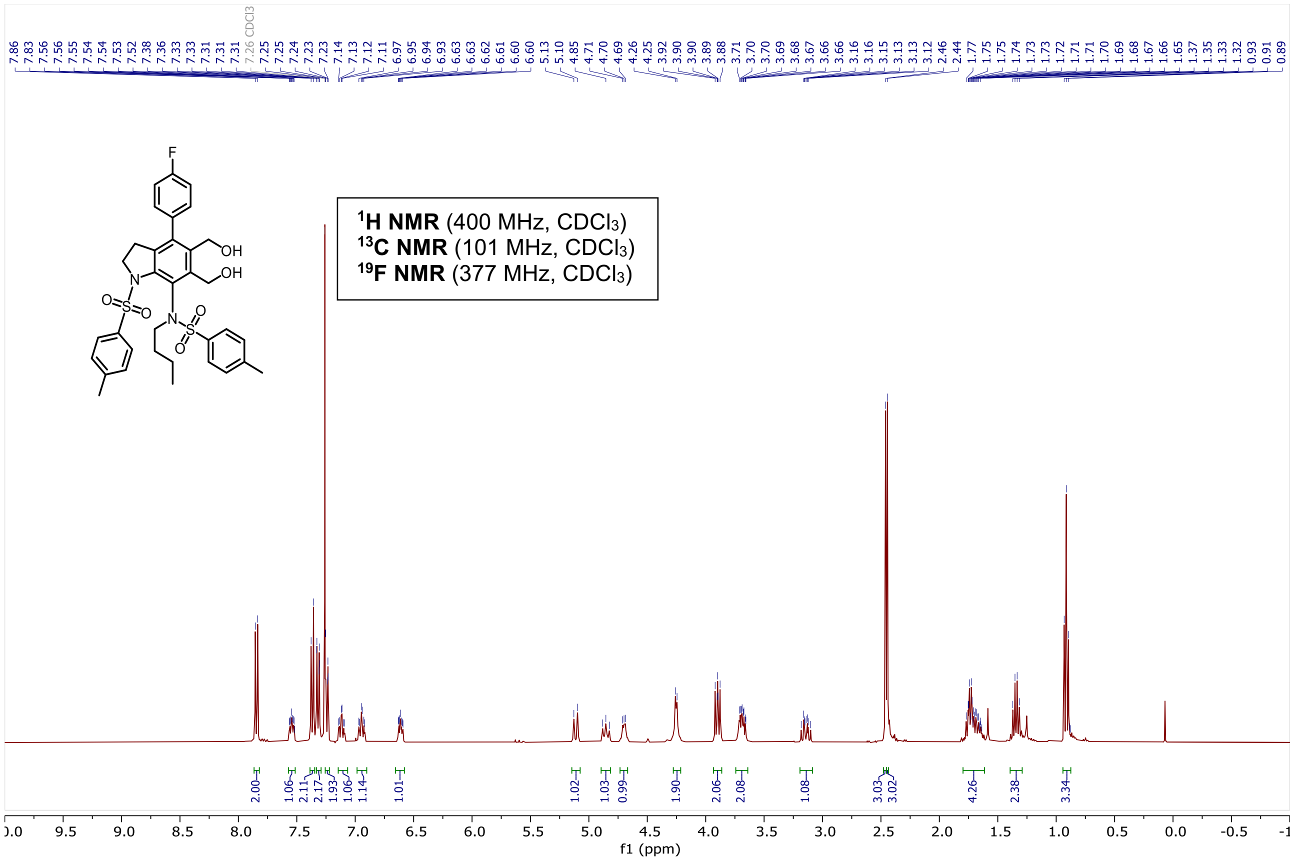

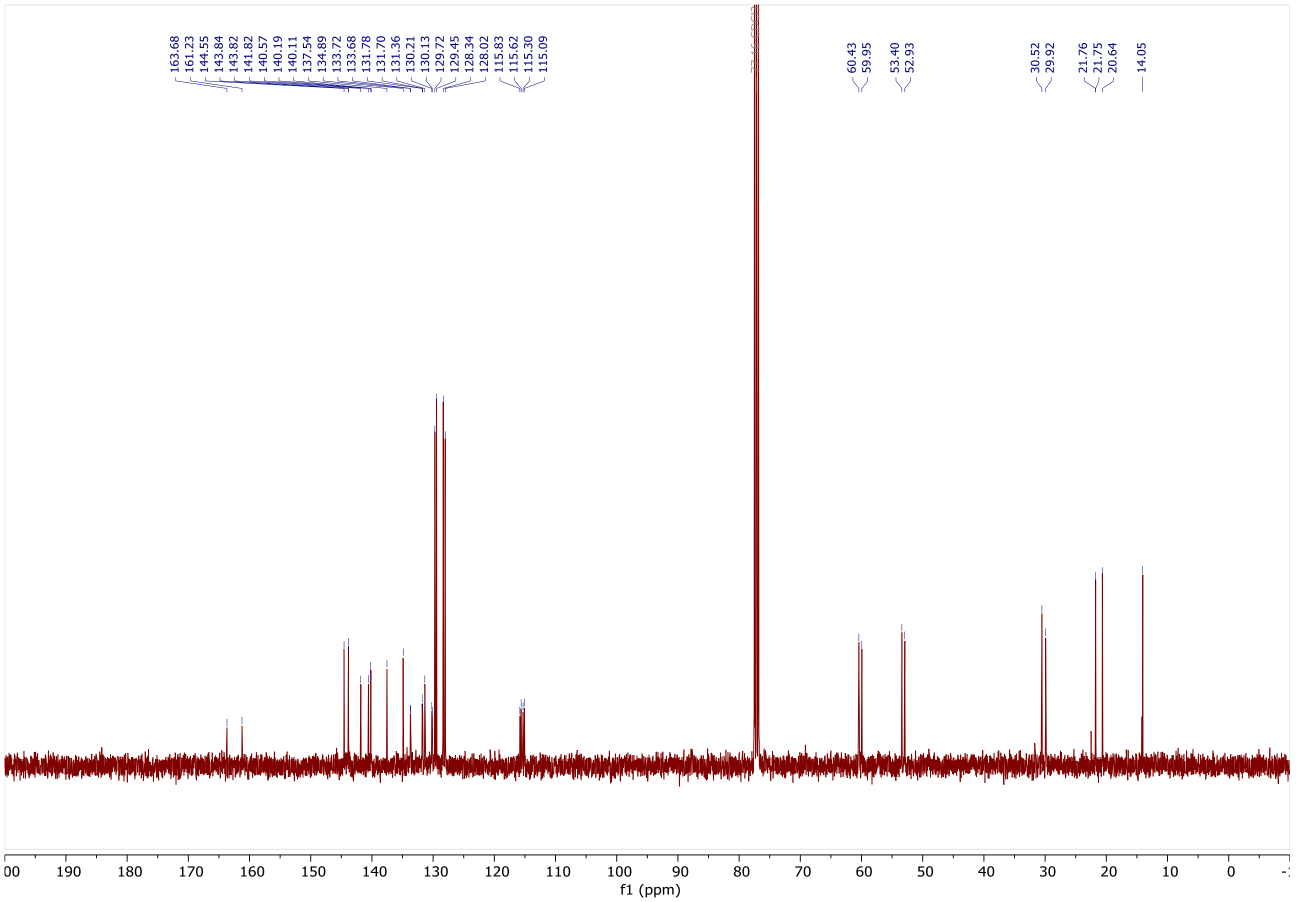

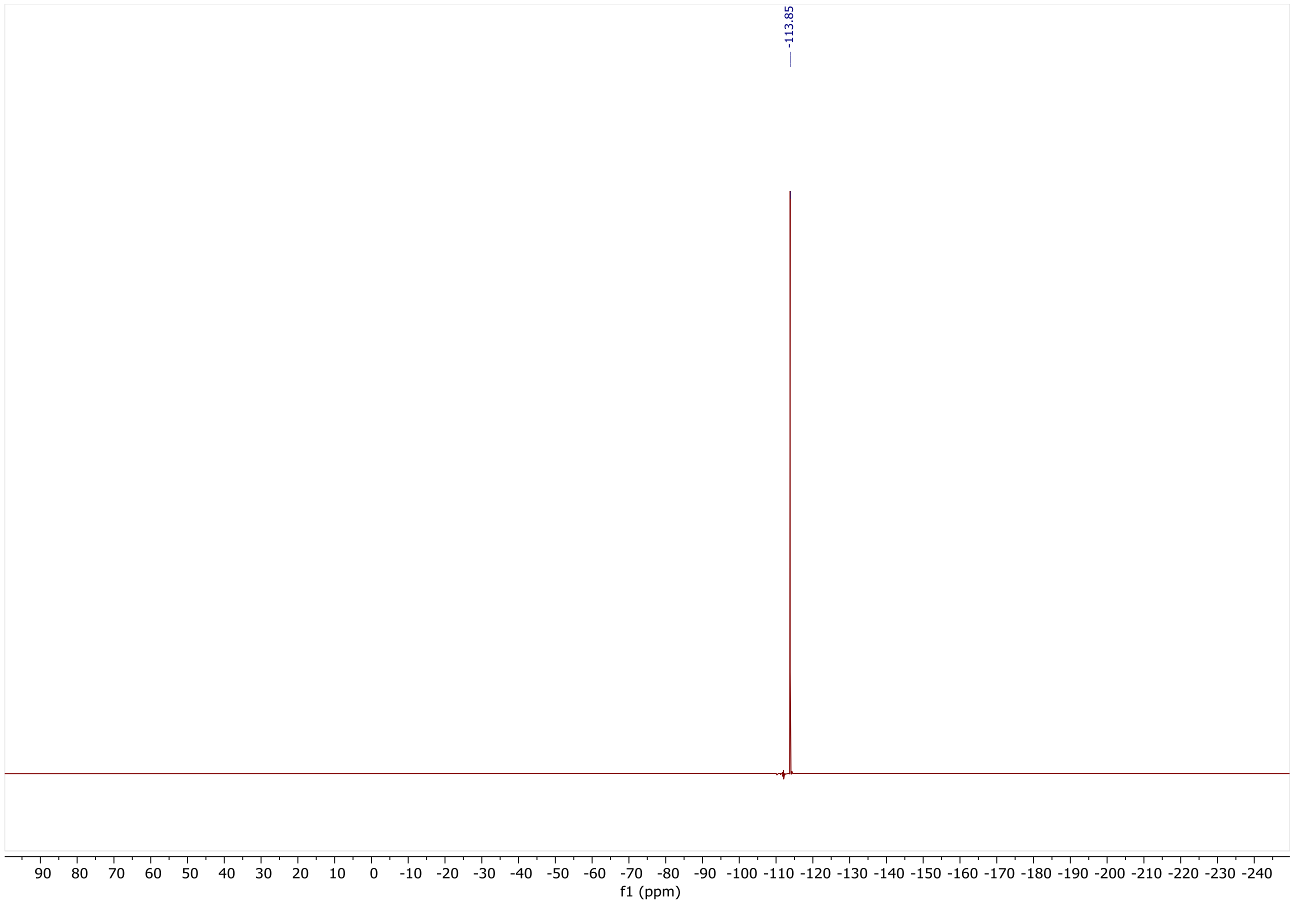
-113.85
90 80 70 60 50 40 30 20 10 0 -10 -20 -30 -40 -50 -60 -70 -80 -90 -100 -110 -120 -130 -140 -150 -160 -170 -180 -190 -200 -210 -220 -230 -240
f1 (ppm)

2cb ***N*-Butyl-*N*-(5-(4-(dimethylamino)phenyl)-4-phenyl-1-tosylindolin-7-yl)-4-methylbenzenesulfonamide** and ***N*-butyl-*N*-(6-(4-(dimethylamino)phenyl)-4-phenyl-1-tosylindolin-7-yl)-4-methylbenzenesulfonamide**

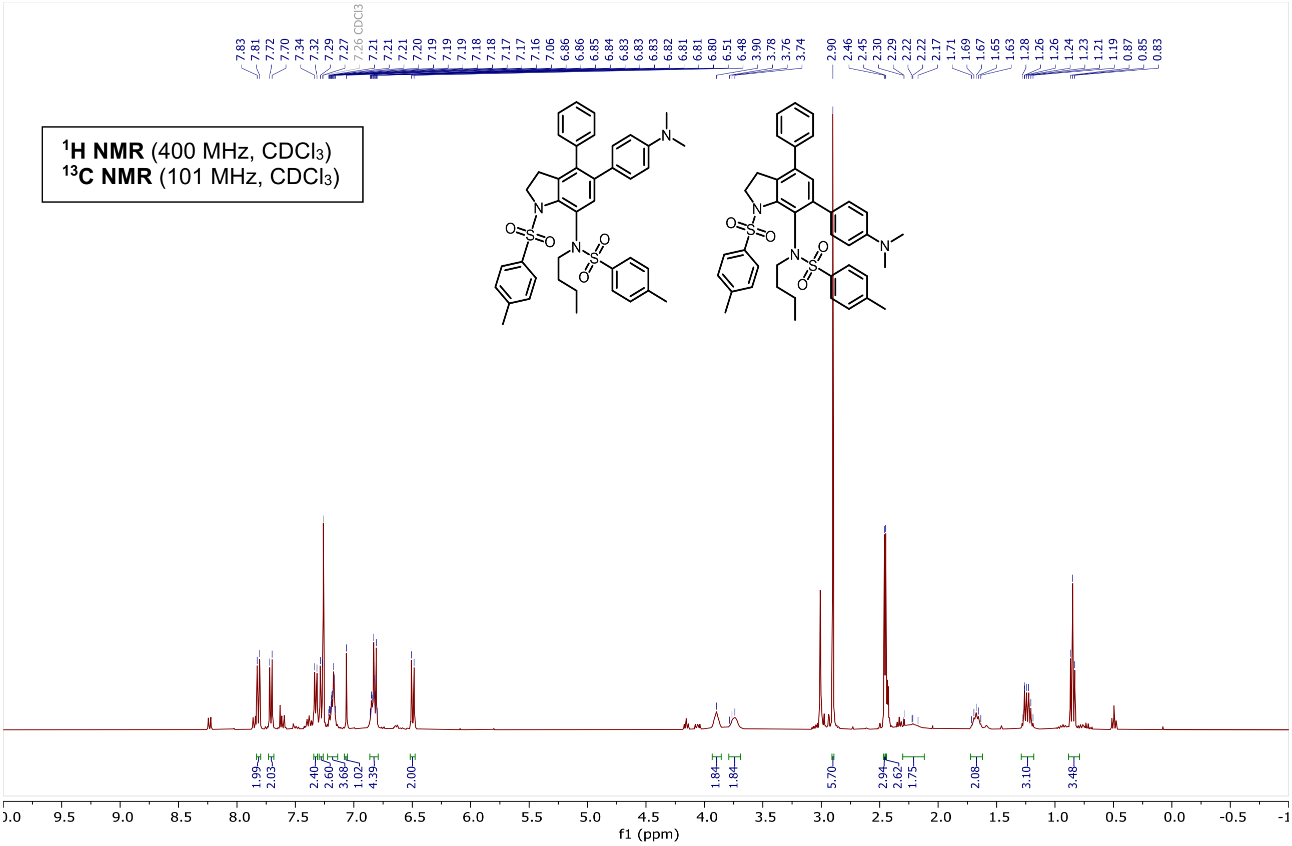

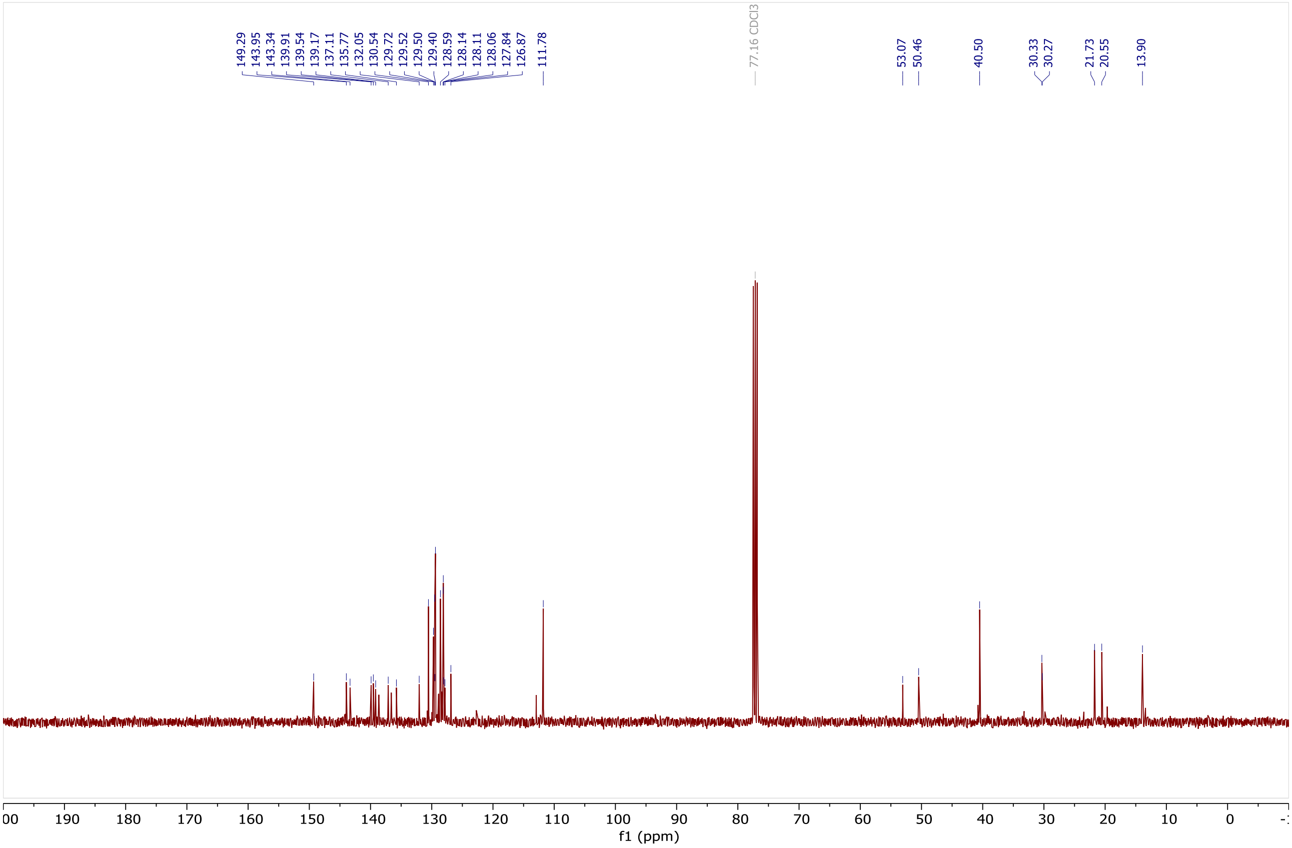

2aw ***N*-Benzyl-4-methyl-*N*-(4-methyl-1-tosyl-6-(trimethylsilyl)indolin-7-yl)benzenesulfonamide** and ***N*-benzyl-4-methyl-*N*-(4-methyl-1-tosyl-5-(trimethylsilyl)indolin-7-yl)benzenesulfonamide**

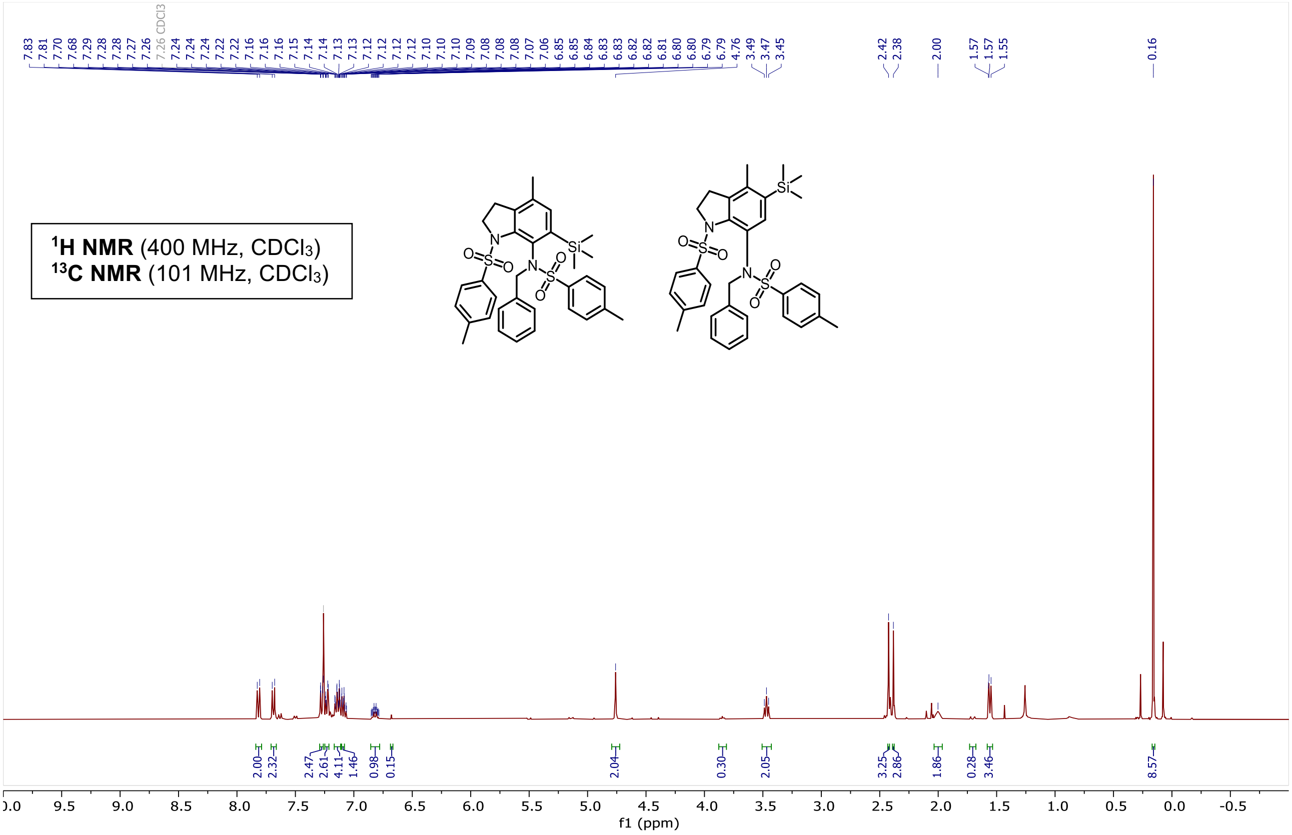

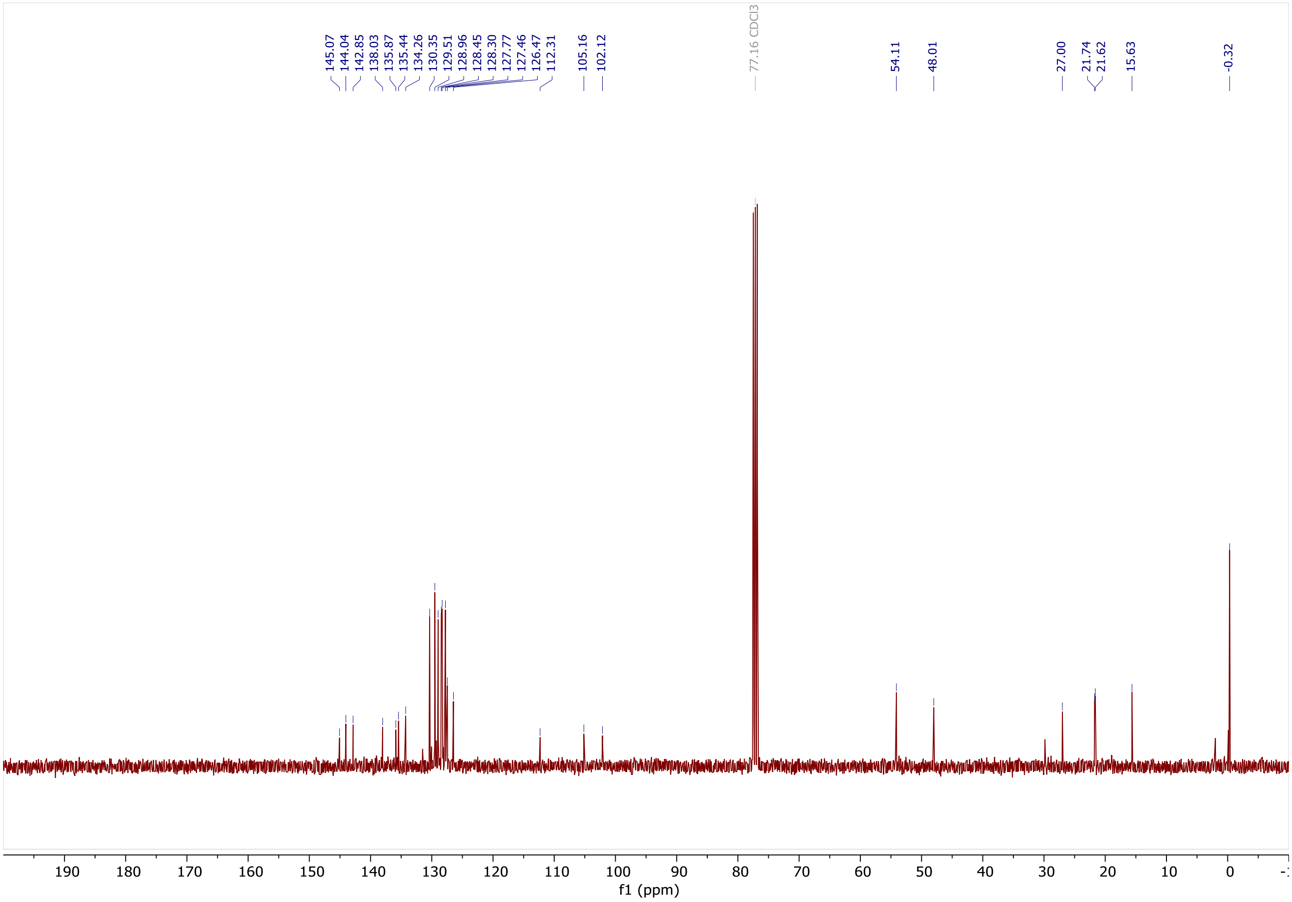

2ax ***N*-Benzyl-4-methyl-*N*-(4-methyl-1-tosyl-6-(triethylsilyl)indolin-7-yl)benzenesulfonamide**

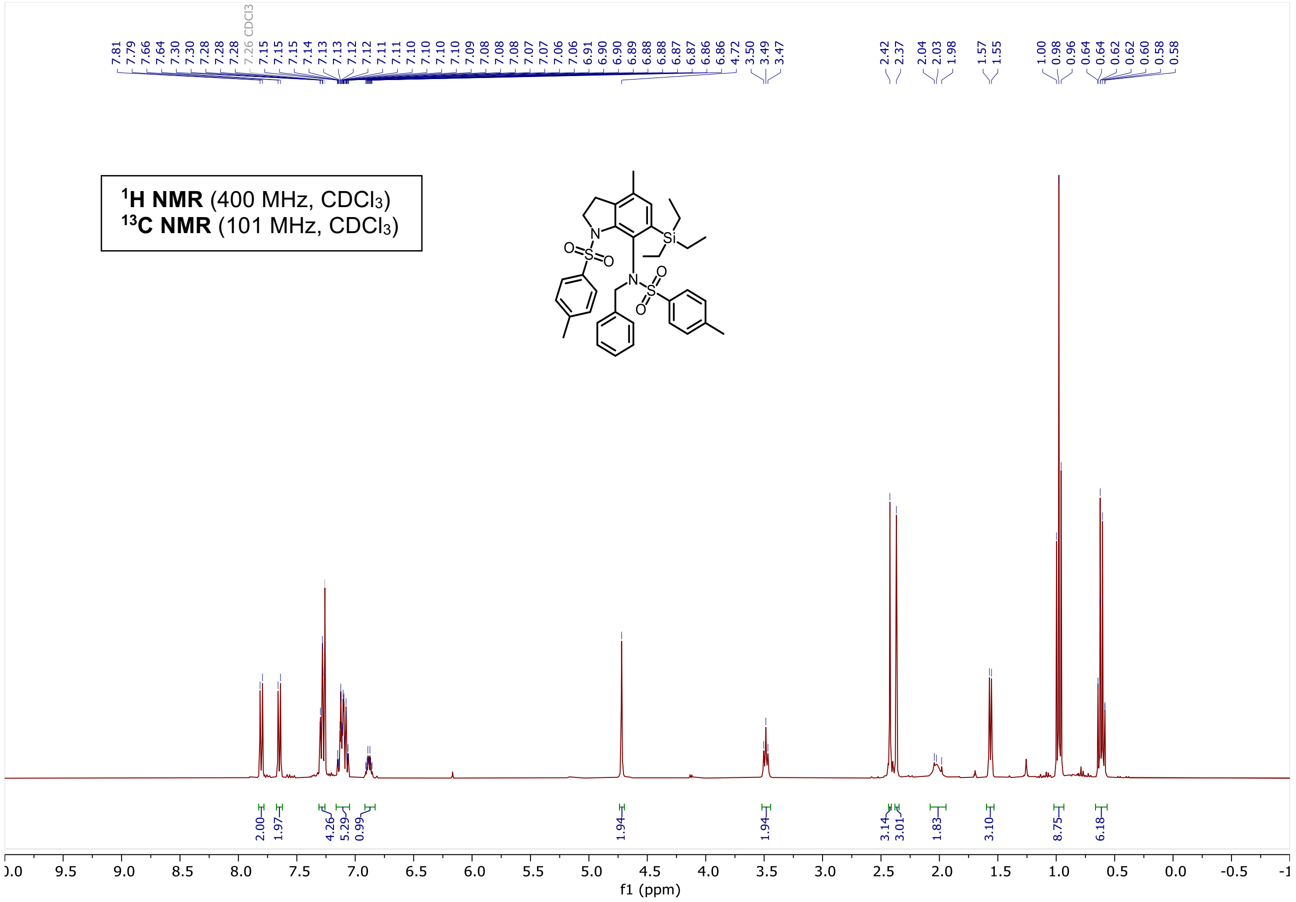

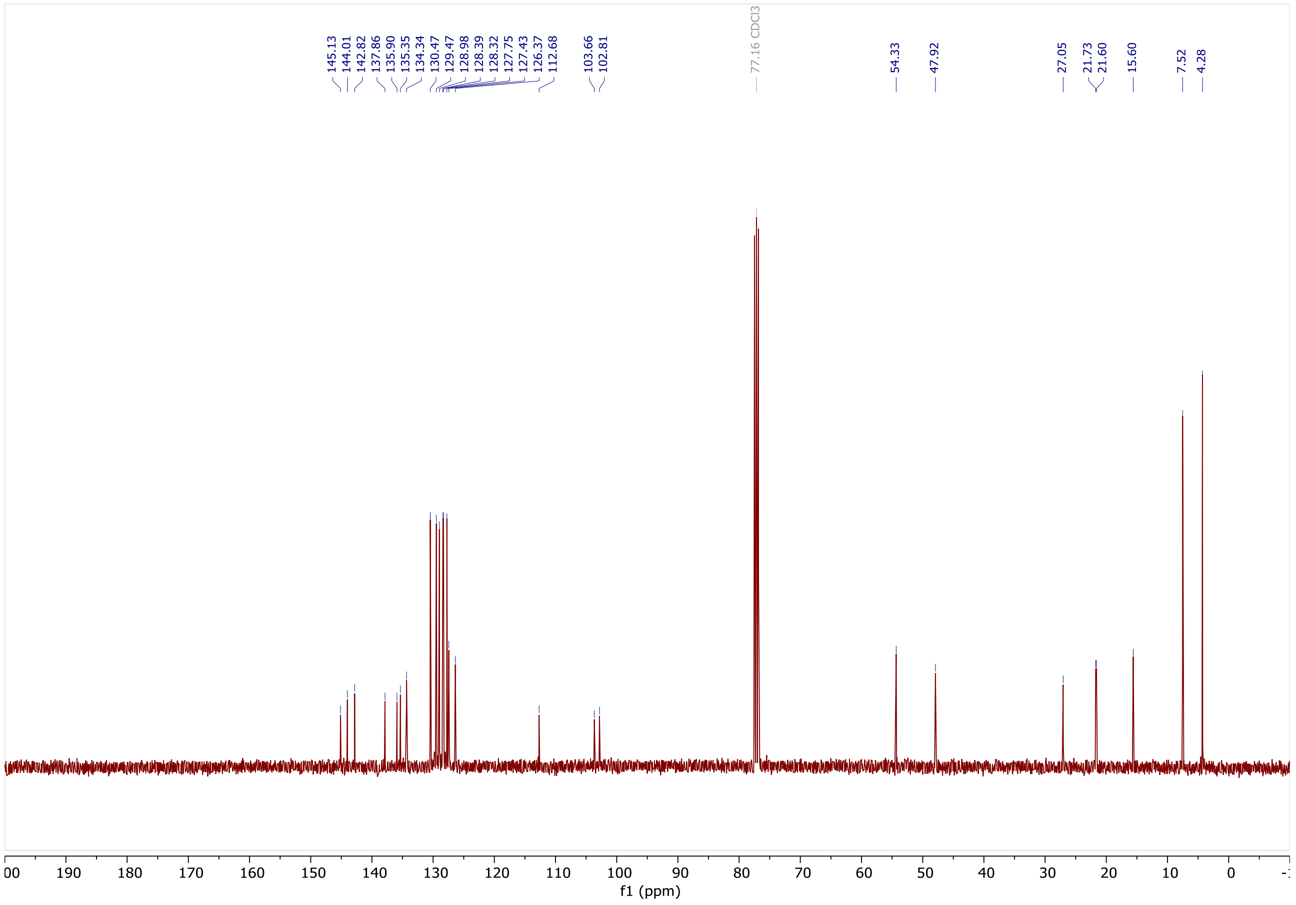

2ay ***N*-Benzyl-4-methyl-*N*-(4-methyl-6-(4,4,5,5-tetramethyl-1,3,2-dioxaborolan-2-yl)-1-tosylindolin-7-yl)benzenesulfonamide**

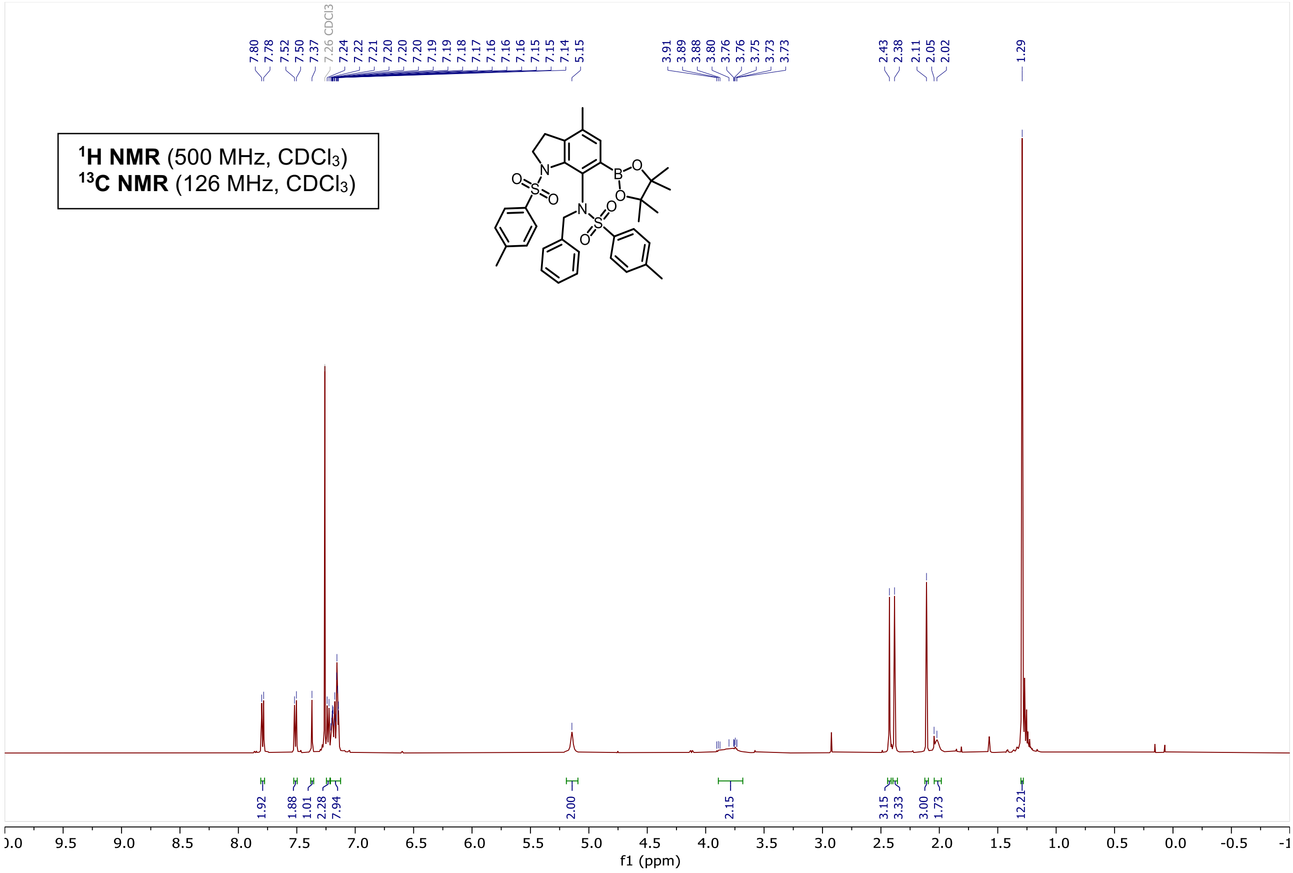

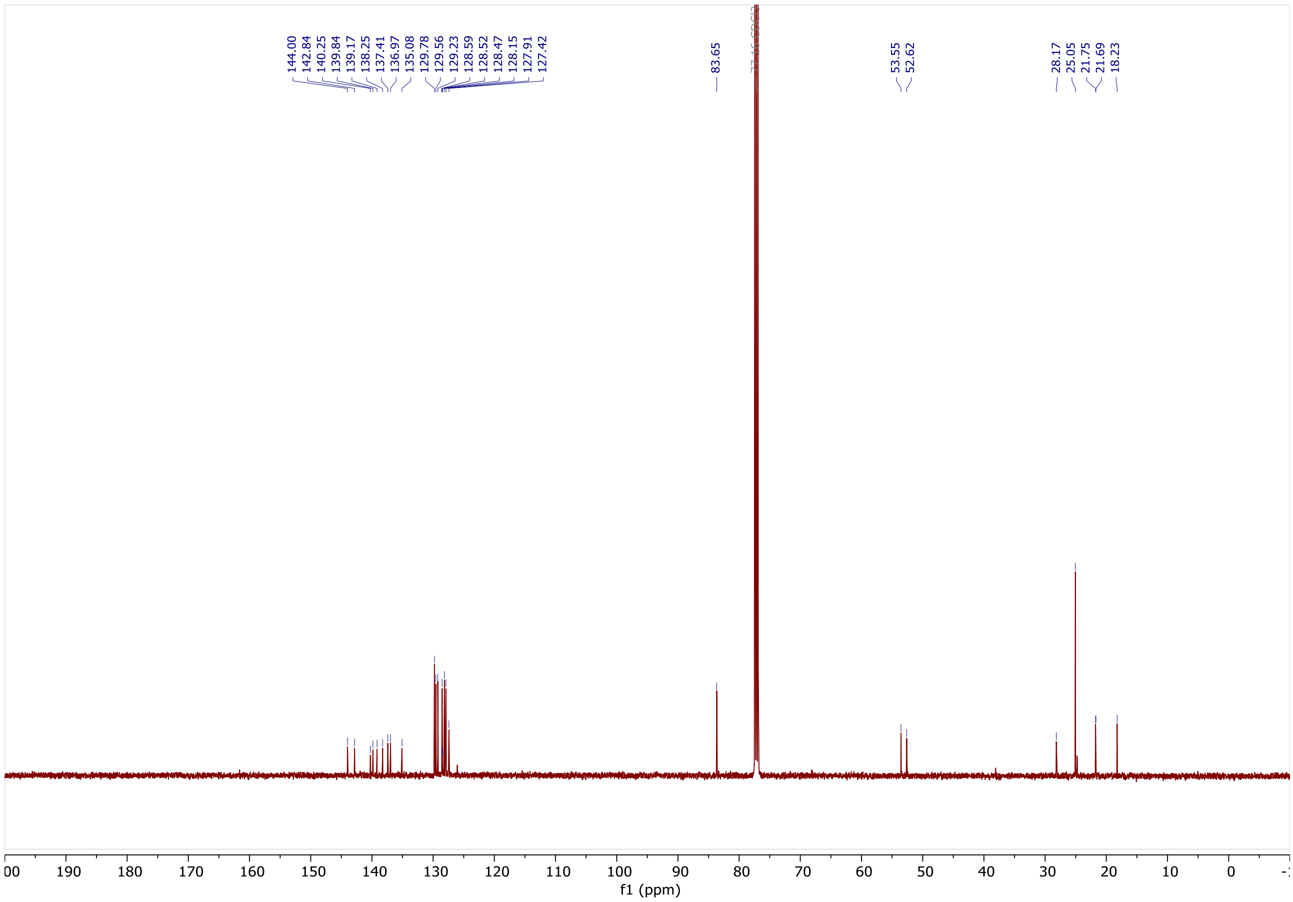

2ds (7-(9H-Carbazol-9-yl)-4-methyl-1-tosylindolin-5-yl)methanol

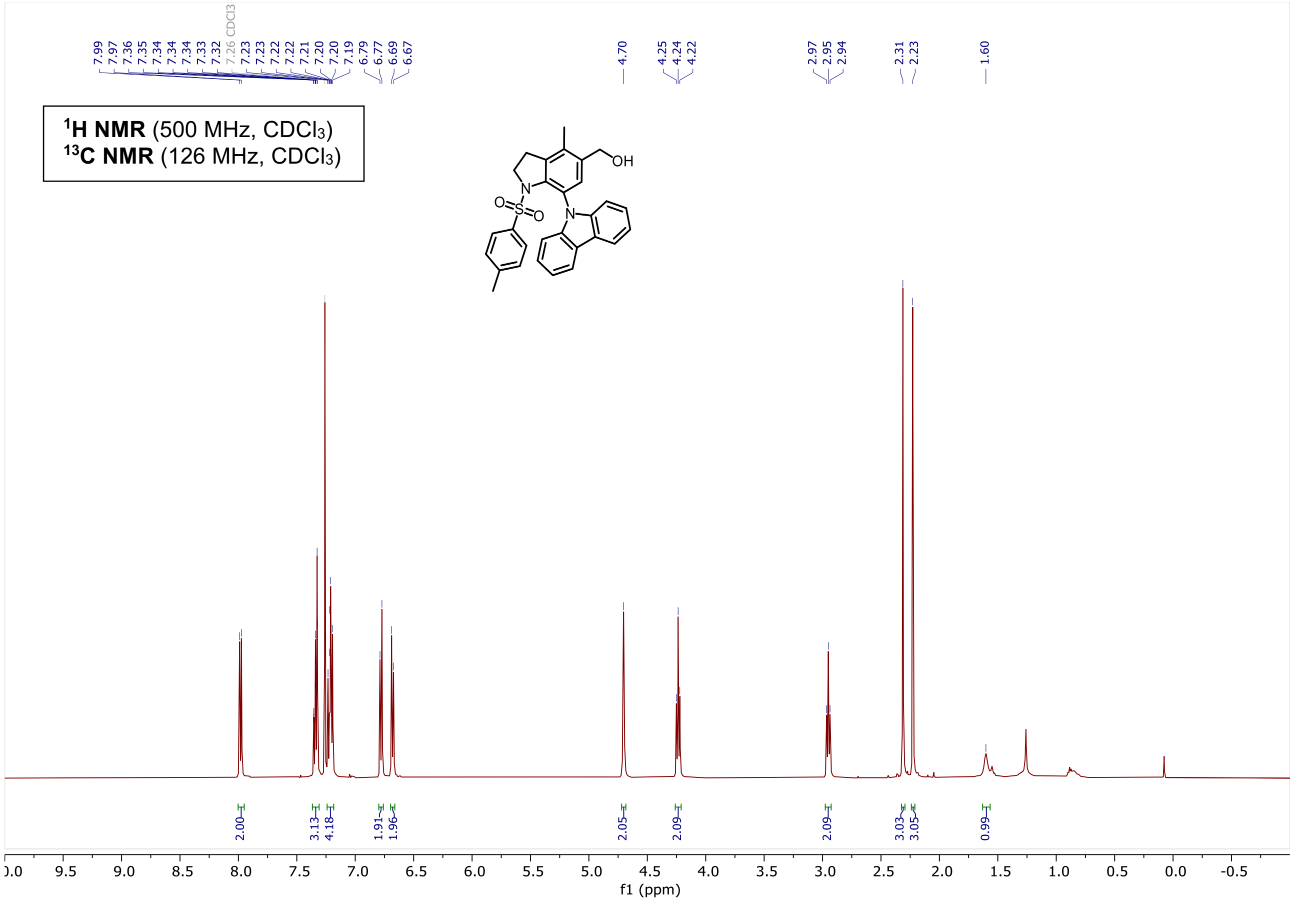

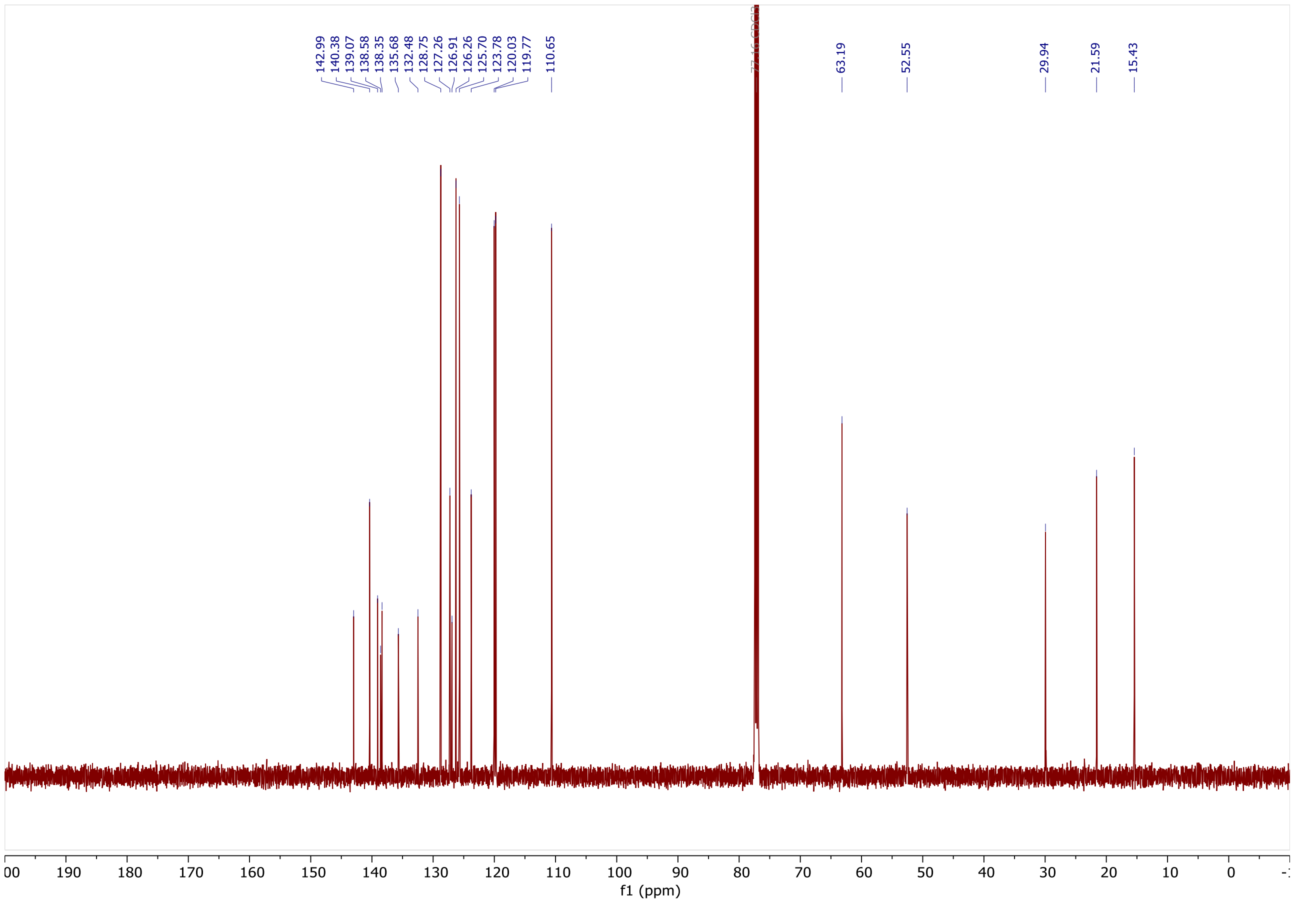

2db 4-(7-(9H-Carbazol-9-yl)-4-methyl-1-tosylindolin-5-yl)-*N*,*N*-dimethylaniline

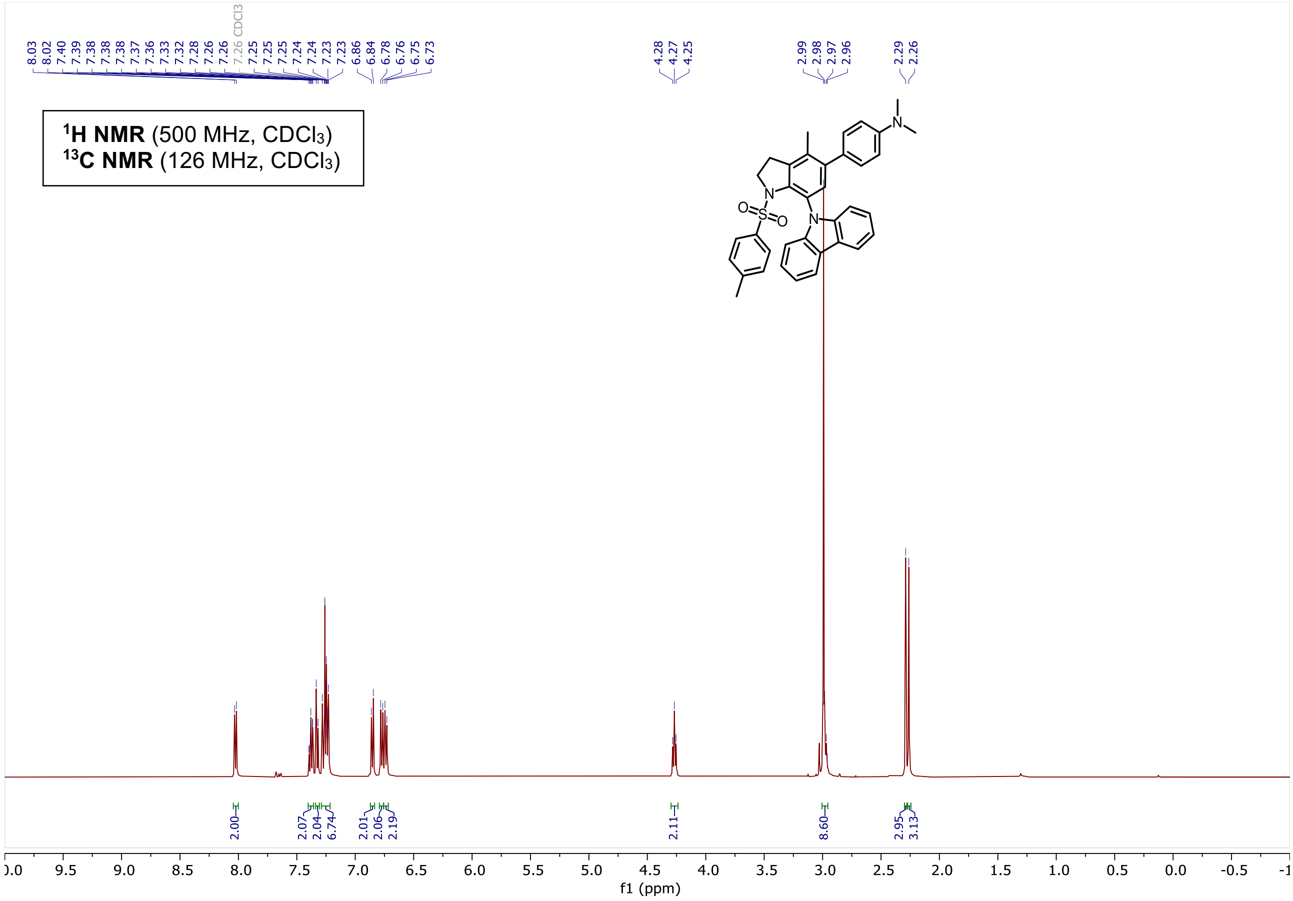

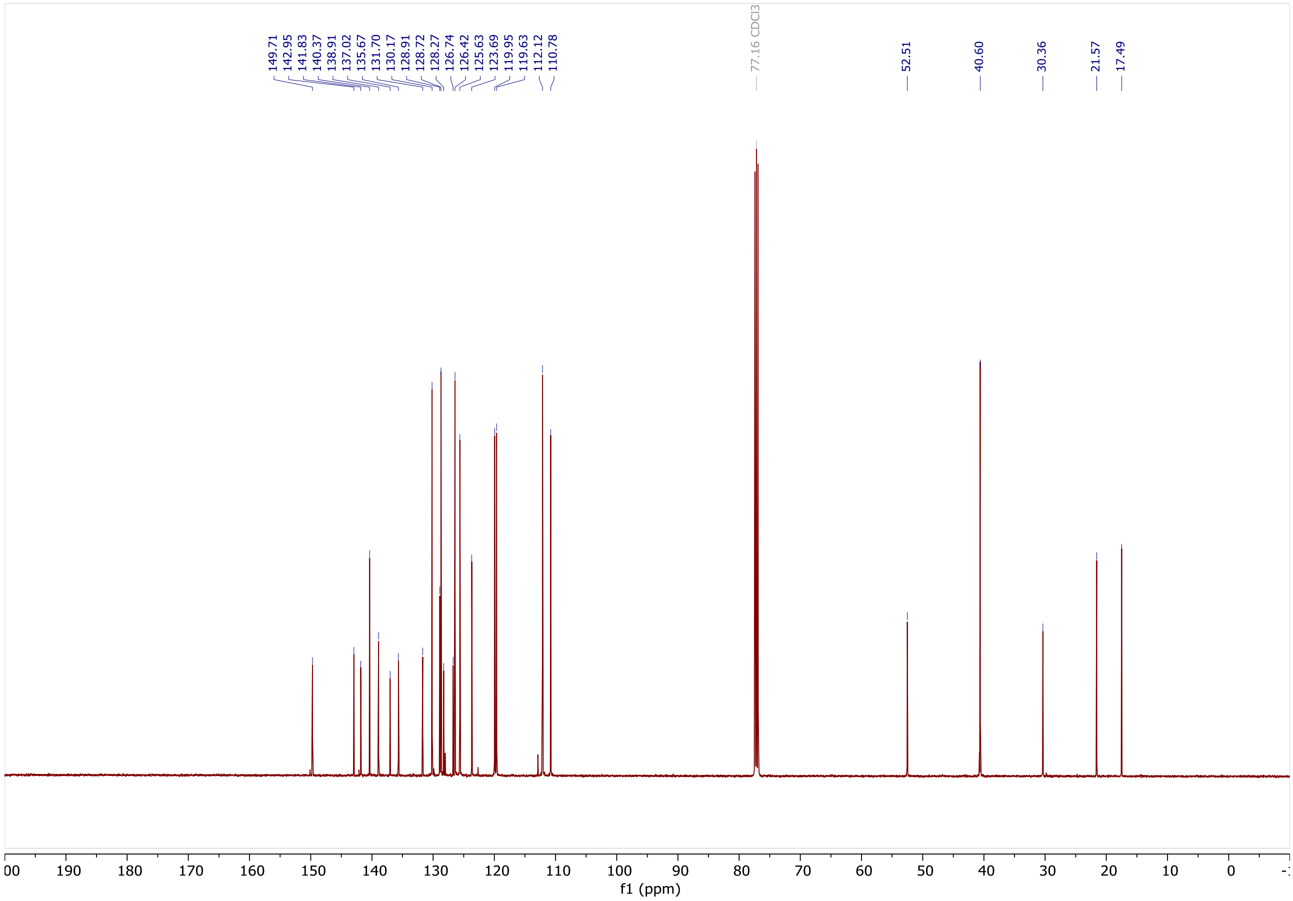

2de 9-(5-(4-Methoxyphenyl)-4-methyl-1-tosylindolin-7-yl)-9H-carbazole

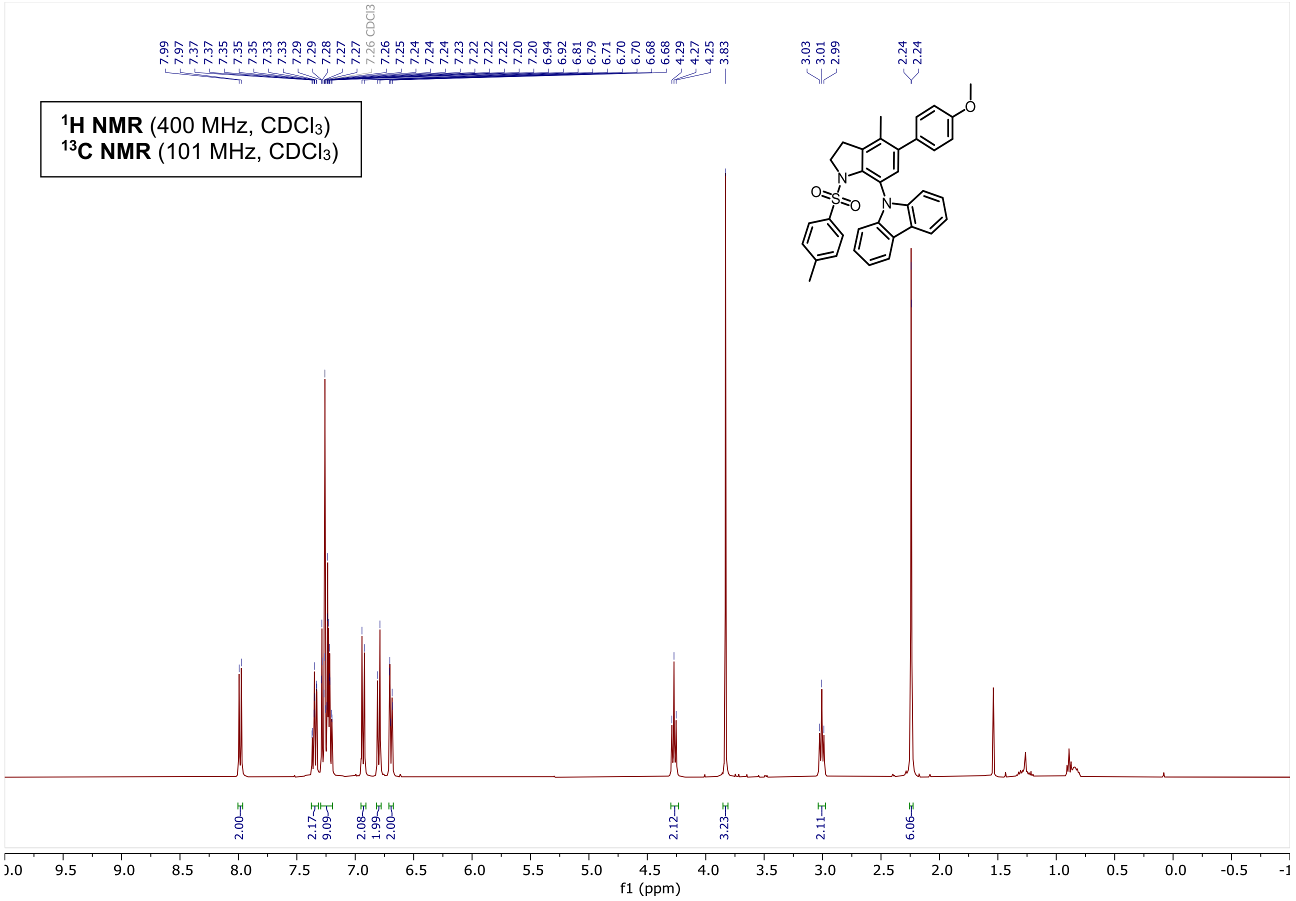

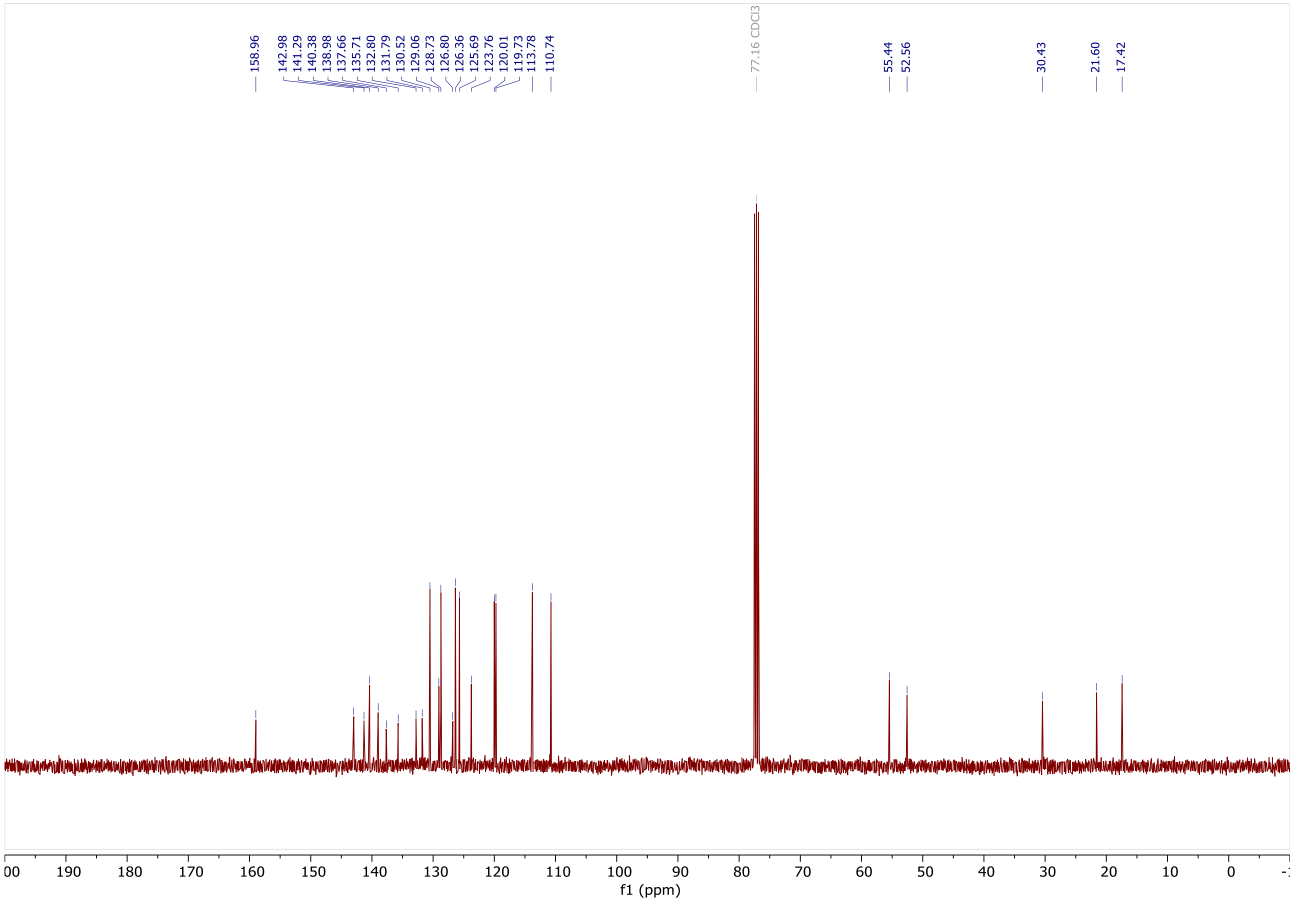

2df 9-(4-Methyl-5-phenyl-1-tosylindolin-7-yl)-9H-carbazole

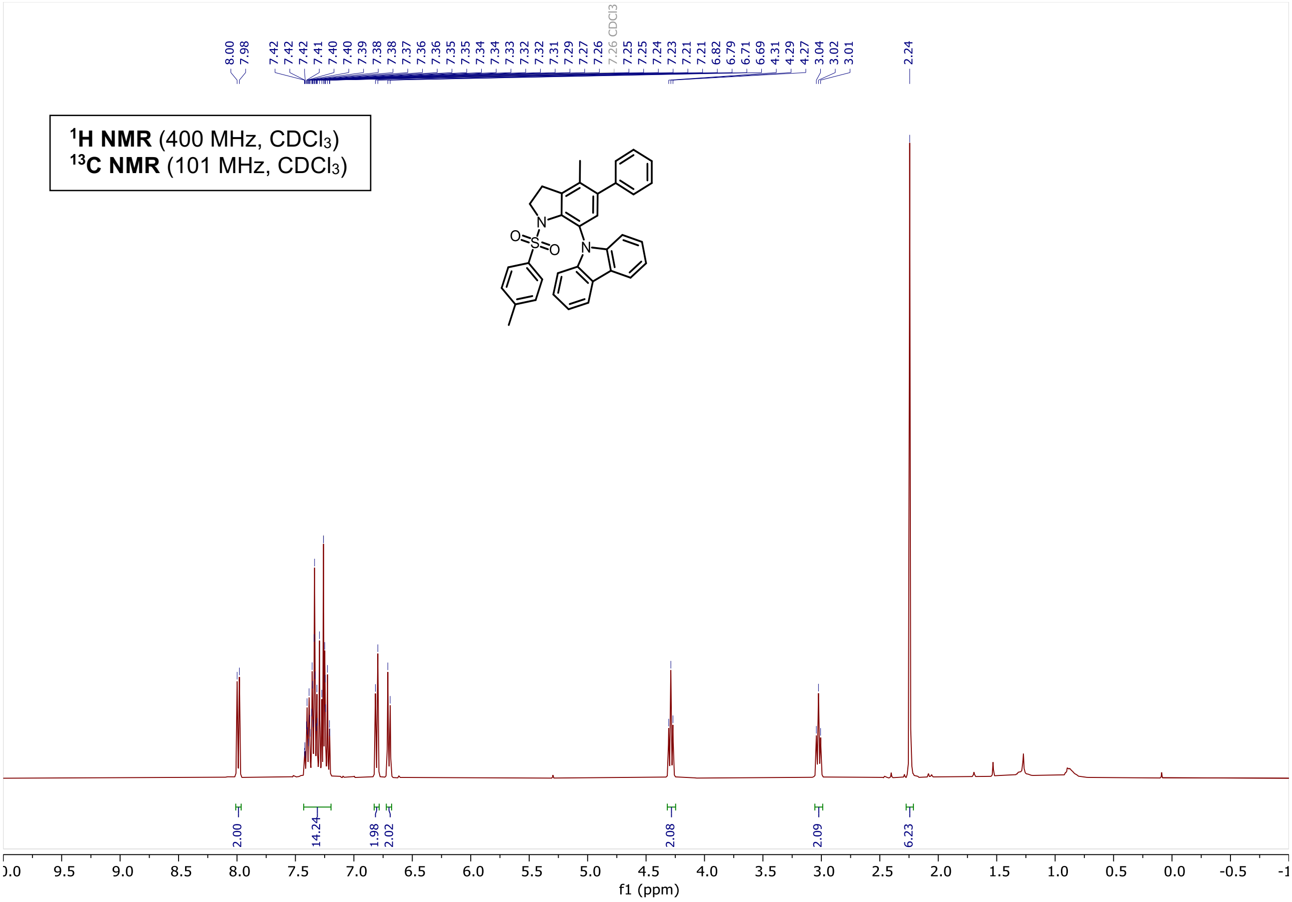

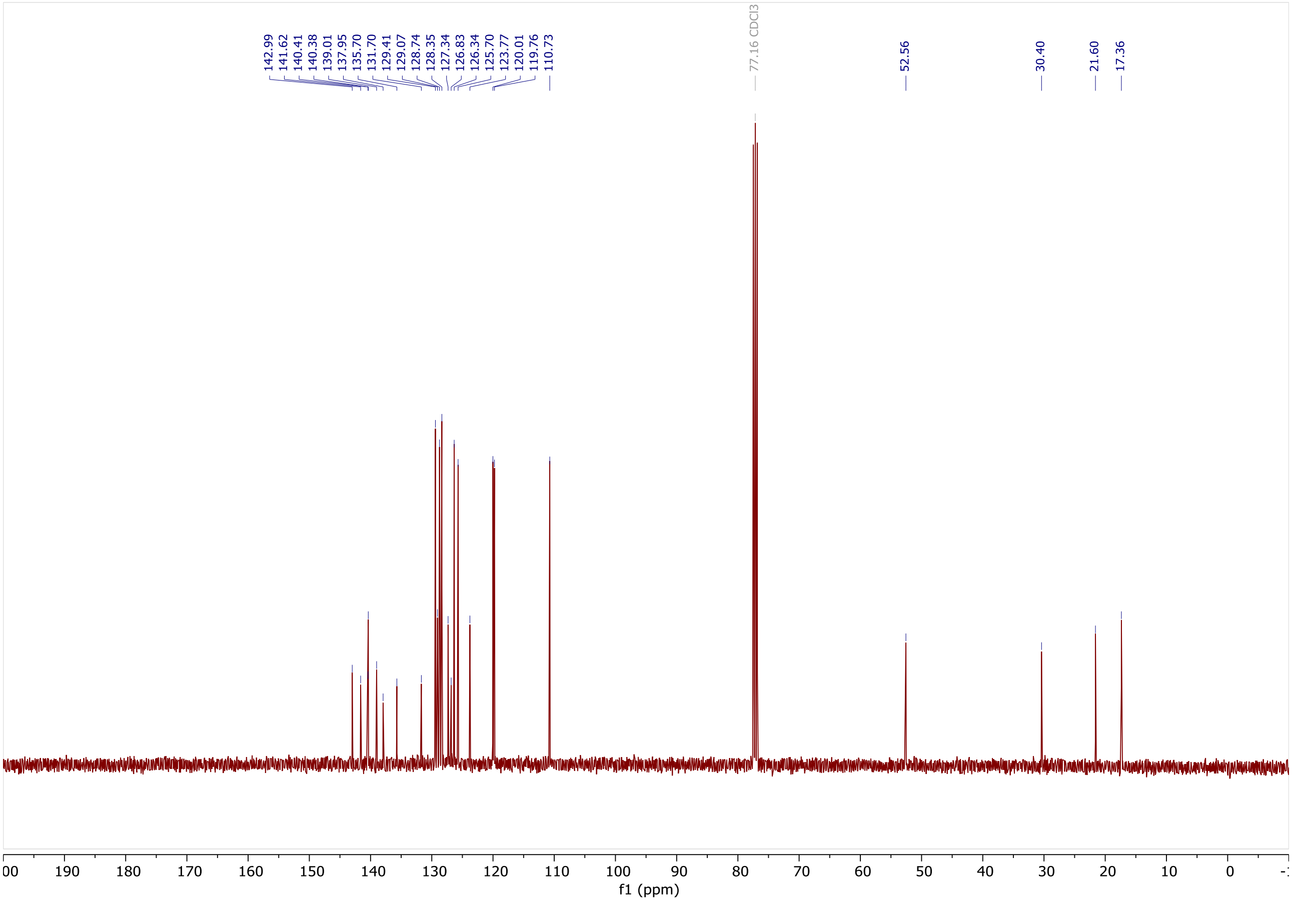

2di 9-(5-(4-Fluorophenyl)-4-methyl-1-tosylindolin-7-yl)-9H-carbazole

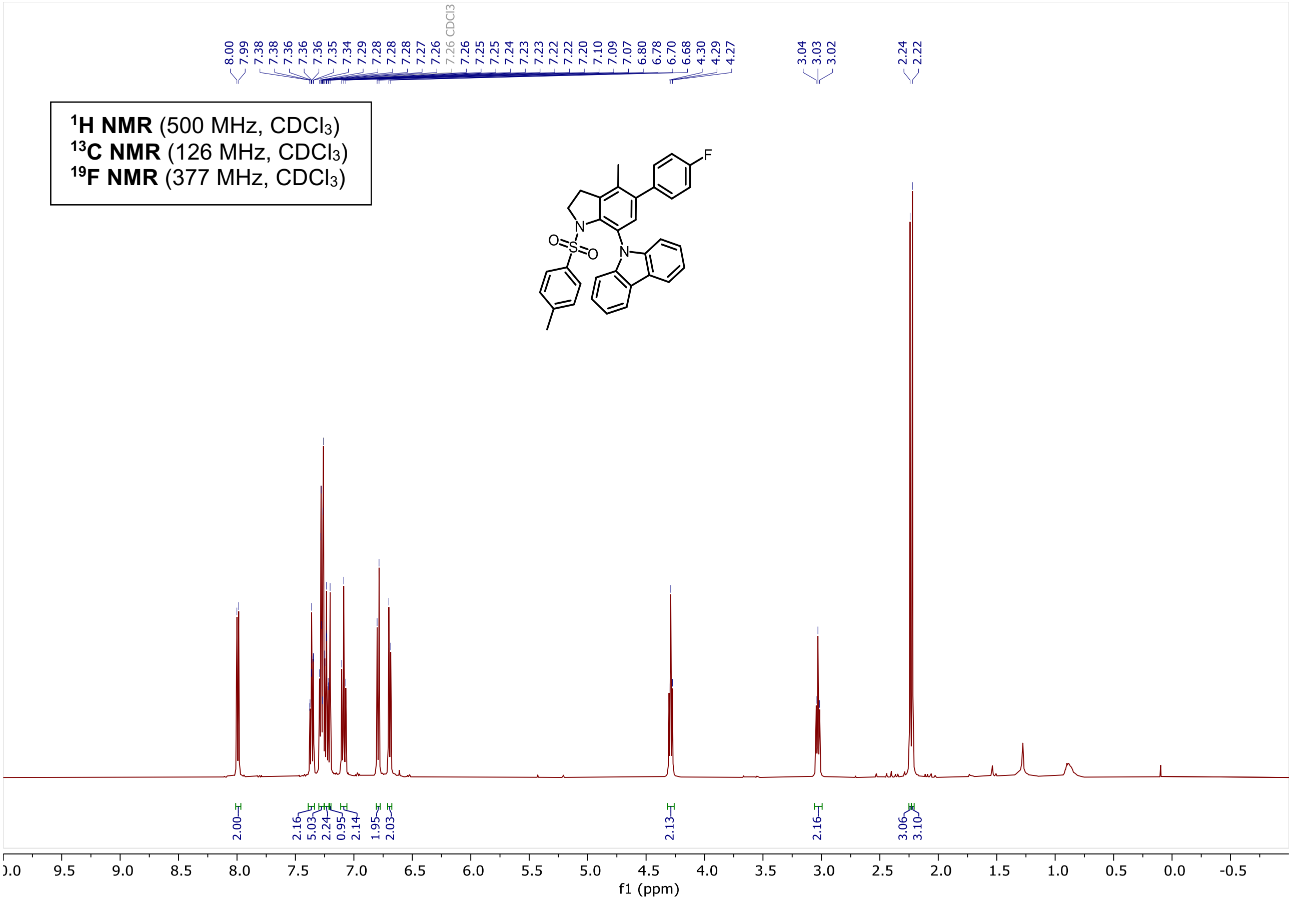

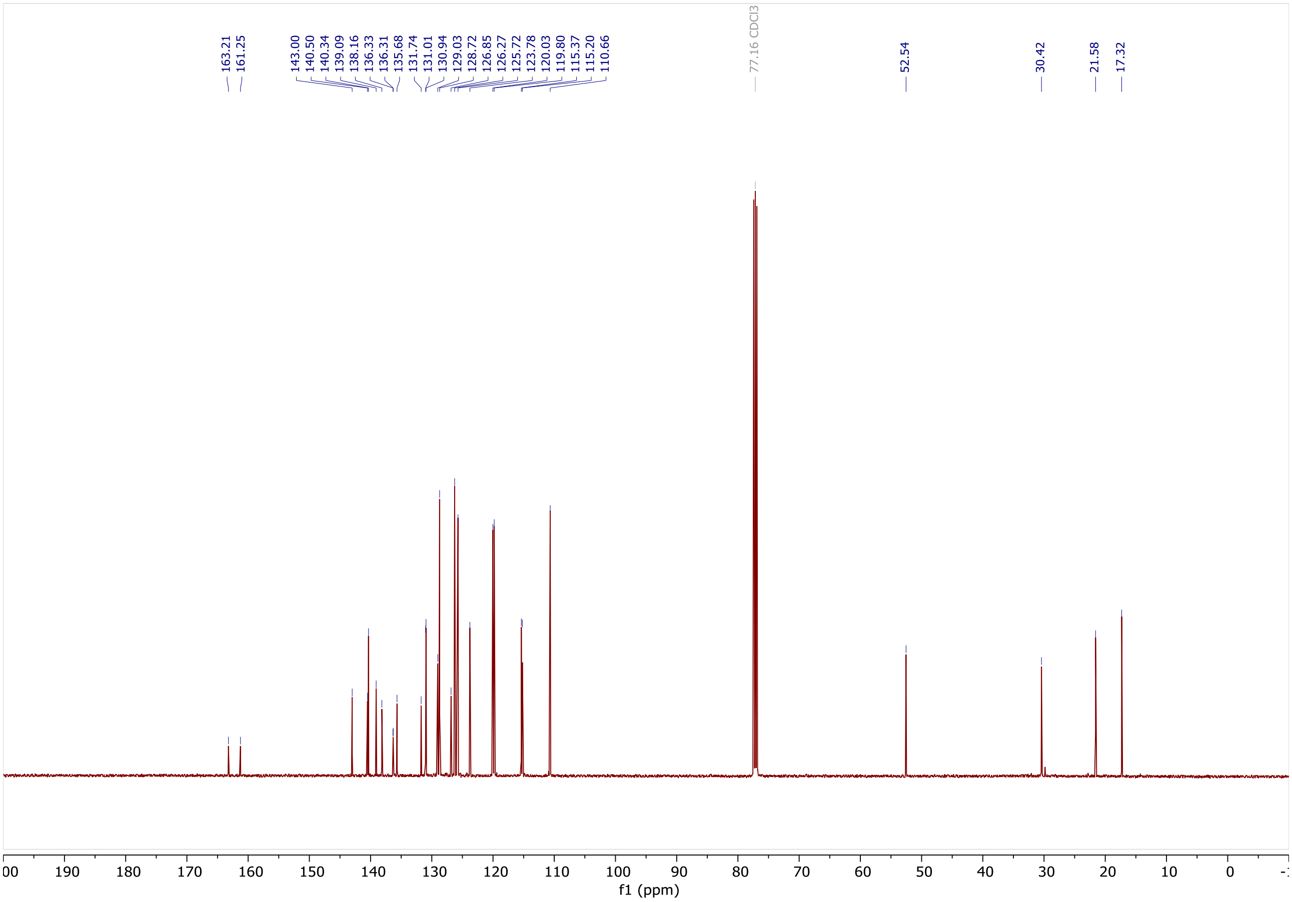

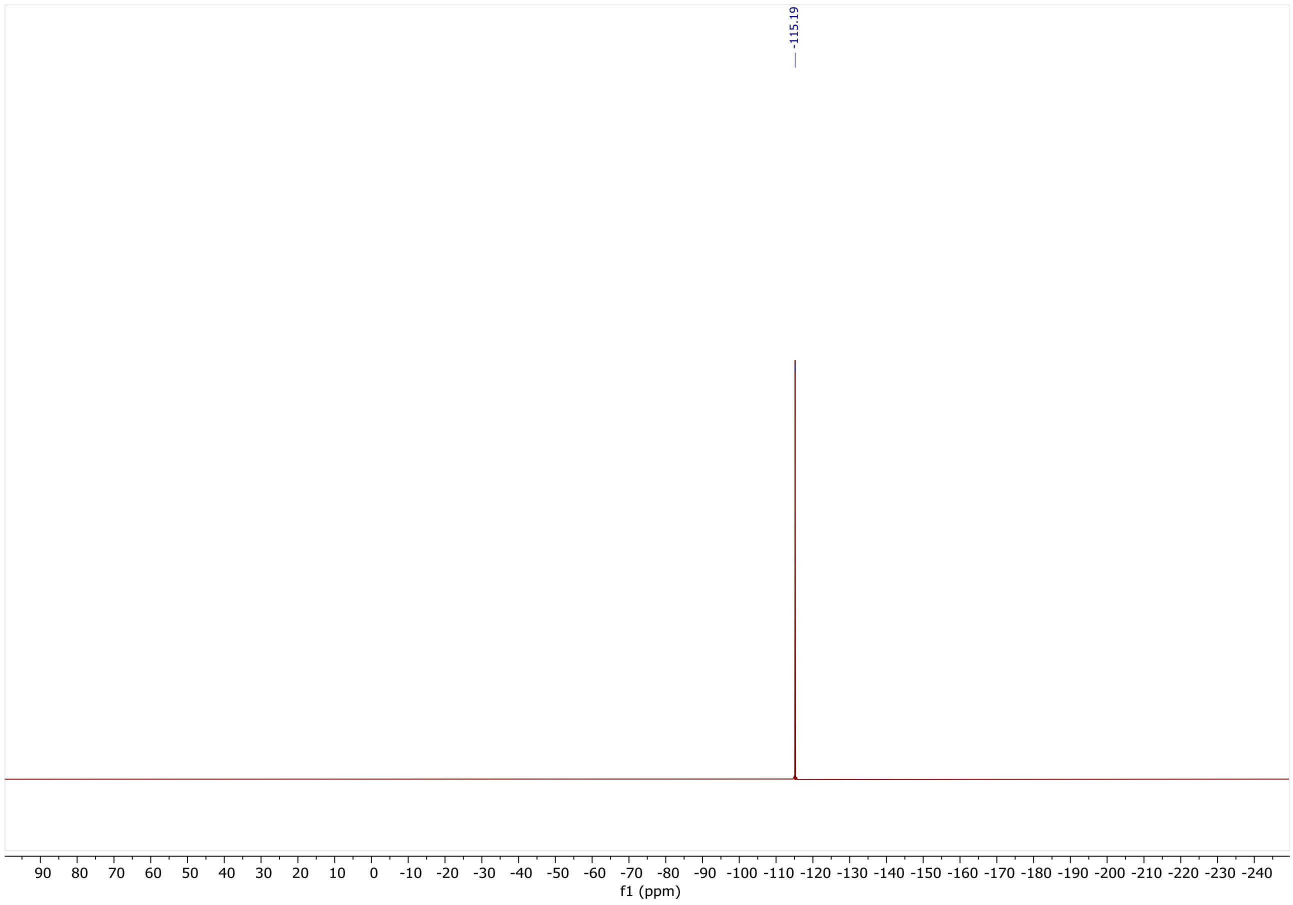
-115.19
90 80 70 60 50 40 30 20 10 0 -10 -20 -30 -40 -50 -60 -70 -80 -90 -100 -110 -120 -130 -140 -150 -160 -170 -180 -190 -200 -210 -220 -230 -240
f1 (ppm)

2do 4-(7-(9H-Carbazol-9-yl)-4-methyl-1-tosylindolin-5-yl)benzonitrile

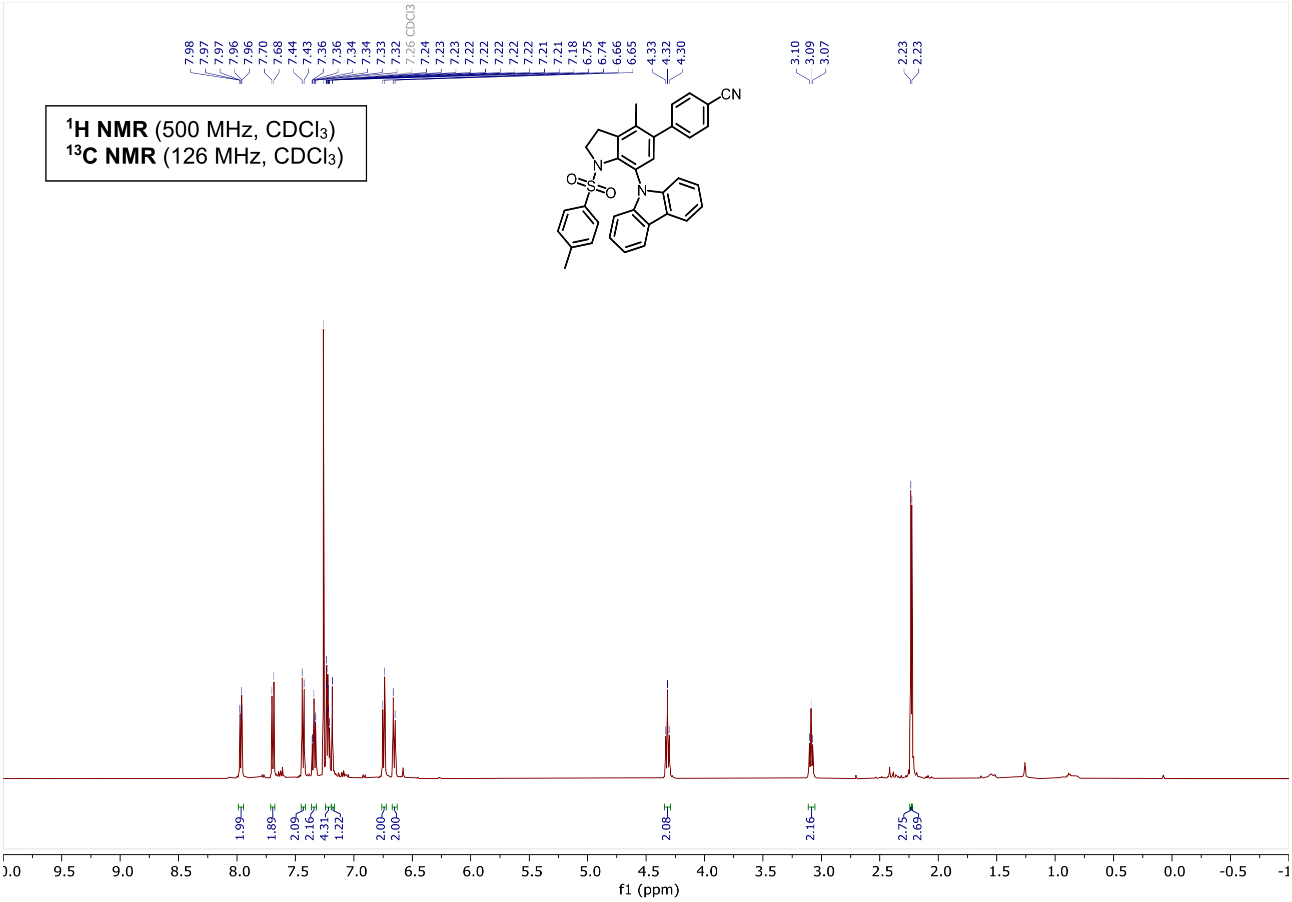

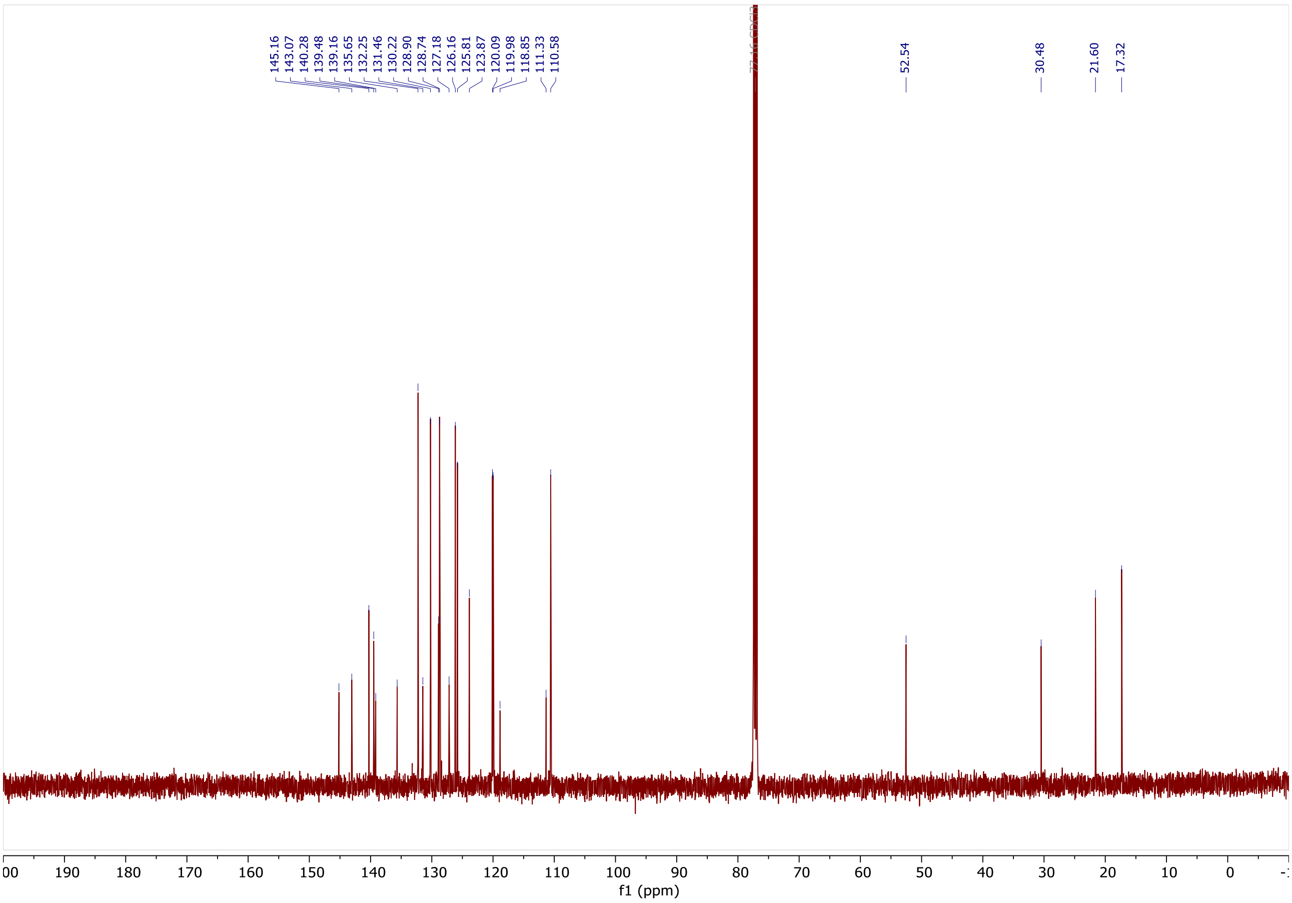

2dp 9-(5-(2-Methoxyphenyl)-4-methyl-1-tosylindolin-7-yl)-9H-carbazole

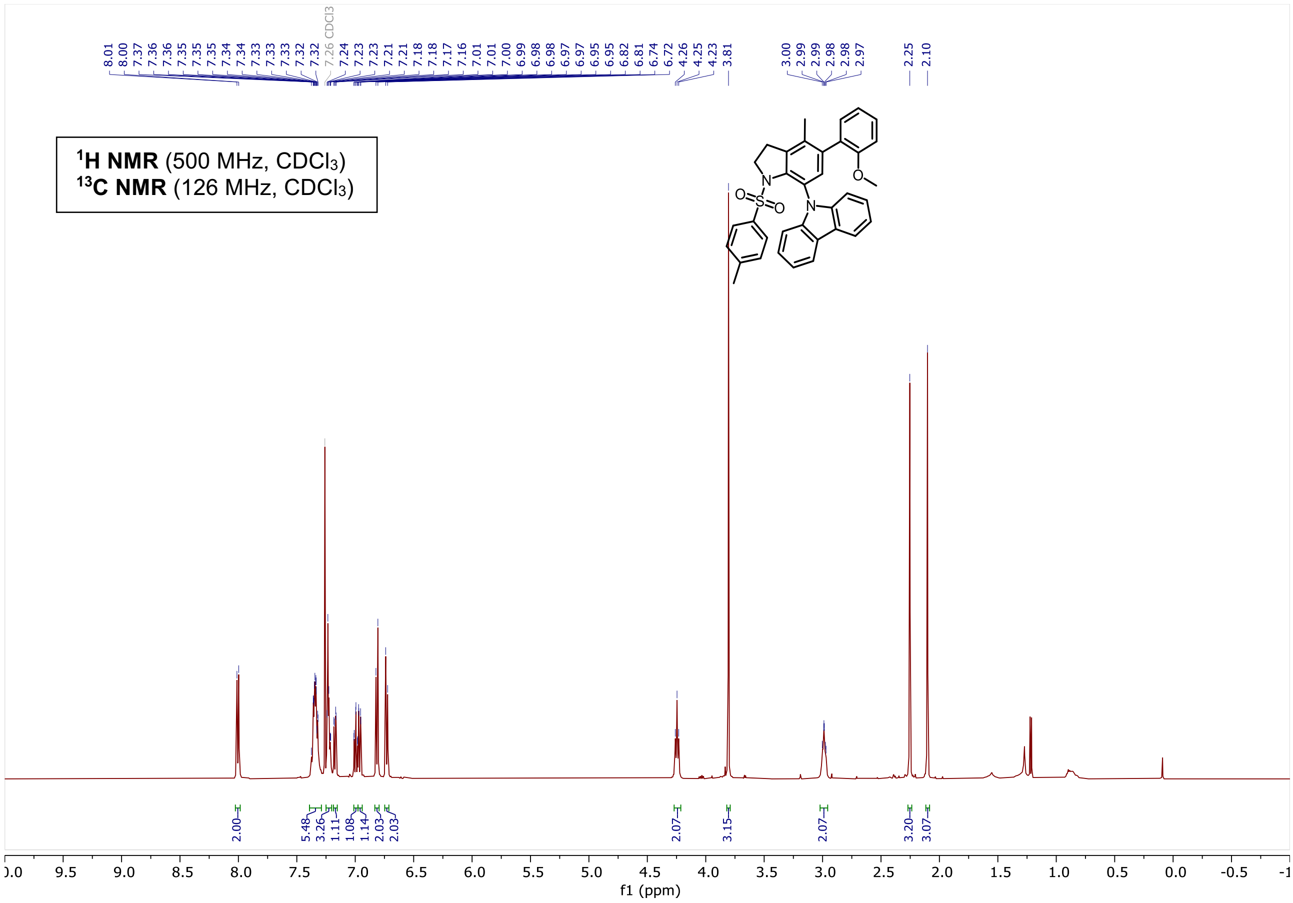

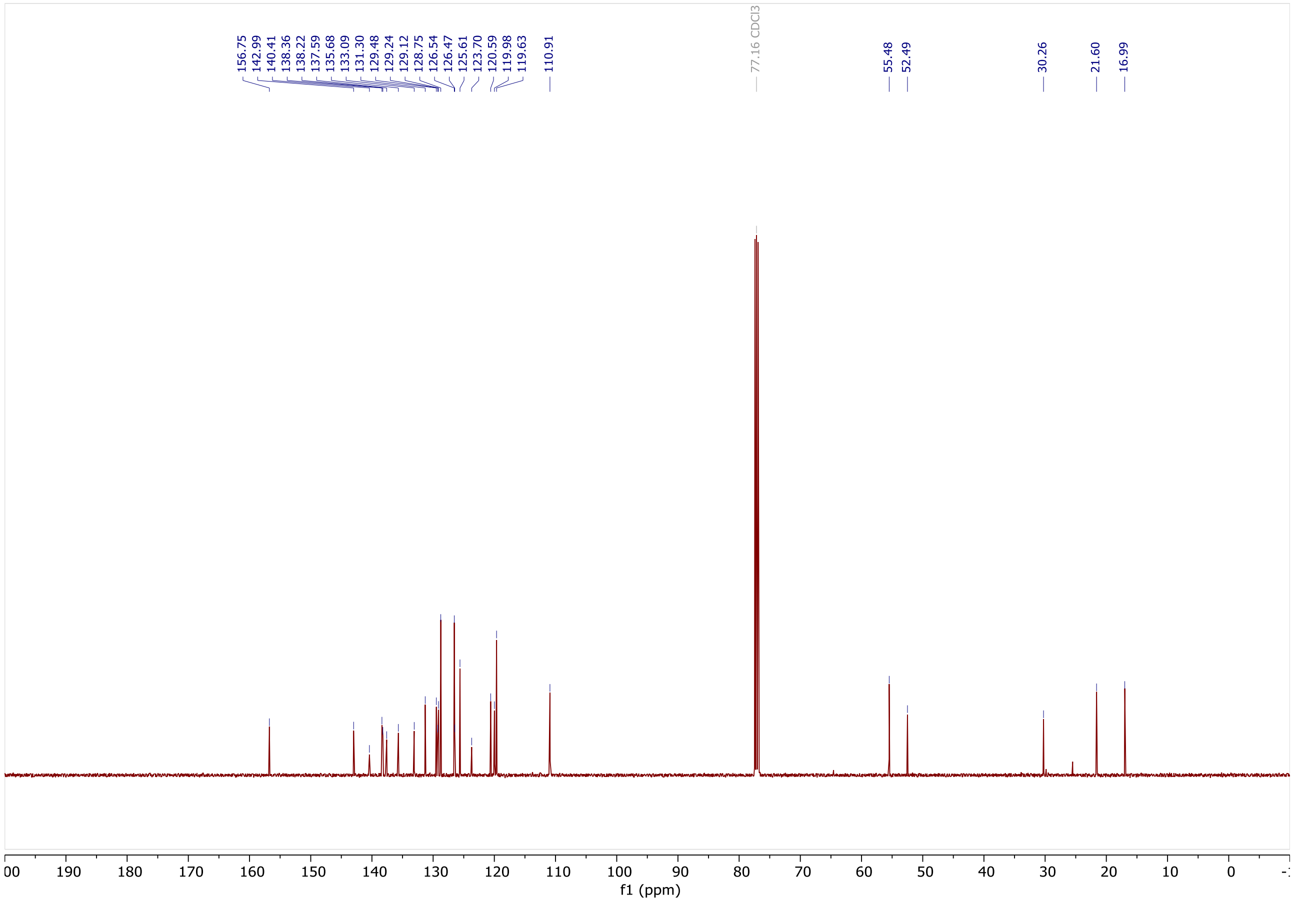

2da (7-(9H-Carbazol-9-yl)-4-methyl-1-tosylindoline-5,6-diyl)dimethanol

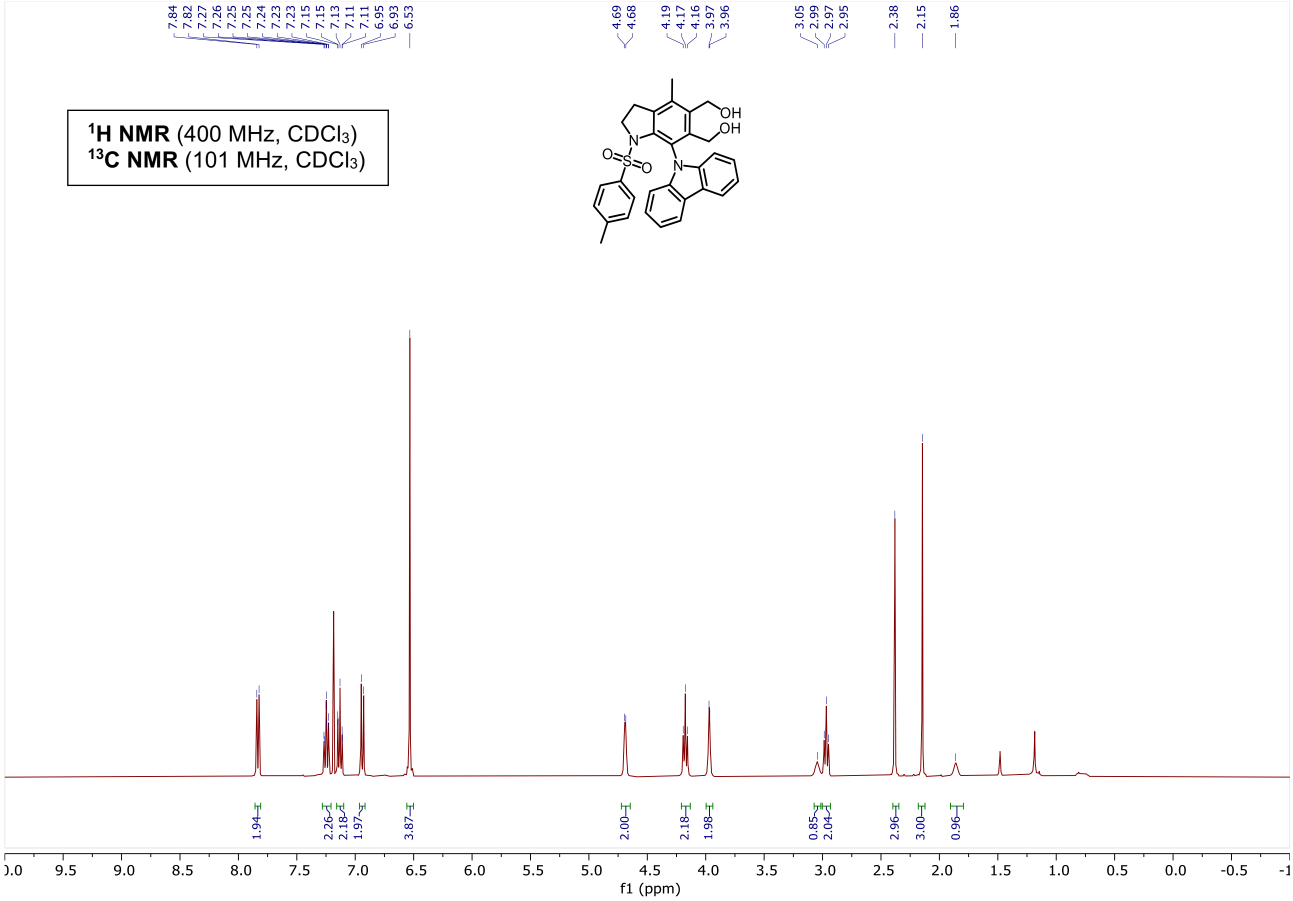

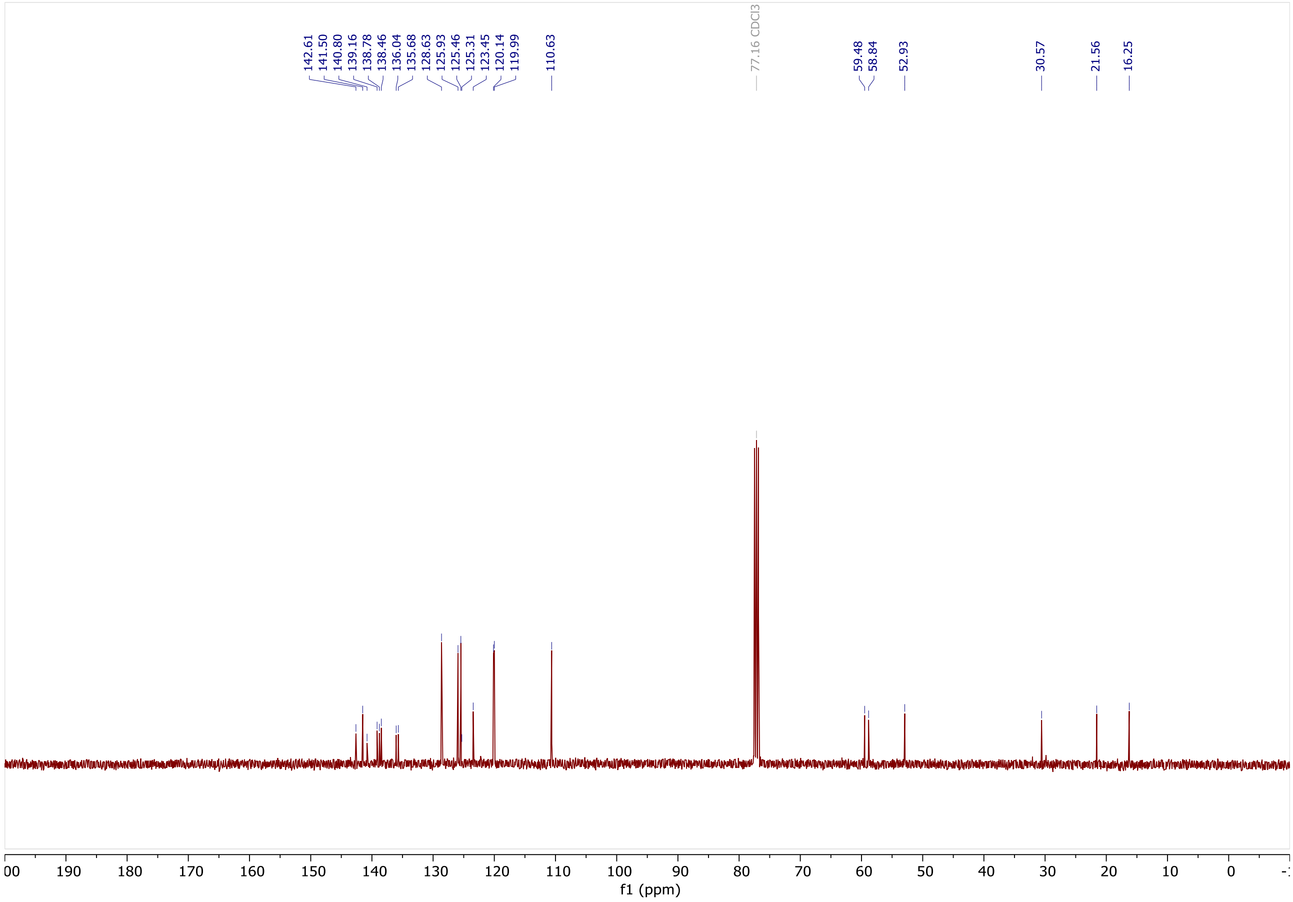

2dw 9-(4-Methyl-1-tosyl-6-(triethylsilyl)indolin-7-yl)-9*H*-carbazole

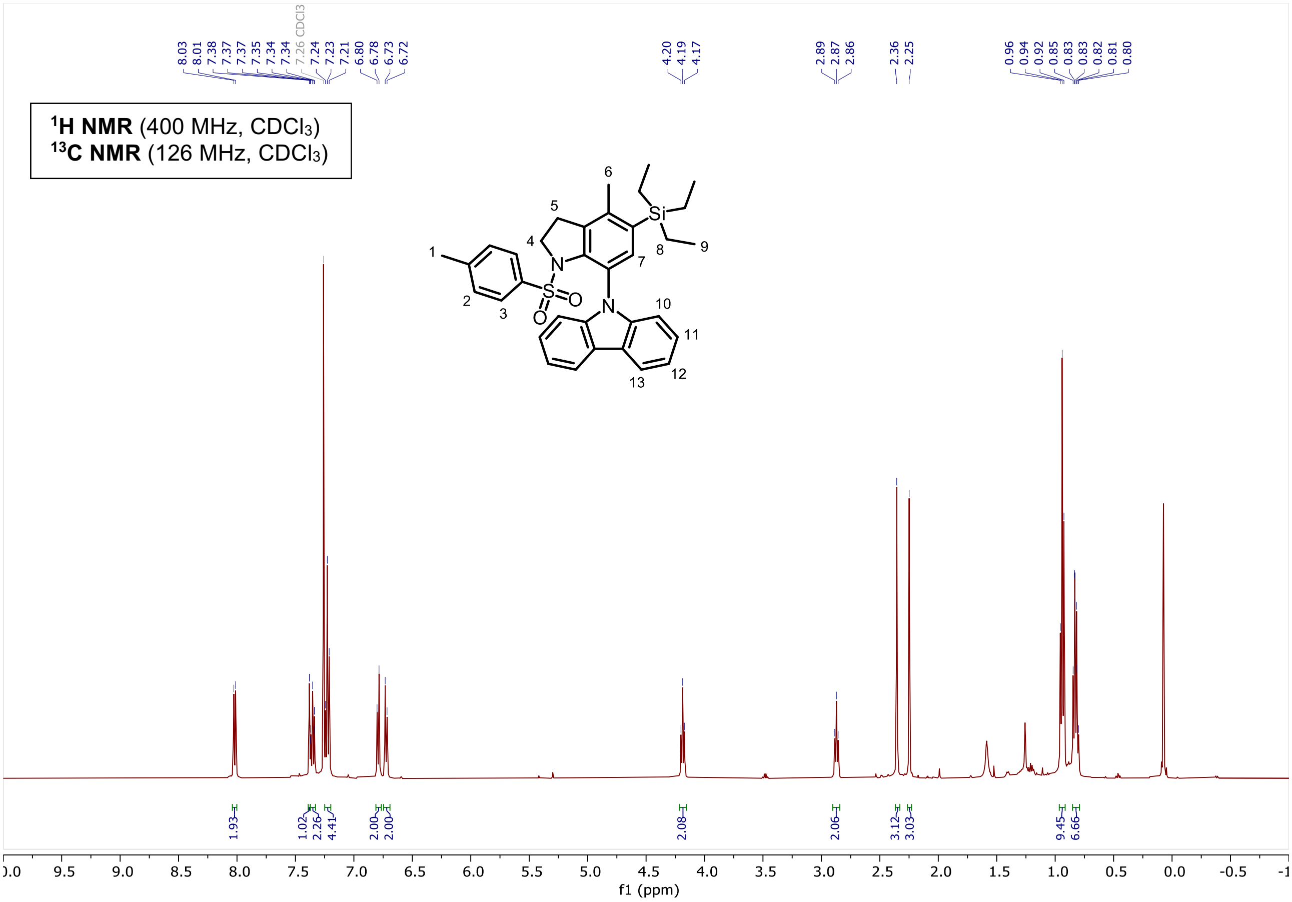

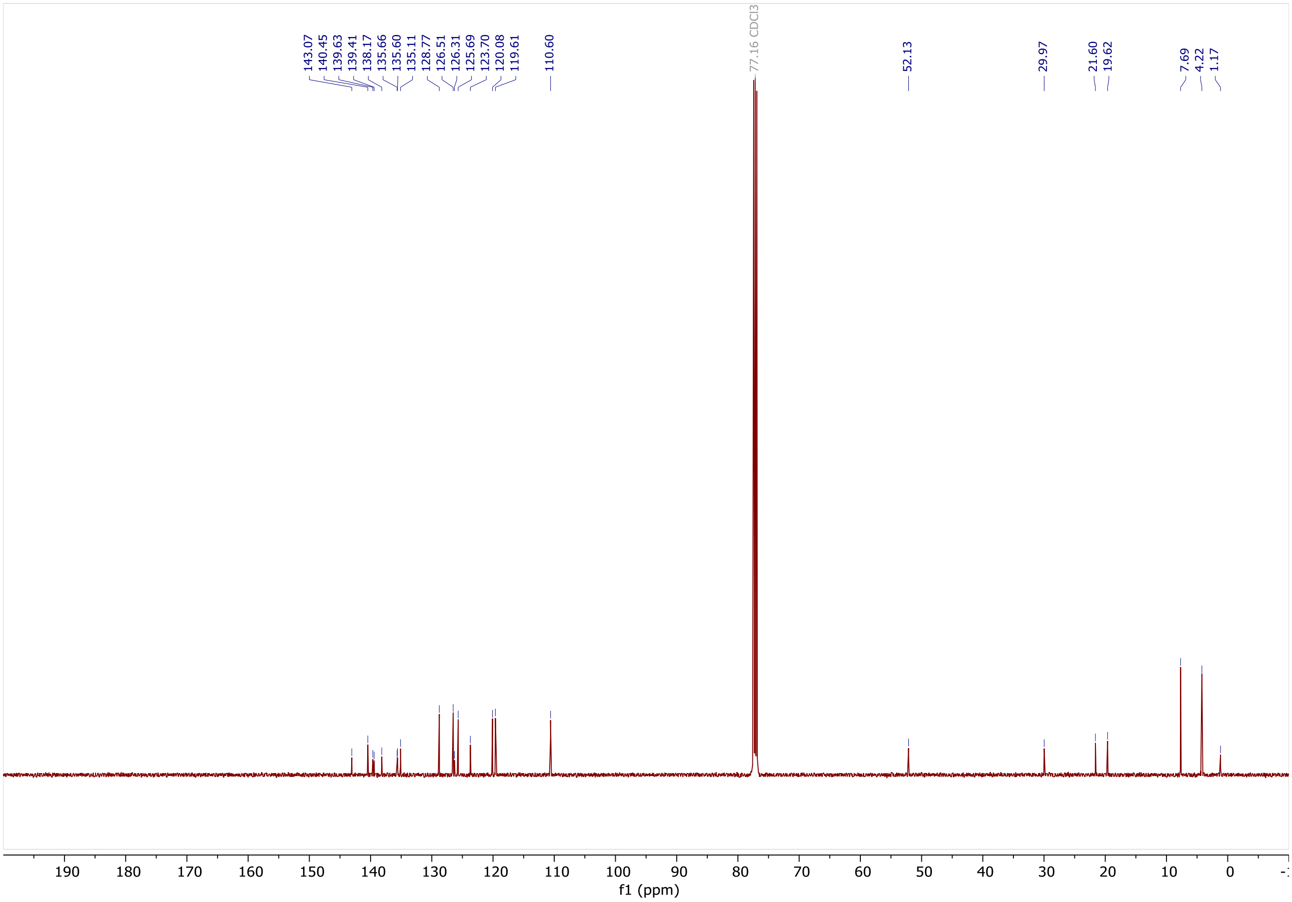

7aa *N*,*N'*-(2,3-Dihydro-1H-indene-5,6-diyl)bis(*N*-butyl-4-methylbenzenesulfonamide)

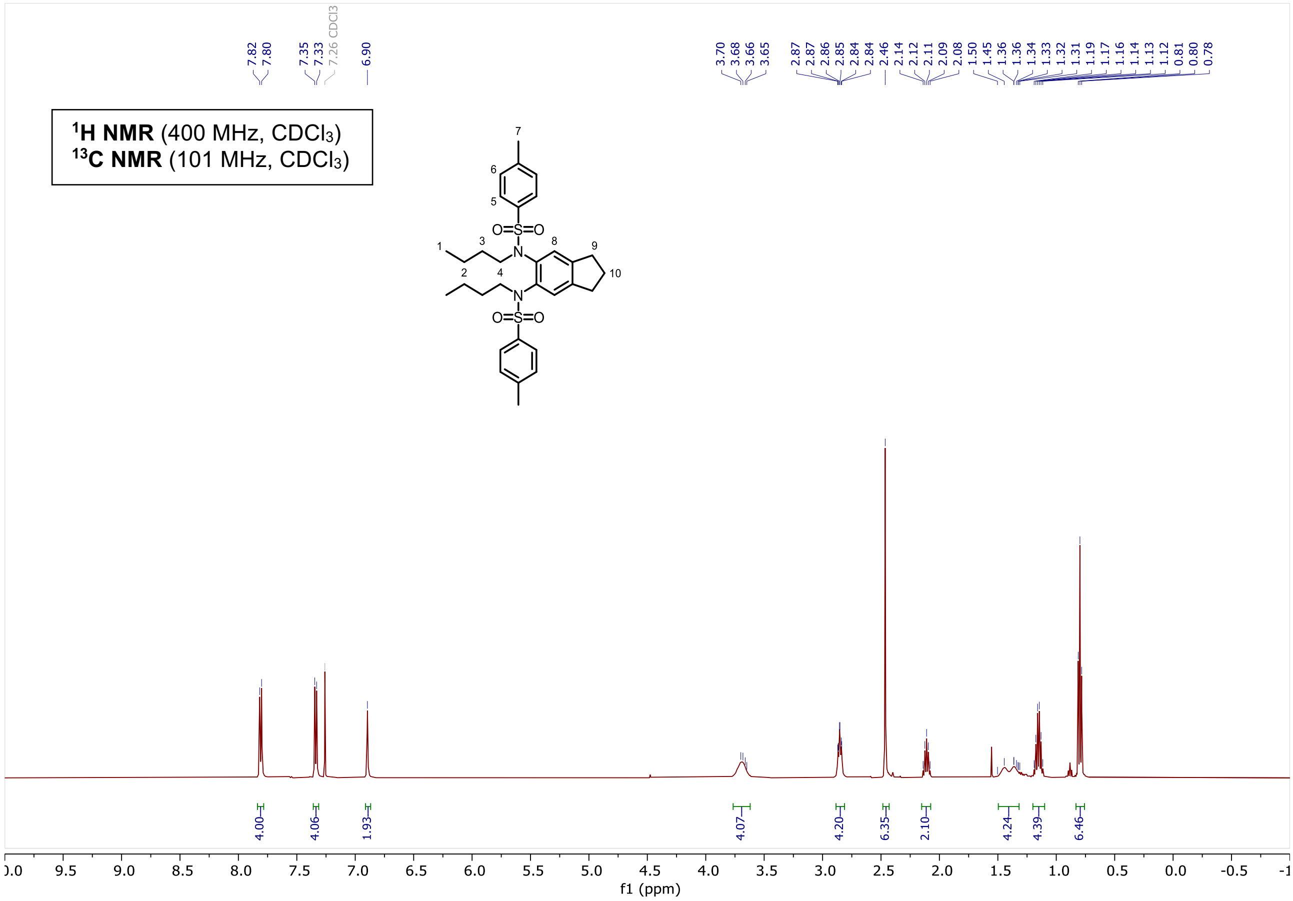

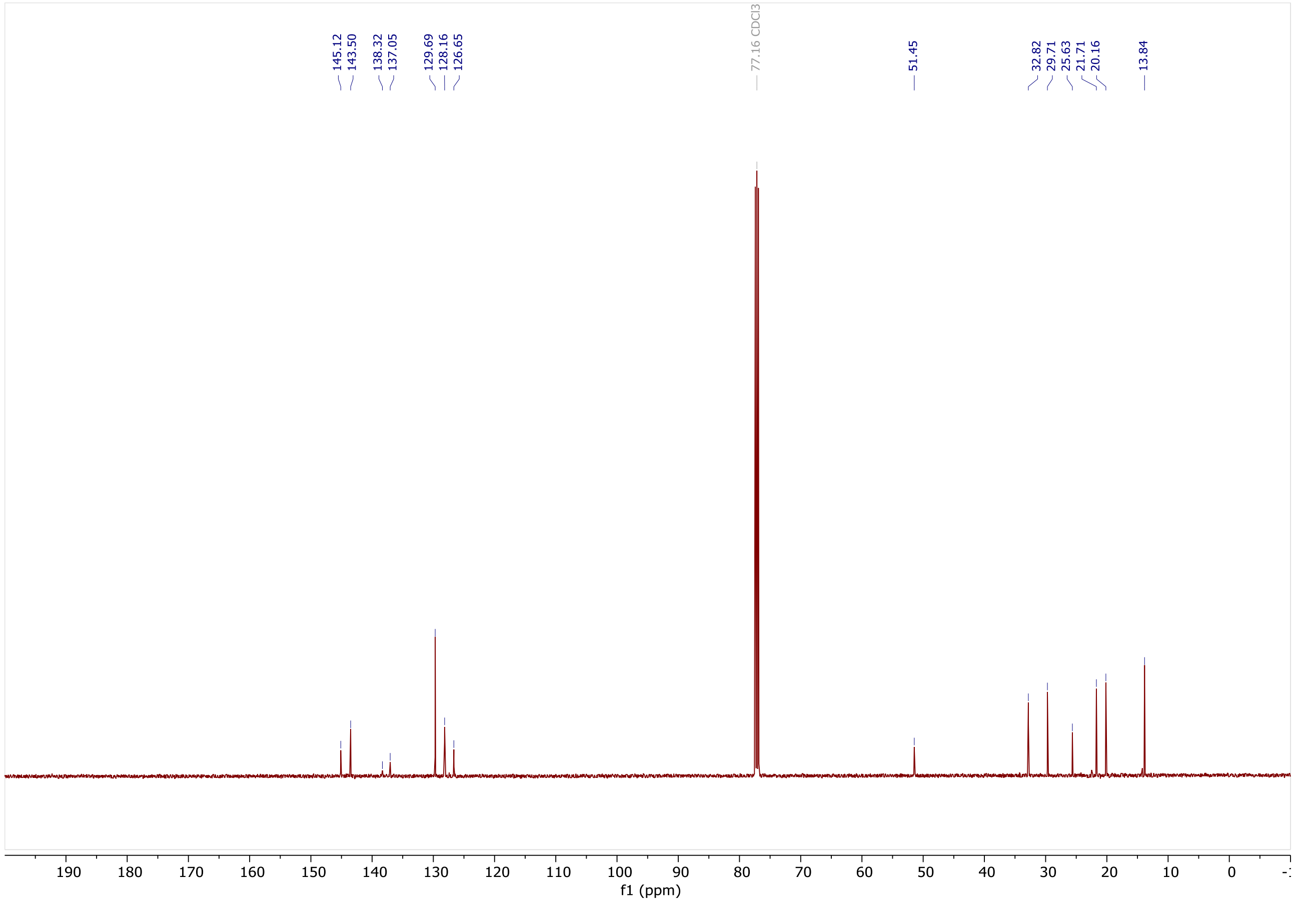

7ab ***N*,*N'*-(2,3-Dihydro-1H-indene-5,6-diyl)bis(*N*-benzyl-4-methylbenzenesulfonamide)**

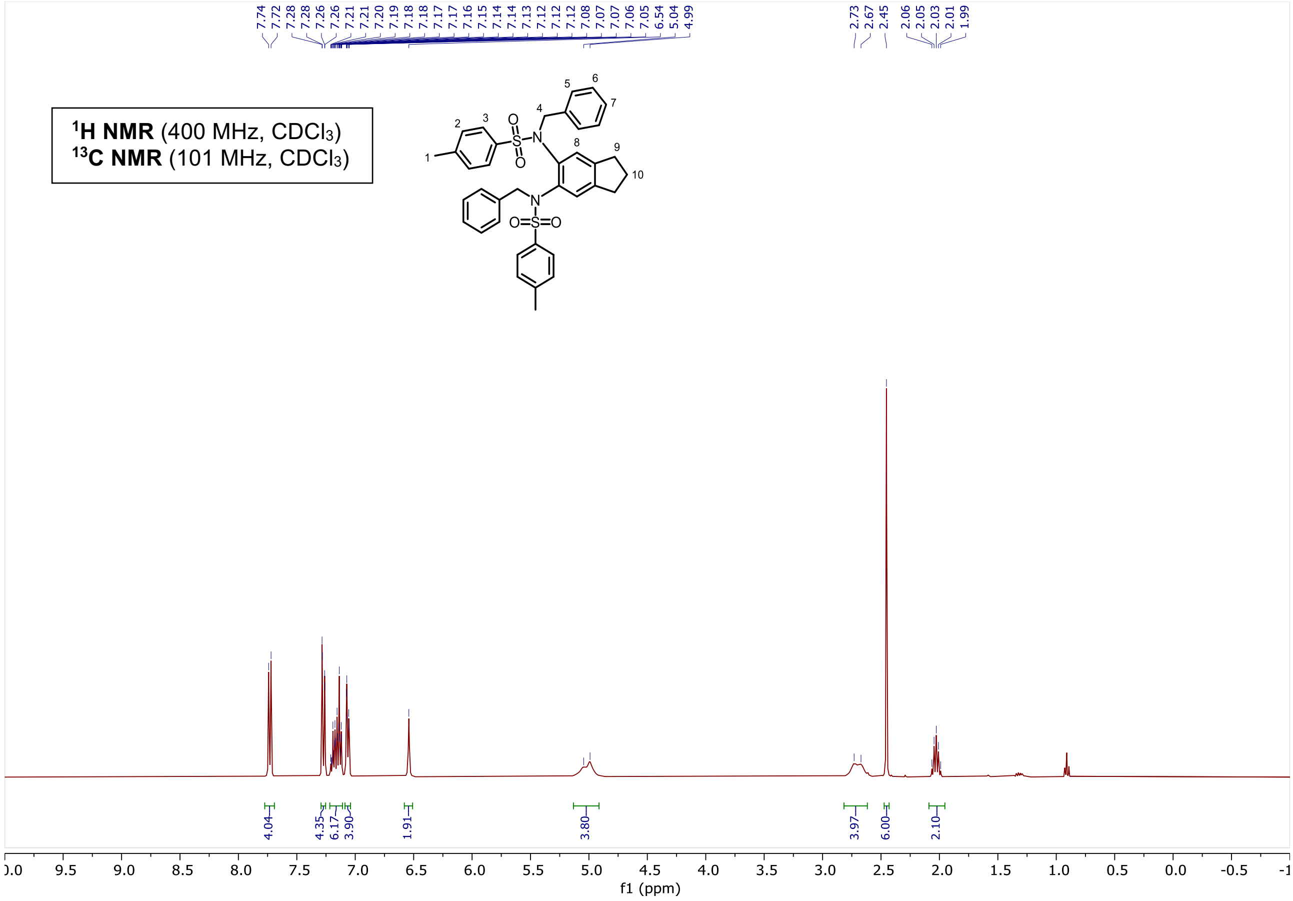

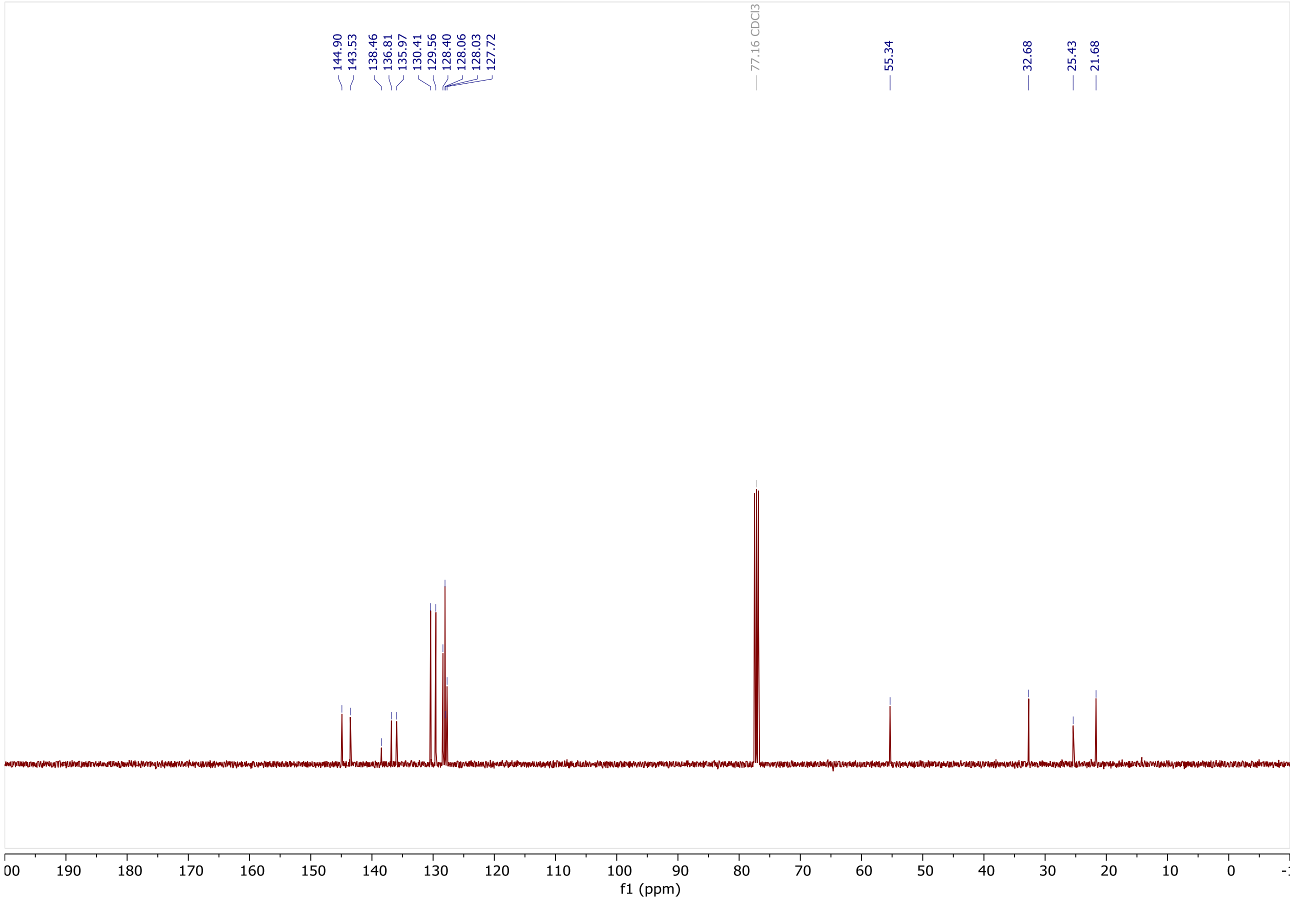

7ba Dimethyl 5,6-bis((*N*-butyl-4-methylphenyl)sulfonamido)-1,3-dihydro-2H-indene-2,2-dicarboxylate

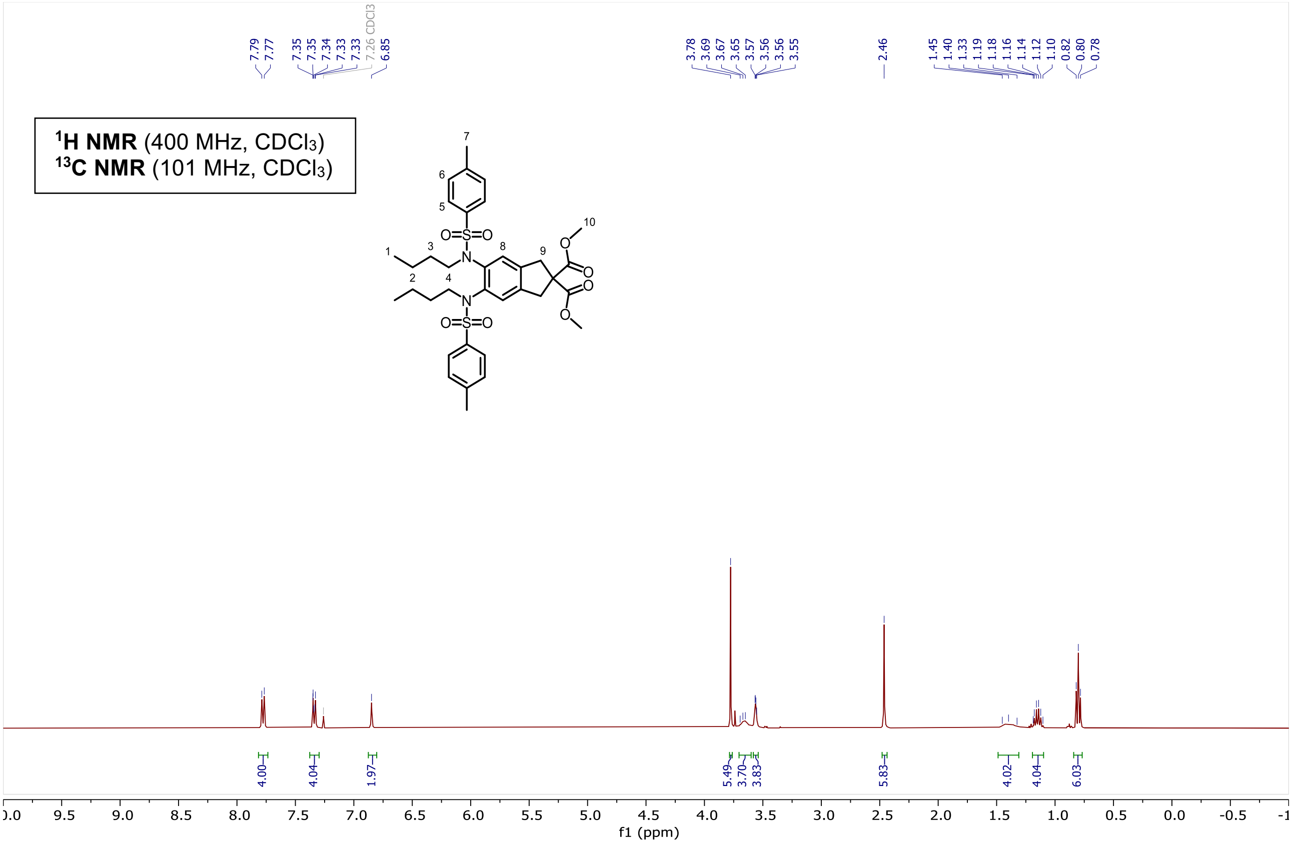

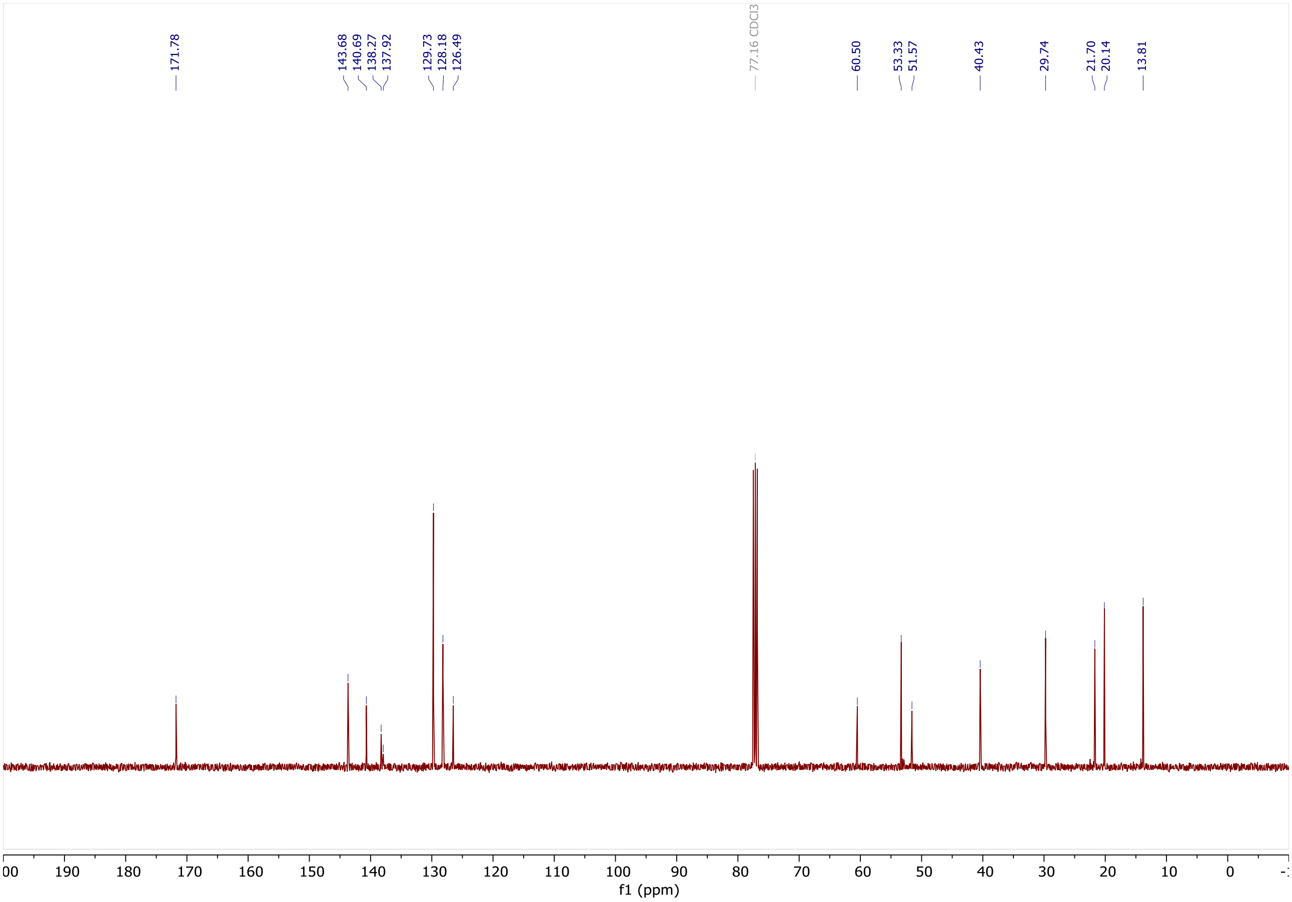

7ca *N,N'*-(2-Tosylisoindoline-5,6-diyl)bis(*N*-butyl-4-methylbenzenesulfonamide)

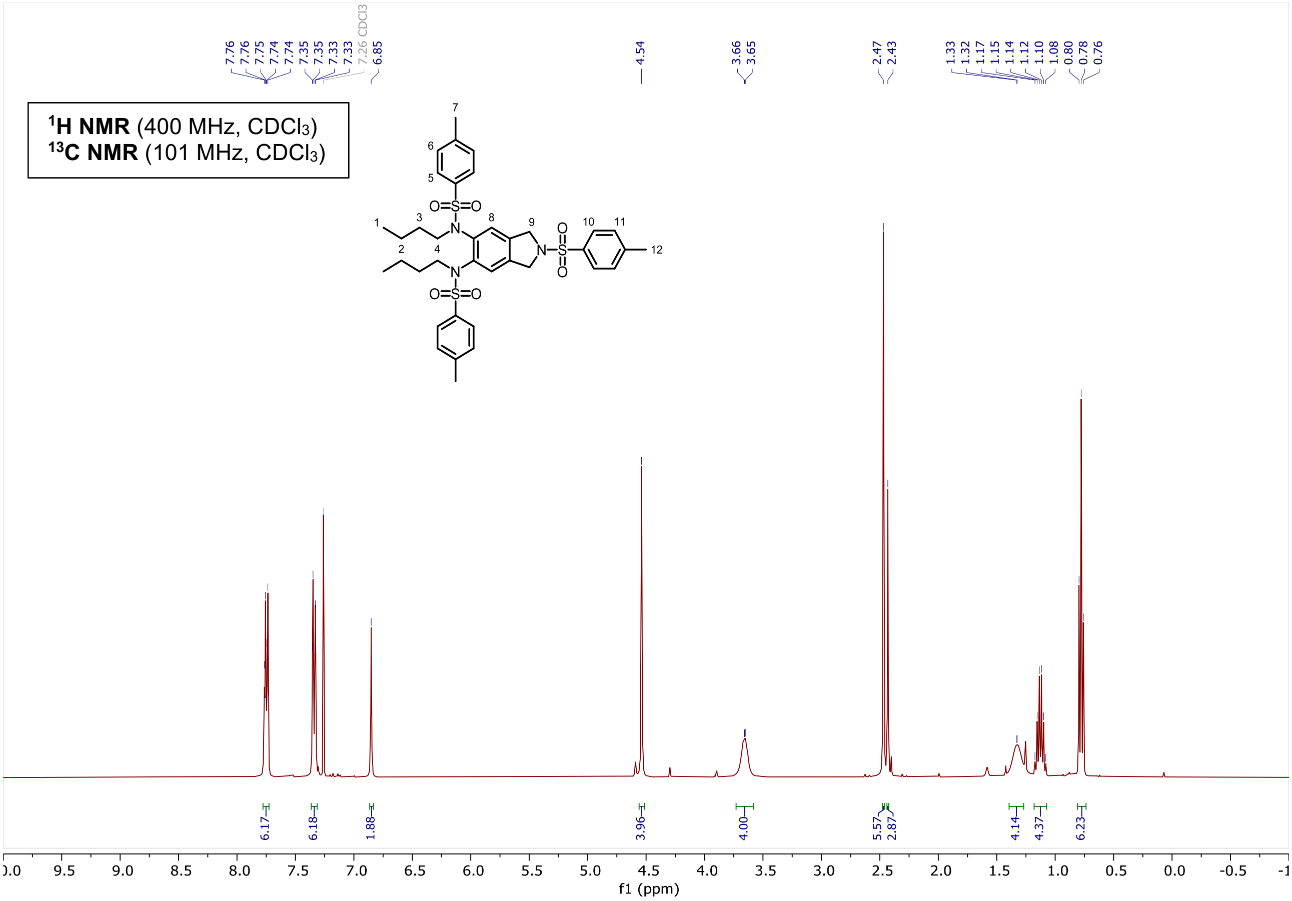

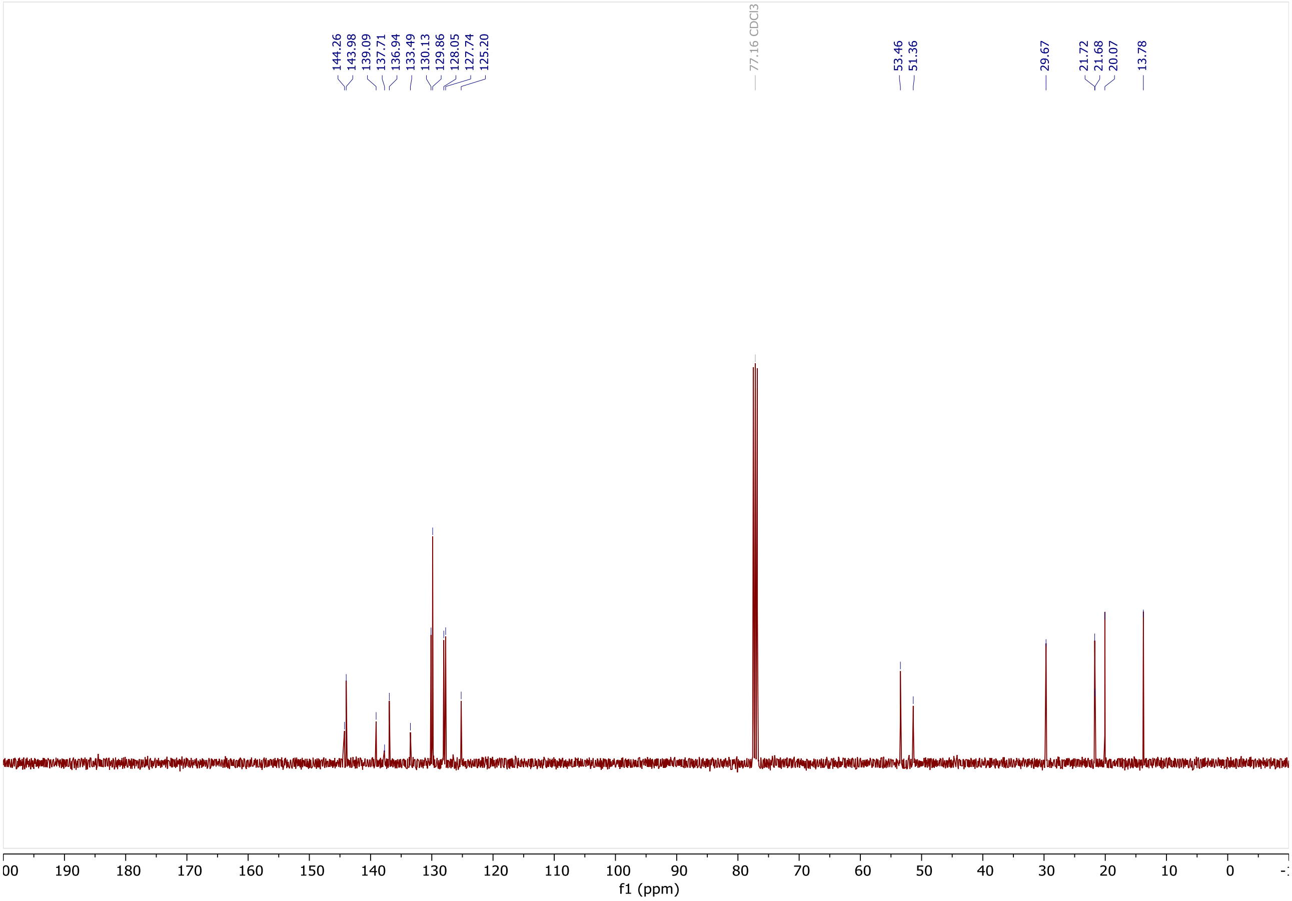

7ea ***N,N'*-(2,2-Dioxido-1,3-dihydrobenzo[c]thiophene-5,6-diyl)bis(*N*-butyl-4-methylbenzenesulfonamide)**

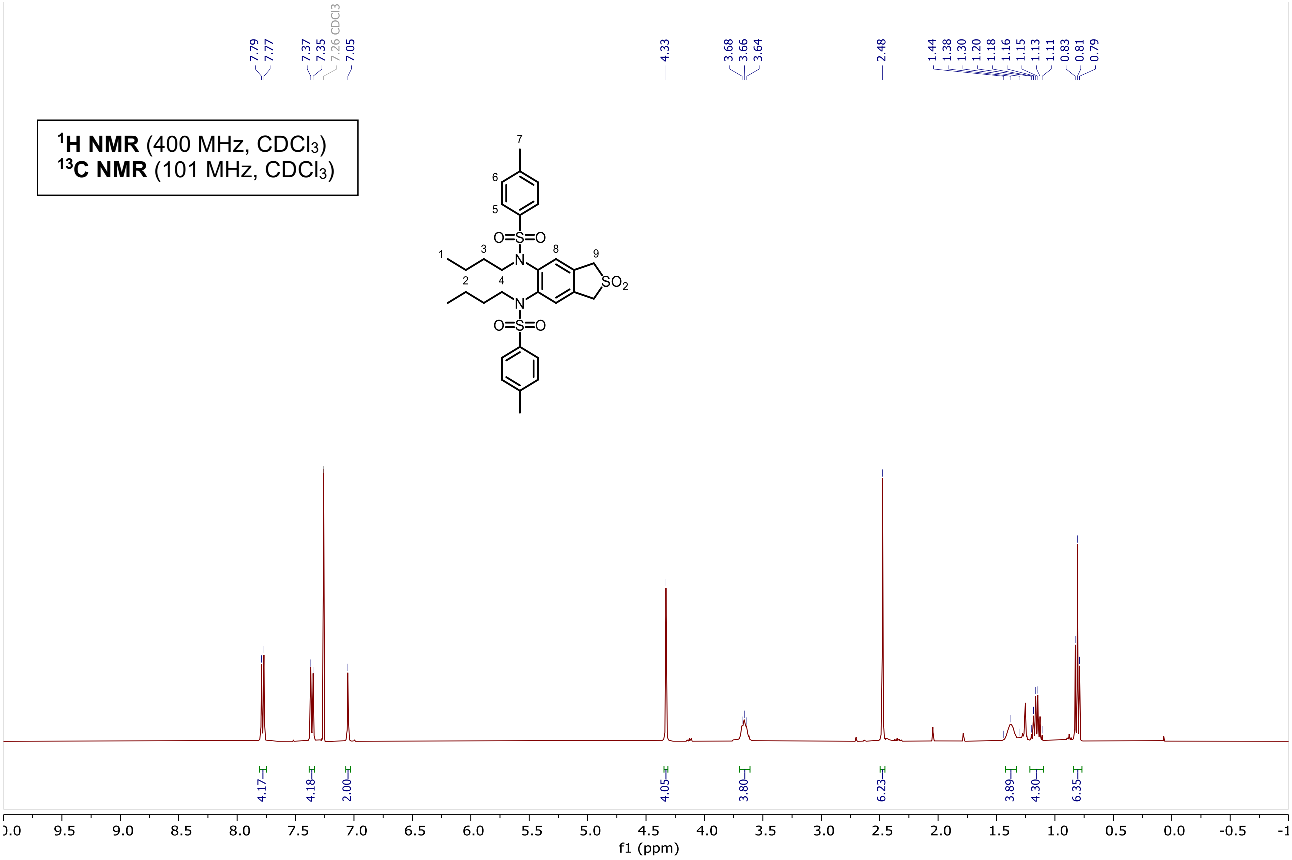

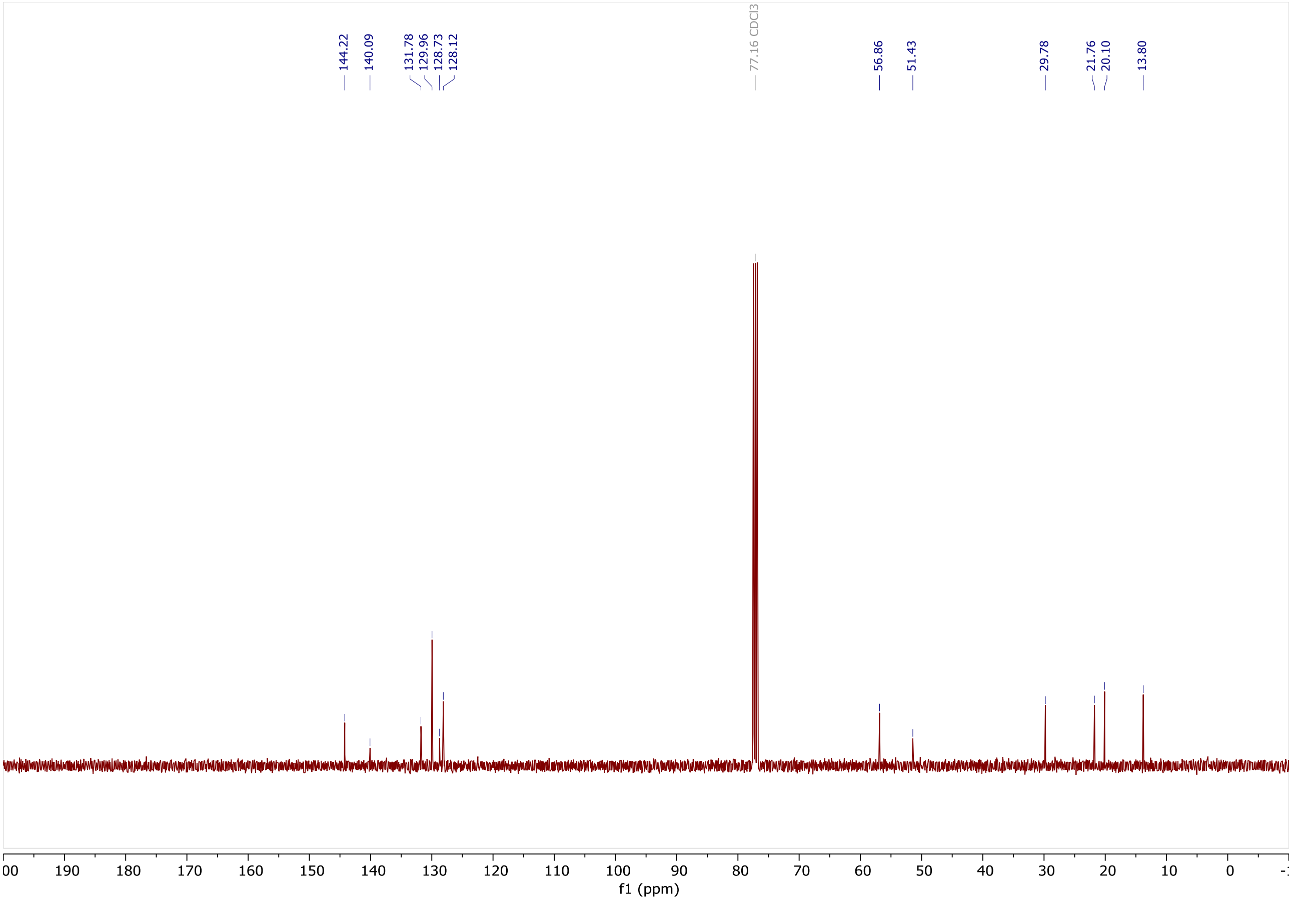

7da *N*,*N'*-(5,6,7,8-Tetrahydronaphthalene-2,3-diyl)bis(N-butyl-4-methylbenzenesulfonamide)

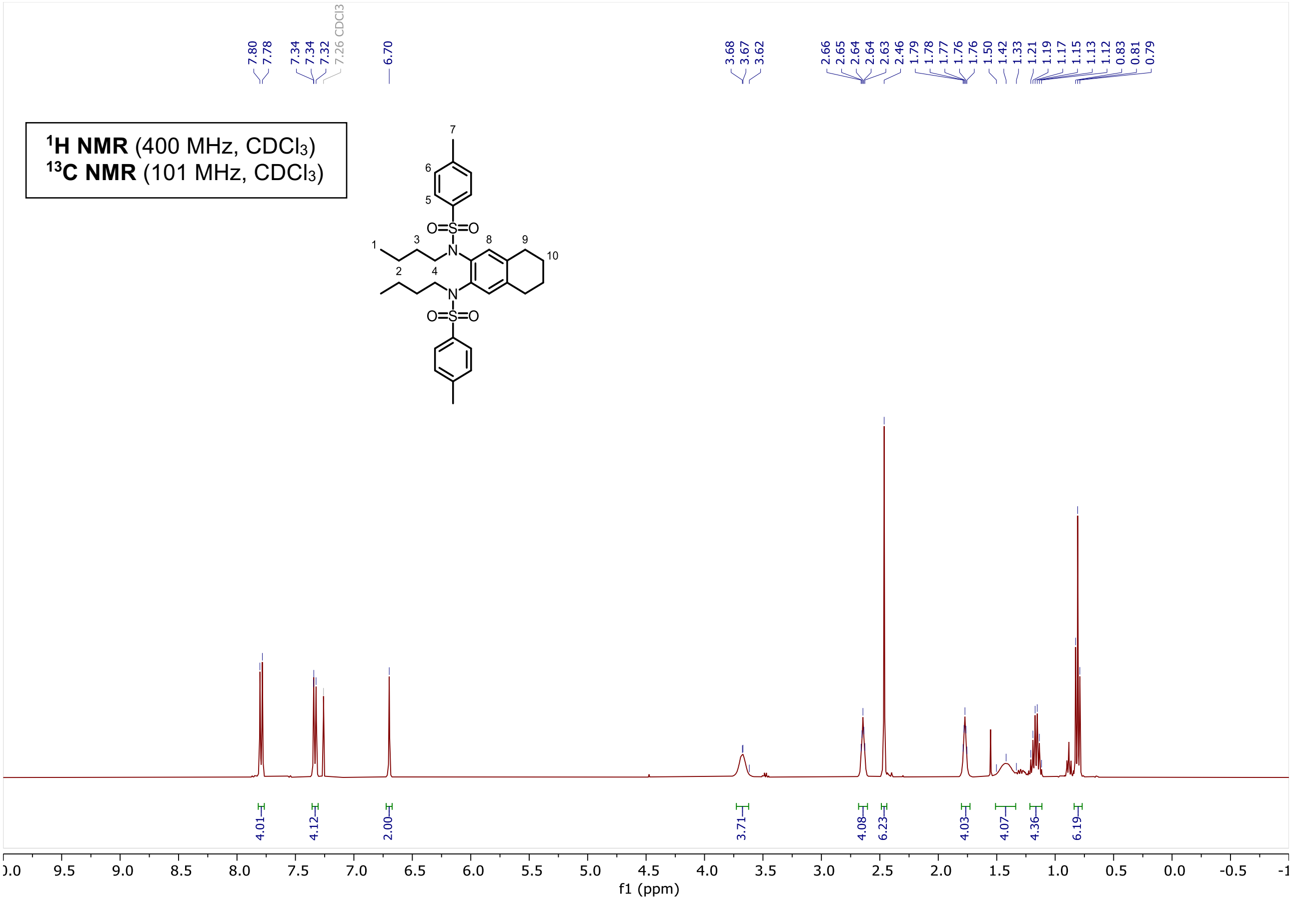

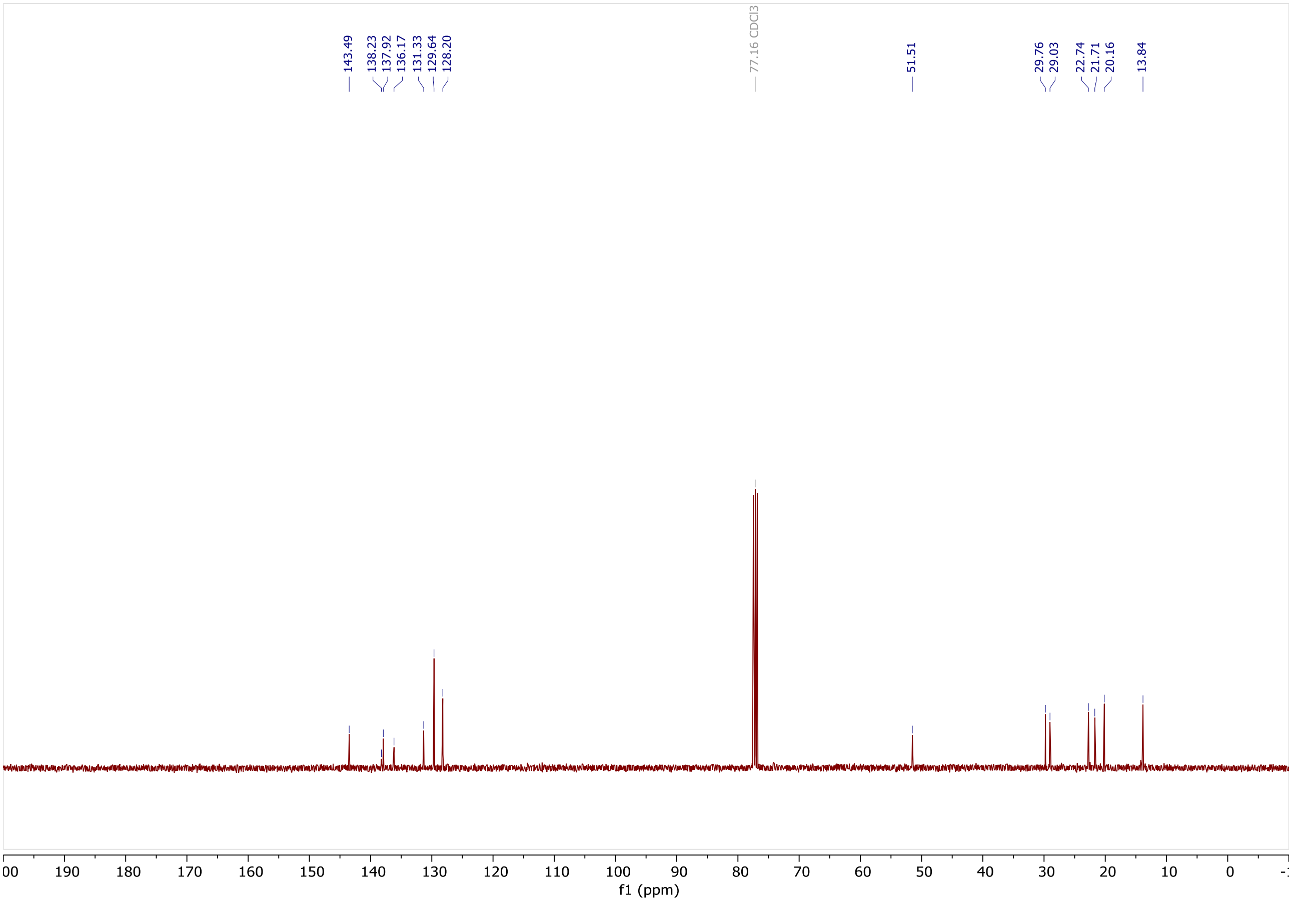

7ac *N*-(6-(9H-Carbazol-9-yl)-2,3-dihydro-1H-inden-5-yl)-*N*-butyl-4-methylbenzenesulfonamide

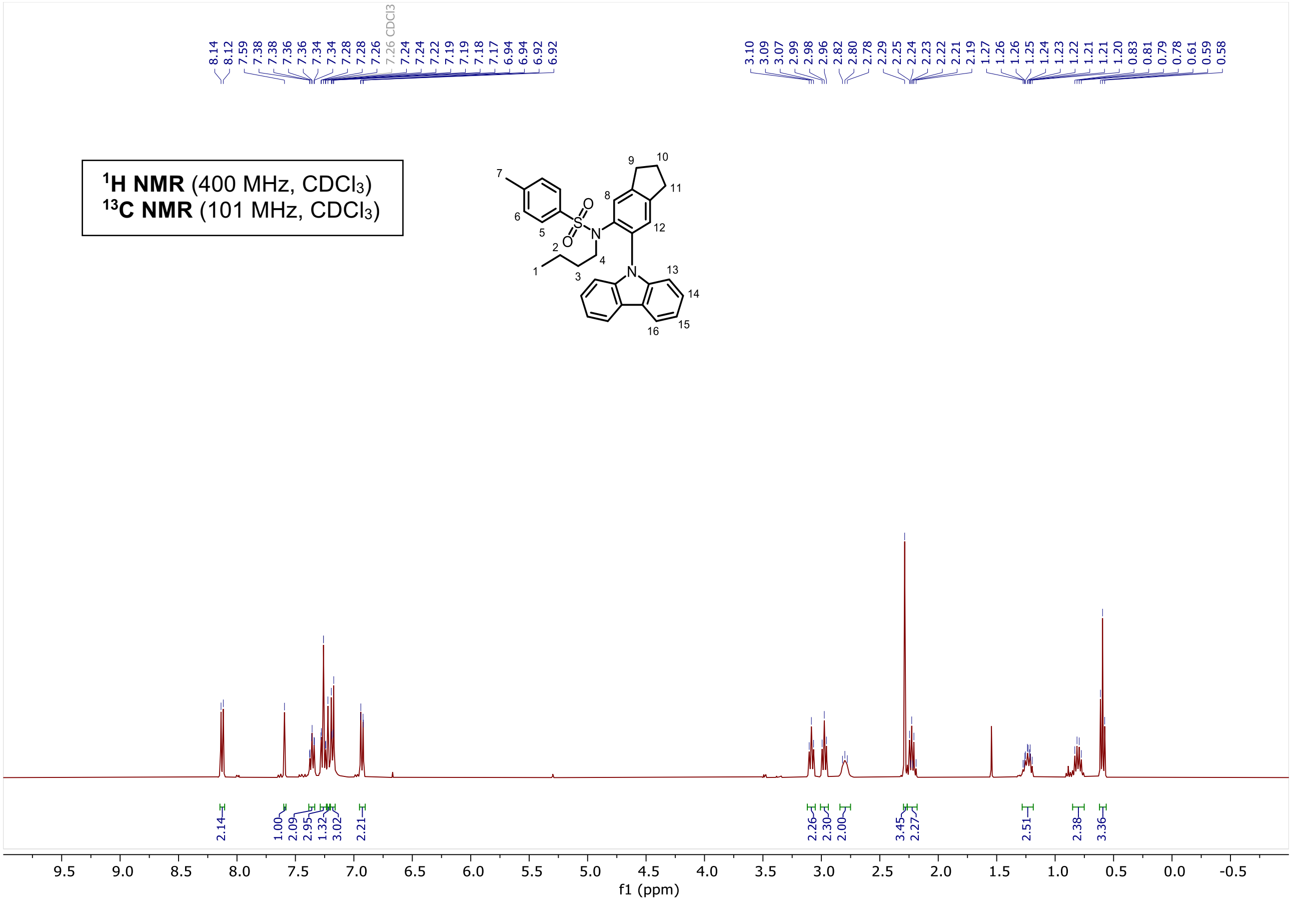

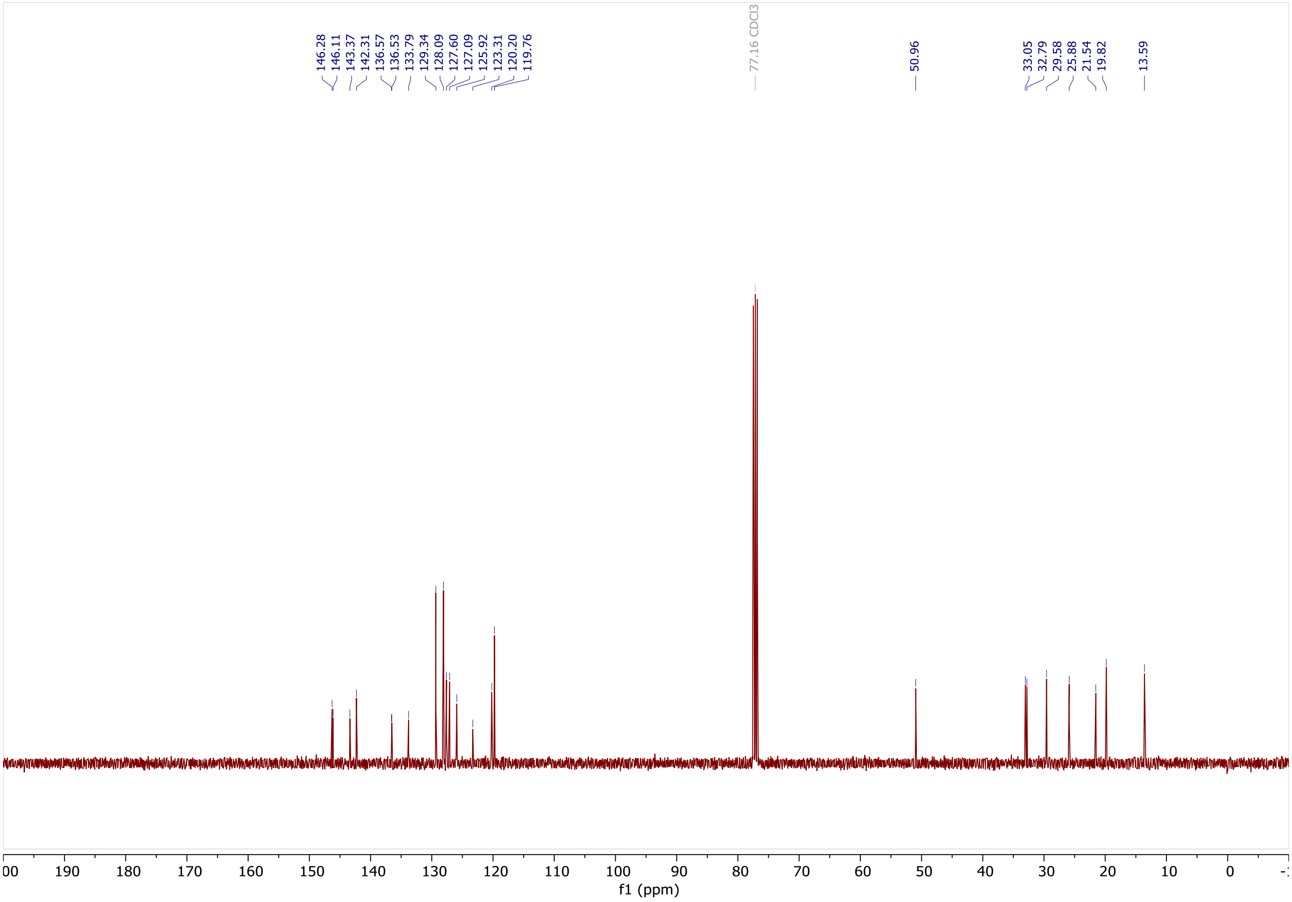

7ad *N*-(6-(9H-Carbazol-9-yl)-2,3-dihydro-1H-inden-5-yl)-*N*-benzyl-4-methylbenzenesulfonamide

^{1}H NMR (500 MHz, $CDCl_3$)
^{13}C NMR (126 MHz, $CDCl_3$)

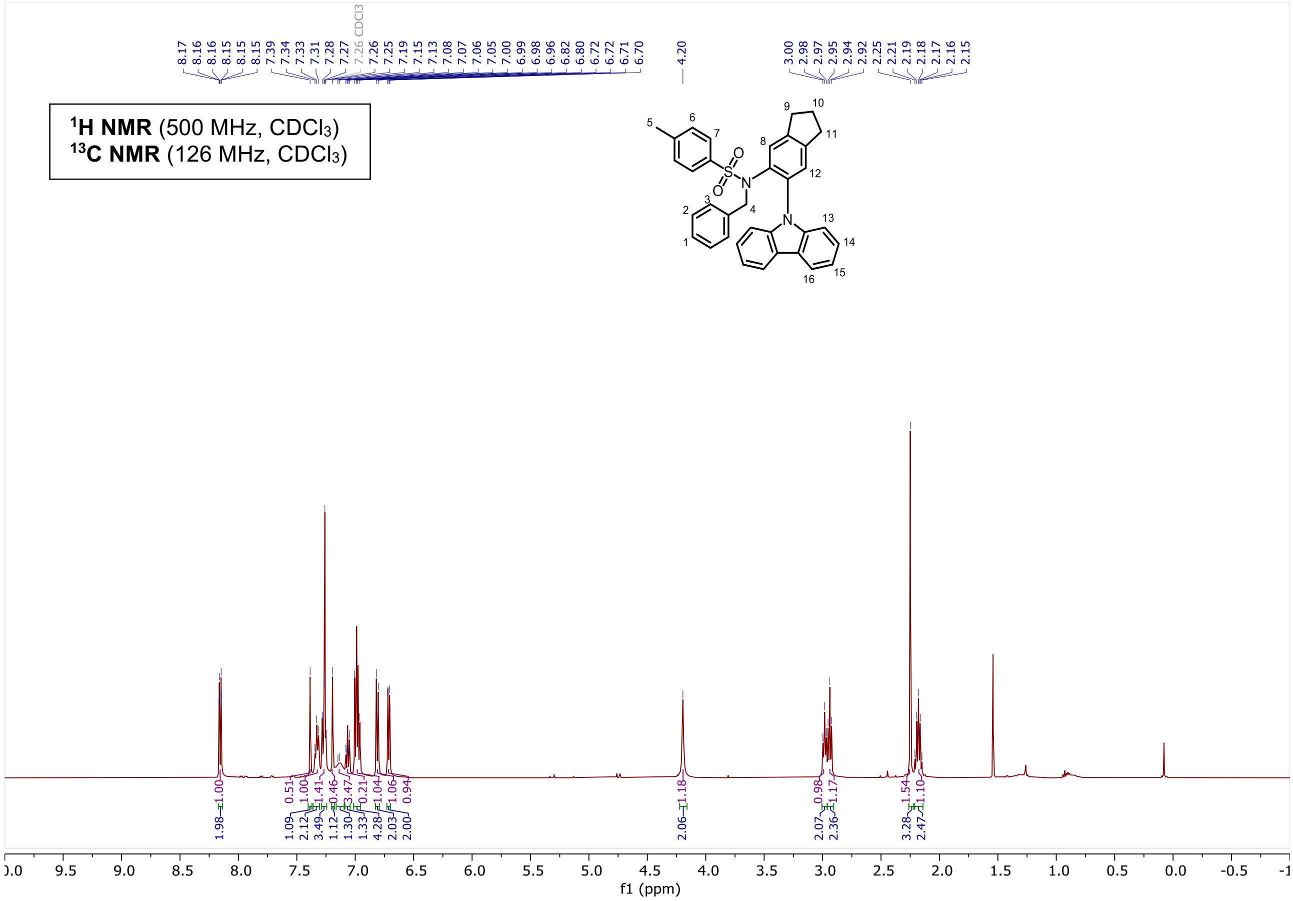

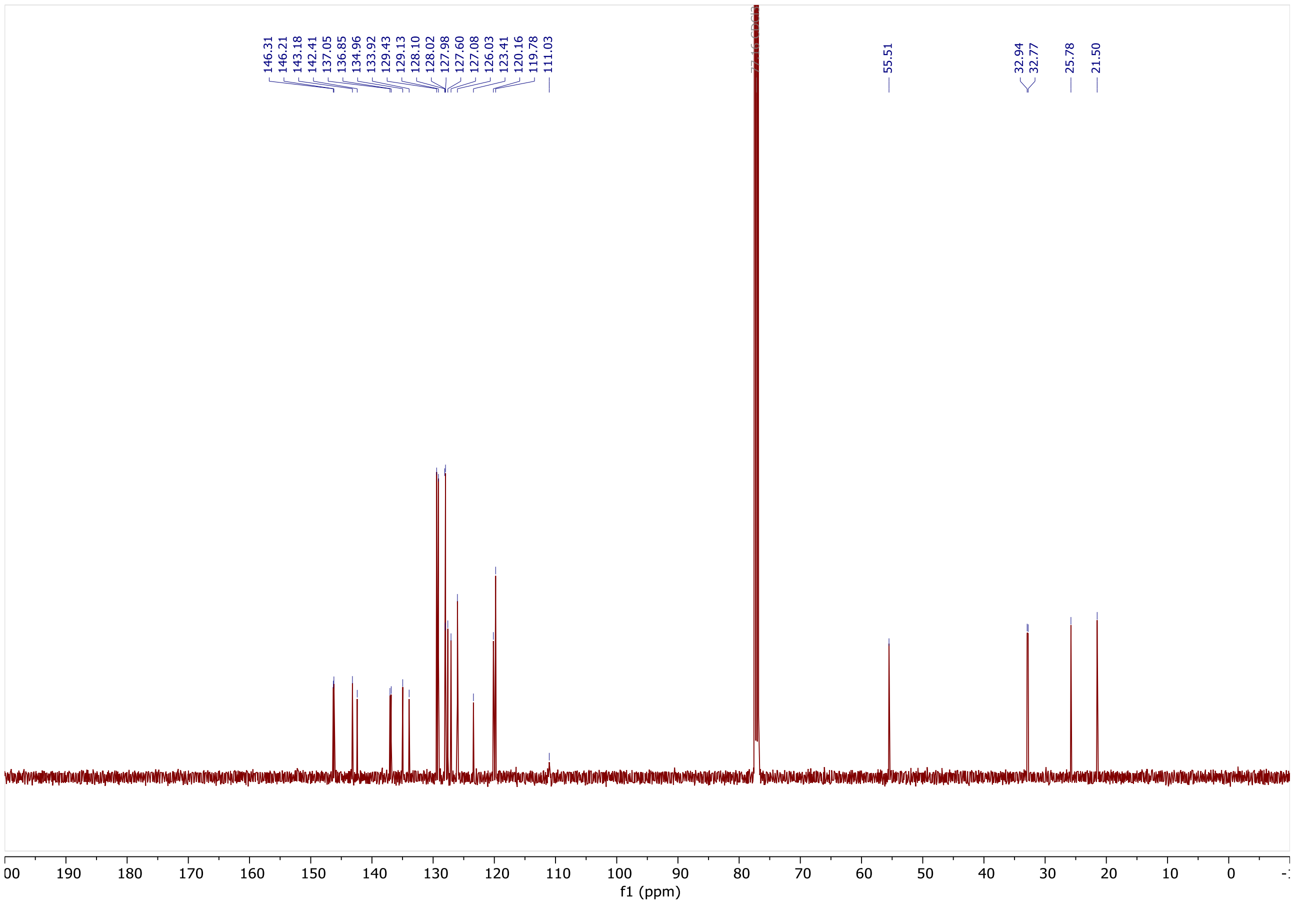

7ae *N*-(6-(9H-Carbazol-9-yl)-2,3-dihydro-1H-inden-5-yl)-*N*-isopropyl-4-methylbenzenesulfonamide

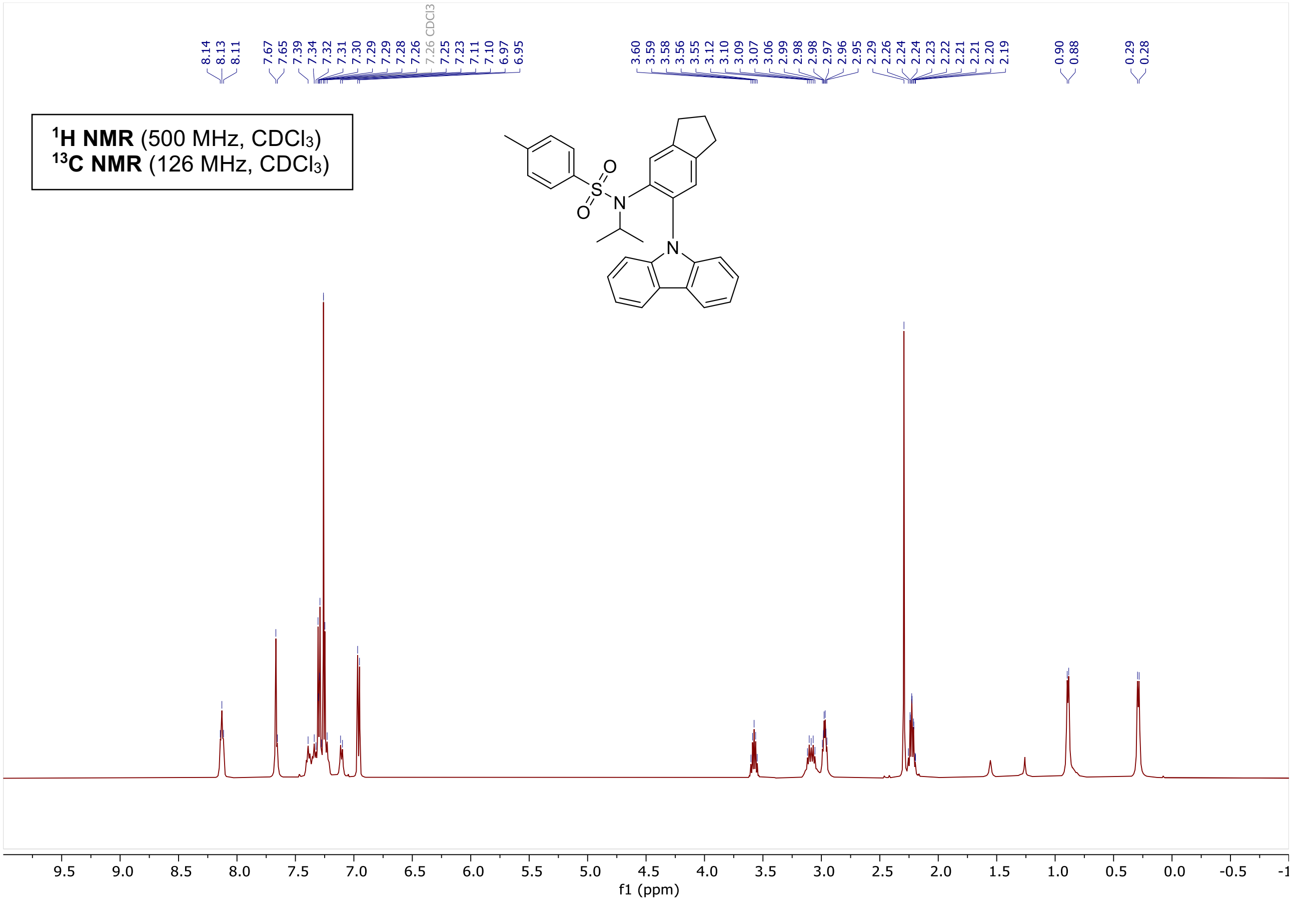

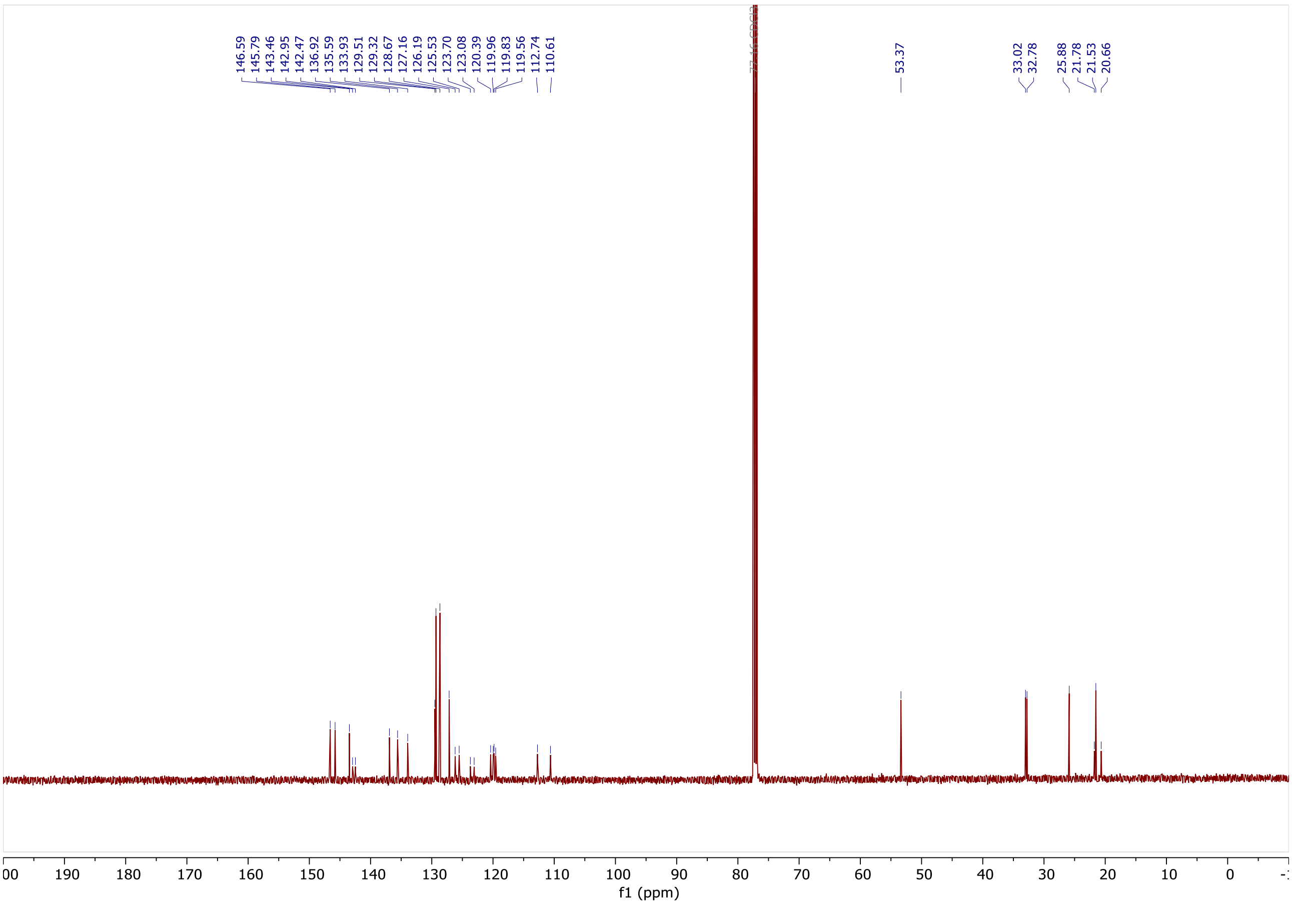

7af *N*-(6-(9*H*-carbazol-9-yl)-2,3-dihydro-1H-inden-5-yl)-*N*-cyclohexyl-4-methylbenzenesulfonamide

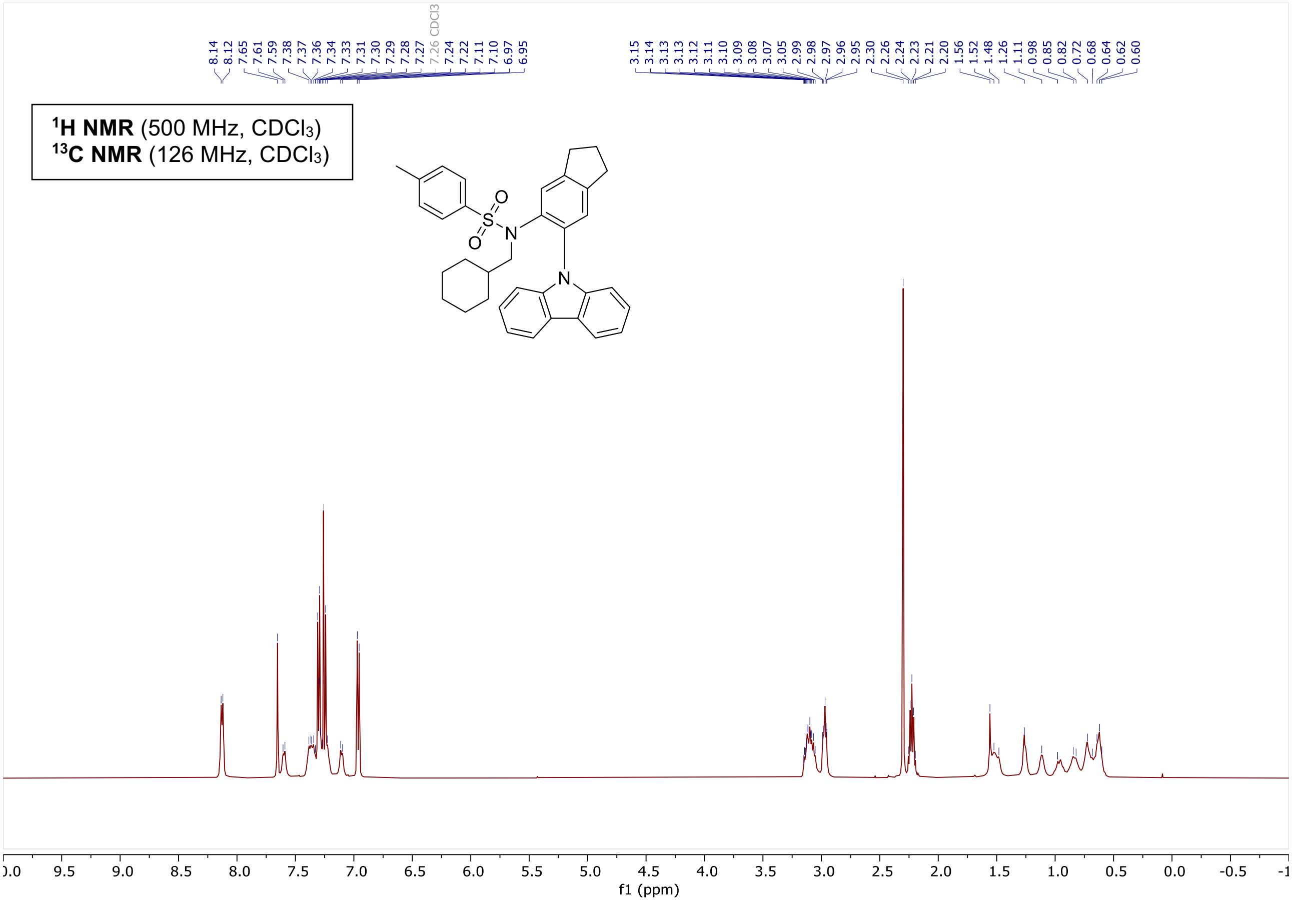

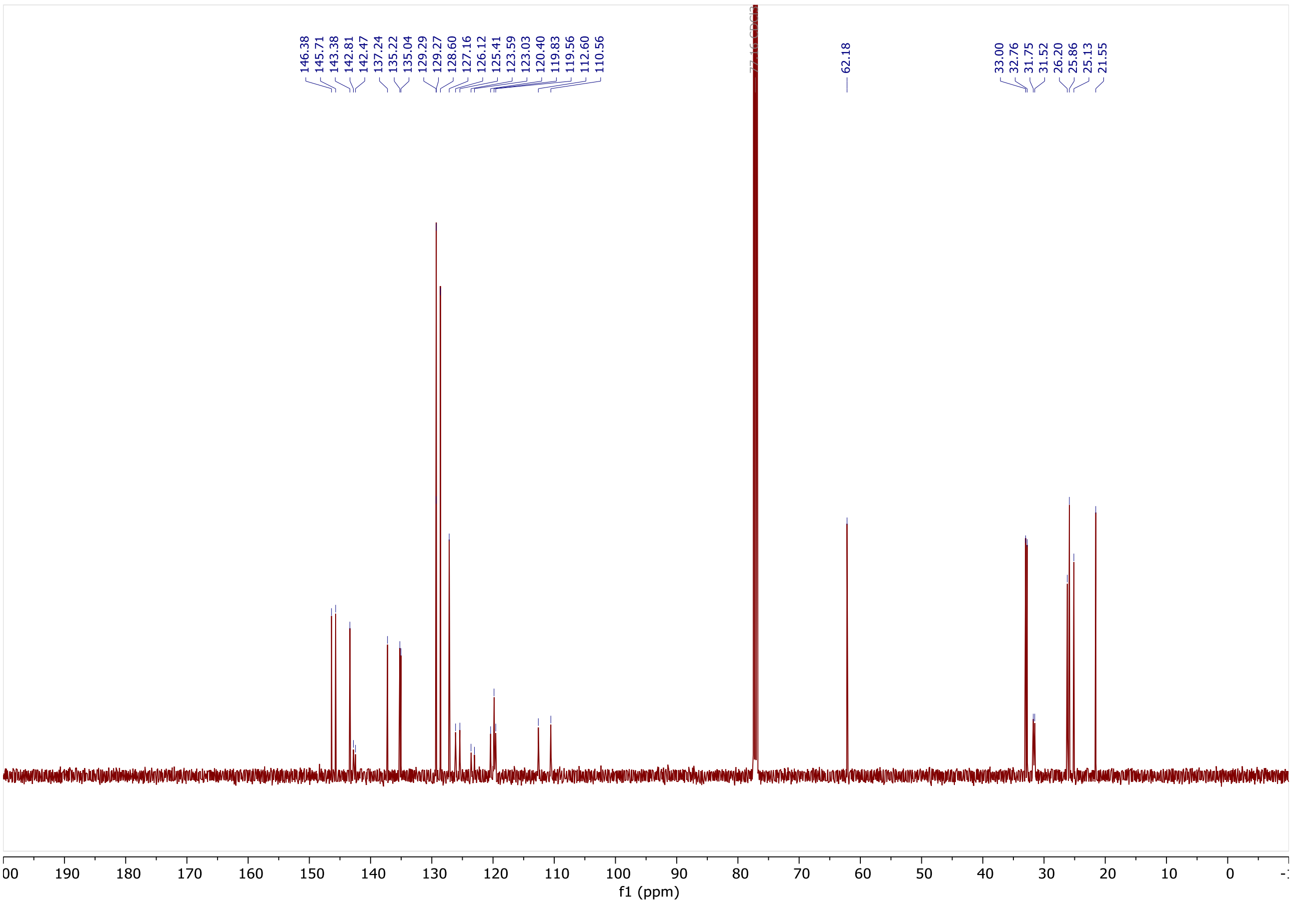

7bc Dimethyl 5-((*N*-butyl-4-methylphenyl)sulfonamido)-6-(9H-carbazol-9-yl)-1,3-dihydro-2H-indene-2,2-dicarboxylate

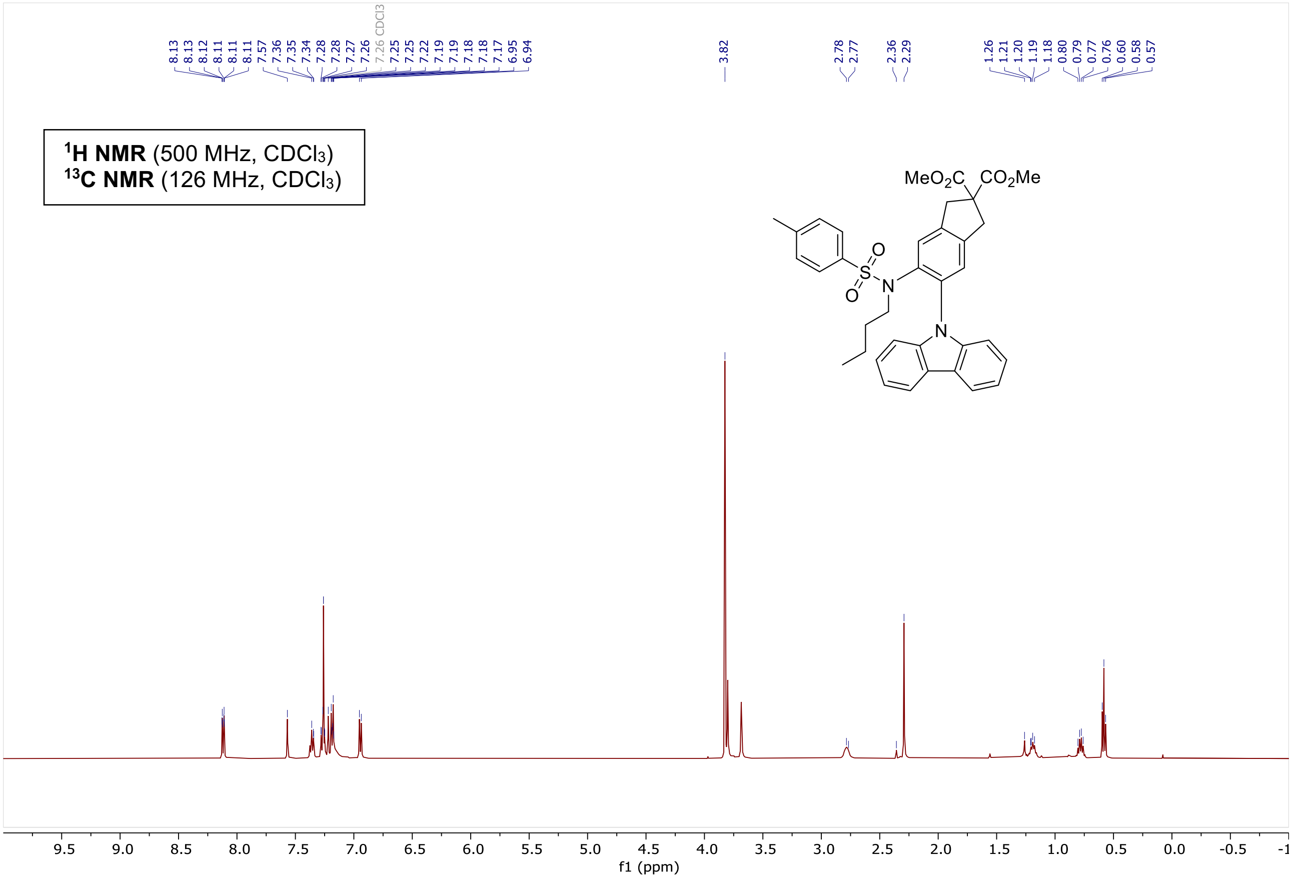

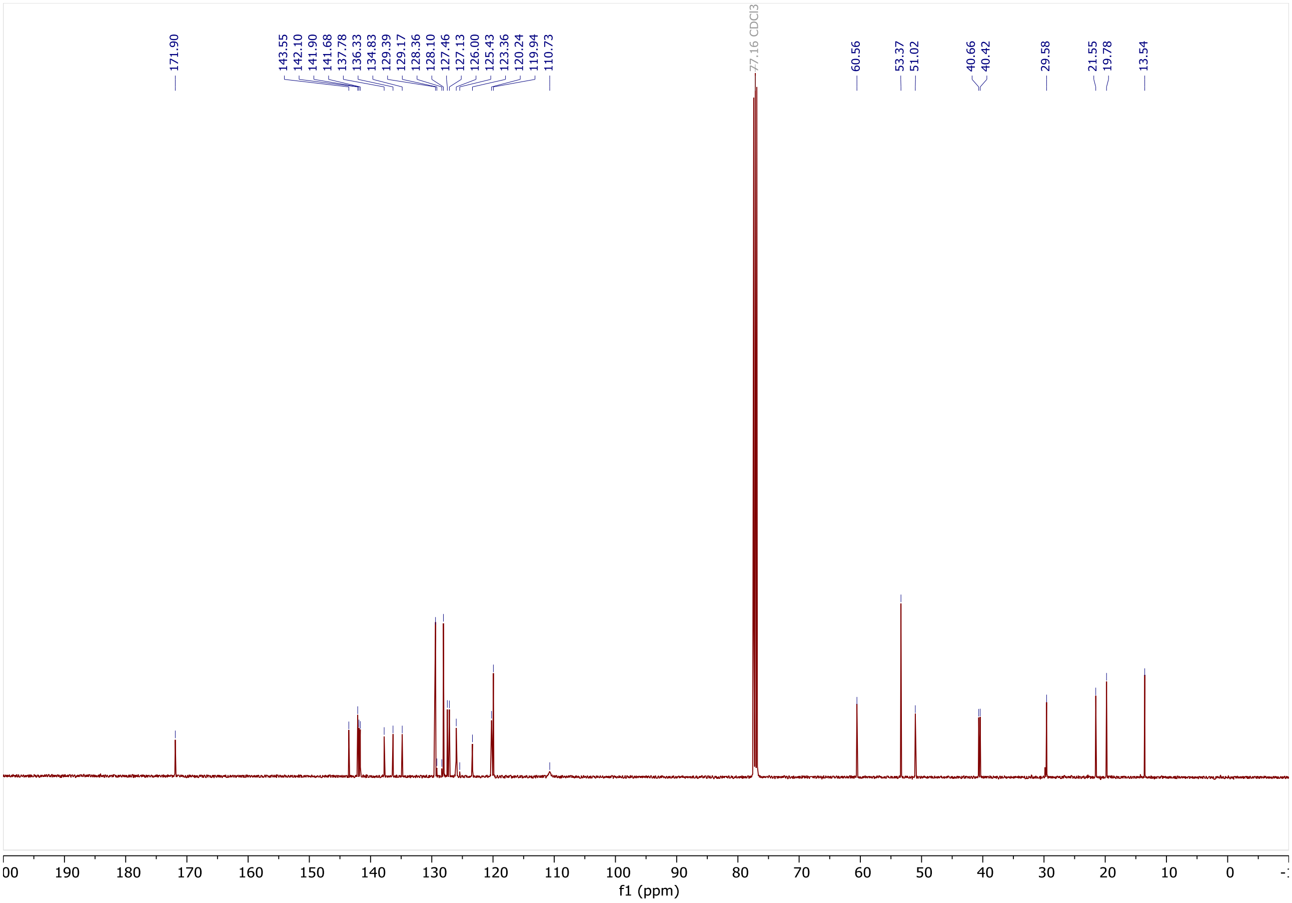

7cc ***N*-(6-(9*H*-Carbazol-9-yl)-2-tosylisoindolin-5-yl)-*N*-butyl-4-methylbenzenesulfonamide**

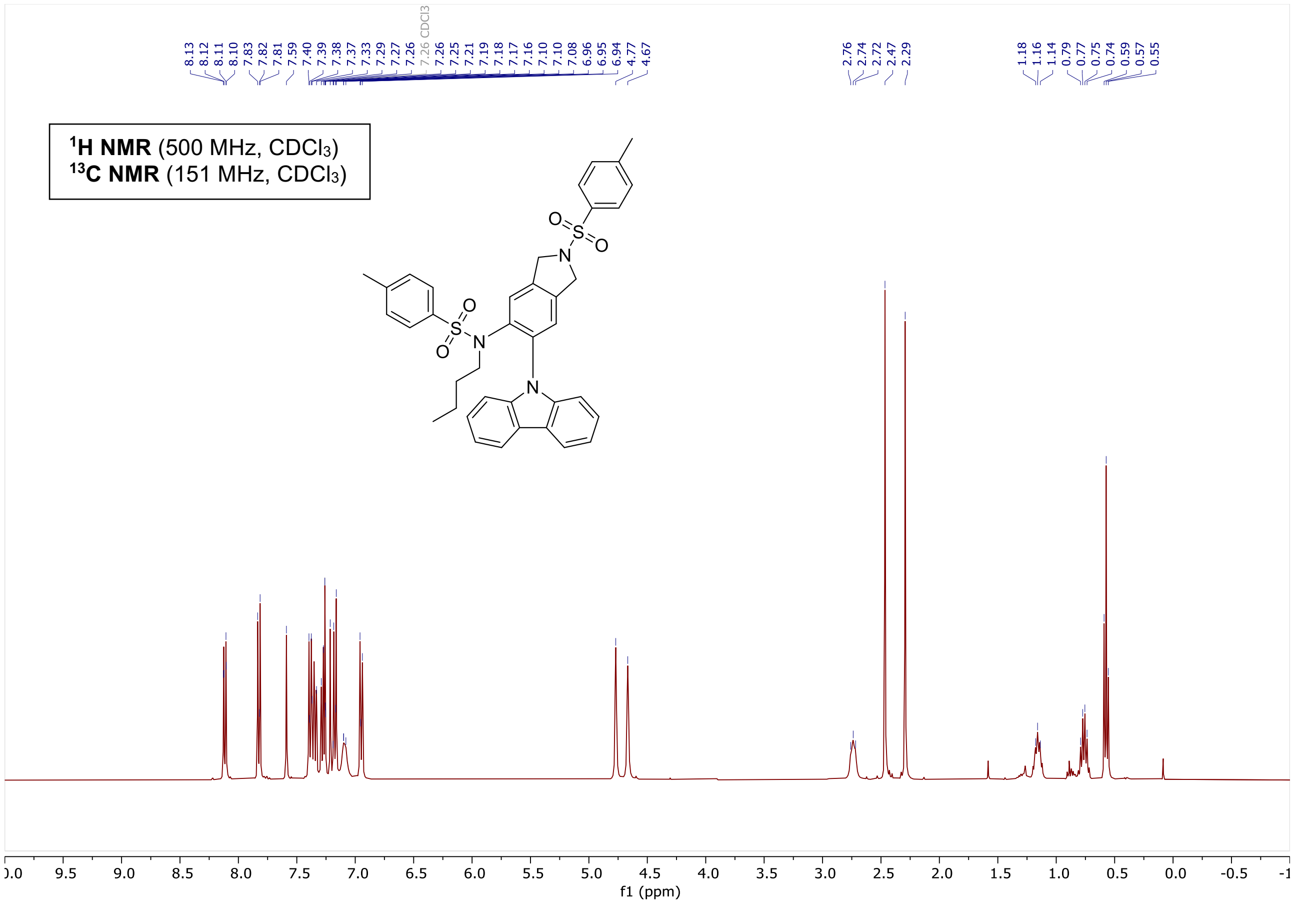

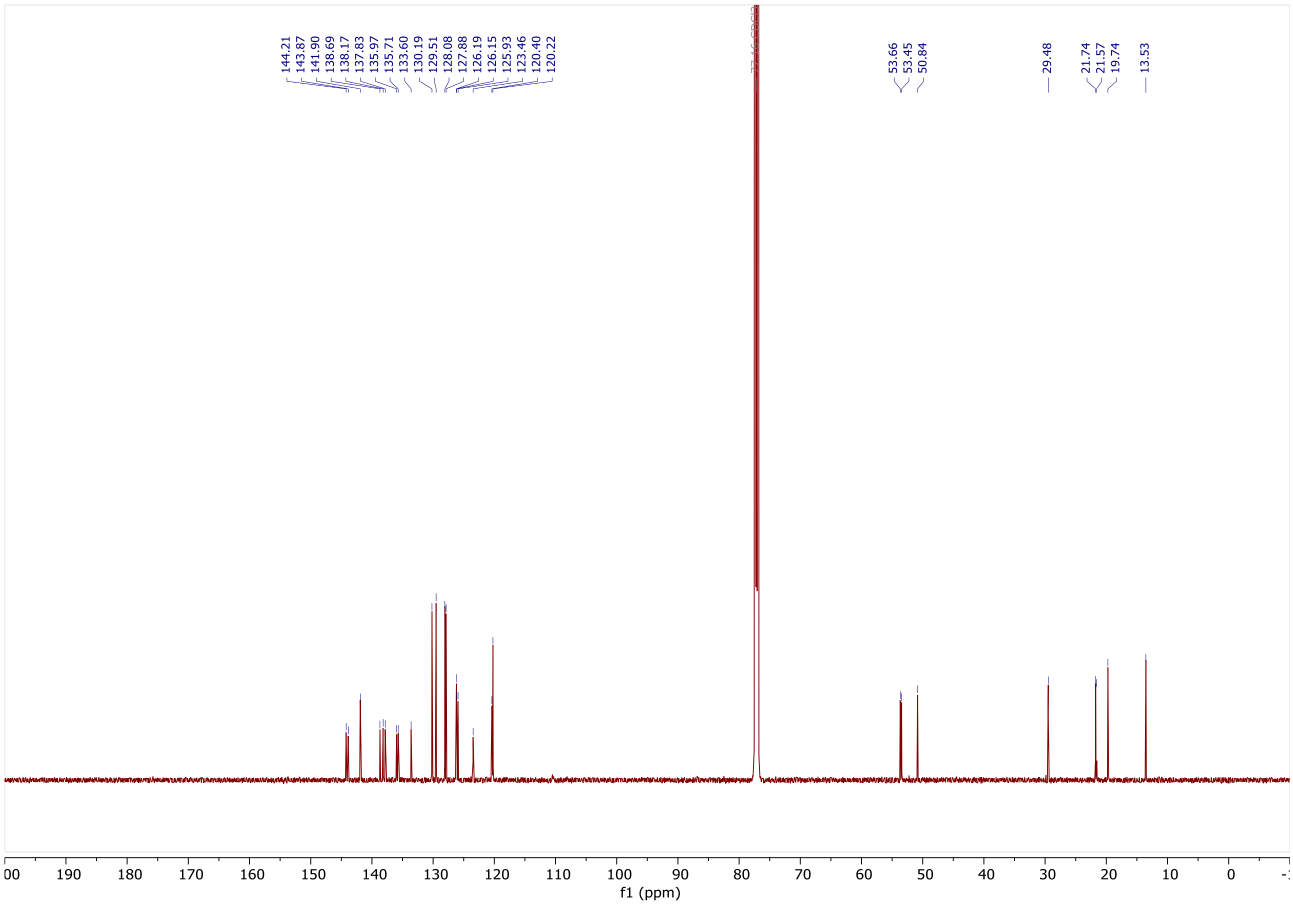

Dynamic HPLC (DHPLC)

DHPLC was performed on a Dionex Ultimate 3000 system comprising of a Dionex LPG-3400SD pump, WPS-3000SL autosampler, TCC-3000SD column compartment fitted with the appropriate Daicel Chiralpak column (dimensions: 0.46 cm ø × 25 cm), corresponding guard column (0.4 cm ø × 1 cm), and a 3 µL heat exchanger, and a DAD-3000RS diode array detector. Wavelengths (λ) are reported in nm, retention times (τ_R) are reported in minutes and solvent flow rates are reported in mL min^{-1}. Chromatograms were analysed using DCXplorer MMXVII[5] and barriers were calculated using the method described by Mayor and co-workers.[6]

2aa

DHPLC measurements were performed with a Chiralpak® IC (250 mm, i.d. 4.6 mm, particle size 5 µm) column (n-hexane:ethanol, 70:30, 1.0 mL/min) at temperatures between 15.0 and 50.0 °C.

T [ºC]	tR1 [min]	tR2 [min]	wh1 [s]	wh2 [s]	h1 [%]	hp [%]	h2 [%]	A1	A2	k1 [1/s]
15	11.18	35.09	28.0	60.4	100	0.53	0.42	50.82	49.18	1.83E-04
	11.17	35.09	28.0	58.8	100	0.50	0.40	50.77	49.23	1.72E-04
	11.17	35.06	27.8	60.6	100	0.50	0.44	50.71	49.29	1.75E-04
20	10.49	28.55	22.8	81.2	100	0.93	40.48	50.05	49.95	3.00E-04
	10.48	28.53	22.8	81.4	100	0.94	40.43	50.03	49.97	3.04E-04
	10.48	28.52	22.8	80.8	100	0.92	40.45	50.07	49.93	3.00E-04
25	9.86	25.42	19.2	65.0	100	1.61	40.39	49.99	50.01	5.32E-04
	9.86	25.41	19.2	65.4	100	1.64	40.36	50.00	50.00	5.41E-04
	9.86	25.41	19.2	64.8	100	1.63	40.39	49.98	50.02	5.37E-04
30	9.31	22.71	16.8	53.0	100	2.94	41.04	49.94	50.06	9.17E-04
	9.31	22.71	16.6	52.6	100	2.94	41.08	49.88	50.12	9.27E-04
	9.31	22.71	16.6	52.8	100	2.95	41.09	49.91	50.09	9.28E-04
35	8.83	20.37	14.8	43.6	100	5.73	42.86	49.86	50.14	1.57E-03
	8.83	20.37	14.8	43.2	100	5.72	42.90	49.86	50.14	1.56E-03
	8.83	20.36	14.8	43.6	100	5.72	42.78	49.84	50.16	1.56E-03
40	8.41	18.34	13.4	36.6	100	12.67	45.88	49.76	50.24	2.65E-03
	8.40	18.33	13.4	36.6	100	12.69	45.91	49.75	50.25	2.66E-03
	8.40	18.32	13.2	36.6	100	12.69	45.89	49.76	50.24	2.68E-03
45	8.01	16.55	12.2	32.0	100	34.12	51.45	49.57	50.43	4.58E-03
	8.02	16.56	12.2	32.0	100	34.13	51.26	49.63	50.37	4.58E-03
	8.02	16.56	12.2	32.0	100	34.08	51.22	49.60	50.40	4.58E-03
50	7.72	15.05	11.8	30.0	77.7	100	48.24	49.11	50.89	7.68E-03
	7.72	15.03	11.6	30.2	76.74	100	48.04	48.90	51.10	7.70E-03
	7.72	15.03	11.6	30.2	77.02	100	47.93	49.03	50.97	7.71E-03

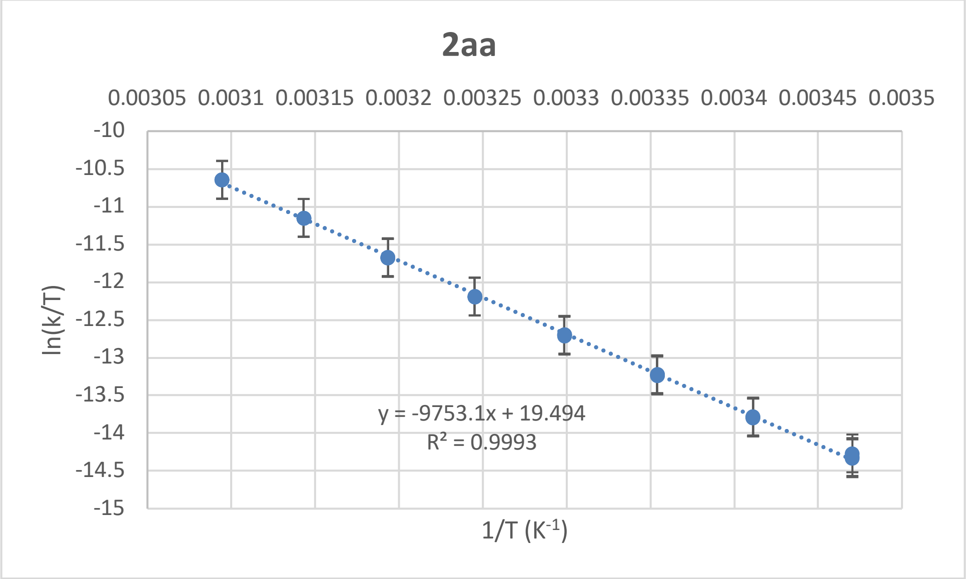

dH (kcal/mol)	19.76
dS (cal/mol)	-7.12
dG (kcal/mol)	21.89
$T_{1/2}$ (min) @ 25°C	21.0

References

[1] S. J. Mansfield, K. E. Christensen, A. L. Thompson, K. Ma, M. W. Jones, A. Mekareeya, E. A. Anderson, *Angew. Chemie - Int. Ed.* **2017**, *56*, 14428–14432.

[2] T. G. Schenck, J. M. Downes, C. R. C. Milne, P. B. Mackenzie, H. Boucher, J. Whelan, B. Bosnich, *Inorg. Chem.* **1985**, *24*, 2334–2337.

[3] P. J. Smith, Y. Jiang, Z. Tong, H. D. Pickford, K. E. Christensen, J. Nugent, E. A. Anderson, *Org. Lett.* **2021**, *23*, 6547–6552.

[4] Z. Tong, O. L. Garry, P. J. Smith, Y. Jiang, S. J. Mansfield, E. A. Anderson, *Org. Lett.* **2021**, *23*, 4888–4892.

[5] M. Rickhaus, L. Jundt, M. Mayor, *Chimia (Aarau).* **2016**, *70*, 192–202.

[6] O. Trapp, *J. Chromatogr. B Anal. Technol. Biomed. Life Sci.* **2008**, *875*, 42–47.
